# Development of a Multilayered Prognostic Model for Wilms’ Tumor Based on Characteristic Lymphocyte Genes

**DOI:** 10.1155/genr/1964582

**Published:** 2025-12-18

**Authors:** Zexi Li, Jing Liu, Yurui Wu

**Affiliations:** ^1^ Department of Thoracic Surgery and Oncology, Capital Center for Children’s Health, Capital Medical University, Capital Institute of Pediatrics, Beijing, China, shouer.com.cn

**Keywords:** clinical indicators, genetic, lymphocytes, prognostic nomogram, Wilms’ tumor

## Abstract

**Objective:**

To develop a prognostic nomogram for Wilms’ tumor (WT) integrating genetic and clinical factors to improve evaluation accuracy and clinical utility.

**Methods:**

RNA sequencing (RNA‐seq) data from 125 WT patients and single‐cell RNA (scRNA‐seq) data from 2437 samples were analyzed using bioinformatics tools for data processing, including normalization and scaling with SCTransform, and cell clustering with Seurat. Principal component analysis (PCA) and Uniform Manifold Approximation and Projection (UMAP) were utilized for data visualization. Differential gene expression analysis identified pivotal genes for the Genetic Feature Prognostic Model for WT (GPM‐WT). Univariate Cox regression analysis refined this model by incorporating clinical prognostic indicators. Survival analysis, Cox regression, and ROC curve assessments evaluated these models’ prognostic capabilities. Immune cell infiltration and drug sensitivity were quantified, linking these to patient risk categories.

**Results:**

Six prognostic lymphocyte genes (*KLRC1*, *APOC2*, *GBP2*, *SLA*, *MLLT3*, and *SIGLEC5*) were identified for GPM‐WT. Clinical factors, age and sex, were integrated to refine the model. The Lymphocyte Gene and Clinical Features Prognostic Nomogram (LGCPN‐WT) effectively distinguished high from low‐risk groups, predicting 2–5‐year survival rates with area under the curve (AUC) values of 0.771, 0.774, 0.751, and 0.785. Elevated immune cell infiltration and enhanced drug sensitivity characterized the high‐risk group, exhibiting significant responsiveness to chemotherapy, targeted, and immunotherapy treatments (*p* < 0.05).

**Conclusions:**

The study developed an integrated LGCPN‐WT model, significantly enhancing survival prediction accuracy and clinical utility for WT, thus supporting personalized treatment approaches.

## 1. Introduction

Wilms’ tumor (WT) is the predominant renal malignancy in children, accounting for more than 90% of all pediatric kidney cancers, and is most commonly diagnosed between the ages of two and four [1, 2]. WT typically manifests as a rapidly growing abdominal mass. If left untreated, it can quickly spread beyond the kidney and metastasize to distant organs, including the lungs and liver, via the bloodstream [3, 4]. Research studies [5, 6] show that the survival rates for patients with untreated WT significantly decline, especially in the disease’s advanced stages. However, early diagnosis and treatment can lead to a 5‐year survival rate of over 90% [7]. Consequently, accurate prognostic assessments are essential to guide early interventions and develop personalized treatment plans, enhancing treatment success and quality of life.

The pathogenesis of WT involves complex genetic and molecular changes that directly influence the development and proliferation of renal cells [8, 9]. Key genetic factors are crucial in regulating the development of the kidneys and reproductive system. For example, mutations in the *WT1* gene frequently associate with Denys–Drash syndrome, and mutations in *WT2* are linked to Beckwith–Wiedemann syndrome [10]. Additionally, mutations in the *CTNNB1* gene can activate the Wnt signaling pathway, affecting cell proliferation and differentiation [11]. Furthermore, abnormalities in genetic imprinting, such as changes in *IGF2*, significantly affect tumor development [12].

The common imaging techniques currently used to diagnose WT are ultrasound, computed tomography, and magnetic resonance imaging. These techniques assess the tumor’s location, size, and presence of metastasis [13, 14]. Histopathological analysis is still considered the gold standard for clinical diagnosis [15]. Despite their effectiveness, these diagnostic methods primarily focus on the tumor’s morphological characteristics and anatomical position. They have limited capacity to reveal molecular features or predict disease progression and prognosis. This limitation highlights the need for more advanced diagnostic methods, especially those exploring the molecular aspects of the tumor.

Given the complex genetic background and molecular heterogeneity of WT, researchers are investigating diagnostic strategies that leverage molecular biomarkers. Effective analysis of gene mutations and cellular expression patterns has improved the prediction of disease progression and treatment responses. Prognostic models that incorporate gene characteristics have significantly increased the accuracy of prognosis predictions [16, 17]. Recent studies indicate that immune and stromal gene signatures can further refine tumor microenvironment characterization and prognostic stratification [18]. However, many existing models do not adequately integrate comprehensive clinical information with molecular data, which diminishes their predictive efficacy. Additionally, cellular components such as lymphocytes, fibroblasts, and endothelial cells within the tumor microenvironment play pivotal roles in regulating tumor growth, spread, and treatment responses [19, 20]. Current studies often neglect the intricate interactions among these cell types and their significance in tumor progression, thereby limiting the comprehensiveness and accuracy of prognostic models.

**Figure 1:**
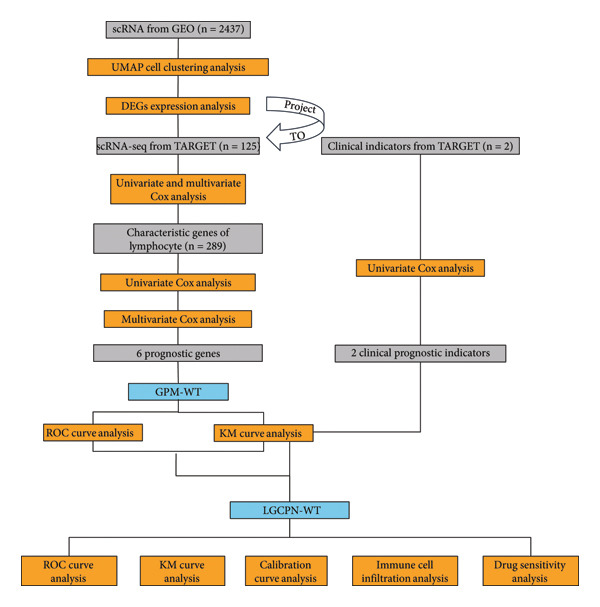
Research process for developing the multilayered prognostic model for Wilms′ tumor (WT).

Consequently, our research adopts an innovative approach by directly identifying key cell types that regulate tumor behavior and treatment responses via transcriptomic analysis of the tumor microenvironment. This strategy enables precise identification of cell types that are critical in shaping tumor dynamics and responses to treatment. We then screen for characteristic genes of these pivotal cells that influence survival and refine the model by integrating clinical indicators. The research process is depicted in Figure 1. Through this comprehensive methodology, we aim to deliver more personalized and accurate prognostic assessments for patients with WT.

## 2. Methods

### 2.1. Data Collection and Preparation

This study utilized RNA sequencing (RNA‐seq) and clinical data from 125 patients with WT, sourced from the Therapeutically Applicable Research to Generate Effective Treatments (TARGET) database. Additionally, single‐cell RNA sequencing (scRNA‐seq) data, comprising 2437 samples, were acquired from the Gene Expression Omnibus (GEO) database under dataset number GSM5344367. The scRNA‐seq data underwent processing with the “Seurat” R package, which excluded cells exhibiting gene expression levels below 300 or above 6500, as well as cells with mitochondrial gene expression exceeding 10%. Data normalization and scaling were performed using the SCTransform function, followed by data reduction through principal component analysis (PCA).

### 2.2. Cell Cluster and Feature Genes Analysis

Cell clusters were identified using Uniform Manifold Approximation and Projection (UMAP) technology and manually annotated based on established cell‐type marker genes. The expression of these marker genes across different cell types was verified using bubble plots. Differential gene analysis on the identified cell clusters selected genes that exhibited a fold change greater than three and a *p* value less than 0.05. These genes were then projected onto the RNA‐seq dataset, and patients were classified through hierarchical clustering of tumor‐specific genes. Both univariate and multivariate Cox regression analyses were conducted using the “survival” R package to identify genes associated with survival, which were subsequently visualized at the single‐cell level using UMAP.

### 2.3. Development of the Genetic Feature Prognostic Model for WT (GPM‐WT)

The GPM‐WT was established based on selected specific genes. The “ggplot2” R package was employed to create scatter plots and heatmaps displaying gene expression patterns across different risk levels, categorized by the median value. Kaplan–Meier survival curves assessed the prognostic capabilities of the model by comparing survival differences between high‐ and low‐risk patient groups. The impact of the model’s risk score was demonstrated using box plots generated by the “ggpubr” R package. Additionally, the “survival,” “survminer,” and “timeROC” R packages were used to plot ROC curves, assessing the predictive performance of the model.

### 2.4. Integration of Clinical Factors Into GPM‐WT

Univariate Cox regression analysis identified clinical factors associated with WT patient survival, which were integrated into the prognostic model to enhance its predictive accuracy and clinical applicability. The “rms” R package was utilized to produce nomograms visualizing the model, and ROC curves evaluated the predictive efficacy of the optimized model. Calibration curves compared the optimized model’s predictions with observed survival rates, thus reflecting model accuracy.

### 2.5. Evaluation of Immune Cell Dynamics and Drug Response

The “CIBERSORT” R package estimated the infiltration of 22 types of immune cells in two WT patient groups based on risk scores. Box plots depicted the differences in immune cell infiltration between high‐ and low‐risk patients. The responsiveness of these risk groups to therapeutic drugs was assessed using the “pRRophetic” R package, with drug sensitivity data (IC50) sourced from the Genomics of Drug Sensitivity in Cancer (GDSC) website (https://www.cancerrxgene.org/).

### 2.6. Statistical Analysis

All statistical analyses, model fittings, and graphic representations were performed using R statistical software. Spearman’s rank correlation was employed for all correlation analyses, while Kaplan–Meier plots, univariate, and multivariate Cox regression analyses utilized the coxph function. All statistical tests were two‐sided, with a significance level set at *p* < 0.05 to control for Type I errors.

## 3. Results

### 3.1. Cellular Composition in the Tumor Microenvironment

Clustering algorithms delineated nine distinct cell populations, each manually annotated with specific marker genes (Figures 2(a), 2(b)). scRNA‐seq identified five major cell populations—lymphocytes, tumor/epithelial cells, myeloid cells, endothelial cells, and fibroblasts—precisely annotated with unique marker genes (Figure 2(c)). Bubble plots demonstrated differential gene expression across these primary cell groups (Figure 2(d)). Correlation analysis indicated that dominant genes were coenriched in several cellular regions, suggesting that mRNA coregulation might be largely driven by cellular abundance heterogeneity, which implies coordinated communication across cell types (Figure 2(e)).

Figure 2Cellular composition and gene expression in the tumor microenvironment. (a) UMAP plot showing nine distinct cell populations, color‐coded by cell type. (b) Expression profiles of specific marker genes across different cell types. (c) UMAP visualization of five major cell populations, each highlighted with a unique color. (d) Bubble plot demonstrating differential gene expression across primary cell groups. (e) Left: mixed matrix plot depicting gene expression correlations between cell types; middle: heatmap detailing gene expression levels across different cell types; and right: correlation matrix illustrating gene expression correlations among cell types.

**Figure:**
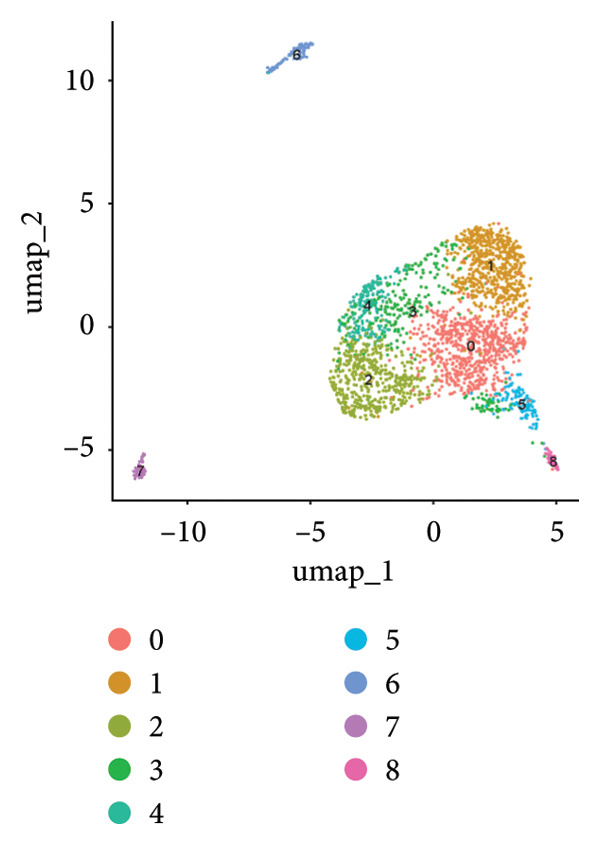
(a)

**Figure:**
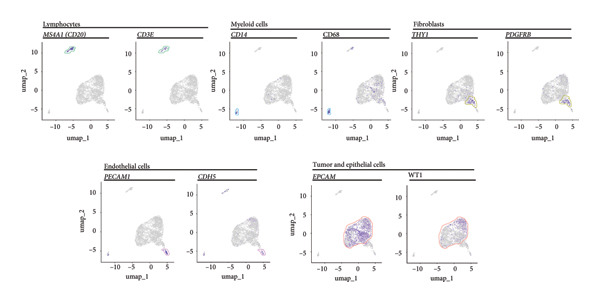
(b)

**Figure:**
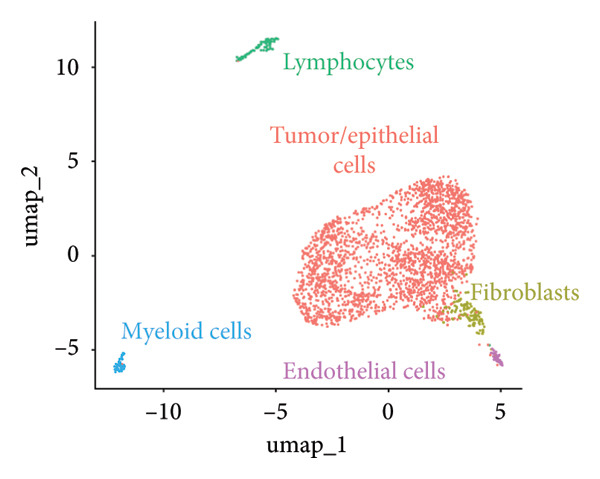
(c)

**Figure:**
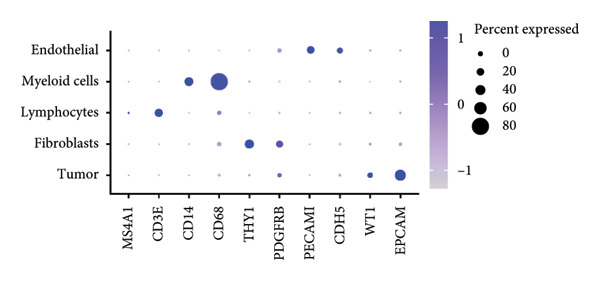
(d)

**Figure:**
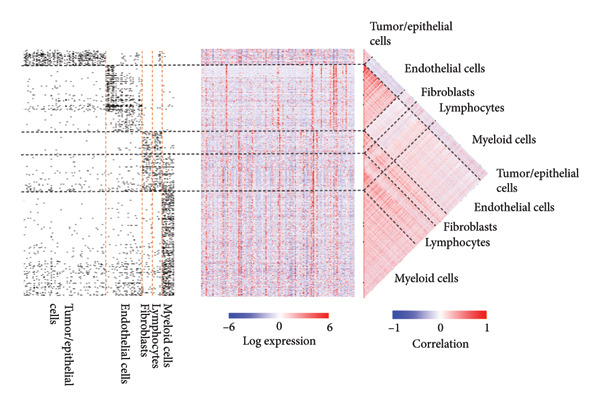
(e)

### 3.2. Impact of Lymphocyte‐Associated Genes on WT Prognosis

Univariate Cox regression analysis identified specific genes linked to lymphocytes, tumor cells, fibroblasts, and myeloid cells that significantly influenced survival (*p* < 0.05, Figure 3(a)). Multivariate analysis confirmed the prognostic significance of lymphocytes, which showed the highest level of statistical significance (the smallest *p* = 0.001, Figure 3(b)). Therefore, we focused the downstream analyses on lymphocyte‐associated gene signatures. Kaplan–Meier survival curves indicated that *Src-like adaptor (SLA)*, *mixed lineage leukemia translocated to 3 (MLLT3)*, and *sialic acid-binding Ig-like lectin 5 (SIGLEC5)* are associated with higher survival rates, classifying them as “better” prognostic genes, while *killer cell lectin-like receptor C1 (KLRC1)*, *apolipoprotein C2 (APOC2)*, and *guanylate binding protein (GBP2)*, linked to lower survival rates, are identified as “worse” prognostic genes (*p* < 0.001, Figure 3(c)). scRNA‐seq visualized the expression distribution of the six key genes (*KLRC1*, *APOC2*, *GBP2*, *SLA*, *MLLT3*, and *SIGLEC5*) within lymphocytes (Figures 3(d), 3(e), 3(f), 3(g), 3(h), 3(i)).

Figure 3Analysis of gene expression and its prognostic impact in WT patients. (a) Forest plot from univariate Cox regression showing hazard ratios for different cell types. (b) Forest plot from multivariate Cox regression displaying hazard ratios for selected cell types. (c) Kaplan–Meier survival curves for 135 WT patients, categorized by lymphocyte signature genes. (d)–(i) UMAP plots showing expression distributions of *KLRC1*, *APOC2*, *GBP2*, *SLA*, *MLLT3*, and *SIGLEC5* in scRNA‐seq data.

**Figure:**
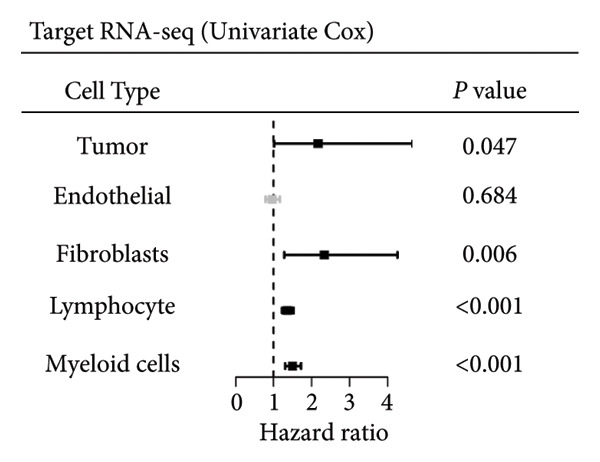
(a)

**Figure:**
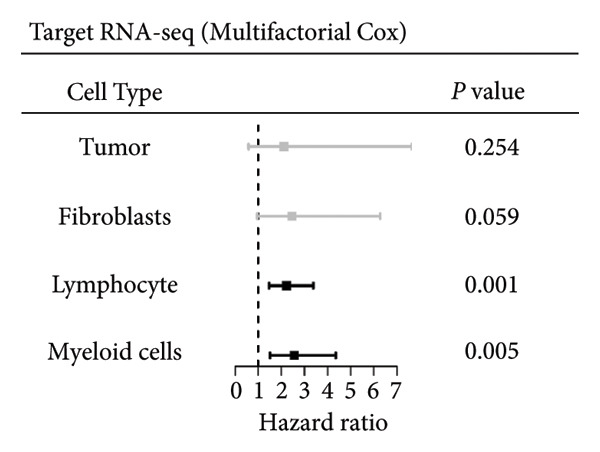
(b)

**Figure:**
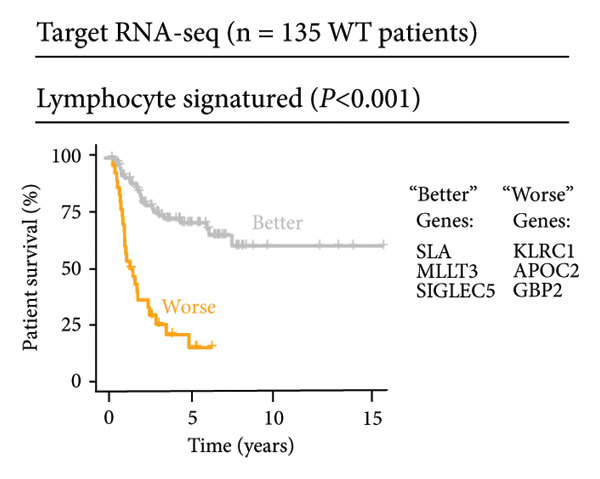
(c)

**Figure:**
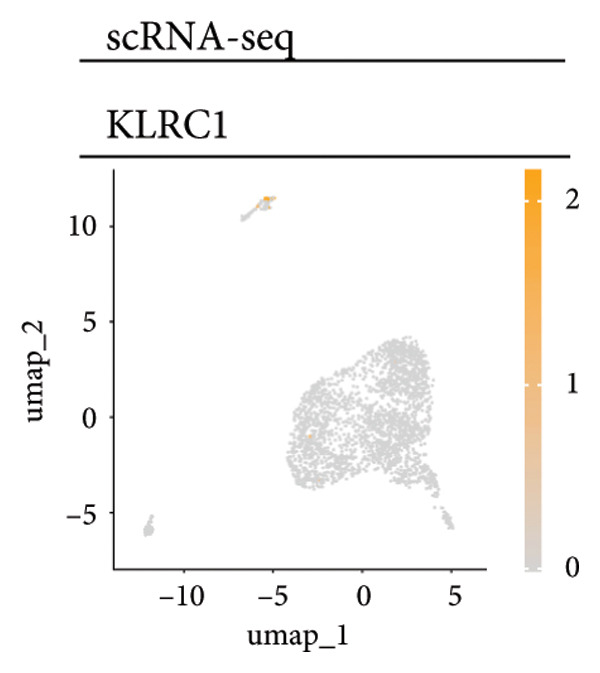
(d)

**Figure:**
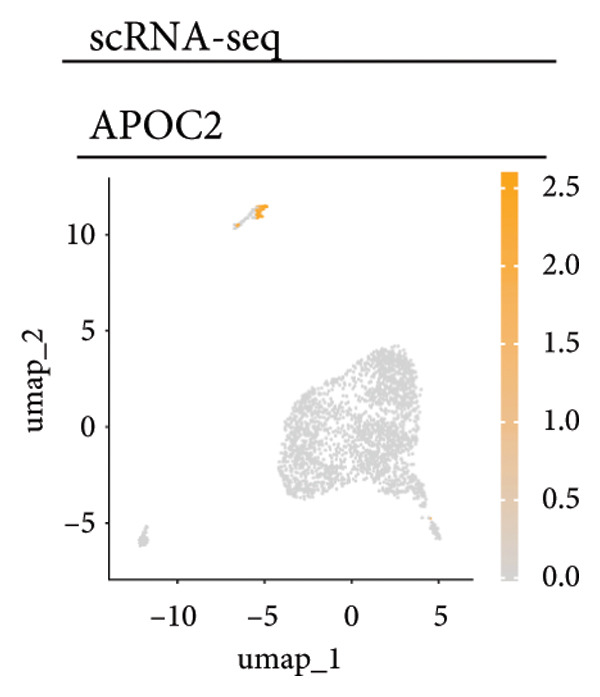
(e)

**Figure:**
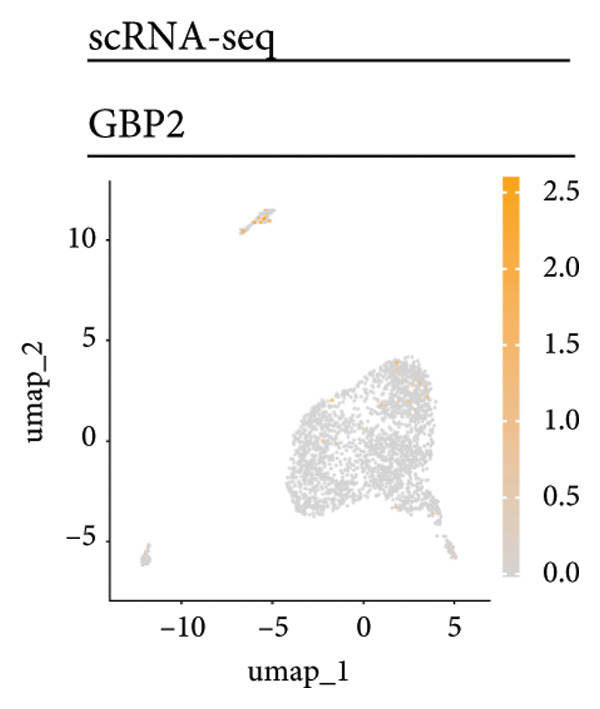
(f)

**Figure:**
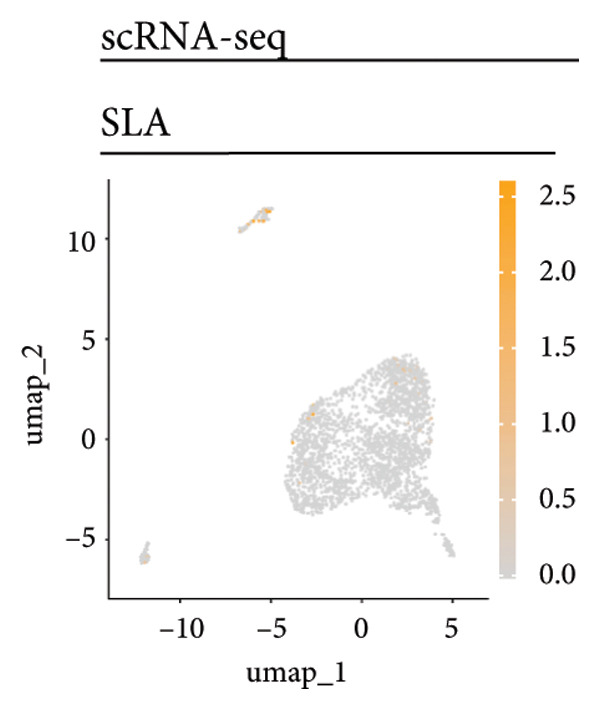
(g)

**Figure:**
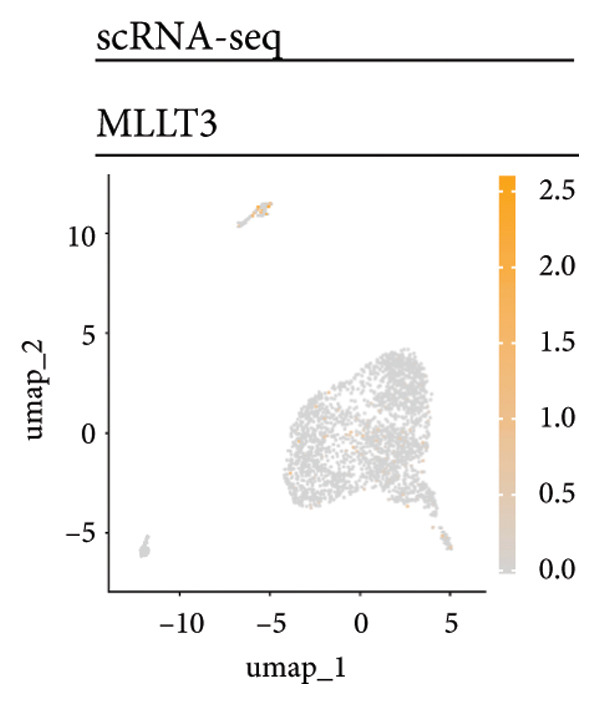
(h)

**Figure:**
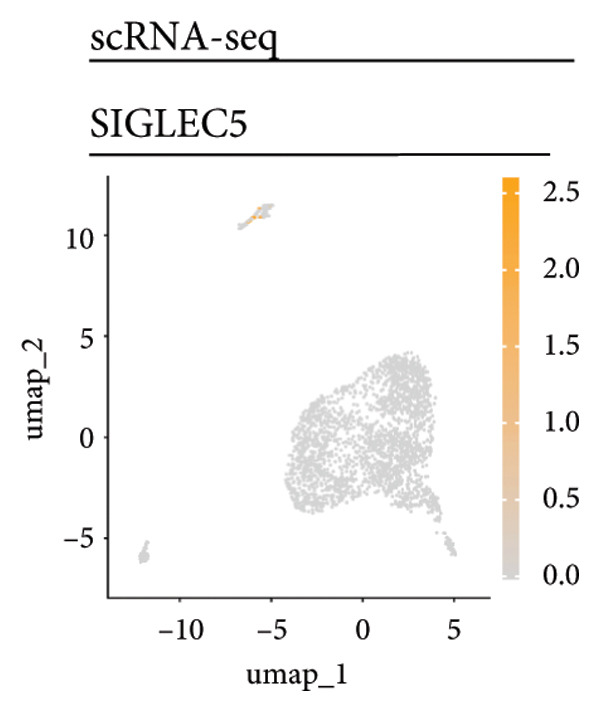
(i)

### 3.3. Construction and Validation of GPM‐WT

Based on the prognostically significant genes identified from the lymphocyte cluster, we constructed the GPM‐WT model. It was subsequently validated through comprehensive analyses of risk scores, survival outcomes, and predictive performance. Scatter plots and heatmaps revealed that patients with higher risk scores faced more adverse outcomes (Figure 4(a)). Kaplan–Meier survival curves validated the model’s predictive accuracy, showing significantly lower survival rates in the high‐risk group compared to the low‐risk group (*p* < 0.001, Figure 4(b)). Risk scores were consistently higher among deceased patients than survivors (*p* < 0.001, Figure 4(c)). ROC curves confirmed the model’s effectiveness in predicting 1‐, 2‐, and 3‐year survival rates, with area under the curve (AUC) values of 0.660, 0.745, and 0.710, respectively (Figure 4(d)).

Figure 4Performance evaluation of GPM‐WT. (a) Scatter plot and heatmap displaying risk scores, survival times, and gene expression levels. (b) Kaplan–Meier survival curves of GPM‐WT. (c) Box plot of GPM‐WT. (d) ROC curves of GPM‐WT.

**Figure:**
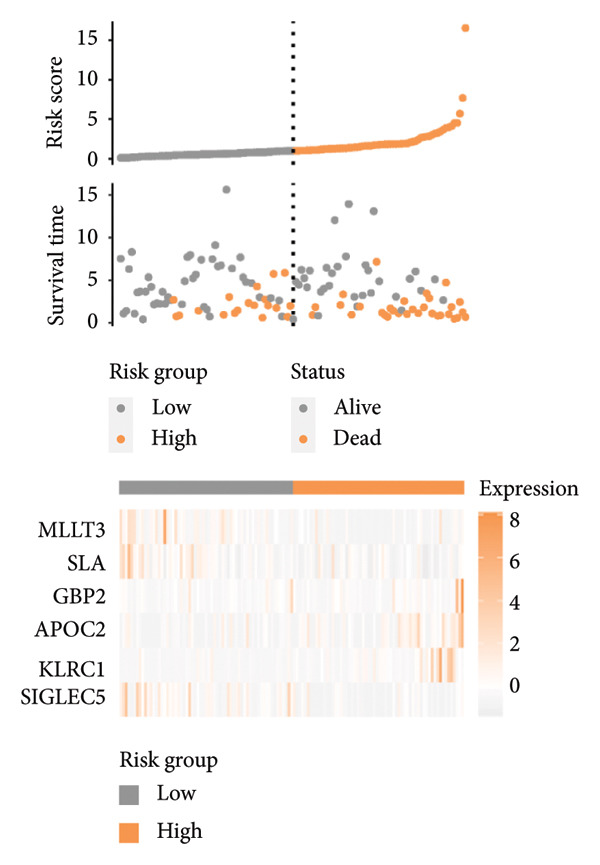
(a)

**Figure:**
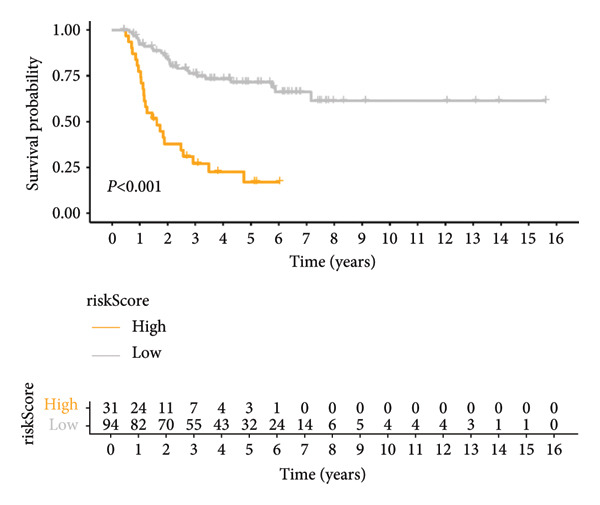
(b)

**Figure:**
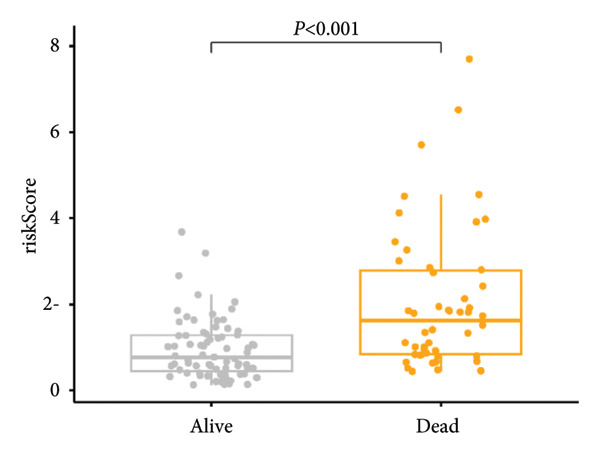
(c)

**Figure:**
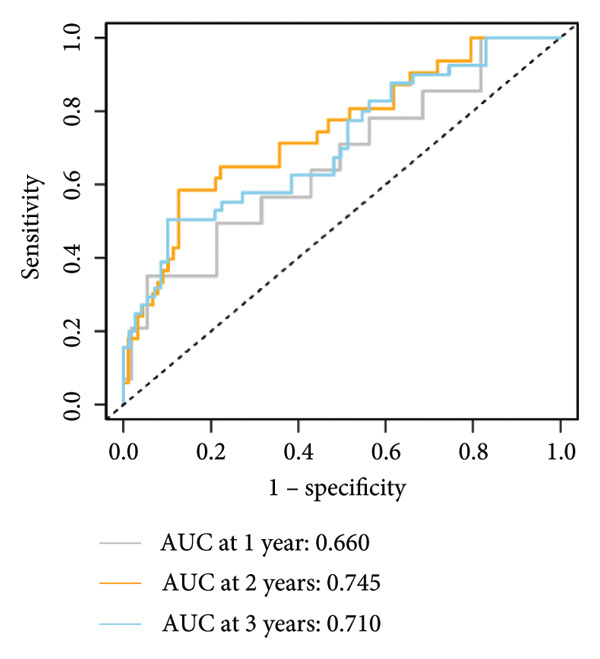
(d)

### 3.4. Development and Assessment of the Lymphocyte Gene and Clinical Feature Prognostic Nomogram (LGCPN‐WT)

Univariate Cox regression analysis revealed significant impacts of age and gender on WT patient survival (both *p* < 0.05, Figure 5(a)). The nomogram incorporating the expression scores of six genes—*KLRC1*, *APOC2*, *GBP2*, *SLA*, *MLLT3*, and *SIGLEC5*—along with age and gender was developed, forming the LGCPN‐WT (Figure 5(b)). ROC curves displayed the LGCPN‐WT’s proficiency in predicting 1‐ to 5‐year survival rates, with AUC values of 0.646, 0.771, 0.774, 0.751, and 0.785, respectively (Figure 5(c)). Calibration curves confirmed the consistency between the LGCPN‐WT’s predictions and actual survival rates (Figure 5(d)). Kaplan–Meier survival curves highlighted significantly lower survival rates in the high‐risk group compared to the low‐risk group (*p* < 0.001, Figure 5(e)).

Figure 5Analysis of prognostic clinical factors and performance evaluation of LGCPN‐WT. (a) Univariate Cox regression analysis of clinical factors. (b) Overview of LGCPN‐WT. (c) ROC curves of LGCPN‐WT. (d) Calibration plots of LGCPN‐WT. (e) Kaplan–Meier survival curves of LGCPN‐WT.

**Figure:**
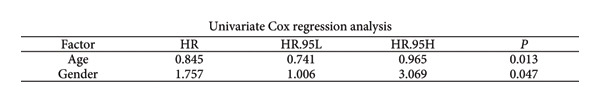
(a)

**Figure:**
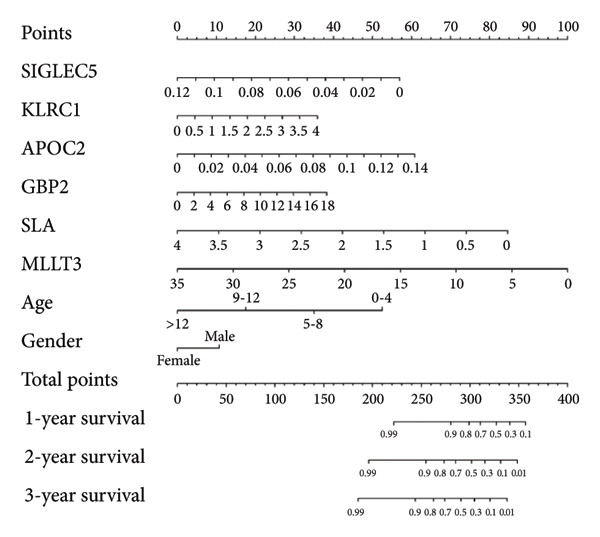
(b)

**Figure:**
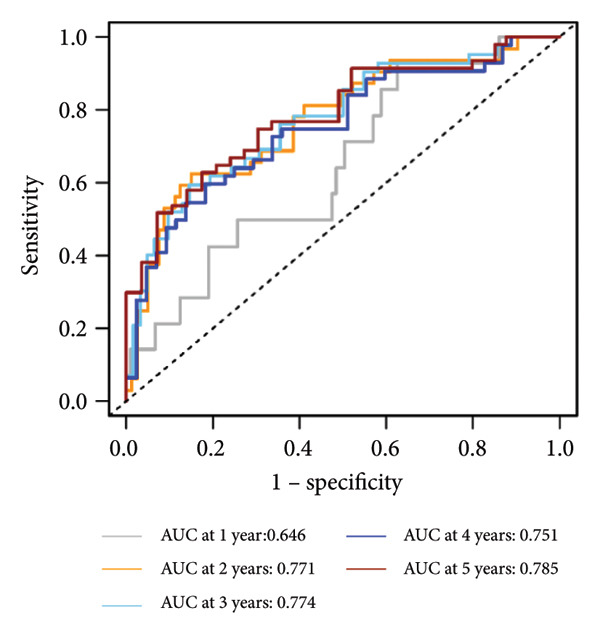
(c)

**Figure:**
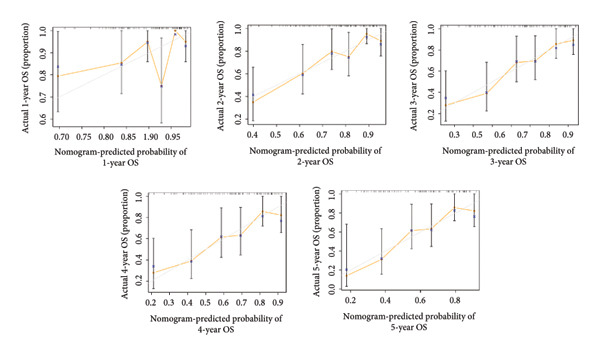
(d)

**Figure:**
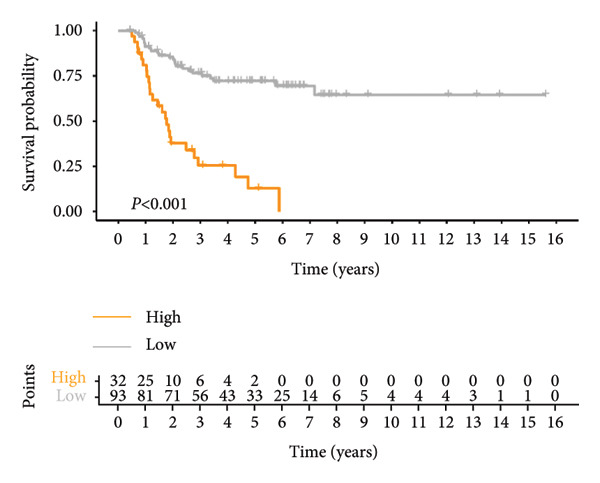
(e)

### 3.5. Comparative Analysis of Immune Cell Profiles and Drug Sensitivity in Different Risk Groups

Based on the LGCPN‐WT model, patients were stratified into high‐ and low‐risk groups. Compared to the low‐risk group, the high‐risk group exhibited higher levels of naïve B cells and activated mast cells (*p* < 0.01), as well as activated CD4 memory T cells, follicular helper T cells, regulatory T cells, activated NK cells, and M2 macrophages (*p* < 0.001, Figure 6(a)). This mixed enrichment pattern suggests that the high‐risk group presents a more complex immune landscape with both pro‐ and antitumor immune components. The high‐risk group had significantly heightened sensitivity to chemotherapy drugs, including docetaxel, paclitaxel, camptothecin, epirubicin, teniposide, cytarabine, and vinblastine (*p* all < 0.05, Figure 6(b)). Additionally, this group demonstrated markedly increased sensitivity to targeted drugs, such as crizotinib, alisertib, AZD5582, luminespib, niraparib, talazoparib, buparlisib, and LCL161 (all *p* < 0.05, Figure 6(c)), as well as to immunotherapy drugs, including obatoclax, mesylate, and leflunomide (all *p* < 0.05, Figure 6(d)). The higher predicted sensitivity to taxanes and anthracyclines likely reflects transcriptomic vulnerabilities characteristic of this molecular subtype rather than actual clinical responsiveness. Complete drug‐sensitivity results and corresponding *p* values are provided in Supplementary Tables 1 and 2.

Figure 6Comparative analysis of immune cell profiles and drug sensitivity in sigh *vs*. sow risk WT patients. (a) Box plots of differential expression of various immune cell types. (b) Box plots comparing expression levels of chemotherapy drugs (docetaxel, paclitaxel, camptothecin, epirubicin, teniposide, cytarabine, and vinblastine). (c) Box plots for targeted therapy drugs (crizotinib, alisertib, AZD5582, luminespib, niraparib, talazoparib, buparlisib, and LCL161). (d) Box plots for immunotherapy drugs (obatoclax, mesylate, and leflunomide).

**Figure:**
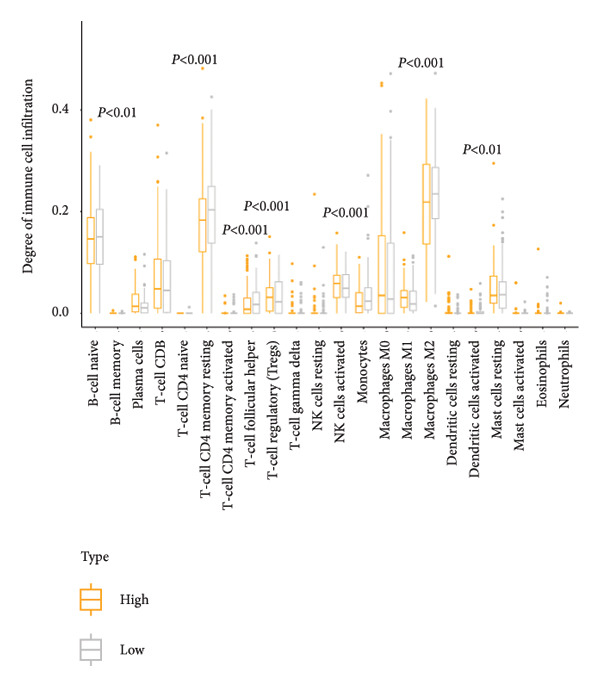
(a)

**Figure:**
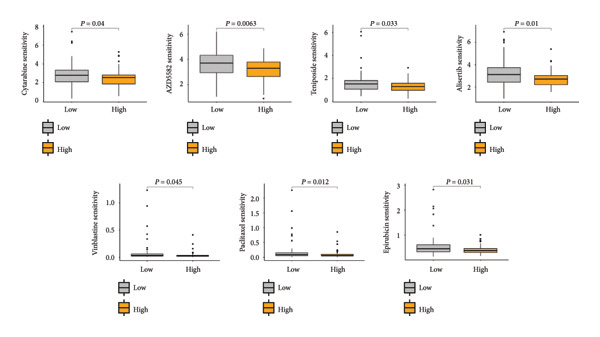
(b)

**Figure:**
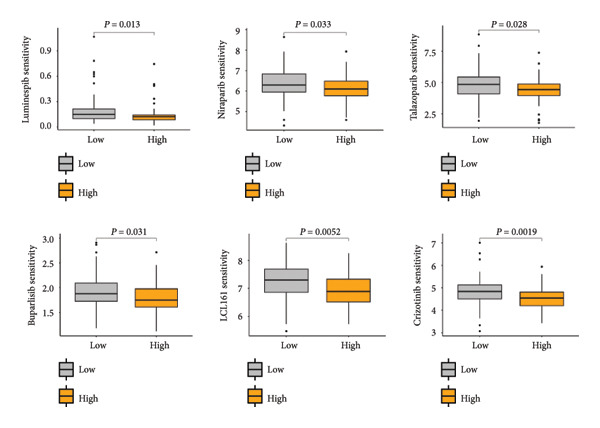
(c)

**Figure:**
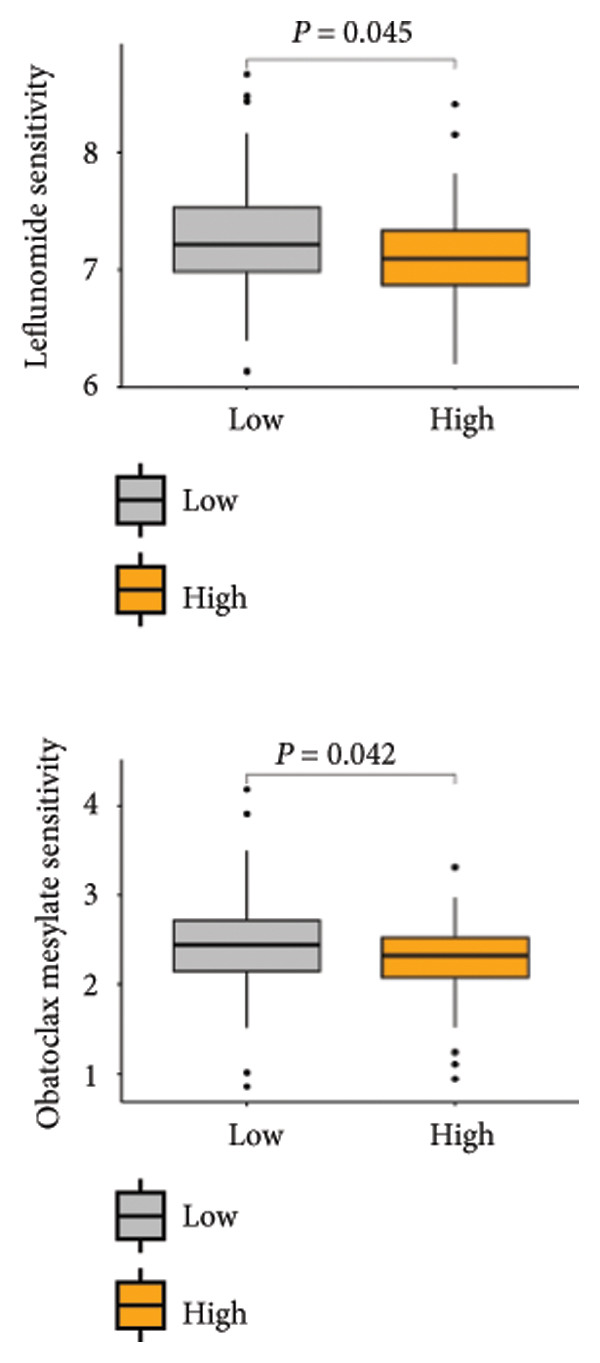
(d)

## 4. Discussion

This study developed and validated a multilayered prognostic model for WT that integrates lymphocyte signature genes with key clinical factors to substantially improve survival prediction. Using single‐cell sequencing, the model identifies specific cell types closely linked to WT prognosis, providing new insights and tools for clinical decision‐making. Similar single‐cell–based approaches have proven effective in other cancers, such as head and neck squamous cell carcinoma [21]. By combining molecular features with clinical indicators, our model offers a more comprehensive and biologically relevant framework for prognosis assessment.

Our findings emphasize the strong association between lymphocytes and WT prognosis and redefine their role in the tumor microenvironment. Previous studies [22, 23] reported that high densities of infiltrating T cells, particularly CD8^+^ T cells, correlate with improved survival. We extend these findings by demonstrating that lymphocyte gene expression patterns, rather than cell density alone, more accurately influence patient outcomes. WT cells can evade immune surveillance by secreting immunosuppressive cytokines (TGF‐*β* and IL‐10) or upregulating checkpoint molecules such as PD‐L1 [24, 25]. Our results suggest that specific lymphocyte‐related genes actively shape this microenvironment, providing potential targets for immunomodulatory therapy.

Six lymphocyte‐associated genes (*KLRC1*, *APOC2*, *GBP2*, *SLA*, *MLLT3*, and *SIGLEC5)* showed significant prognostic relevance. Among them, *MLLT3*, a YEATS family gene, may regulate transcriptional activation through phase separation, contributing to WT progression [26]. *KLRC1* and *SIGLEC5* mediate immune activation and suppression, respectively, and are involved in tumor immune surveillance [27, 28]. *APOC2* may influence tumor biology via lipid metabolism and inflammation [29, 30]. *GBP2*, a GTPase family member, and SLA are implicated in immune regulation and tumor growth [31, 32]. *SLA* plays a role in regulating immune escape and tumor growth [33, 34].

Gender and age were identified as significant clinical factors influencing WT prognosis. Epidemiological studies have established links between these factors and WT incidence; however, their precise effects on prognosis have not been thoroughly investigated [35]. Our findings are consistent with previous research showing that younger age is associated with poorer prognosis [36], likely due to the immature immune systems and high genetic heterogeneity of tumors in younger patients, which contribute to increased tumor aggressiveness. Although gender was not identified as a significant risk factor, male patients were found to face higher risks [16].

Compared to the GPM‐WT, based solely on six genes, the LGCPN‐WT—which integrates clinical factors and gene data—enhanced the prediction accuracy of 2‐year survival rates, increasing the AUC from 0.745 to 0.774, and of 3‐year survival rates from 0.710 to 0.751. This result indicates that a comprehensive model, by encompassing a wide array of biological and clinical factors, can significantly improve the predictive accuracy for WT patient prognosis. Similarly, comprehensive models have demonstrated effectiveness and reliability in other cancer studies [37–39], affirming the applicability and efficacy of our method.

Despite its good performance in predicting long‐term survival rates, the LGCPN‐WT model was less accurate in predicting 1‐year survival rate (AUC = 0.646). This reduced accuracy likely arises from the complex interplay of tumor biology and initial treatment responses. In the 1‐year posttreatment, rapid adaptations in the tumor microenvironment and initial treatment responses may not be fully reflected at the gene expression level, thereby limiting the short‐term accuracy of gene expression–based prognostic models [40, 41]. This observation aligns with existing literature studies [42–44] that notes early tumor responses often entail rapid biomarker changes within months of initiating treatment, potentially delaying gene‐level manifestations.

Our study evaluates both the models’ effectiveness in predicting survival and provides a comprehensive assessment of patient immune infiltration and drug sensitivity. Immune infiltration analysis showed that certain immune cells were significantly active in the high‐risk group (*p* < 0.001), indicating that their immune microenvironments could either suppress or promote tumor survival. Notably, the concurrent increase in both antitumor (CD4 memory T cells and NK cells) and immunosuppressive (regulatory T cells and M2 macrophages) cell types suggests an “activated but dysfunctional” immune microenvironment, in which compensatory immune activation coexists with immunoregulatory suppression, ultimately favoring immune escape and tumor progression. This immune imbalance may help explain the poorer outcomes observed in the high‐risk group. This immune cell spectrum analysis is crucial for selecting personalized immune therapies. For patients exhibiting high levels of immunosuppressive cells, immune checkpoint inhibitors could better activate an effective immune response.

Furthermore, the predicted drug‐sensitivity profiles provide important insights into potential therapeutic strategies for WT. The observed higher predicted sensitivity to taxanes and anthracyclines in the high‐risk molecular subgroup suggests possible transcriptomic vulnerabilities that could be exploited to optimize chemotherapeutic regimens. Taxanes, which stabilize microtubules and inhibit mitosis, and anthracyclines, which intercalate DNA and induce apoptosis, are among the most effective cytotoxic agents used in pediatric solid tumors [45, 46]. The predicted susceptibility of this subgroup may reflect alterations in cell‐cycle regulation, DNA damage response, or apoptotic signaling pathways that render tumor cells more responsive to these agents. In addition, the model identified potential responsiveness to emerging agents such as obatoclax and leflunomide. Obatoclax functions as a pan–BCL‐2 inhibitor that promotes apoptosis by neutralizing antiapoptotic proteins, while leflunomide suppresses tumor proliferation through inhibition of dihydroorotate dehydrogenase, thereby impairing pyrimidine synthesis and cell‐cycle progression [47, 48]. Although these drugs are not currently approved for WT treatment, their predicted activity in the high‐risk molecular subgroup highlights promising avenues for drug repurposing and rational combination therapy.

This study has several limitations. Firstly, the relatively small sample size may restrict the generalizability of the results, and subsequent studies should incorporate a larger patient dataset to confirm and broaden our findings. Secondly, while this study integrated gene expression with clinical data, it did not fully account for other potential biomarkers and environmental factors that could significantly affect WT prognosis. Thirdly, the clinical information available in the TARGET database was limited, which constrained the scope of our multivariate analysis. Only age, gender, survival status, and survival time were accessible, whereas important parameters such as laterality, body weight, and disease duration were not recorded. This restriction, inherent to the database’s genomic and transcriptomic focus, may have reduced the comprehensiveness of our clinical risk evaluation. Moreover, the GDSC database used for drug‐sensitivity prediction does not include data on immune checkpoint inhibitors such as PD‐1 blockers, which limited our ability to assess potential immunotherapy efficacy for WT. Furthermore, our model, primarily based on RNA‐seq data, faces inherent challenges due to the dynamic nature of gene expression, which limits its accuracy in predicting short‐term survival rates. Moreover, the lack of external validation with independent datasets may limit the confidence in extending our results to broader populations until further verification is performed. Lastly, while our research offers new insights for personalized treatment, translating these findings into practical clinical benefits will require additional prospective studies and clinical trials. Future research should further investigate these areas to improve the precision and clinical utility of the WT prognostic model.

In conclusion, this study developed the innovative LGCPN‐WT, a multilayered prognostic model that combines single‐cell sequencing data with traditional clinical indicators, significantly enhancing survival prediction accuracy for WT patients. Our results reveal the crucial role of lymphocytes and their specific genes in WT prognosis and introduce new approaches and tools for precision medicine and personalized treatment.

**Nomenclature**
APOC2: Apolipoprotein C2
AUC: Area under the curve
CTNNB1: Catenin beta 1
GEO: Gene Expression Omnibus
GPM‐WT: Genetic Feature Prognostic Model for Wilms’ Tumor
GBP2: Guanylate‐binding protein 2
GDSC: Genomics of Drug Sensitivity in Cancer
IC50: Inhibitory concentration 50
IGF2: Insulin‐like growth factor 2
KLRC1: Killer cell lectin like receptor C1
LGCPN‐WT: Lymphocyte Gene and Clinical Feature Prognostic Nomogram for Wilms′ Tumor
MLLT3: Mixed lineage leukemia translocated to 3
NK: Natural killer
PCA: Principal component analysis
PD‐L1: Programmed death‐ligand 1
ROC: Receiver operating characteristic
RNA‐seq: RNA sequencing
scRNA‐seq: Single‐cell RNA sequencing
SIGLEC5: Sialic acid binding Ig‐like lectin 5
SLA: Src‐like adaptor
SCTransform: Scaled data transformation (used in the context of data normalization for sequencing data)
TARGET: Therapeutically applicable research to generate effective treatments
TGF‐β: Transforming growth factor‐beta
Tregs: Regulatory T cells
UMAP: Uniform Manifold Approximation and Projection
WT: Wilms’ tumor

## Conflicts of Interest

The authors declare no conflicts of interest.

## Funding

The authors declare that no financial support was received for the research, authorship, and/or publication of this article.

## Supporting Information

Supplementary Table 1 presents the complete drug‐sensitivity profiling results. Supplementary Table 2 provides the *p* values corresponding to the drug sensitivity.

## Supporting information


**Supporting Information** Additional supporting information can be found online in the Supporting Information section.

## Data Availability

All data utilized in this study are publicly available. Data were sourced from the TARGET database (https://ocg.cancer.gov/programs/target/data-matrix) and the GEO database (https://www.ncbi.nlm.nih.gov/geo/). The methodologies employed for data processing and analysis—including normalization, scaling, cell clustering, and differential gene analysis—are comprehensively detailed in the Methods section. This ensures reproducibility and facilitates validation by other researchers.
