# Supplementary material for: Development of a Multilayered Prognostic Model for Wilms’ Tumor Based on Characteristic Lymphocyte Genes
Source: Genet Res (Camb). 2025 Dec 18;2025:1964582. doi: 10.1155/genr/1964582 (PMC12721763; doi:10.1155/genr/1964582)
Supplement: Supplementary file 1 — Supporting Information Additional supporting information can be found online in the Supporting Information section. [file GENR-2025-1964582-s001.zip › Supplementary Table 1.pdf]

Supplementary table 1 Drug-sensitivity results for chemotherapeutic and targeted agents.

	Camptothecin_1003	Vinblastine_1004	Cisplatin_1005	Cytarabine_1006	Docetaxel_1007	Gefitinib_1010	Navitoclax_1011	Vorinostat_1012	Nilotinib_1013	Olaparib_1017	Axitinib_1021	AZD7762_1022	SB216763_1025	KU-55933_1030	Afatinib_1032	Staurosporine_1034	PLX-4720_1036	NU7441_1038	Doramapimod_1042	Wee1 Inhibitor_1046
TARGET-50-PAJNAV	0.6851	0.1843	689.5825	28.8506	0.0238	26.0201	7.1876	2.9319	21.8418	297.7714	16.3364	0.8525	80.8597	81.7495	4.3979	0.1240	188.1325	15.5642	80.0552	16.4944
TARGET-50-PAJPEW	0.1208	0.0419	28.2085	8.0161	0.0080	26.3474	23.9012	4.3507	45.8892	109.4207	22.1973	1.7068	141.1019	79.4188	7.8040	0.0391	83.5699	14.9277	96.6523	9.9823
TARGET-50-PAJMLZ	0.1055	0.0448	86.4915	14.9022	0.0193	26.2565	4.1516	6.3609	64.9224	111.6641	18.0599	2.3579	284.2162	93.3291	5.6615	0.0997	86.7639	15.5600	86.6769	11.5911
TARGET-50-PAKRVH	0.1210	0.0344	24.9062	8.7770	0.0160	20.1130	23.2188	7.1948	54.7392	100.8719	23.5087	1.9102	174.0762	83.6212	5.0095	0.0453	78.3780	14.4828	71.2572	13.4260
TARGET-50-PADZUB	0.0510	0.0126	13.5705	3.0179	0.0080	30.0267	6.1150	3.9175	60.6994	68.1360	19.6051	0.8636	284.7452	101.0352	8.3728	0.0467	76.6231	17.2200	100.4488	6.5331
TARGET-50-PADXAY	0.1950	0.0235	32.8009	8.6097	0.0132	60.8591	3.9630	6.9391	22.9394	260.8839	31.2796	1.0133	179.6854	101.7170	17.0194	0.1628	180.2116	15.5261	78.5318	7.2302
TARGET-50-PAJPCM	0.0631	0.0108	11.8825	4.7123	0.0067	11.2463	4.4706	4.0943	24.2632	98.8178	27.0465	0.8992	227.4626	99.8502	4.6748	0.1377	71.5582	17.1519	144.9801	2.6254
TARGET-50-PAJPAR	0.1630	0.1283	43.3320	13.2684	0.0203	27.8168	1.4474	3.3687	47.8338	112.3631	25.8303	2.1519	329.0792	91.2345	9.1117	0.1087	106.9627	17.0347	112.4328	7.8553
TARGET-50-PAJNZS	0.1321	0.0445	29.0682	20.1295	0.0139	22.5204	5.9918	5.6456	58.7568	105.9229	25.1091	1.5029	288.0459	88.9839	5.5063	0.0955	117.7053	15.9618	134.5377	20.3807
TARGET-50-PAJMVC	0.1018	0.0387	9.8848	5.6464	0.0093	13.4814	40.8045	4.5746	17.0664	52.9040	21.6193	0.6772	90.0143	71.0325	2.6070	0.0156	118.0286	13.0838	110.2619	10.5701
TARGET-50-PALGAZ	0.1189	0.0238	31.6442	5.7102	0.0143	32.6761	9.6579	4.9344	36.7644	68.4170	24.1793	1.4494	180.5844	87.5753	8.2983	0.0560	80.8367	15.0117	86.9108	8.0950
TARGET-50-CAAAAR	0.0943	0.0172	30.3773	5.1260	0.0084	16.3625	5.5950	4.7578	39.3257	48.1821	22.5465	1.8025	142.0172	78.9765	3.2191	0.0267	45.9050	14.2595	85.3312	9.9107
TARGET-50-PAKGMU	0.1261	0.0373	63.8743	10.4440	0.0126	50.4164	4.9213	3.6171	41.0761	138.6963	26.4018	1.0707	244.8784	94.7734	10.6800	0.0632	171.7682	16.0544	112.3386	11.0113
TARGET-50-PAKRCC	0.1026	0.0257	37.0752	3.8639	0.0143	26.4511	1.3161	2.5020	38.9679	80.2410	35.0596	0.9226	193.6014	98.5516	6.3711	0.0858	75.7729	15.7399	62.2705	2.8155
TARGET-50-PAKFME	0.1011	0.0300	30.8922	7.1615	0.0132	29.5386	3.8234	4.8944	41.3835	79.0288	23.3793	0.8924	230.4344	84.2876	6.7375	0.0333	62.1191	14.7593	88.7193	6.1475
TARGET-50-PAJNNC	0.0649	0.0235	12.1794	4.8293	0.0106	19.7362	5.3640	3.5018	35.2169	58.4962	23.2960	0.8261	100.9553	89.2192	5.2612	0.0788	93.9043	15.0935	80.5447	8.1532
TARGET-50-PAJNJJ	0.1112	0.1040	81.5785	11.5752	0.0344	44.2260	3.2720	5.9437	87.1056	153.9649	28.8987	3.1163	313.2760	106.0496	6.2847	0.0891	126.6670	16.6304	108.4089	18.6404
TARGET-50-PAKJGM	0.0279	0.0055	4.1483	0.9764	0.0032	22.7788	19.3907	3.0517	11.1810	32.7597	16.4146	0.3316	135.6273	86.9775	3.6207	0.0124	67.0419	15.2264	134.6611	3.9969
TARGET-50-PAJNGH	0.0570	0.0343	15.9489	6.0406	0.0135	16.0106	9.3759	5.3200	46.8594	47.1431	23.6302	1.8527	177.0312	89.1605	4.5932	0.0735	72.8592	15.4537	64.1423	7.8594
TARGET-50-CAAAAC	0.0972	0.0357	25.8857	3.3269	0.0299	33.3692	2.1461	4.5666	41.8578	50.0742	21.2839	1.3375	217.4489	108.8811	8.9406	0.1598	155.9934	17.0306	61.1460	4.3780
TARGET-50-PAJNBN	0.0493	0.0103	17.8889	1.9905	0.0063	14.8086	1.9725	2.6360	24.7082	45.3790	25.9951	0.5519	273.5033	100.0525	3.4999	0.0343	58.2927	16.1343	110.9381	2.4383
TARGET-50-PAKYLT	0.4692	0.0270	90.4646	9.8638	0.0283	18.2531	3.9861	5.9992	29.4311	66.0370	13.0280	1.9843	95.5568	70.2000	5.5134	0.0964	108.6508	13.4730	70.0223	12.5758
TARGET-50-PAJMKN	0.1596	0.0555	80.8315	13.9503	0.0282	25.4788	11.8187	8.4167	46.3427	102.7201	25.7170	2.2597	182.3384	89.7011	4.9227	0.0363	70.1052	15.7336	107.5924	25.6062
TARGET-50-PALERC	0.0525	0.0101	16.7407	2.3644	0.0057	14.9601	6.8643	3.4555	27.6623	47.4215	19.1274	0.8729	214.2965	83.4506	3.3313	0.0293	39.0064	14.7574	103.0630	6.1102
TARGET-50-PAJMFU	0.0686	0.0164	22.6507	3.0302	0.0128	32.0040	9.2442	4.5464	53.9997	59.3388	29.4577	0.9634	254.1397	97.6666	7.4996	0.0297	90.7429	16.3705	133.5794	7.4771
TARGET-50-PAKZHF	0.0326	0.0199	9.5010	4.9843	0.0064	23.2638	36.2541	3.6284	47.2950	60.8450	23.1100	1.0210	173.3156	87.2349	5.7617	0.0260	63.4724	15.4946	94.2080	9.5199
TARGET-50-PAJLSP	0.0545	0.0246	14.9015	2.4545	0.0114	17.8546	5.9282	3.5170	48.4450	53.6756	24.0873	1.6906	121.6750	98.6627	5.3388	0.0885	165.6008	15.3496	54.1756	5.8198
TARGET-50-PAJMKI	0.0514	0.0252	14.2803	5.9949	0.0088	30.1043	2.6598	3.6638	34.4071	66.5678	23.6106	1.0866	306.4555	89.0886	8.0447	0.0412	61.2700	15.0149	85.0685	4.1324
TARGET-50-PAKUIT	0.0789	0.0211	16.5006	3.7977	0.0116	32.9724	1.3728	2.7314	30.2011	77.0339	20.1124	0.6319	277.6574	96.2360	13.0637	0.1399	80.6195	16.1588	78.9081	2.8846
TARGET-50-PALDWP	0.0400	0.0071	9.3580	1.4773	0.0052	19.7492	3.9551	2.6985	25.5361	36.0522	19.2068	0.4947	264.9572	92.4055	4.5420	0.0343	62.8350	15.7308	100.3886	2.8598
TARGET-50-PAJMJT	0.0600	0.0125	5.1994	3.1951	0.0079	24.2560	27.1848	4.4637	65.4677	24.9418	16.3593	1.2736	239.8629	96.6080	6.3580	0.0371	80.3695	16.4858	102.2114	8.0043
TARGET-50-PAKWPM	0.0483	0.0178	13.7611	4.2020	0.0093	21.6776	3.5114	4.1992	42.6178	68.9951	24.3334	0.9830	318.2934	99.1666	4.9830	0.0361	81.9974	16.3630	108.7138	10.6753
TARGET-50-PAJLKC	0.0445	0.0111	16.5064	3.6112	0.0081	18.4347	2.3008	2.0298	37.4158	54.7565	25.5378	0.9512	149.0177	98.6880	4.0465	0.0661	77.0066	16.0500	61.4916	2.9091
TARGET-50-PALKRS	0.0980	0.0121	14.2050	4.8393	0.0053	29.1599	63.8002	4.9927	23.8048	98.2411	19.5369	0.6164	93.4228	77.5711	5.3982	0.0196	156.0309	14.3981	112.1942	10.0235
TARGET-50-PAKNRX	0.1700	0.3318	57.9782	37.8703	0.0590	30.1404	5.1902	8.3213	117.4474	234.1039	38.0761	4.7224	295.0973	109.4298	8.0651	0.1342	361.3627	17.2044	124.0829	32.9701
TARGET-50-PAJNTJ	0.0619	0.0218	17.0339	7.2543	0.0108	19.8319	27.6622	3.7724	64.0863	52.4133	24.8232	4.9437	205.3784	93.1326	4.3158	0.0634	81.1954	15.8638	83.9678	15.8440
TARGET-50-PAJNUS	0.0606	0.0116	11.1942	3.7230	0.0065	23.4017	11.8972	4.1311	47.4399	30.6932	19.5751	1.0880	249.4896	94.2243	5.0191	0.0257	62.6077	16.2993	109.0957	6.2972
TARGET-50-PAKNTW	0.0188	0.0049	1.8535	0.2473	0.0028	21.1116	11.5983	2.3525	16.6137	25.8647	17.1967	0.1739	224.6740	87.5529	6.2337	0.0162	30.6624	15.1651	119.0671	1.8343
TARGET-50-PAJPAU	0.0559	0.0614	17.7577	4.8257	0.0255	23.4061	3.2873	5.8665	67.4981	39.8904	31.8040	1.2289	177.6486	91.0143	6.8320	0.0670	62.5741	16.1553	95.3433	7.7033
TARGET-50-PAJMVU	0.0604	0.0205	19.2951	6.6986	0.0110	66.4805	11.7887	4.8733	46.4009	78.1107	34.4521	1.2330	345.6882	107.1213	7.1832	0.0262	93.1327	17.2540	138.4167	6.9956
TARGET-50-PAJNZI	0.0779	0.0167	12.9643	4.3376	0.0105	18.9724	1.2506	2.5017	28.7427	63.2723	28.5807	1.2123	197.1181	96.7186	6.5089	0.1198	56.9619	16.4703	89.8150	2.5065
TARGET-50-PAJPDN	0.1009	0.0219	9.1975	6.8630	0.0088	30.8081	34.6521	5.1897	31.7757	70.5910	18.0130	0.8118	87.0571	67.2060	10.5747	0.0227	112.4992	13.3925	110.2251	9.7659
TARGET-50-PAJNVE	0.0622	0.0103	7.4817	2.4235	0.0063	13.1576	1.3243	3.2976	23.1724	22.8774	15.1513	0.7758	210.7367	77.8707	5.2969	0.0843	32.4433	15.2922	85.1649	2.2677
TARGET-50-PAJMSE	0.0941	0.0164	53.5631	7.0817	0.0148	21.4669	4.3637	5.5303	51.5975	74.0619	28.7487	1.1697	204.4222	86.2650	5.8392	0.0461	54.8499	15.2520	84.9350	6.7599
TARGET-50-PAJNZU	0.1036	0.0240	32.9057	7.8487	0.0105	17.8666	2.7347	4.6492	52.7204	46.3672	25.1025	1.3767	241.9302	82.5634	3.6169	0.0301	39.3472	14.5512	87.8138	8.3767
TARGET-50-PALGLU	0.0403	0.0112	10.0823	1.5346	0.0041	12.5819	3.8482	2.7248	19.4865	75.1491	17.1254	0.5214	145.4334	83.6690	3.3448	0.0350	71.9533	14.7622	104.9374	3.8090
TARGET-50-PAJNZK	0.1011	0.0275	23.5645	3.7124	0.0160	28.9835	2.4489	3.5976	40.7414	46.6209	26.8661	1.0855	258.8292	88.3819	6.4498	0.0514	75.9067	15.2335	78.5351	3.0552
TARGET-50-PAJMUF	0.0393	0.0065	14.3733	1.7861	0.0036	30.0584	3.8409	3.5208	23.1109	36.2675	18.9939	0.9545	181.1717	86.1190	4.1472	0.0204	32.6754	14.9162	102.0684	5.0722
TARGET-50-PAJMRL	0.0534	0.0125	21.0058	6.4391	0.0064	32.6390	4.7282	6.6732	61.4245	99.1135	31.3294	1.6504	250.1453	93.2207	11.3272	0.1097	72.9821	16.1831	83.9969	4.2427
TARGET-50-PAJMXF	0.0813	0.0193	24.1134	3.6611	0.0085	17.2098	6.2716	5.2069	42.6597	51.5708	19.9422	1.7952	192.1030	91.8911	3.5158	0.0427	86.6825	15.6473	89.6410	7.1110
TARGET-50-PAKPDF	0.0716	0.0188	29.9547	4.9115	0.0140	19.3459	2.8353	4.2761	39.3621	73.0175	29.6887	1.5588	247.1490	95.3113	4.7568	0.0725	59.3713	15.7132	85.3455	5.4686
TARGET-50-PAEBXA	0.1015	0.0369	33.8065	5.9196	0.0100	28.8968	12.7091	4.5962	43.3158	80.8935	29.4201	0.9132	207.5102	94.0652	6.2398	0.0433	70.2793	15.7286	102.7414	8.0781
TARGET-50-CAAAAS	0.1402	0.0476	26.9081	7.0153	0.0405	19.2135	5.0253	6.2177	71.4330	51.3017	20.3962	3.5391	119.5904	89.3104	6.3067	0.1270	121.2728	15.2098	51.2786	11.5565
TARGET-50-PAJNLT	0.0497	0.0184	11.9258	1.7115	0.0077	24.5216	4.6909	4.9327	38.4508	50.3291	20.3726	1.4768	181.8182	82.8970	7.5518	0.0636	51.7552	14.4713	85.2221	4.0509
TARGET-50-PAJPHA	0.0904	0.0315	19.4895	4.6320	0.0099	23.2214	8.6755	6.0366	42.6063	49.6636	18.4817	1.0434	193.2492	81.7117	6.3147	0.0553	85.2588	14.6718	83.6585	11.1063
TARGET-50-PAJMMY	0.1331	0.0157	22.8178	2.8879	0.0067	14.2782	2.8471	3.7509	28.7849	32.9191	22.4831	0.9305	128.3767	77.7508	3.3452	0.0374	58.4746	13.8086	66.1474	3.8224
TARGET-50-PAJLTH	0.0533	0.0187	13.0989	2.6300	0.0104	27.4858	7.6657	4.0825	38.2018	41.2145	22.8083	1.0561	184.0599	96.5315	5.2245	0.0501	91.0472	16.2457	74.2882	5.9397
TARGET-50-PAJNCC	0.0954	0.0145	32.1363	6.9743	0.0097	32.2448	2.0324	3.0835	26.6748	92.4418	7.7985	0.2379	382.0911	68.8907	10.3222	0.0780	143.5083	13.9112	77.1657	3.4051
TARGET-50-PAJLUJ	0.0357	0.0092	5.9531	1.8293	0.0035	18.1506	10.2373	2.9592	15.2867	27.9296	18.2192	0.4719	192.2661	79.9293	4.1236	0.0108	50.4060	14.8288	128.6629	7.4542
TARGET-50-PAJNCJ	0.0868	0.0240	55.1914	6.2186	0.0106	22.7770	4.7927	5.6887	43.1469	79.6195	21.7701	1.3922	168.3777	88.9441	5.1118	0.0493	67.0029	14.6948	72.0156	5.8755
TARGET-50-PAKZER	0.0381	0.0205	15.3028	2.5443	0.0064	31.0242	1.2226	2.7057	28.8778	78.3958	21.0378	0.4640	272.4597	93.9185	6.3226	0.0465	62.3101	15.6981	108.0349	4.7146
TARGET-50-PAJMEP	0.0829	0.0152	24.0744	3.2508	0.0070	17.8800	2.8465	3.6067	36.3870	39.9174	19.8838	1.4214	153.1684	84.1907	3.6810	0.0588	53.2832	14.4272	64.1718	4.9494
TARGET-50-PAJMEL	0.1757	0.0214	32.2416	5.6419	0.0109	17.6586	10.2032	4.9190	34.2207	48.2718	17.5179	2.3276	130.8571	70.5765	5.2114	0.0220	41.0741	13.2439	78.9164	15.5273
TARGET-50-PAJNVX	5.0458	0.2621	297.3257	74.5527	0.0878	49.0042	12.1061	8.0260	34.1804	250.8988	21.3605	3.0726	164.0451	67.9662	18.9099	0.1082	156.0676	13.4716	92.7169	97.8987
TARGET-50-PAKXWB	0.1816	0.0357	36.1310	8.0877	0.0102	26.7139	4.9936	4.1505	19.2470	77.4817	21.9781	0.7349	136.0345	76.8373	5.1362	0.0212	81.8180	14.6932	119.6528	13.9739
TARGET-50-CAAAAB	0.0688	0.0269	27.4750	5.1674	0.0122	16.1379	1.3546	2.4701	52.6736	48.8199	25.6809	0.8650	183.6275	101.9159	4.8799	0.0716	136.4893	16.1971	72.9719	5.6016
TARGET-50-PALJIP	0.0704	0.0170	24.8425	3.3355	0.0085	19.8025	4.6456	3.4630	24.3566	79.3413	18.0669	0.6545	132.1847	83.6240	4.9324	0.0483	62.3117	14.5615	63.8462	3.9180
TARGET-50-PAKMUB	0.1231	0.1166	51.4749	27.3861	0.0276	32.5597	17.7085	6.4685	54.3426	151.9457	21.5889	2.1579	155.9121	72.4902	10.8066	0.0269	102.5193	13.8998	85.3995	50.2112
TARGET-50-PALKCW	0.0680	0.0160	43.9817	4.7902	0.0073	28.4943	12.3970	4.5384	39.1263	85.3519	25.5538	0.8922	208.9393	93.8516	5.4509	0.0293	88.0501	15.8791	114.5744	9.0297
TARGET-50-CAAAAQ	0.0286	0.0079	4.8104	0.4865	0.0041	12.9705	3.9477	2.3957	19.5721	22.9179	19.0200	0.5802	180.7669	100.7980	2.6465	0.0364	65.1932	16.5649	97.5536	2.5800
TARGET-50-PALFME	0.0401	0.0144	13.8713	2.1152	0.0100	30.0332	10.5804	3.3341	39.4945	71.7453	27.4488	0.6808	303.1355	109.9323	7.5701	0.0934	69.8124	17.2135	95.3982	2.5710
TARGET-50-PAKNXS	0.1726	0.0618	40.1335	18.1840	0.0168	16.0705	3.8033	3.9994	60.0805	157.3516	22.0982	1.7384	227.5505	86.3921	6.5100	0.0846	160.6046	16.0201	117.1245	11.8448
TARGET-50-PAJNAA	1.0048	0.9230	391.9171	83.4983	0.2975	81.5546	30.9354	13.9221	188.4504	402.9422	31.9448	9.0418	214.2576	87.6260	18.0448	0.1508	178.0832	15.4130	79.4806	92.4255
TARGET-50-PAJPGY	0.0707	0.0263	33.8999	3.4456	0.0070	26.5252	1.7861	3.3815	39.5402	96.7689	18.8257	0.4967	208.9362	92.1687	5.4915	0.0489	100.2431	15.1470	103.4444	4.9981
TARGET-50-PAJNEC	0.0304	0.0027	2.3826	1.9211	0.0046	48.7461	113.5053	4.2757	29.0186	128.5764	33.4197	0.5666	116.3694	93.6558	7.1542	0.0188	270.9271	15.1814	170.0917	6.6747
TARGET-50-PAJNDU	0.0404	0.0028	6.9599	0.4864	0.0019	14.0700	126.8254	3.2468	9.7726	64.7805	15.3606	0.2621	99.6458	75.9735	2.4859	0.0104	69.3443	13.8366	144.5967	3.1567
TARGET-50-PAKGZX	0.0549	0.0231	16.7518	5.9939	0.0078	27.0507	12.8959	3.8414	23.3183	95.6643	35.5040	0.6941	164.2786	64.8723	6.3575	0.0094	39.4024	13.2324	94.2094	11.0606
TARGET-50-PAKFYV	0.2303	0.0164	102.6377	10.9708	0.0153	10.6192	13.5088	4.8629	21.8953	77.9286	22.2527	0.6987	179.9338	95.2460	5.0768	0.1079	95.1580	15.6832	95.8846	5.3417
TARGET-50-PAKGED	0.1751	0.0233	12.9894	6.3995	0.0041	24.9520	229.2872	2.9707	28.6000	170.1367	19.5122	0.3863	81.0907	70.2595	3.3397	0.0143	183.2303	13.5038	117.3465	3.1423
TARGET-50-PAJLTI	0.0733	0.0248	20.7548	5.9910	0.0095	26.0287	8.6294	5.2549	59.6547	41.7342	22.7585	1.8197	189.5835	84.8073	4.9281	0.0498	69.9659	15.1580	81.1188	10.7123
TARGET-50-CAAAAO	0.0462	0.0091	14.1195	1.5998	0.0050	22.2884	11.7458	2.9505	28.2241	64.2852	21.0870	0.5562	197.8252	93.1749	6.2232	0.0320	67.6166	15.8770	133.3404	4.8325
TARGET-50-PAJNCZ	0.0822	0.0166	27.2893	4.6580	0.0096	22.8939	5.8238	4.8365	43.0096	51.5714	20.9778	1.0230	231.0503	86.4801	5.2101	0.0310	59.5939	14.8134	86.3193	7.1326
TARGET-50-PAJMIZ	0.0446	0.0113	12.1465	2.6498	0.0090	32.4896	16.8138	5.0453	29.5023	81.8402	25.1676	0.9359	187.7475	104.7894	7.3786	0.0262	124.1825	16.5945	134.6060	11.3808
TARGET-50-PAJMFY	0.0836	0.0178	37.2634	4.0778	0.0127	22.9562	3.0691	4.3723	41.6629	71.4894	24.8137	1.3962	230.8196	100.4805	4.8081	0.0556	70.6243	16.0396	94.3425	6.8397
TARGET-50-PAKULH	0.0773	0.0128	18.3143	2.5927	0.0067	27.1303	2.5886	2.2935	17.9165	77.0222	23.1827	0.3168	176.6468	102.8181	7.8874	0.0527	126.2306	16.6128	95.6147	2.5991
TARGET-50-PAKVET	0.4103	0.4863	221.7846	74.3087	0.1524	36.1686	5.9400	17.2929	99.8415	137.4821	44.0909	5.9140	194.7347	84.5524	8.6360	0.0843	62.8627	15.6076	95.7312	60.2143
TARGET-50-PAJMEN	0.0529	0.0075	46.5764	3.4813	0.0050	21.5239	4.9218	4.1941	23.0139	73.4395	21.7195	0.9492	195.0331	100.3606	4.5711	0.0355	60.7406	16.2223	111.3500	4.7703
TARGET-50-PALDTE	0.0642	0.0186	30.3682	4.8600	0.0114	23.9555	3.5890	4.0777	57.7916	63.1125	20.5472	1.3757	191.3061	90.4362	5.2102	0.0305	63.8845	14.9384	83.1903	5.7999
TARGET-50-CAAAAH	0.0324	0.0043	5.5035	7.4372	0.0037	45.7789	95.8497	4.1038	22.1601	83.5742	15.7095	0.3976	110.2863	79.6882	9.1891	0.0300	176.7699	14.6885	142.6853	3.4024
TARGET-50-PAJNRH	0.0820	0.0542	39.0468	8.6570	0.0134	23.8147	4.0587	4.2143	41.7239	73.7925	36.5753	0.8206	288.1776	102.3441	5.0584	0.0469	93.6040	18.2171	152.0285	8.4034
TARGET-50-PAJNRL	0.0487	0.0237	18.4642	2.0385	0.0081	31.0074	3.6449	2.1889	32.9751	52.1505	31.4281	0.5544	214.5005	105.0319	7.0041	0.0532	100.8866	17.0577	95.0274	2.5756
TARGET-50-PAJNUP	1.3895	0.3421	194.3453	82.0843	0.0862	70.9672	19.5649	10.8380	49.6879	294.0830	25.8957	3.6758	93.7559	65.8997	20.3475	0.0897	313.9621	12.9355	79.3177	85.9264
TARGET-50-PAJPDC	0.0782	0.0392	12.5803	2.8872	0.0093	18.6392	1.7127	1.3483	12.4738	84.9940	34.3872	0.4608	119.8702	90.2570	4.5604	0.0356	116.5287	16.2594	111.9423	4.8790
TARGET-50-PAJMLI	0.0837	0.0166	31.7682	5.8256	0.0088	22.4108	4.3419	5.2280	52.6358	49.9968	21.2782	1.6103	239.6364	89.6498	4.8902	0.0405	72.8105	14.9112	81.1214	8.7931
TARGET-50-PAJLWT	0.0727	0.0114	26.9470	5.0511	0.0057	15.8095	6.0549	3.4523	27.2990	61.3745	22.1411	0.7875	247.2127	97.9475	3.1580	0.0293	52.9557	16.6929	115.4004	8.3420
TARGET-50-PALEZT	0.0594	0.0228	45.8870	5.8053	0.0094	29.9546	3.9641	6.5838	28.9606	86.9633	31.2106	0.8145	188.7508	91.5712	7.3910	0.0430	99.2921	16.0818	116.9741	7.6797
TARGET-50-PAJLLF	0.1000	0.0454	30.4382	13.7465	0.0161	70.9494	1.4082	2.7879	81.3552	111.0839	29.5670	1.4911	234.3908	104.4653	17.7060	0.0965	243.0639	16.6009	94.6666	8.8187
TARGET-50-PAJLNJ	0.0431	0.0102	11.6427	1.8459	0.0055	23.0128	7.6040	3.2032	25.8991	37.4107	18.3876	0.5027	198.8610	85.3971	4.8474	0.0187	52.1557	14.8827	112.4491	4.8276
TARGET-50-PALFRD	0.0659	0.0143	28.0418	2.7936	0.0062	24.5168	10.1274	4.3418	23.2261	89.7236	24.2643	0.7087	169.9591	90.4321	5.1307	0.0290	84.7870	15.3617	108.0077	4.6092
TARGET-50-PAKKSE	0.1532	0.0210	30.3849	7.5449	0.0091	25.0547	9.0813	4.7603	28.8323	61.0180	16.6106	1.5670	193.3578	77.4744	6.4175	0.0269	55.1458	14.2205	116.2433	17.8374
TARGET-50-PAJLPX	0.1063	0.0359	27.2764	6.1060	0.0132	46.5682	1.8185	5.6145	35.9616	105.5740	28.3443	0.9031	225.3025	87.0136	11.5906	0.0916	116.6810	15.2371	100.2981	4.0826
TARGET-50-PAJMKJ	0.1328	0.0557	32.4730	6.1926	0.0183	28.7996	4.0603	2.9128	30.2642	111.7091	28.9669	0.5092	138.7852	98.9492	9.9277	0.0843	140.8142	16.0597	86.8367	4.8482
TARGET-50-PAKSDG	0.1005	0.0135	23.7192	4.7894	0.0068	16.8771	5.2191	3.1302	30.8165	59.0142	21.7155	0.9776	259.7236	93.1313	3.4772	0.0495	56.0465	16.2901	117.5646	4.0827
TARGET-50-PAKZFK	0.0885	0.0204	38.3515	6.0303	0.0104	28.1733	14.1419	4.6977	54.1300	77.4621	23.9467	1.0240	206.6340	103.4620	6.9074	0.0435	116.7322	16.1935	98.8833	11.5600
TARGET-50-PAKYFC	0.0889	0.0269	34.0307	5.7764	0.0163	31.0993	4.9247	6.1697	49.7624	54.4519	22.8351	1.8625	190.6852	82.7850	8.0152	0.0429	48.7125	14.5717	84.9660	9.9757
TARGET-50-PAJNNR	0.0820	0.0174	29.7396	7.0243	0.0064	29.5422	4.1923	3.0122	37.6627	74.6132	17.0579	1.0158	234.9096	86.4718	6.8508	0.0344	137.7029	15.3615	89.7301	7.2629
TARGET-50-PAJLIP	0.3072	0.0786	125.6696	27.3657	0.0300	39.2354	5.9334	8.5280	123.0074	209.0798	44.1140	2.0381	303.4781	90.9508	15.5342	0.1976	243.5583	16.1916	95.3762	19.1258
TARGET-50-PAJNYT	0.3926	0.0854	42.6394	23.9830	0.0284	29.9384	5.2693	4.4655	49.2913	68.1515	12.7794	2.1614	101.3873	66.5979	9.5941	0.0545	104.9048	12.5732	69.1784	28.2671
TARGET-50-PALGVY	0.0430	0.0122	13.6169	2.5587	0.0055	27.0272	23.6961	3.5421	33.4607	62.4255	20.3846	0.6782	170.8270	88.4288	5.8633	0.0210	53.1174	15.3463	114.2226	6.4302
TARGET-50-PAECJB	0.0301	0.0096	11.8323	1.9367	0.0053	18.6624	2.9673	3.4470	25.1526	28.1710	18.2011	1.1011	184.6270	83.2174	3.5321	0.0226	34.5430	14.7563	98.2567	6.5459
TARGET-50-CAAAAM	42.0321	1.3504	962.9440	172.5863	0.1791	24.6347	34.9099	9.4746	11.0668	490.9675	9.6687	5.8520	34.4312	60.0929	12.6252	0.4902	298.3361	11.5999	72.4563	100.3516
TARGET-50-PAKSCC	0.0539	0.0070	17.1156	1.5703	0.0042	19.9350	2.0344	2.1200	15.4524	51.1572	19.3126	0.3511	228.1431	92.4519	3.4147	0.0236	62.8749	15.3823	107.3946	4.3264
TARGET-50-PAKRZW	0.3001	0.0540	94.5825	12.5592	0.0154	19.8792	9.9118	4.6548	13.7063	121.4474	15.6335	1.5697	104.0543	77.1604	3.8780	0.0532	83.0944	14.3670	108.1776	20.1687
TARGET-50-PAJNSL	0.0604	0.0120	26.8215	3.6073	0.0075	12.0955	6.2159	3.6475	37.5324	52.3051	22.3123	1.5334	195.9084	82.2584	2.5450	0.0372	31.6403	14.4273	85.8874	6.5793
TARGET-50-PAKKNS	0.1565	0.0561	58.9708	12.0993	0.0195	44.2112	6.4603	8.1755	68.1857	134.4631	28.9213	1.5033	292.0796	92.7095	6.1069	0.0647	123.2692	15.7014	98.4384	14.4504
TARGET-50-CAAAAL	0.0948	0.0223	34.9657	5.5514	0.0198	27.0535	10.3284	5.4362	68.7255	72.3440	32.3221	1.6385	242.7431	94.3146	7.3918	0.0342	81.0629	15.6928	113.3198	6.9079
TARGET-50-CAAAAJ	0.0365	0.0092	7.9933	1.5934	0.0058	21.2713	8.5227	3.4041	42.2267	32.4345	19.0995	0.6229	240.5808	87.9839	5.1594	0.0210	47.3854	15.1203	115.0318	4.7501
TARGET-50-PAKNAL	0.1563	0.0394	26.3330	7.0134	0.0182	20.5463	10.8319	4.1171	52.9130	69.0631	25.7154	1.9206	184.3757	98.3428	5.6514	0.0686	174.1093	15.6972	97.6995	19.0675
TARGET-50-PAKXXF	0.0420	0.0085	9.8061	5.6080	0.0038	30.9186	10.5755	3.7944	17.3395	81.0565	17.5496	0.5307	227.6760	81.7548	6.0943	0.0388	56.3710	15.5426	153.8316	2.7644
TARGET-50-PAKECR	0.1707	0.0615	83.6711	11.0330	0.0140	33.8555	2.6938	2.5328	24.4620	157.1768	27.0469	0.6768	188.0204	95.4566	8.4873	0.0911	139.6159	15.9288	122.7309	7.4707
TARGET-50-CAAAAP	0.2306	0.0220	35.7029	10.9653	0.0162	46.9955	6.5200	4.1176	44.3314	92.1553	29.7494	1.4666	223.9834	92.6911	13.4829	0.0980	152.9389	15.6769	74.8854	7.4060
TARGET-50-PAEAFB	0.0522	0.0177	17.7257	5.0656	0.0064	29.6928	3.1913	4.0003	47.4656	44.9319	28.5885	0.8795	257.1257	95.8078	7.6804	0.0568	77.7163	16.7114	135.1256	5.9693
TARGET-50-PAJLKR	0.0649	0.0282	16.2762	3.7025	0.0121	59.3159	8.9827	3.9106	51.8102	94.9304	30.4369	0.8395	379.5282	107.2497	13.4389	0.0527	106.8640	17.0685	96.4322	4.4013
TARGET-50-PAKMSV	0.0254	0.0055	2.6297	1.1089	0.0032	27.7926	10.5653	3.1415	16.2173	29.3043	16.6013	0.3496	163.3518	82.6815	5.3780	0.0206	61.0250	15.2532	115.1105	3.3469
TARGET-50-PALLFB	0.0643	0.0152	34.1073	5.1069	0.0077	29.8313	2.6958	4.9226	50.4557	106.4210	32.2814	0.9864	278.9582	93.6891	8.4694	0.0671	81.2251	15.7087	85.0866	4.0675

Supplementary table 1 continue1

	Nutlin-3a (-)_1047	Mirin_1048	PD173074_1049	ZM447439_1050	Alisertib_1051	RO-3306_1052	MK-2206_1053	Palbociclib_1054	Dactolisib_1057	Pictilisib_1058	AZD8055_1059	PD0325901_1060	Obatoclax Mesylate_1068	5-Fluorouracil_1073	Dasatinib_1079	Paclitaxel_1080	Crizotinib_1083	Rapamycin_1084	Sorafenib_1085	BI-2536_1086
TARGET-50-PAJNAV	162.4690	82.5232	152.5608	21.5751	12.9706	17.8185	14.5013	77.3428	0.8805	4.1699	0.7480	2.6852	6.1115	318.7258	2.8948	0.8091	27.0874	0.2454	16.0558	1.6428
TARGET-50-PAJPEW	61.1511	122.5805	97.6507	21.1144	16.7195	17.1721	22.9884	26.3267	0.1115	3.6184	0.6993	1.0101	3.7852	135.2437	2.1901	0.0484	32.6159	0.0818	17.0263	2.0957
TARGET-50-PAJMLZ	258.3179	200.1884	69.0098	14.9199	19.0397	12.9272	30.5572	100.0159	0.5664	8.5560	1.0162	3.4950	6.4479	550.5548	8.4641	0.1358	47.7836	0.2721	14.3156	1.3507
TARGET-50-PAKRVH	73.2297	261.5925	47.5009	18.2957	9.3736	12.6487	27.6943	49.5275	0.4370	7.5795	0.8960	2.0050	5.8117	84.5491	4.4744	0.0757	27.6544	0.1883	29.4071	1.1552
TARGET-50-PADZUB	54.1478	140.2624	68.9317	19.3026	5.9079	32.3968	28.5526	32.9110	0.3055	5.5618	0.9736	0.6056	6.3785	70.7885	13.8858	0.0331	28.3538	0.2492	17.2999	2.0938
TARGET-50-PADXAY	201.5580	159.6390	160.5788	28.4927	8.1172	22.5905	22.3416	147.4826	0.1843	5.2076	0.7013	3.9643	5.2824	152.6896	4.6191	0.0583	27.8087	0.0575	14.7756	0.5968
TARGET-50-PAJPCM	331.2569	189.0549	36.9002	31.2665	11.9701	17.6677	41.5091	33.2712	0.1273	5.3360	0.9824	1.3354	3.8746	71.8815	11.6119	0.0242	31.1004	0.1015	10.7928	1.0614
TARGET-50-PAJPAR	536.5959	231.9378	70.4364	24.6676	14.6421	23.4307	25.5222	67.9118	0.5290	7.3635	0.9794	6.4661	4.4780	350.1183	11.0590	0.1903	40.1164	0.3483	12.2528	2.0237
TARGET-50-PAJNZS	247.5264	165.1575	68.4580	18.7006	10.8660	17.1512	42.7202	93.6251	0.3952	8.4821	0.8493	1.9914	7.0432	131.7678	7.4420	0.1569	27.8466	0.1764	20.0066	1.3363
TARGET-50-PAJMVC	382.3362	120.5924	60.4813	21.2299	12.2881	6.2912	8.8994	64.1235	0.1129	1.9292	0.7171	2.6454	1.0212	49.2162	0.6513	0.0591	19.6097	0.0439	8.0626	0.3237
TARGET-50-PALGAZ	80.9248	167.0916	49.6953	17.5559	8.8859	11.9414	23.0155	41.5764	0.2165	4.8631	0.8951	1.3990	4.2950	167.2494	6.1996	0.0839	24.9105	0.1384	16.8435	1.1927
TARGET-50-CAAAAR	131.1192	110.6354	37.3033	16.9169	3.4820	12.9780	12.4044	33.5366	0.1579	2.4188	0.8967	1.6605	4.2255	294.0255	3.2937	0.0486	21.6645	0.1172	13.0185	0.7924
TARGET-50-PAKGMU	287.7965	107.0951	86.5652	25.1754	11.6145	14.7329	32.1205	55.7632	0.3694	7.5762	0.8132	1.9384	4.7551	109.0931	4.6033	0.0695	29.7448	0.2122	18.7572	1.2835
TARGET-50-PAKRCC	79.6533	110.6957	43.3704	19.1089	6.2735	16.1938	19.4776	56.2616	0.4553	5.6781	1.0212	1.7922	3.4055	203.3048	9.1760	0.0534	16.3284	0.2375	9.4929	0.7453
TARGET-50-PAKFME	83.8400	130.1067	52.8956	16.4339	4.4321	16.1408	20.5689	38.4707	0.2420	4.4713	0.9114	1.2874	4.4198	178.4942	6.3791	0.0587	22.2791	0.1275	13.2905	1.2964
TARGET-50-PAJNNC	34.8791	117.4060	61.7989	21.4471	8.0745	19.1127	12.4577	36.8007	0.1543	3.3691	0.9010	1.3518	6.0429	123.1019	8.6237	0.0517	34.5687	0.0977	16.3406	0.9928
TARGET-50-PAJNJJ	86.7787	115.8740	143.9965	27.5251	14.0472	21.0570	36.9663	56.0912	0.2657	4.0504	0.9168	2.5836	6.2617	320.4274	7.0720	0.4765	39.9114	0.1659	17.9113	1.3408
TARGET-50-PAKJGM	55.8566	82.4794	41.3686	19.9106	2.2471	13.2344	15.2052	13.4865	0.0720	3.0874	0.6709	0.5778	2.4304	12.7406	1.1075	0.0109	9.8010	0.0372	11.8750	0.9174
TARGET-50-PAJNGH	52.6388	143.1376	36.8447	14.1716	5.3174	15.5518	24.9592	73.4407	0.3197	4.9760	1.0151	1.5940	4.0123	136.1182	9.3243	0.0815	18.0926	0.1567	16.4522	1.3400
TARGET-50-CAAAAC	95.6916	324.9516	47.3135	14.5529	12.5927	23.2054	22.1789	75.0072	0.3595	7.9670	0.9621	2.0969	4.2538	146.2345	49.7328	0.0994	26.8197	0.1568	15.7951	1.3319
TARGET-50-PAJNBN	46.4687	91.3260	16.4886	18.9435	2.9725	18.0881	20.4616	22.2022	0.1938	4.2989	1.1403	1.8795	4.9377	93.0053	2.9043	0.0300	22.6494	0.1317	9.5546	0.4916
TARGET-50-PAKYLT	238.1356	210.2959	48.4748	13.3596	17.0509	8.7620	24.6277	68.9249	0.3785	6.1747	0.7937	4.9090	5.4583	99.4010	8.7532	0.0541	34.2714	0.0929	17.1810	0.7996
TARGET-50-PAJMKN	111.8524	154.3335	81.5623	22.3768	12.7370	19.6842	20.0900	35.5428	0.2478	3.2219	0.8331	2.1429	4.8494	163.8579	1.4858	0.2274	33.2892	0.0950	21.3427	2.4615
TARGET-50-PALERC	98.8721	86.0561	35.8620	16.6103	3.4921	12.2647	15.2942	21.2492	0.1329	3.6112	0.8651	1.2269	3.3354	67.3770	2.0054	0.0349	21.9978	0.0752	11.0348	0.8194
TARGET-50-PAJMFU	47.4143	109.0848	37.0681	27.9715	7.7425	19.8906	30.5006	29.0620	0.2398	7.1547	0.8956	1.4071	5.2073	63.3382	2.7730	0.1031	34.2833	0.1718	15.4644	1.8961
TARGET-50-PAKZHF	45.7888	78.2276	49.0155	22.0004	3.9582	23.3637	19.7123	30.2700	0.1805	2.7081	0.8458	0.8415	3.8583	64.3084	3.2414	0.0383	16.3913	0.1468	12.1338	2.4891
TARGET-50-PAJLSP	73.1751	225.5783	59.3011	16.0022	4.6921	17.8800	21.6398	38.2491	0.2694	4.6347	0.8696	2.2466	4.9364	108.3507	16.9700	0.0366	22.6736	0.1630	16.3827	0.9304
TARGET-50-PAJMKI	155.5529	133.1310	41.3328	15.1452	7.1071	8.3438	24.4861	56.3791	0.1240	4.5175	0.9330	1.5955	3.9947	106.5074	5.7045	0.0303	17.1650	0.1170	15.9976	0.7805
TARGET-50-PAKUIT	221.8322	135.6859	56.2505	22.6298	14.8728	16.9005	23.9412	39.8934	0.1779	8.0078	1.0207	2.3513	3.3606	93.5833	18.8188	0.0613	31.7995	0.1713	14.8649	0.9660
TARGET-50-PALDWP	81.7390	98.3279	26.9139	15.4671	2.9230	12.9605	21.5158	27.5280	0.1842	5.4112	0.9590	1.1210	3.0740	57.8308	7.3798	0.0225	13.3662	0.1081	9.2157	0.5347
TARGET-50-PAJMJT	65.7611	179.3910	46.6658	15.6763	7.6798	18.0759	14.2923	26.8971	0.1361	4.0122	0.8962	1.0657	3.6724	68.3601	16.6408	0.0394	33.8482	0.0855	15.7200	2.1331
TARGET-50-PAKWPM	31.5988	84.6724	79.8449	20.0508	6.4428	20.2581	24.0612	30.9738	0.2351	3.4082	0.8556	0.9070	4.2392	38.1414	4.4422	0.0494	22.4363	0.0729	12.6398	1.9451
TARGET-50-PAJLKC	110.9086	113.4517	37.8652	14.8662	3.4070	23.3968	19.2941	44.0353	0.3917	4.7240	0.9122	1.5012	4.9929	97.3645	20.1854	0.0183	19.3113	0.1881	11.5756	1.2762
TARGET-50-PALKRS	116.1464	82.5630	125.4559	24.8351	7.7283	10.5525	13.9585	29.9308	0.0916	1.7793	0.6126	1.8408	2.4713	56.1054	0.9803	0.0356	25.4546	0.0601	14.7135	0.8516
TARGET-50-PAKNRX	687.1738	466.0666	83.1154	27.7610	41.0359	18.4267	71.3999	149.5544	0.3943	9.3198	0.9710	3.4333	8.9453	115.0859	6.9470	0.4515	60.4391	0.1371	32.4408	1.9645
TARGET-50-PAJNTJ	118.3058	160.7852	66.8900	17.2858	6.1989	20.0377	27.1939	31.3551	0.1869	4.7766	0.8994	1.4569	5.5568	104.2516	7.6805	0.0744	25.6843	0.0911	17.1023	2.3288
TARGET-50-PAJNUS	76.1807	139.7132	39.9351	17.7515	4.3438	21.0911	12.8556	25.4076	0.1372	2.9289	0.8875	1.7328	4.2991	94.3414	9.1074	0.0312	27.4943	0.0932	11.9586	1.6850
TARGET-50-PAKNTW	30.5237	59.7030	36.9481	21.7091	0.9400	15.2955	13.4301	5.3345	0.0580	2.6577	0.8019	0.2926	2.2700	21.8432	4.8596	0.0060	7.4055	0.0473	5.3935	0.6174
TARGET-50-PAJPAU	66.2834	115.2321	61.8327	18.0113	6.1373	31.4636	33.1135	38.6892	0.2613	4.0689	0.9905	0.8381	3.4386	98.2946	5.8880	0.1830	26.2049	0.1285	24.5737	2.2518
TARGET-50-PAJMVU	116.5224	165.5059	64.8210	34.1047	10.3290	39.6177	24.8486	31.1334	0.1531	3.8304	0.8906	2.8035	4.9869	152.4578	7.8407	0.0617	31.1882	0.1828	18.0112	1.4077
TARGET-50-PAJNZI	147.7772	89.5998	31.7575	22.9403	6.7027	21.3298	20.5134	52.6115	0.2295	5.5213	1.0081	2.7084	3.4615	110.3253	15.1764	0.0381	21.8532	0.1440	8.3757	1.0596
TARGET-50-PAJPDN	126.5338	88.1312	106.9321	25.7436	7.5498	8.2513	29.9060	44.3015	0.1105	4.3800	0.6184	1.4480	1.8585	59.2662	2.7617	0.0915	36.3211	0.0405	14.9956	0.6118
TARGET-50-PAJNVE	157.5006	107.2579	19.4986	12.3457	4.4785	13.8725	20.2360	41.1348	0.2298	5.6324	1.0958	2.6105	4.7377	202.9186	20.4063	0.0273	21.1114	0.1700	10.7696	0.7969
TARGET-50-PAJMSE	63.7999	114.7218	42.1811	19.8817	3.5602	17.5353	18.1755	56.9351	0.3045	6.2765	0.9138	1.5536	3.3441	170.6858	5.5881	0.0823	24.3715	0.1684	14.0384	2.1189
TARGET-50-PAJNZU	76.1427	124.4647	33.9608	14.4753	4.7427	11.6887	21.1336	40.5042	0.2277	3.4528	1.0233	1.6229	4.4590	255.3587	2.8120	0.0729	29.1918	0.1944	16.4836	0.9962
TARGET-50-PALGLU	48.5690	75.8449	62.0912	18.2286	3.5060	8.0129	10.6755	20.1423	0.0870	1.3270	0.8014	1.1027	2.6584	125.7181	7.5546	0.0241	19.5796	0.0381	7.5429	0.6221
TARGET-50-PAJNZK	118.4340	122.6494	43.7519	15.1850	4.3719	18.1957	20.8481	39.3339	0.2382	3.8174	0.9194	1.5904	3.3707	105.0588	18.5505	0.0774	21.7667	0.1814	12.9685	0.4124
TARGET-50-PAJMUF	109.0756	61.8203	29.4312	16.4663	1.8514	15.7399	12.5387	17.4763	0.0793	2.2436	0.7788	1.1642	2.1527	58.0766	1.6911	0.0218	13.5673	0.0933	7.8947	1.1364
TARGET-50-PAJMRL	381.4990	227.7411	66.8607	21.8968	6.1514	18.2536	63.9813	86.0704	0.5875	12.4068	0.9342	2.7361	6.8253	125.9348	22.9332	0.0192	42.4167	0.1781	15.6600	1.5284
TARGET-50-PAJMXF	70.2567	103.7313	47.8488	18.0105	6.3924	18.2939	13.7696	36.6854	0.1780	3.2276	0.8809	1.6237	4.3956	142.6886	4.4044	0.0454	29.7768	0.0924	13.9334	1.8868
TARGET-50-PAKPDF	133.0510	133.3238	25.8364	20.3828	4.5351	18.8767	27.0579	68.3792	0.3532	6.7801	1.0312	2.2188	4.0419	282.3718	9.0408	0.0461	19.7254	0.1436	11.5502	1.3551
TARGET-50-PAEBXA	56.0177	84.4009	54.0464	28.0549	6.6932	22.8661	18.9983	27.7076	0.1417	2.9776	0.7938	1.7823	3.9653	112.9378	2.2159	0.0912	30.2956	0.1413	13.9383	1.7153
TARGET-50-CAAAAS	91.0893	242.0300	63.5246	13.4338	11.1694	18.1349	19.7943	123.5749	0.4123	6.7610	0.9756	1.8516	5.1544	629.1766	31.1552	0.2268	39.1068	0.1648	24.5630	1.2716
TARGET-50-PAJNLT	59.1033	102.1245	61.7482	13.4195	5.1592	13.0329	23.3453	40.2343	0.1969	5.0666	0.8376	1.9440	2.8302	181.2852	5.9025	0.0420	22.3829	0.1057	15.9170	0.7667
TARGET-50-PAJPHA	178.0311	116.3730	56.5960	12.8760	3.6222	13.1132	13.6804	32.2544	0.1042	3.1787	0.7993	1.6828	3.1987	184.7647	7.4126	0.0554	15.7035	0.1003	18.2212	0.9834
TARGET-50-PAJMMY	96.2055	75.0762	44.5617	11.0163	1.3265	10.0267	10.4344	68.3203	0.1499	3.1769	0.9149	2.2038	2.2740	215.8165	3.9765	0.0202	12.6596	0.1412	13.5354	0.4565
TARGET-50-PAJLTH	41.7715	104.2540	69.5394	20.2081	6.3622	24.0565	17.0792	26.9237	0.2076	3.1473	0.8429	0.6106	4.0048	109.7267	9.9612	0.0498	23.3589	0.1104	17.3060	1.2105
TARGET-50-PAJNCC	137.9458	69.1779	60.1098	11.1284	13.2854	12.3692	11.9855	27.2597	0.1723	3.3105	0.7928	1.5423	3.1629	81.2880	15.5102	0.0645	23.8232	0.0987	10.8889	0.6568
TARGET-50-PAJLUJ	34.6829	56.0630	34.9450	19.7216	1.9659	18.5426	9.6693	10.6155	0.0710	1.5394	0.7186	0.8689	3.3335	14.3808	0.8665	0.0213	15.8263	0.0387	8.9583	1.7015
TARGET-50-PAJNCJ	101.3795	99.8880	41.0210	23.2095	3.5775	12.7427	17.4567	43.6026	0.2999	4.0114	0.9247	2.0722	4.2594	334.1492	7.8350	0.0573	27.6871	0.1822	11.3247	1.5412
TARGET-50-PAKZER	99.7068	60.5649	67.0091	19.9602	2.2636	18.4194	9.6877	29.2714	0.0788	1.4543	0.8035	1.6163	2.7157	116.4381	4.5756	0.0383	17.4692	0.0839	7.3641	1.0891
TARGET-50-PAJMEP	80.0676	102.7891	50.3633	13.0892	2.0812	11.3811	19.2657	46.2631	0.2642	4.1284	0.9244	2.0667	3.5488	205.8402	6.3208	0.0314	13.8844	0.1803	12.1360	0.5297
TARGET-50-PAJMEL	32.6895	97.0606	38.7386	14.9278	6.2782	10.8536	14.0115	32.9700	0.1042	2.2236	0.8300	1.8543	4.4770	278.1031	1.9927	0.0652	31.7637	0.0911	12.7154	1.2890
TARGET-50-PAJNVX	293.3802	385.1719	54.2866	17.0002	69.9470	10.3485	27.5093	172.6088	0.3273	3.9862	0.7460	6.1228	14.0293	772.3732	4.6097	0.6540	51.6200	0.1623	20.7349	1.6373
TARGET-50-PAKXWB	73.4244	84.1763	50.0108	16.2827	5.8652	18.7254	11.7942	35.1373	0.2092	2.2695	0.7002	1.0647	4.6170	56.8077	0.6088	0.1100	16.8718	0.1028	17.9318	1.7390
TARGET-50-CAAAAB	135.2082	133.0565	46.7288	15.8933	6.5357	21.2844	18.1513	54.4696	0.3794	5.0038	0.9252	1.6035	4.7556	135.4844	8.7081	0.0527	21.1099	0.1892	15.0435	0.9667
TARGET-50-PALJIP	99.4726	93.1544	47.7394	12.1161	6.0482	15.6033	8.5341	47.3170	0.1454	2.3059	0.8408	0.7988	3.7488	175.2729	3.9576	0.0279	22.1871	0.0961	14.5034	1.1038
TARGET-50-PAKMUB	113.8561	118.3943	93.7203	16.9998	11.6474	15.8704	14.3391	58.0072	0.1471	2.3919	0.7124	2.0641	4.4009	384.1619	2.6826	0.3576	32.1851	0.0897	20.6917	2.7060
TARGET-50-PALKCW	46.7236	76.7857	49.2747	27.1580	5.7977	19.8179	22.2746	32.9830	0.1754	3.2488	0.8199	1.1861	4.1054	84.2430	2.4337	0.0706	26.3351	0.0903	10.9579	2.5100
TARGET-50-CAAAAQ	58.1471	83.4283	33.6784	19.4015	1.9163	18.7671	11.5322	15.2232	0.0925	2.7047	0.8977	1.4167	2.9275	57.4805	6.9201	0.0138	12.6385	0.0764	7.6028	0.5782
TARGET-50-PALFME	67.9604	107.7682	44.9501	23.9095	4.4154	27.3600	24.7677	36.8408	0.2384	6.4013	1.0211	1.4004	4.3794	124.3904	18.3275	0.0558	24.4085	0.1981	12.4180	1.0801
TARGET-50-PAKNXS	607.9549	250.6311	110.5375	19.3406	18.4367	15.4808	33.3390	93.8771	0.5260	8.9983	0.8907	3.3163	4.8622	126.0535	5.6790	0.1410	43.8157	0.1386	26.0255	1.0202
TARGET-50-PAJNAA	593.9474	399.9220	189.5273	28.5235	75.7920	28.8534	59.8645	98.9737	0.7606	11.0018	0.7862	4.5183	10.2924	1123.5533	8.0923	3.7882	127.5742	0.2878	66.1688	3.5522
TARGET-50-PAJPGY	96.5544	78.2461	71.7063	17.4087	2.4821	9.6534	10.0743	44.2165	0.1064	1.5421	0.7902	1.5833	2.9961	335.8078	9.7182	0.0507	19.3183	0.0940	10.5777	1.3557
TARGET-50-PAJNEC	86.7691	63.8796	352.1579	41.0562	8.4635	16.3059	24.7733	15.3277	0.0395	2.3533	0.5470	0.7737	1.1557	4.9054	0.3501	0.0115	17.5899	0.0396	30.2399	0.3923
TARGET-50-PAJNDU	132.6534	35.5453	108.6365	24.7626	2.9512	4.8151	10.1428	5.8999	0.0332	1.2795	0.5532	0.7817	0.9235	14.4890	0.1392	0.0077	17.1535	0.0115	7.7974	0.1456
TARGET-50-PAKGZX	20.7848	77.2404	28.9225	20.3381	3.6252	15.8416	25.8534	28.4553	0.1882	2.8570	0.7577	0.5236	4.0677	68.9278	0.2452	0.0871	13.2140	0.1590	17.1091	0.9313
TARGET-50-PAKFYV	180.7527	175.4107	31.0680	17.9891	13.9502	12.0848	54.8690	48.2951	1.6838	16.0856	1.0363	2.3571	4.3062	198.8965	7.3688	0.1323	18.7474	0.4530	13.4258	0.7405
TARGET-50-PAKGED	334.5116	33.3588	386.6957	33.4544	7.5979	8.0460	5.6406	19.1262	0.0265	0.8209	0.5174	1.5138	0.8147	92.1096	0.2321	0.0217	32.4080	0.0207	18.4916	0.1865
TARGET-50-PAJLTI	71.9983	139.4037	57.5845	16.3485	3.0082	13.2940	21.6017	33.4427	0.2026	3.6870	0.8727	2.3580	4.4659	122.7038	6.8712	0.0594	25.8838	0.1539	16.8048	1.3037
TARGET-50-CAAAAO	53.4111	69.8152	49.7925	25.7580	5.4393	16.4942	21.3477	20.9389	0.1511	3.8846	0.8144	0.8849	3.9194	42.7880	1.8617	0.0311	23.4051	0.0966	9.9857	1.2719
TARGET-50-PAJNCZ	54.1831	112.1164	29.9216	14.8799	5.2754	13.5891	23.0135	35.9416	0.1942	4.0483	0.9562	1.7430	4.5321	135.8098	2.7691	0.0529	24.2509	0.0999	12.9426	0.8843
TARGET-50-PAJMIZ	79.9653	107.6469	55.0911	28.0624	6.2615	14.5060	25.3174	23.3733	0.1808	4.2421	0.7491	1.1665	4.2315	18.1019	2.2127	0.0260	18.5837	0.0674	14.3806	1.7454
TARGET-50-PAJMFY	41.3384	103.4615	28.2842	21.2139	5.4955	18.6047	22.9894	38.3152	0.3152	5.4570	0.9793	2.1809	4.9401	127.9838	4.2501	0.0694	27.7080	0.1887	13.9685	1.3791
TARGET-50-PAKULH	37.3201	80.2539	45.6659	20.6249	3.7394	17.5020	12.8921	44.9399	0.2457	3.6671	0.7779	1.2646	3.2133	45.4067	11.9259	0.0389	15.9625	0.1156	7.2635	1.6321
TARGET-50-PAKVET	268.1090	323.1441	91.0528	18.8256	23.2151	23.8311	40.8305	128.1934	0.8475	6.8124	1.0669	3.0234	5.5379	591.3110	1.9449	1.9441	50.5786	0.2271	44.5166	3.8262
TARGET-50-PAJMEN	79.5757	64.4643	55.3916	24.8407	5.5180	16.6864	15.0016	19.4426	0.1667	2.6070	0.8637	1.4446	3.7915	70.1797	3.5136	0.0284	32.2353	0.0708	12.0820	1.4244
TARGET-50-PALDTE	64.0514	166.4281	40.2864	15.2970	4.6589	11.1977	23.5878	43.1164	0.2798	6.6654	0.9563	1.5808	3.8896	112.4735	4.1630	0.0594	17.2492	0.1767	15.0460	0.9042
TARGET-50-CAAAAH	241.3005	48.3536	177.9925	37.9286	5.9332	8.3997	19.7614	24.3380	0.0823	4.5639	0.5798	1.1404	1.3606	37.6477	5.9967	0.0126	23.9702	0.0324	13.4984	0.2528
TARGET-50-PAJNRH	178.8680	77.7639	55.3199	31.6591	9.0896	42.2925	31.7666	33.5056	0.4048	4.7489	0.9706	1.4317	3.8520	28.8909	2.4076	0.1116	35.5694	0.1378	18.4198	3.0929
TARGET-50-PAJNRL	83.9505	69.1896	54.3381	23.2951	3.0301	32.9080	29.1200	47.8183	0.3074	4.8969	0.9535	1.0154	3.9851	111.4551	7.6149	0.0444	11.9012	0.2166	10.5080	1.2113
TARGET-50-PAJNUP	520.3912	408.6261	186.1229	32.0397	62.3520	12.6657	36.0021	108.8814	0.3767	6.2190	0.6117	3.5868	7.1141	486.9248	5.5947	0.9825	75.8346	0.2032	53.2050	2.3058
TARGET-50-PAJPDC	42.3279	110.4336	43.1497	29.4595	4.8053	25.3051	15.7929	37.5872	0.1510	2.5655	0.8544	0.3407	4.8780	121.3112	3.1602	0.0895	11.5397	0.1510	8.5922	2.4271
TARGET-50-PAJMLI	75.4375	111.2834	33.1740	16.1057	3.3181	10.6532	18.4084	45.1626	0.2608	3.9086	0.9349	2.4886	3.6915	195.6830	4.7306	0.0374	21.2187	0.1290	10.8517	0.8224
TARGET-50-PAJLWT	133.9864	80.3408	50.1894	21.5450	7.1295	22.1435	19.0171	18.2213	0.1488	3.9038	0.8025	1.5195	3.8441	46.1759	2.6820	0.0306	26.8390	0.0909	10.1925	2.0774
TARGET-50-PALEZT	132.4487	95.6319	49.5188	24.2727	8.6919	19.8207	35.2015	58.9273	0.3205	6.2994	0.8416	1.7871	5.7215	54.2021	3.0218	0.0787	24.0639	0.0913	14.9654	2.3493
TARGET-50-PAJLLF	359.9147	206.0841	100.5624	31.0767	10.8910	14.6189	64.9573	49.7447	0.3623	9.0526	0.8370	1.6734	6.7354	106.9957	24.4633	0.1064	22.7217	0.4244	20.7205	1.2356
TARGET-50-PAJLNJ	79.7103	66.6140	36.9680	17.6157	2.3150	14.4696	14.8522	15.6812	0.1104	2.8687	0.7905	0.7279	2.8704	53.6354	2.2449	0.0280	16.2221	0.0907	9.5188	1.0454
TARGET-50-PALFRD	79.7153	53.8855	53.1913	23.8721	6.2524	20.7408	14.0344	27.5886	0.1462	2.1637	0.6874	1.1779	3.1797	68.7780	1.8619	0.0379	20.5396	0.0735	10.5977	1.8352
TARGET-50-PAKKSE	92.0163	94.9509	44.5693	15.7214	4.4068	12.5504	17.1106	24.3791	0.1306	2.5280	0.7826	1.5697	4.9497	119.4771	1.9709	0.0558	25.3395	0.0748	13.6179	1.2473
TARGET-50-PAJLPX	185.7763	154.6225	88.0293	20.1281	5.3679	10.6136	33.8698	61.0336	0.1841	5.1854	0.8813	1.8780	2.8343	266.3180	28.9105	0.0949	22.9989	0.1317	13.3607	0.5441
TARGET-50-PAJMKJ	75.0334	100.8335	72.8553	24.6403	7.9150	17.9983	10.1691	43.8199	0.1246	2.1774	0.7489	1.5627	3.3205	73.6861	7.1301	0.1110	15.5551	0.1069	9.2466	2.5484
TARGET-50-PAKSDG	110.7795	121.6901	28.3237	17.4250	4.0796	20.7492	12.9053	31.8030	0.2042	3.4878	0.9413	2.5210	3.8007	170.2906	4.8344	0.0377	22.0750	0.1507	13.5833	0.8543
TARGET-50-PAKZFK	64.3365	88.0727	46.0357	23.3685	6.0351	16.7079	21.6193	39.1161	0.2985	4.5002	0.8347	2.4244	5.4107	96.0449	4.0185	0.0400	26.3161	0.1434	12.2002	1.6778
TARGET-50-PAKYFC	51.7314	103.2454	51.2315	15.1142	4.8478	11.5455	27.2283	35.3145	0.1893	4.0951	0.9171	1.4743	4.0793	213.4191	5.1272	0.1215	27.0129	0.1433	12.7562	1.2280
TARGET-50-PAJNNR	457.2804	115.2451	67.5145	18.0242	8.7364	12.7717	33.1571	58.6754	0.2238	6.6977	0.8562	1.2956	5.1379	126.1174	10.6432	0.0394	27.3231	0.2476	19.9442	1.3841
TARGET-50-PAJLIP	614.4334	303.2612	100.1524	16.3685	46.6988	19.2408	47.4631	204.9350	0.8728	13.9418	0.9870	6.1552	7.6618	190.3363	16.9164	0.1522	45.4506	0.2410	35.5971	1.4654
TARGET-50-PAJNYT	106.9896	145.7993	56.5937	13.9989	9.2586	5.8879	22.7607	81.6824	0.3090	4.5905	0.7673	2.9682	6.8281	403.7471	12.1199	0.4703	47.4984	0.1573	14.4280	1.4084
TARGET-50-PALGVY	42.6047	69.6548	52.1652	27.2038	5.4786	20.4005	16.7130	14.2838	0.1033	2.1928	0.7829	0.9861	3.6818	54.6607	2.3177	0.0255	27.3245	0.1032	10.7548	1.3207
TARGET-50-PAECJB	49.5919	73.0975	26.0123	15.0749	1.5991	13.0209	11.3155	22.1397	0.1120	2.3538	0.8697	1.1562	2.9576	67.2289	4.1625	0.0394	15.6334	0.0706	7.8187	1.0636
TARGET-50-CAAAAM	136.0433	637.0830	459.1496	22.5051	123.2375	5.1041	10.0796	113.6948	0.2283	4.3642	0.4826	4.9608	17.1930	831.7394	17.2259	0.7136	91.9322	0.0362	21.0736	2.9418
TARGET-50-PAKSCC	146.3835	61.2044	33.1289	21.0711	3.5537	13.2847	12.7115	15.1414	0.0893	3.3072	0.7876	1.2722	3.0150	33.8522	2.0929	0.0227	17.8338	0.0557	7.5584	0.6113
TARGET-50-PAKRZW	129.5795	100.0384	99.3223	19.9312	17.5938	13.0475	11.1624	24.7284	0.1438	2.3180	0.6929	1.6160	6.1790	70.4138	1.2280	0.1420	28.5495	0.0619	14.2786	2.1991
TARGET-50-PAJNSL	99.0725	95.5795	36.4697	16.0073	3.0971	11.7568	14.2145	27.5259	0.1790	3.6944	0.9385	1.4875	3.2548	139.4808	2.9178	0.0426	21.7597	0.1196	12.1694	0.7401
TARGET-50-PAKKNS	126.0589	136.3706	94.3942	23.6898	19.2451	17.3677	41.5108	69.2183	0.3778	5.7560	0.8870	1.4934	5.5686	239.8163	4.0501	0.1728	32.1558	0.1508	20.0213	1.7007
TARGET-50-CAAAAL	58.2141	148.5575	38.8330	24.6876	9.2880	19.4698	28.5249	36.8966	0.2216	5.5368	0.9920	2.3183	5.3762	195.8686	3.4652	0.1526	48.4724	0.1771	19.0315	1.3740
TARGET-50-CAAAAJ	89.8608	86.8912	33.8810	17.8877	3.1384	15.5092	20.2851	15.9727	0.1112	3.6000	0.8853	0.8438	3.9409	72.5195	4.5962	0.0237	21.5202	0.0989	11.4766	0.7791
TARGET-50-PAKNAL	85.6441	176.8861	68.6178	23.9316	8.9508	16.7190	20.1412	41.7318	0.1708	3.1421	0.8861	2.7378	7.2035	159.7435	9.3076	0.1090	31.2805	0.0805	14.6609	1.3840
TARGET-50-PAKXXF	422.7743	73.0929	73.0347	26.6830	3.6911	12.3687	24.6490	21.5599	0.1250	7.0536	0.7094	1.5618	1.8719	46.6189	9.1002	0.0102	23.9902	0.0842	10.1478	0.8535
TARGET-50-PAKECR	85.5798	126.2151	93.5277	33.6320	11.1689	17.5628	14.7700	26.5513	0.1267	1.9040	0.7734	1.6277	6.7365	218.3763	7.8508	0.1390	31.2219	0.1203	10.2973	1.7953
TARGET-50-CAAAAP	85.7108	127.4042	43.3880	19.8351	3.7780	15.2258	35.8992	61.4340	0.5235	9.0360	0.8777	2.5435	5.1770	78.9405	20.1549	0.1011	20.0400	0.1968	12.0778	1.0573
TARGET-50-PAEAFB	25.8058	86.4633	62.9883	28.0228	2.4960	20.8946	25.9448	23.0042	0.1214	4.4391	0.8390	1.4991	3.4380	61.7351	6.7641	0.0555	27.0999	0.1300	10.3883	1.6954
TARGET-50-PAJLKR	64.7819	108.4635	84.1965	22.3762	11.3769	31.7515	26.4621	53.3377	0.3876	5.9011	0.8939	0.9461	5.7704	116.0693	14.1627	0.0597	21.7648	0.2232	19.8165	1.2760
TARGET-50-PAKMSV	83.1691	72.2157	49.1560	18.8742	1.7401	12.1421	16.1886	12.4883	0.0726	4.1275	0.6669	0.7091	2.0140	7.1897	3.1942	0.0100	9.0913	0.0326	9.7419	0.6336
TARGET-50-PALLFB	241.9987	106.0883	44.8469	18.1693	16.6964	15.3993	44.9445	69.2301	0.4325	8.3877	1.0186	3.7131	4.5722	127.9121	8.1021	0.0385	35.5186	0.1804	12.7329	0.6614

Supplementary table 1 continue2

	Irinotecan_1088	Oxaliplatin_1089	BMS-536924_1091	GSK1904529A_1093	Tozasertib_1096	PF-4708671_1129	PRIMA-1MET_1131	Erlotinib_1168	Niraparib_1177	MK-1775_1179	Dinaciclib_1180	Gemcitabine_1190	Bortezomib_1191	GSK269962A_1192	SB505124_1194	Tamoxifen_1199	Fulvestrant_1200	EPZ004777_1237	YK-4-279_1239	Daporinad_1248
TARGET-50-PAJNAV	81.0702	93.1436	10.9173	114.3859	27.9759	40.3342	57.7349	12.3633	170.3210	3.8395	0.0827	0.4180	0.0118	13.8929	9.7293	58.2603	24.9760	218.4916	49.3870	0.0265
TARGET-50-PAJPEW	15.2104	33.2165	13.0990	138.8878	23.7267	41.8736	223.7027	14.7200	128.9496	2.8030	0.0749	0.2920	0.0084	15.6991	10.8521	48.6383	26.1577	233.1492	13.8719	0.0109
TARGET-50-PAJMLZ	21.3291	88.6032	11.4641	84.5667	16.6137	50.5401	116.5640	15.7193	152.3538	3.0220	0.0585	0.7936	0.0085	19.8303	11.1916	41.6899	16.7928	196.4504	18.6489	0.0194
TARGET-50-PAKRVH	20.8496	52.4928	8.5044	101.9893	18.0171	66.4962	109.1042	12.1761	67.8117	2.7726	0.0597	1.1751	0.0080	22.2513	8.8386	47.5973	21.9862	195.0268	16.1535	0.0091
TARGET-50-PADZUB	9.0152	22.4885	5.9354	95.1247	21.6862	44.7421	186.5801	17.7108	98.2265	1.4519	0.0818	0.1925	0.0076	20.9644	13.0600	35.9627	20.4701	166.1790	5.4709	0.0112
TARGET-50-PADXAY	42.8303	64.0649	30.5811	68.4739	23.2496	75.7456	83.7370	23.0010	198.6378	0.7346	0.0976	0.7142	0.0045	19.1502	9.6188	37.0274	29.4773	231.7695	8.8783	0.0721
TARGET-50-PAJPCM	16.5266	37.2790	5.3677	59.0224	18.3787	79.6227	97.9453	11.4766	119.5623	0.5885	0.0833	0.3478	0.0043	33.9285	8.1219	20.6227	16.0383	140.3105	4.9748	0.0082
TARGET-50-PAJPAR	30.0146	73.7212	9.4703	55.6706	22.4810	56.9831	206.2875	9.1216	159.6770	2.0800	0.0658	2.5846	0.0074	25.3245	12.8120	32.9064	19.7679	254.7975	17.9100	0.0175
TARGET-50-PAJNZS	22.5845	58.0204	9.7620	79.0089	21.3079	47.1289	231.2173	13.5958	175.2411	3.9482	0.1313	2.9970	0.0066	18.1257	9.8386	39.3080	20.6472	198.3798	15.5027	0.0571
TARGET-50-PAJMVC	10.3198	28.7636	10.4700	35.6235	16.2369	51.8986	116.5417	10.0219	69.5858	2.8315	0.0203	0.2917	0.0139	17.0259	5.4217	37.1147	15.5986	124.3429	12.7983	0.0018
TARGET-50-PALGAZ	24.6289	81.9284	7.0793	68.9474	18.8493	56.9292	109.1273	17.2063	78.9936	1.9588	0.0753	0.6180	0.0078	22.3915	9.8285	33.2122	21.1633	173.4731	10.7308	0.0117
TARGET-50-CAAAAR	13.8685	52.7156	6.4277	74.7403	14.0884	55.7636	105.6060	10.6940	69.8683	2.1466	0.0620	0.6481	0.0086	17.2976	9.2933	33.0340	15.7477	139.3311	6.6669	0.0083
TARGET-50-PAKGMU	20.6354	64.1125	10.2397	123.4936	18.4092	54.0316	104.6238	22.9261	107.5355	2.7195	0.0623	0.4979	0.0083	23.4561	9.1231	59.6455	21.5136	234.2971	15.8652	0.0114
TARGET-50-PAKRCC	26.0122	39.3081	9.3751	69.1400	20.8856	64.4831	62.0417	12.7275	88.8546	0.8231	0.0639	1.0211	0.0053	21.5522	10.4170	25.7449	12.5501	138.3949	8.2027	0.0085
TARGET-50-PAKFME	17.3795	50.7280	10.3104	75.3882	15.2945	56.8691	121.6194	13.4190	83.9676	1.4782	0.0678	1.1635	0.0061	21.4699	10.5015	37.6781	19.6215	159.7775	8.5447	0.0211
TARGET-50-PAJNNC	11.0905	34.8467	9.9005	97.9662	23.2056	65.2383	68.1574	15.2083	59.0958	1.7312	0.0740	0.2059	0.0087	24.7822	7.7947	27.3030	15.2573	177.8323	10.8006	0.0089
TARGET-50-PAJNJJ	21.0847	66.9347	9.1044	109.2890	18.6896	53.8174	318.1759	14.1561	113.8556	3.8663	0.1318	1.4086	0.0079	21.7721	9.1999	50.4735	27.2890	334.9881	27.6047	0.0237
TARGET-50-PAKJGM	2.9936	15.2179	5.9987	47.1522	12.3957	36.8537	37.3511	10.7722	25.3313	0.7045	0.0270	0.0492	0.0054	12.3819	8.1982	28.2853	12.6201	111.8495	2.7667	0.0052
TARGET-50-PAJNGH	8.0547	46.2576	7.5385	60.8251	20.5860	50.3962	138.6180	12.9002	57.3983	1.7323	0.0606	0.4394	0.0072	17.8460	8.3383	32.5077	16.4404	128.1862	11.8574	0.0276
TARGET-50-CAAAAC	20.5616	57.7494	12.8417	50.8818	14.0953	69.1022	48.4755	16.5942	45.2389	1.8425	0.0611	0.9153	0.0072	23.1028	9.9642	44.3085	19.2278	145.4713	28.9334	0.0300
TARGET-50-PAJNBN	10.2789	22.7804	4.8391	47.6442	13.7240	66.9859	68.5229	10.7908	53.8174	0.6904	0.0528	0.2176	0.0059	21.2817	9.4418	17.7980	10.5750	112.4230	4.3144	0.0030
TARGET-50-PAKYLT	53.0918	116.2320	9.0573	80.3412	9.7843	35.4172	90.7260	15.5402	84.1894	1.7735	0.0365	1.3277	0.0095	13.4386	9.1012	37.8799	19.3731	134.8499	17.7970	0.0103
TARGET-50-PAJMKN	34.1417	77.0184	9.1324	75.2685	23.6483	44.9732	107.9949	10.6011	66.6095	7.9636	0.0662	1.0611	0.0092	18.6218	8.6962	46.0164	24.0694	223.0352	18.2632	0.0166
TARGET-50-PALERC	9.0184	31.0077	6.1220	40.3308	10.4673	50.4081	79.8180	8.6689	54.0126	1.7719	0.0518	0.3443	0.0053	17.6630	9.8677	20.8763	13.4527	123.1748	4.1580	0.0048
TARGET-50-PAJMFU	10.4741	29.6808	6.7510	94.5109	21.2577	47.3461	89.9268	13.3039	65.5999	1.7074	0.0626	0.2944	0.0066	21.4158	8.7923	34.6747	17.8624	171.3528	7.7234	0.0045
TARGET-50-PAKZHF	4.8909	19.1422	5.2264	121.5480	36.3025	55.4543	82.4822	13.8281	69.9015	2.0405	0.0606	0.1026	0.0074	19.6445	10.4507	42.6737	18.7802	159.2397	6.9335	0.0174
TARGET-50-PAJLSP	9.2877	31.6388	9.5897	73.7202	19.4387	68.1068	106.9237	9.7031	54.6114	1.1590	0.0735	0.1902	0.0061	18.7131	8.4133	37.4764	20.6264	143.5058	12.5684	0.0132
TARGET-50-PAJMKI	7.3664	57.4474	5.7736	82.7168	11.7485	62.1696	71.6003	20.3118	76.6285	0.9897	0.0563	1.5614	0.0051	22.7632	9.2338	31.1325	16.3192	134.5342	6.8361	0.0080
TARGET-50-PAKUIT	13.0862	28.4172	8.6386	38.6722	14.6385	84.1134	73.2329	20.6270	81.1037	1.2233	0.0702	0.8982	0.0048	29.0504	11.6321	28.4199	15.0499	146.9189	8.4709	0.0088
TARGET-50-PALDWP	7.1406	28.9163	5.4324	42.1460	11.3624	54.1335	62.3774	10.5508	41.3576	0.6226	0.0459	0.1323	0.0055	17.1428	10.5226	19.5985	12.5405	112.0663	3.9835	0.0049
TARGET-50-PAJMJT	6.2371	36.9471	8.2982	79.6263	16.5247	42.5029	78.6019	15.0749	31.6069	2.0635	0.0638	0.2196	0.0082	19.1588	9.1582	38.5083	19.4193	136.9466	7.1028	0.0046
TARGET-50-PAKWPM	6.4472	32.8961	6.2114	116.0302	20.3583	41.5889	94.4333	14.4077	69.6938	1.9624	0.0657	0.1900	0.0057	17.0208	9.8263	39.2997	18.1424	138.6307	8.4211	0.0241
TARGET-50-PAJLKC	5.8064	34.7116	9.9847	75.9207	18.2047	52.9766	44.7510	11.4650	58.8450	0.6217	0.0501	0.5161	0.0054	21.0705	10.0285	33.6809	15.3601	115.3031	7.2023	0.0121
TARGET-50-PALKRS	7.8963	30.6086	10.3004	99.7117	18.3241	45.8419	100.3352	10.8045	62.1694	1.8984	0.0320	0.0907	0.0134	16.2932	7.4471	62.0322	19.6113	256.9609	6.1631	0.0055
TARGET-50-PAKNRX	45.5699	72.5658	14.2683	142.9515	34.6123	75.7597	199.4346	15.6704	243.1231	4.2630	0.1445	5.5410	0.0066	29.8543	8.4716	43.5426	32.7461	293.0261	46.9062	0.0559
TARGET-50-PAJNTJ	8.8450	51.7178	9.4111	69.4486	27.9787	43.8099	122.3048	12.7889	44.8359	4.6548	0.0849	0.9566	0.0099	17.4660	8.4293	27.4815	24.3528	181.9038	11.8784	0.0096
TARGET-50-PAJNUS	7.7967	37.0889	6.0373	75.1149	15.4467	50.1992	79.0216	14.6079	41.2256	1.6439	0.0577	0.3081	0.0083	19.0247	10.0874	30.3545	17.0258	143.6131	5.5016	0.0052
TARGET-50-PAKNTW	2.0686	5.6065	4.2692	41.5126	11.8966	46.0196	107.9005	12.6758	23.2380	0.4695	0.0326	0.0358	0.0057	13.8639	9.5827	16.6195	10.4187	100.2789	1.8328	0.0034
TARGET-50-PAJPAU	9.1110	32.5638	6.2896	61.4042	28.8923	54.3974	92.2153	12.1072	33.4931	1.7590	0.0781	0.4843	0.0061	25.3397	7.6877	35.9732	21.2098	235.9788	22.9775	0.0317
TARGET-50-PAJMVU	12.9974	44.9710	11.1695	84.7728	29.5020	88.1240	155.3563	17.9096	70.4036	1.4048	0.0896	0.8040	0.0076	30.2307	11.1521	33.8967	22.6789	281.3798	7.4333	0.0149
TARGET-50-PAJNZI	12.0487	30.7328	9.7496	53.3457	26.0584	66.8884	85.9519	11.9512	74.0259	1.1806	0.0283	1.1377	0.0055	27.7486	9.9345	18.6251	12.5647	116.0832	8.4490	0.0082
TARGET-50-PAJPDN	10.8672	28.9730	15.0544	67.3369	12.2160	42.2913	173.1745	12.0218	70.9289	2.8562	0.0369	0.1226	0.0134	16.2801	6.9654	43.8474	17.0771	232.7836	6.4781	0.0044
TARGET-50-PAJNVE	7.2445	31.8320	6.9338	33.2763	11.3296	56.1558	138.8258	8.1186	47.3798	0.6378	0.0326	0.6717	0.0065	23.3703	12.5504	20.2999	12.5947	89.9292	3.8694	0.0145
TARGET-50-PAJMSE	17.4537	49.0277	6.3815	80.7048	21.6129	55.1300	55.2508	11.3225	78.1504	1.4962	0.0547	0.9943	0.0089	21.0105	9.9762	37.2376	19.6984	128.1490	8.3924	0.0175
TARGET-50-PAJNZU	16.1298	70.1006	6.0121	81.8352	12.5820	56.3694	89.6899	16.6219	74.2860	2.1111	0.0731	0.7841	0.0082	20.7393	10.2765	33.4812	19.3320	158.1749	7.5847	0.0099
TARGET-50-PALGLU	4.6466	23.5129	6.1767	56.3811	11.9592	48.9512	147.3398	6.6133	58.8325	0.9471	0.0424	0.1133	0.0066	21.6554	10.6891	31.1849	15.6638	150.7551	4.7585	0.0120
TARGET-50-PAJNZK	21.8094	41.7972	7.2056	60.9823	14.4401	79.9944	87.6384	10.3664	57.1989	1.0173	0.0659	2.3922	0.0065	19.2519	12.4834	27.6200	19.8581	184.5045	12.1489	0.0066
TARGET-50-PAJMUF	5.2682	31.2956	9.3071	50.7533	12.3683	49.4296	96.0957	18.7820	39.3801	1.9135	0.0527	0.1332	0.0083	15.9643	9.6261	28.5488	16.3664	156.4203	3.5052	0.0050
TARGET-50-PAJMRL	8.6073	69.1171	14.2104	79.0986	15.1980	48.4936	261.5019	13.3477	106.3756	0.7203	0.0497	0.3302	0.0075	28.5904	11.2245	39.7836	21.3122	173.7230	6.2797	0.0195
TARGET-50-PAJMXF	11.1853	52.9841	7.7056	65.7678	21.3338	42.6005	51.0681	9.0953	39.0840	2.1600	0.0390	0.4736	0.0063	17.6041	9.6033	30.0584	16.7685	138.1143	10.8929	0.0121
TARGET-50-PAKPDF	15.4888	37.5479	6.1975	53.3203	21.0786	70.1372	76.2585	13.0701	54.8611	1.2988	0.0445	1.6860	0.0072	25.5313	9.3350	30.6852	15.1404	113.6397	9.4481	0.0118
TARGET-50-PAEBXA	16.2858	28.2371	7.8587	84.4555	29.2359	60.5230	107.4447	13.2494	91.8830	1.9775	0.0759	0.4660	0.0071	23.4352	9.2244	34.9313	20.6226	217.3335	10.9230	0.0137
TARGET-50-CAAAAS	20.8327	85.7045	12.8439	88.3574	18.0194	56.9662	99.1082	11.6379	53.4652	4.0077	0.0701	0.9263	0.0083	21.1389	8.3975	40.9642	16.3464	142.1192	26.2098	0.0481
TARGET-50-PAJNLT	7.6683	45.5563	16.1583	49.8896	12.7675	60.9512	156.0283	10.6686	43.2410	1.1231	0.0515	0.2744	0.0082	20.8877	10.9336	32.4827	16.6718	137.8029	6.7554	0.0094
TARGET-50-PAJPHA	10.7602	56.9012	12.0711	47.2109	12.3566	41.8732	147.2781	11.7231	49.4407	3.0724	0.0647	1.6856	0.0109	14.3141	9.5048	33.0231	20.6188	152.3880	11.9550	0.0122
TARGET-50-PAJMMY	15.4161	51.6711	9.3942	42.8142	10.6665	54.1339	75.5417	6.2842	48.2964	0.8492	0.0419	3.3686	0.0088	17.9424	8.7125	30.6227	13.2945	108.7003	5.8938	0.0089
TARGET-50-PAJLTH	7.5794	21.6061	6.4719	83.7108	21.1362	42.8530	74.6263	14.0529	36.0661	1.5101	0.0495	0.3765	0.0084	18.1062	8.4177	45.2460	20.4084	199.1370	12.3096	0.0113
TARGET-50-PAJNCC	11.4145	43.9859	8.2313	95.2824	3.6190	41.9787	53.1669	20.2527	61.2886	1.0528	0.0619	0.2611	0.0057	9.2280	9.6813	29.4789	17.3053	172.6324	7.1436	0.0095
TARGET-50-PAJLUJ	3.2702	17.4109	4.4111	68.0240	17.4889	46.7223	37.4430	11.3794	36.3978	1.6820	0.0409	0.0539	0.0061	16.9365	10.0764	28.4205	13.2121	110.0983	3.2964	0.0052
TARGET-50-PAJNCJ	17.8270	45.5716	6.5399	62.6739	17.6840	57.4724	87.2326	13.8126	92.2782	2.1424	0.0642	0.9133	0.0079	21.3682	10.6623	32.9134	14.9383	153.8029	9.1949	0.0117
TARGET-50-PAKZER	4.4583	25.3696	8.4148	66.1819	14.8539	52.5078	109.6138	13.9478	73.6861	1.1551	0.0777	0.1313	0.0060	20.1081	11.7826	34.0335	19.9111	183.1217	6.0854	0.0272
TARGET-50-PAJMEP	13.0619	29.6857	8.4405	35.4722	11.6643	52.3668	118.6680	7.2476	59.2824	0.9773	0.0551	1.7648	0.0066	17.5347	9.6416	25.4625	13.2563	117.0828	5.1408	0.0163
TARGET-50-PAJMEL	26.5370	65.8776	5.5557	82.5228	15.2383	42.1168	89.0002	13.6311	82.5551	3.5894	0.0506	0.8307	0.0111	14.0607	9.9391	32.8527	17.2130	133.6930	9.0235	0.0124
TARGET-50-PAJNVX	206.0453	395.5915	12.0101	163.9066	21.8988	45.8590	264.8558	30.1177	397.0609	32.4612	0.0924	21.0133	0.0096	16.5023	10.2549	69.3706	35.7023	204.2390	121.7103	0.1017
TARGET-50-PAKXWB	17.5739	55.9903	6.2038	79.8202	16.5794	34.6808	55.7666	12.9208	79.9914	3.3071	0.0845	0.5125	0.0061	14.4463	8.7153	33.2902	16.1788	155.6238	10.8672	0.0280
TARGET-50-CAAAAB	10.1167	44.0745	9.7871	90.7891	19.4780	51.4837	42.5751	10.2655	68.3917	1.5140	0.0701	1.0996	0.0076	18.7422	8.0674	38.2087	14.5828	136.3541	11.6663	0.0175
TARGET-50-PALJIP	10.3860	55.5421	7.1172	80.9779	12.3034	63.8232	45.4509	13.0313	93.4024	0.9036	0.0542	0.2533	0.0052	19.5689	10.0129	31.6106	16.2681	166.1063	9.3654	0.0142
TARGET-50-PAKMUB	15.0477	142.5257	8.3913	186.7090	31.5580	42.7097	116.8310	19.3007	128.3419	10.9979	0.0685	0.3545	0.0138	16.0618	10.1459	60.5377	28.1503	242.1712	37.2983	0.1002
TARGET-50-PALKCW	11.8904	32.5533	6.1652	72.6527	25.9251	45.6834	77.3462	13.1027	88.7922	2.0296	0.0589	0.1758	0.0052	18.9532	9.2326	33.6820	18.1928	174.7274	8.1214	0.0131
TARGET-50-CAAAAQ	3.1241	11.7977	6.8033	42.0355	13.3064	51.7712	81.8072	7.0682	23.1709	0.8190	0.0313	0.0884	0.0074	18.9585	8.7367	23.8627	11.3585	106.0222	4.1843	0.0040
TARGET-50-PALFME	6.1040	25.8573	9.1839	70.4616	19.7404	72.7586	84.3277	16.3955	55.6994	0.6177	0.0595	0.2031	0.0068	26.2388	10.8910	27.0918	16.6402	156.7066	6.7012	0.0071
TARGET-50-PAKNXS	34.0516	77.7337	6.6808	87.2690	13.0303	51.2594	182.9796	8.1074	108.8846	1.4300	0.0545	2.5793	0.0074	18.4144	11.0484	38.8980	25.8808	233.8853	16.3352	0.0208
TARGET-50-PAJNAA	108.1882	278.4854	13.6563	382.8544	45.0572	52.0887	403.1369	45.5892	227.5040	41.1079	0.2498	63.7257	0.0174	20.5034	7.7802	75.5119	56.6299	486.5861	168.7867	0.0411
TARGET-50-PAJPGY	8.0748	80.2665	7.6646	61.2985	12.9133	40.4628	79.0245	13.7911	68.0545	1.3320	0.0573	0.3570	0.0100	18.6406	10.3659	43.3990	18.4060	165.0636	10.6771	0.0200
TARGET-50-PAJNEC	2.1672	9.1512	24.4906	110.3041	15.8507	70.3717	61.1948	21.7147	30.2975	0.9156	0.0290	0.0749	0.0087	23.2053	7.6102	90.7817	20.6374	545.0671	2.7566	0.0032
TARGET-50-PAJNDU	3.5475	7.7625	9.2845	24.0961	7.4052	38.5908	124.6415	8.5993	41.9449	0.6854	0.0147	0.0280	0.0091	13.3137	7.5500	30.4506	12.8431	209.2109	1.5822	0.0015
TARGET-50-PAKGZX	9.9863	38.0896	3.9588	116.2244	33.7118	68.2971	96.1938	14.8110	101.0273	2.0519	0.0613	0.2611	0.0069	17.9814	10.1510	34.2132	15.6434	115.5187	7.4257	0.0420
TARGET-50-PAKFYV	43.8600	46.6692	5.0628	44.7642	10.0581	71.1780	154.1379	6.1526	153.8281	1.6330	0.0508	1.3182	0.0073	25.3530	8.9744	25.7698	13.8426	108.2320	8.3297	0.0037
TARGET-50-PAKGED	9.9285	11.3178	17.0039	83.1312	16.4759	40.6417	98.4784	11.7230	128.2667	0.4251	0.0194	0.1846	0.0312	14.7436	7.8865	64.9350	16.5065	449.1684	3.2760	0.0021
TARGET-50-PAJLTI	10.7628	39.1797	6.4997	59.7297	16.5041	43.6636	143.9083	13.3313	62.8381	1.8610	0.0755	0.9822	0.0085	17.3360	9.4338	39.7401	20.0918	150.6270	7.7587	0.0252
TARGET-50-CAAAAO	6.7906	18.0824	5.6073	64.0263	16.8237	47.6483	79.1559	9.2936	68.1073	1.1455	0.0503	0.1018	0.0065	22.0686	9.5853	30.1455	15.3267	168.6313	4.7872	0.0030
TARGET-50-PAJNCZ	15.9155	42.8204	7.3612	64.0950	12.5382	52.4995	89.4524	12.0804	62.3373	1.7867	0.0570	0.6447	0.0073	17.7161	9.5506	29.5375	15.3355	131.5566	6.6832	0.0084
TARGET-50-PAJMIZ	4.9239	25.2863	7.6505	104.8017	17.1021	56.1327	60.7117	16.6058	40.2379	2.5082	0.0491	0.1227	0.0080	24.5620	7.8320	63.1184	18.6901	178.5750	8.7038	0.0070
TARGET-50-PAJMFY	17.7691	37.3489	7.1168	59.6599	14.8381	57.6643	55.6739	12.1296	75.2775	1.8584	0.0653	0.7957	0.0061	24.0301	9.3629	30.4300	14.8350	163.2277	9.6707	0.0093
TARGET-50-PAKULH	9.4254	51.4719	8.4155	109.7703	18.7940	48.4306	78.2646	15.4238	56.7005	0.6041	0.0632	0.0365	0.0044	20.0319	9.7445	46.7021	16.8977	120.3532	7.7141	0.0139
TARGET-50-PAKVET	98.5312	221.3165	10.9405	106.7708	47.9886	51.1584	162.0539	15.7345	114.0688	14.5546	0.1171	11.8420	0.0090	22.8445	8.6582	51.4061	32.1563	278.6310	92.6497	0.1299
TARGET-50-PAJMEN	8.2342	27.3606	7.6210	47.0800	19.0076	52.6605	58.0305	14.3899	60.4289	1.2842	0.0430	0.2202	0.0087	23.6143	9.7345	27.3441	12.6315	120.0520	4.6137	0.0055
TARGET-50-PALDTE	12.6219	40.3122	7.1082	80.1022	12.5056	56.1414	74.4845	10.6405	58.5051	1.3734	0.0574	0.6234	0.0075	19.1321	9.6199	35.5030	17.4763	133.5049	7.4706	0.0079
TARGET-50-CAAAAH	4.1324	11.8758	13.1839	58.7364	11.2983	43.7932	249.8541	23.3422	54.5831	0.6398	0.0209	0.1802	0.0193	17.3272	6.9721	61.7458	14.0352	344.8107	2.8079	0.0027
TARGET-50-PAJNRH	11.1787	30.4472	8.7925	50.3606	33.6005	56.0573	118.3430	12.3441	82.7033	1.9141	0.0709	0.2457	0.0063	31.7802	9.2308	33.1914	17.1584	265.4177	19.8709	0.0067
TARGET-50-PAJNRL	6.0755	18.8067	7.9492	61.9792	25.9367	69.5208	63.1804	12.7647	43.4829	0.6529	0.0604	0.3075	0.0072	26.7247	9.3152	33.6851	17.5827	143.0511	8.3787	0.0091
TARGET-50-PAJNUP	139.1307	241.1209	13.9673	293.2958	39.8009	44.3170	182.6971	33.3955	230.0167	9.2631	0.1719	9.9562	0.0253	15.1630	8.0338	88.3775	40.7291	547.5435	87.1899	0.0916
TARGET-50-PAJPDC	10.4512	33.5401	4.0967	123.3603	36.9690	65.5367	41.8443	12.0105	74.1732	1.4671	0.1364	0.0640	0.0041	28.8726	7.5878	33.4145	14.8557	105.1087	10.1236	0.0470
TARGET-50-PAJMLI	12.7634	49.9437	7.5050	71.5720	12.4133	46.7823	88.9968	14.5052	67.6832	1.9712	0.0520	0.8573	0.0098	16.2866	10.1514	39.0279	16.4724	129.0934	6.8928	0.0097
TARGET-50-PAJLWT	10.0726	23.3269	7.7836	56.3115	18.6330	38.2930	115.5947	7.7936	76.2035	2.2968	0.0580	0.5753	0.0059	16.7486	10.3205	27.5179	16.5236	182.5953	5.4097	0.0105
TARGET-50-PALEZT	9.4756	37.9472	11.8438	73.5556	17.4495	56.7508	64.6096	13.4306	74.2324	2.1162	0.0581	0.1913	0.0053	25.6085	8.5789	40.2085	14.9937	140.1805	10.7022	0.0111
TARGET-50-PAJLLF	19.5295	59.4664	11.2202	160.2588	21.3531	56.3779	203.3397	29.2955	153.6451	1.5171	0.2395	3.5140	0.0048	27.8666	9.2096	53.4293	31.4662	317.2125	9.8259	0.0177
TARGET-50-PAJLNJ	6.8002	21.9656	5.2150	56.9856	11.6100	44.7627	64.1907	11.2349	39.5304	1.2387	0.0520	0.1223	0.0065	15.9522	10.1415	28.3723	14.7643	142.2600	3.8694	0.0041
TARGET-50-PALFRD	8.7198	24.2889	8.4585	63.9952	20.8606	44.1464	53.1323	10.6442	67.1393	1.2518	0.0393	0.3250	0.0049	16.9804	9.1586	33.4243	17.1037	180.5697	7.8024	0.0073
TARGET-50-PAKKSE	20.0140	60.4943	5.3814	76.5019	13.5646	31.7107	97.8019	16.2601	71.2597	3.4954	0.0556	0.8131	0.0100	10.8677	10.0897	33.0146	18.8980	155.3366	7.4911	0.0117
TARGET-50-PAJLPX	20.0417	59.4835	11.3782	59.6536	15.6567	81.1721	178.2053	17.4200	92.2755	0.9312	0.1032	2.9471	0.0074	28.3025	10.6022	38.6049	23.4245	217.7503	10.5916	0.0238
TARGET-50-PAJMKJ	17.0667	46.4201	9.3276	169.2326	33.2087	59.6644	40.9807	17.4200	93.2965	1.3251	0.0766	0.2781	0.0053	21.1305	7.7995	45.6867	21.9080	165.0953	20.2510	0.0176
TARGET-50-PAKSDG	17.5107	51.9557	6.4849	48.0551	13.8131	60.4932	87.9904	8.8009	80.8043	0.9808	0.0588	0.6849	0.0066	19.7295	11.7438	19.8765	16.3936	149.4844	5.7562	0.0077
TARGET-50-PAKZFK	10.1360	46.3898	6.4087	127.7574	20.7234	54.4975	98.6527	16.9247	95.4302	2.0415	0.0658	0.2737	0.0082	20.8965	9.8525	54.6134	17.2035	172.6552	11.8211	0.0080
TARGET-50-PAKYFC	16.1345	52.3397	7.0737	67.0476	16.0199	51.4129	132.2836	16.5446	78.5726	2.5112	0.0807	0.8888	0.0093	20.2386	9.7114	34.7904	16.2472	131.2054	8.7039	0.0188
TARGET-50-PAJNNR	10.6673	44.5409	6.8769	79.8468	12.9508	48.7279	170.5225	15.8488	101.7447	1.2353	0.0627	1.3931	0.0052	21.5615	10.1083	39.4771	20.1180	182.6633	5.4340	0.0159
TARGET-50-PAJLIP	58.4803	241.5872	20.4695	117.3933	27.5796	63.7745	180.0417	20.5439	187.1098	2.7104	0.1367	7.4877	0.0116	26.8836	12.1776	49.9467	26.0928	277.7016	31.5468	0.0518
TARGET-50-PAJNYT	37.8801	107.1189	8.3575	113.6284	12.5698	25.2768	102.9025	14.5481	122.3842	11.6917	0.1182	1.4532	0.0121	9.7973	8.5572	49.1325	23.7402	164.8278	19.8178	0.0142
TARGET-50-PALGVY	7.5494	15.2793	5.7073	99.7173	23.2263	55.2544	87.1981	14.8949	73.7285	1.3987	0.0534	0.1224	0.0070	19.8846	10.0447	29.6203	17.6953	186.7301	5.3955	0.0039
TARGET-50-PAECJB	4.3294	28.8395	5.7077	41.6974	10.9551	49.1796	67.3852	13.8168	37.9023	2.4162	0.0496	0.1541	0.0076	16.3916	10.3847	24.6372	11.8516	106.1462	4.1113	0.0074
TARGET-50-CAAAAM	759.2279	492.9407	5.5990	193.9807	23.7093	12.0700	38.0723	25.9302	242.5107	13.2282	0.1090	35.5690	0.2051	4.6420	9.2076	53.5159	42.9614	397.7795	458.0639	1.6856
TARGET-50-PAKSCC	6.9745	23.9156	7.1746	42.6271	11.6387	42.7684	44.1291	9.8000	42.1546	1.5074	0.0482	0.1403	0.0060	14.8571	9.0029	21.6591	13.5155	145.2809	3.5475	0.0057
TARGET-50-PAKRZW	34.4922	53.2662	6.3203	73.4987	17.3672	25.7798	40.7617	12.8317	92.1876	5.4671	0.0528	0.8194	0.0102	11.8211	9.5458	41.4083	22.3239	194.1771	19.6698	0.0188
TARGET-50-PAJNSL	12.4468	38.2226	6.2521	49.9057	12.0032	57.1633	82.6069	8.5348	61.4319	1.8541	0.0513	0.7125	0.0065	18.8207	9.4740	22.5760	13.4271	121.7995	5.0783	0.0061
TARGET-50-PAKKNS	26.7048	76.2562	11.3295	114.0063	22.4099	46.8451	187.3769	19.1756	108.3469	2.9404	0.1231	1.0384	0.0086	18.5566	10.4356	57.2655	25.5065	256.5182	16.0002	0.0359
TARGET-50-CAAAAL	19.8019	50.8068	6.7924	87.9708	18.1793	61.5218	112.5294	14.0228	82.7827	1.6897	0.0750	0.9007	0.0082	23.9501	8.8931	31.1083	20.2167	196.1566	10.2717	0.0049
TARGET-50-CAAAAJ	5.2165	21.3987	6.2764	75.0023	10.3390	47.1630	100.3311	11.5420	41.9519	1.2346	0.0577	0.1636	0.0072	17.2546	10.0768	28.4670	14.7970	145.2157	3.8628	0.0033
TARGET-50-PAKNAL	17.2684	66.8249	11.5933	126.5304	20.7672	44.0222	58.8489	16.6780	76.9303	4.1891	0.1241	0.4890	0.0074	17.8376	8.3411	40.3997	22.1439	163.9625	12.2976	0.0137
TARGET-50-PAKXXF	6.6468	22.8505	11.2552	47.6951	15.4777	44.6260	242.2862	15.0032	97.0021	0.6809	0.0350	0.2663	0.0069	16.3003	9.6856	22.9084	14.3645	144.8516	3.6384	0.0064
TARGET-50-PAKECR	21.5370	44.2852	5.4518	146.1443	32.7898	44.3641	84.1339	21.3176	143.4889	1.4466	0.1244	0.3894	0.0095	20.1291	9.7227	44.3052	28.1082	278.6477	20.7493	0.0279
TARGET-50-CAAAAP	44.4365	51.1735	12.1789	99.3069	20.2266	63.9750	104.0593	21.1840	111.0504	2.0448	0.0837	0.8982	0.0049	22.3890	12.5260	41.3536	21.0877	165.7569	15.0682	0.0087
TARGET-50-PAEAFB	9.9965	15.9197	6.3782	62.2219	22.4392	41.3715	172.0186	14.6157	78.4709	0.9918	0.1513	0.5648	0.0073	17.2014	9.2167	23.4453	18.2216	191.1651	5.0076	0.0208
TARGET-50-PAJLKR	10.2875	42.6687	11.9853	88.3033	23.1504	80.2028	76.7909	17.0366	83.0018	0.8536	0.0715	0.3460	0.0053	30.1897	12.8160	45.6360	27.6018	232.7936	14.8726	0.0074
TARGET-50-PAKMSV	2.6676	9.5634	7.8319	36.6460	10.4375	34.7264	86.0059	10.8050	19.0281	0.7078	0.0365	0.0379	0.0056	11.6772	8.3120	24.1665	13.0886	118.9567	2.5256	0.0050
TARGET-50-PALLFB	11.4198	60.1136	9.0101	57.2302	18.6297	80.2020	187.8196	12.5128	115.3358	0.9444	0.0541	0.6527	0.0056	29.2831	10.9358	28.4995	15.8040	182.5925	8.4778	0.0038

Supplementary table 1 continue3

	BMS-345541_1249	AZ960_1250	Talazoparib_1259	XAV939_1268	Trametinib_1372	Dabrafenib_1373	Temozolomide_1375	AZD5438_1401	IAP_5620_1428	AZD2014_1441	AZD1208_1449	AZD1332_1463	Ruxolitinib_1507	Linsitinib_1510	Epirubicin_1511	Cyclophosphamide_1512	Pevonedistat_1529	Sapitinib_1549	Uprosertib_1553	LCL161_1557
TARGET-50-PAJNAV	25.6731	11.5674	166.1675	64.1877	2.7041	129.4378	694.8483	5.8610	272.2140	10.6633	262.4369	53.9980	151.0441	45.4144	0.7974	271.7489	2.3351	41.1622	34.0175	303.6434
TARGET-50-PAJPEW	36.4833	22.3325	32.9936	121.8196	0.9049	89.1698	691.2016	12.3376	209.4769	12.3296	334.2444	71.6832	221.7778	92.2397	0.4355	221.1717	1.8891	62.4042	27.3133	178.2373
TARGET-50-PAJMLZ	53.0869	15.4747	50.7014	117.8932	7.2955	112.5334	357.3506	16.2177	290.1123	13.5669	179.7245	76.4774	149.5222	65.4892	0.4866	216.1877	3.8180	57.5841	33.2875	174.8670
TARGET-50-PAKRVH	28.8000	8.5182	45.5842	86.1768	3.1424	99.3735	499.6367	14.9205	183.5143	20.7089	251.8834	56.5207	110.9623	61.7115	0.4045	193.0934	7.0981	35.0226	30.6088	106.5671
TARGET-50-PADZUB	30.4511	9.4209	27.7235	110.5678	0.8546	68.8187	503.5926	8.9403	221.1997	18.7719	178.6496	55.5015	157.1782	35.2190	0.2733	182.9677	1.7354	97.1847	31.1715	170.4470
TARGET-50-PADXAY	10.0817	10.8996	61.6500	112.9380	6.7163	211.6241	489.5273	8.6473	190.9092	12.9236	387.7625	65.7893	149.0010	118.4547	0.5008	203.3498	1.1071	133.4798	15.8060	118.3898
TARGET-50-PAJPCM	16.4318	9.6806	52.6859	100.7142	2.1647	59.0007	254.2551	9.6835	271.9248	11.0742	186.1865	44.4839	112.3426	26.3284	0.4120	140.3556	2.0052	36.4955	75.6416	255.2988
TARGET-50-PAJPAR	44.5391	14.1045	37.1515	121.0142	5.6847	93.5924	374.3399	12.5308	374.1722	20.0484	208.2121	73.2956	133.7646	63.3977	0.7226	167.1947	2.7303	57.8227	46.0525	250.2932
TARGET-50-PAJNZS	34.9141	11.3856	55.1194	103.2562	2.0661	89.3007	463.9115	23.9726	429.4565	10.7595	286.8640	67.5333	141.6024	58.7953	0.6294	201.0904	3.7325	41.5217	26.7686	173.3792
TARGET-50-PAJMVC	7.9307	6.7233	14.3526	89.3212	2.3069	200.9443	310.6599	4.0844	101.8254	5.1774	291.9926	44.2427	87.4274	26.3199	0.5543	135.8624	0.5959	24.5509	4.3314	43.3790
TARGET-50-PALGAZ	33.8274	9.3845	28.2311	98.7224	1.1247	116.0192	417.9220	14.8592	268.3772	7.0669	158.5724	48.7425	150.1322	44.0320	0.3771	204.5460	3.0612	53.3358	19.8842	183.7551
TARGET-50-CAAAAR	34.7785	6.8566	20.5218	94.6297	1.7305	98.8247	450.3183	10.1141	156.9800	6.9521	144.3280	38.1382	141.6044	41.0321	0.3338	148.0800	3.5966	42.8845	16.8518	148.0638
TARGET-50-PAKGMU	30.8574	8.0424	48.8179	79.6977	1.6381	99.2545	476.2071	11.1727	240.3624	11.1375	269.8142	51.7221	132.7458	61.4682	0.6756	267.8557	1.7286	106.0189	27.1942	209.5168
TARGET-50-PAKRCC	25.5009	10.1100	38.2617	100.7680	3.2190	84.3293	378.6981	13.2305	175.6773	14.8067	115.7983	85.1323	110.0777	31.1295	0.3554	114.3502	3.9212	43.6859	11.0187	114.7418
TARGET-50-PAKFME	32.3872	7.0428	25.7606	90.8278	1.8658	89.3475	512.2586	8.8215	165.5760	10.3540	184.6476	59.3577	130.6445	45.5788	0.4061	176.4894	4.7611	57.8425	18.4102	128.5733
TARGET-50-PAJNNC	34.1642	7.7038	13.7748	84.8617	1.3165	133.5594	408.6153	10.0421	235.9175	6.0891	193.1284	66.2929	141.1520	42.7561	0.2913	230.3501	1.7794	40.8864	22.9961	168.6791
TARGET-50-PAJNJJ	64.4092	13.4396	32.3722	105.1744	1.6676	118.9247	629.2591	21.1839	426.7277	10.5454	348.9320	60.4154	238.7292	69.5755	0.5618	258.3870	1.9839	37.7172	24.2011	243.1802
TARGET-50-PAKJGM	11.2397	3.0945	3.1244	44.8697	0.4085	60.0813	187.5437	3.3958	53.2631	6.5797	185.0507	24.9969	84.9921	43.1289	0.1311	97.9704	0.4789	29.8611	18.1931	68.3724
TARGET-50-PAJNGH	35.7331	5.0000	19.5605	65.2641	1.9571	83.2602	276.4641	14.2074	109.8353	8.7165	119.5069	38.4539	79.0620	38.7055	0.1766	142.2005	3.2754	37.7546	22.5266	84.6714
TARGET-50-CAAAAC	26.3663	10.5220	20.1321	67.6582	3.5240	174.6191	368.8490	12.1328	143.7230	10.9443	141.8540	92.0909	101.6318	37.2654	0.2304	118.2350	1.8149	57.0037	15.7003	86.7145
TARGET-50-PAJNBN	22.2186	5.4073	25.7345	78.2177	1.9705	68.3912	210.0350	6.3055	127.2555	14.0887	132.6169	32.8862	71.8375	21.3455	0.2478	99.2472	2.9625	25.0572	30.7923	110.4663
TARGET-50-PAKYLT	26.9051	11.2007	82.4267	109.4152	12.5883	170.7691	319.5874	9.5033	177.4839	8.2937	210.6400	50.2858	150.9030	26.1937	0.6372	199.6012	3.3333	81.0705	25.1194	185.7119
TARGET-50-PAJMKN	42.4165	7.8464	36.6607	63.1684	1.5752	111.2666	506.3659	9.2272	288.5190	6.8909	311.3050	50.8601	143.0349	108.3950	0.7027	250.8762	4.3227	15.8854	15.4935	147.8938
TARGET-50-PALERC	23.5037	5.5205	18.3404	86.2106	1.4371	47.3614	306.3228	7.2326	164.4509	6.8708	184.9365	40.1790	106.7387	43.5289	0.2718	133.8817	1.5131	20.6463	16.9046	99.4017
TARGET-50-PAJMFU	28.2633	7.9978	29.7078	79.4789	0.7610	81.3722	302.9871	8.5959	137.3245	12.0593	227.5087	35.8424	98.8465	54.8364	0.3260	151.0139	4.1397	44.1537	34.4061	128.8091
TARGET-50-PAKZHF	27.1371	7.2727	11.8116	63.0807	0.6058	81.0708	436.9079	7.7205	162.9399	7.9746	208.4825	36.3409	133.8834	36.7517	0.2157	198.3521	2.4344	39.7024	22.2445	146.9290
TARGET-50-PAJLSP	26.9047	11.5722	11.6170	84.3319	4.7366	131.3435	322.3039	13.7836	133.9997	17.2559	160.9018	81.8300	107.3688	34.4671	0.1671	148.9596	1.1065	35.7768	25.3822	85.6853
TARGET-50-PAJMKI	26.7889	7.3384	19.1118	90.4914	1.5632	74.2968	387.8597	15.0203	113.3753	11.0744	165.8455	42.4128	118.0685	35.3449	0.4734	144.4791	2.0799	74.3622	17.8784	90.7481
TARGET-50-PAKUIT	35.5880	10.8263	27.6508	116.5022	3.2503	108.1691	376.2247	7.8418	392.2947	9.1483	168.0571	51.7856	134.5046	28.5326	0.3644	177.3781	1.4628	97.3626	32.0787	190.4013
TARGET-50-PALDWP	14.8167	4.2226	14.7208	69.1122	1.6201	79.5746	257.1658	6.5048	167.5056	7.4348	117.0305	27.0581	90.3042	24.6431	0.1870	115.4413	1.2281	43.6249	22.2768	117.8015
TARGET-50-PAJMJT	21.7196	6.9619	6.8856	76.6397	1.9127	125.1910	436.2257	9.4368	186.7645	4.9425	151.9699	48.1025	149.5265	41.0754	0.1578	149.6794	0.8811	54.7256	23.6578	159.1482
TARGET-50-PAKWPM	29.8522	4.7672	17.8655	60.9445	1.0304	60.8279	450.2386	8.9546	205.5274	8.1385	253.0833	43.4333	121.7341	38.0482	0.1840	202.6216	1.9178	34.0214	22.2429	146.0096
TARGET-50-PAJLKC	22.2466	8.6990	16.2925	76.9665	2.7218	86.0542	212.4660	10.1604	85.3013	12.9859	110.7576	67.3994	91.3775	46.0835	0.1057	122.3478	1.4741	38.4112	12.4508	78.7661
TARGET-50-PALKRS	18.9613	7.4975	9.9502	67.8917	1.4854	168.9979	579.9713	4.3377	111.3659	3.9801	246.0718	78.8187	168.6197	70.6540	0.3629	202.9394	0.5845	85.5825	14.9642	141.0400
TARGET-50-PAKNRX	36.1128	27.6529	64.6816	116.4733	1.8616	308.0699	774.5337	31.8413	328.4592	14.0427	335.9769	64.0415	170.1852	100.3128	0.6809	259.7641	8.4930	28.5548	78.8816	279.9100
TARGET-50-PAJNTJ	37.7962	7.9538	14.9590	92.4446	2.8159	85.3471	385.5921	12.6032	179.7372	6.5068	191.5349	66.4041	142.6831	60.2863	0.2062	178.0322	1.7205	24.4574	26.8209	102.7980
TARGET-50-PAJNUS	28.9087	5.4607	10.1760	77.7149	2.1901	100.5409	426.8401	7.6952	170.2780	5.4259	146.0683	34.6097	138.1060	34.2025	0.2390	148.1433	1.3812	48.4268	16.9745	161.5230
TARGET-50-PAKNTW	8.9881	3.1548	2.8276	66.8280	0.5343	31.2067	248.1704	2.9432	40.2428	7.1063	139.6025	32.3155	73.4670	16.6743	0.1553	83.9574	0.3677	90.6487	17.2391	68.0519
TARGET-50-PAJPAU	28.5044	4.1419	15.2441	49.8017	1.2098	70.8071	287.4784	12.1362	98.8067	9.9169	190.9293	49.3158	77.6533	32.9448	0.3090	194.6657	1.8267	26.0037	25.8837	79.3180
TARGET-50-PAJMVU	43.1742	8.3101	15.0419	88.4879	1.3412	150.3278	568.9385	11.2071	243.4456	11.3259	311.7646	53.1144	177.2395	88.7032	0.3662	205.9606	3.2749	77.0183	20.0011	181.4581
TARGET-50-PAJNZI	25.4897	6.0212	36.5348	76.7225	4.0563	70.3089	218.1088	6.5377	152.8226	12.6172	127.1712	53.2450	71.8075	26.6401	0.5018	98.5620	5.2691	39.6344	16.2783	158.7603
TARGET-50-PAJPDN	19.7854	11.0837	8.8412	76.1410	1.2397	112.7094	460.0388	4.6908	136.7392	4.7634	260.7692	73.6529	139.0791	83.0816	0.3158	159.2491	0.5494	106.8897	15.4874	104.9989
TARGET-50-PAJNVE	17.4796	3.6723	10.8625	39.2615	4.2990	60.5235	157.1092	4.3650	75.4321	12.6597	108.7503	39.8302	57.2662	24.1292	0.3064	102.6378	0.4956	49.1784	18.9844	84.5065
TARGET-50-PAJMSE	38.8545	5.2967	37.4936	67.3997	1.6817	68.7950	318.3362	9.6161	135.3964	6.4748	179.1584	28.8582	103.9636	44.0189	0.2202	168.9133	4.7220	31.8608	15.2990	115.8427
TARGET-50-PAJNZU	51.1133	6.2966	26.1271	91.4172	2.0504	89.9717	429.4608	10.4411	213.5867	8.2722	163.0429	37.5184	150.1225	49.1996	0.3860	154.7283	5.8564	30.7530	25.8287	172.5357
TARGET-50-PALGLU	16.5248	6.2082	6.8162	59.0524	1.0901	83.7349	404.4786	4.8464	183.4535	4.7029	170.9670	59.5298	111.8294	28.0295	0.2060	164.3630	0.4597	39.0164	14.3005	216.5719
TARGET-50-PAJNZK	33.6138	7.0420	22.5714	92.4976	1.6810	81.9929	411.6055	9.1482	204.7458	6.3711	119.6860	35.2483	112.2945	35.1374	0.3028	132.3434	2.7941	52.6047	10.1998	129.7857
TARGET-50-PAJMUF	21.7850	3.9747	9.7517	62.8112	1.0542	64.8264	361.0820	5.7941	100.0139	5.2503	199.6242	32.6292	102.3814	41.2178	0.2605	125.6989	0.4703	64.4010	16.4667	94.4673
TARGET-50-PAJMRL	32.4946	7.2207	35.9157	130.5148	7.1601	59.7920	313.3804	13.0535	96.9783	22.7353	186.1184	67.0883	140.0515	58.5176	0.3059	185.3924	2.4128	102.2570	37.5958	118.1051
TARGET-50-PAJMXF	17.2733	4.6290	14.3311	46.4507	2.8239	99.1633	277.8414	8.3216	77.5273	7.1572	174.6058	45.4458	92.7181	42.6252	0.3083	145.1286	1.1923	13.3826	11.8045	71.8197
TARGET-50-PAKPDF	25.3975	7.3590	44.9594	61.5493	4.3754	74.4419	372.4018	7.7490	98.8931	8.7435	130.6066	21.7860	62.5578	32.5422	0.4813	174.1381	4.7902	36.3326	19.4686	85.2212
TARGET-50-PAEBXA	30.3164	9.2302	23.8589	80.9527	1.2245	94.7377	442.2250	11.7671	186.0292	6.9377	277.7544	40.2554	139.7973	53.0979	0.3257	212.6765	1.8685	44.8192	16.4781	154.4758
TARGET-50-CAAAAS	40.6950	6.9958	15.8972	73.6378	3.4902	171.1224	342.5007	18.7033	162.4014	7.0764	144.0647	74.5825	123.7814	28.5771	0.4077	133.3601	3.8793	32.0115	18.2249	122.3578
TARGET-50-PAJNLT	26.3938	7.0609	8.5058	82.0048	3.6526	82.1781	318.7813	12.5898	75.7062	12.3335	201.5259	82.1999	124.5224	55.9768	0.3414	123.4010	1.0582	47.1095	18.8428	80.9588
TARGET-50-PAJPHA	36.3434	5.8648	13.7083	76.6424	1.5047	146.0581	535.6584	9.6366	105.4618	4.7935	206.0578	47.7705	141.4027	54.5206	0.4421	167.6395	1.4514	44.4554	9.4408	110.0237
TARGET-50-PAJMMY	21.1262	3.3582	16.3046	64.1647	3.7128	96.1098	331.9947	7.5466	43.0650	4.5003	115.4697	50.0311	76.5495	31.2006	0.2461	118.3467	2.4472	24.3311	6.5044	52.0929
TARGET-50-PAJLTH	22.8096	5.9150	15.9772	62.8199	0.6248	76.4046	520.6994	9.0083	164.3028	5.4455	167.2779	40.9893	112.2922	23.2136	0.2523	165.9231	1.0802	49.6992	9.9096	105.4770
TARGET-50-PAJNCC	22.3487	6.0039	13.2783	79.6161	0.4840	86.6388	303.8137	9.7659	265.0790	3.1127	172.9863	18.8962	169.7147	49.9065	0.2920	175.1206	0.8015	51.5078	11.5718	294.6840
TARGET-50-PAJLUJ	20.9247	3.0179	4.2763	50.0705	0.5879	61.3565	211.7343	3.5822	103.1736	2.9576	183.8297	15.4445	121.9031	32.7330	0.1519	129.2650	0.7696	50.0051	13.8775	147.5203
TARGET-50-PAJNCJ	22.7652	8.4847	41.2864	90.9700	2.9926	108.7137	510.1373	13.4988	187.3751	9.1084	123.9145	42.8288	99.5411	32.2791	0.4501	177.9045	3.2264	53.4689	22.1505	120.8810
TARGET-50-PAKZER	26.4626	6.5931	7.7389	69.1312	1.1907	99.6776	376.6235	9.6772	178.3751	3.6750	228.4523	40.9423	132.2022	34.6570	0.2220	200.6589	0.4653	72.4404	9.1932	158.7239
TARGET-50-PAJMEP	24.0155	5.1810	16.4124	77.6657	2.9713	75.3311	286.6605	10.8677	79.6888	8.8704	122.9592	56.0076	89.5003	29.5701	0.2523	124.7317	1.5169	29.5380	11.9962	58.7415
TARGET-50-PAJMEL	52.4798	7.7714	35.1410	103.8436	1.3916	100.8251	434.3593	9.7243	202.7948	5.2350	172.2928	31.0801	174.9688	32.6554	0.3854	164.0136	6.2887	57.4998	14.9383	176.6367
TARGET-50-PAJNVX	117.5723	18.9065	244.5006	124.3913	5.6745	259.9922	965.9300	22.6036	361.4869	7.2478	316.8457	54.8664	208.2535	47.7721	3.1843	341.6253	24.3749	168.3533	22.6194	344.6971
TARGET-50-PAKXWB	36.2820	4.3077	26.9778	45.4591	0.8942	89.9308	288.4836	8.1485	79.8841	6.1766	221.6778	29.5297	97.7189	36.0798	0.6922	153.7329	2.0041	38.9885	11.4629	107.8935
TARGET-50-CAAAAB	31.6872	7.9866	16.3071	61.4973	2.7817	99.2053	309.7970	10.7568	111.4525	11.7257	136.5891	63.6631	82.1698	29.8317	0.2621	124.6735	3.2323	29.7746	15.4445	99.8271
TARGET-50-PALJIP	11.8262	8.1145	23.5954	77.0196	0.9847	88.5359	364.2944	8.1981	107.8816	5.5541	134.1824	45.0510	114.4658	28.9346	0.3707	166.7581	0.9551	50.8899	8.0667	115.9247
TARGET-50-PAKMUB	87.0530	7.4518	26.7744	64.7558	1.3545	196.9205	525.0744	10.4152	212.6858	3.4242	259.2953	30.4353	202.6408	78.7329	0.7122	388.0449	3.1408	90.7451	14.5529	270.5824
TARGET-50-PALKCW	24.8859	6.0115	29.7301	69.6166	0.6122	79.1255	336.5316	7.8256	156.5922	7.2845	238.7780	27.1647	111.3018	52.0754	0.2361	188.7210	1.5221	37.3444	20.7937	128.8646
TARGET-50-CAAAAQ	14.8076	3.3119	4.4170	56.9586	2.1521	68.9813	275.0422	4.9832	72.8921	6.4972	123.5915	40.7555	74.8306	20.0806	0.1476	88.1650	0.5808	29.5292	19.7851	83.3556
TARGET-50-PALFME	23.7130	6.9769	22.3896	87.7319	1.7046	101.8275	329.2124	9.4397	164.2058	12.6527	155.8798	37.9848	111.2099	38.1344	0.2000	125.0495	1.5961	80.1884	26.4522	147.2357
TARGET-50-PAKNXS	25.2978	12.6338	51.7587	93.5648	4.5485	88.9731	582.8287	10.5489	289.1258	17.5087	275.4918	47.1264	133.0312	65.0560	0.5690	304.1057	4.7041	49.9917	35.8924	200.8604
TARGET-50-PAJNAA	256.2482	32.6594	159.6252	129.6490	3.9511	230.6877	703.4809	57.6682	496.3546	25.7199	580.5539	109.0794	335.0798	146.7041	3.4285	647.5041	152.3392	85.9322	61.1851	346.8339
TARGET-50-PAJPGY	23.6884	4.0514	12.7330	55.4845	2.1505	130.8112	413.7746	7.9311	122.1267	5.4802	209.0677	59.9149	117.9576	27.0712	0.2827	174.8129	0.6529	51.3961	13.8747	145.3545
TARGET-50-PAJNEC	12.5808	6.7866	2.4059	50.6246	0.7374	223.7611	710.7394	5.0628	97.2066	4.4057	532.5746	185.4437	176.3987	92.9861	0.1397	201.1963	0.3338	84.8609	10.6730	52.0927
TARGET-50-PAJNDU	6.2596	5.1974	2.8819	57.5585	0.9145	60.4273	342.7362	1.8807	37.9649	2.0578	197.9398	53.7000	115.8077	39.5362	0.1302	112.5567	0.0845	42.5219	6.5924	60.5806
TARGET-50-PAKGZX	92.7670	2.7729	31.5422	69.1000	0.2287	94.6480	317.0770	6.8314	226.1317	4.6061	170.2855	8.5895	138.4279	48.9628	0.2772	184.4764	16.0571	46.2662	22.4391	186.6947
TARGET-50-PAKFYV	22.4123	8.0780	90.1780	74.1919	2.9816	109.9870	278.6719	8.0474	194.8028	30.1815	124.7912	26.4688	61.7572	25.5752	1.0180	129.6330	4.8372	20.0373	43.6624	135.5831
TARGET-50-PAKGED	7.5225	29.8400	5.3543	116.4552	2.9882	139.8902	784.9310	2.8784	127.1094	1.4604	243.2410	364.9062	334.0081	46.5921	0.1668	180.2167	0.1312	126.5916	8.1069	168.6338
TARGET-50-PAJLTI	57.3440	6.4181	16.7590	89.4638	2.4876	107.4745	402.4391	13.8189	250.3896	6.8094	195.3405	48.3612	130.2483	43.7288	0.2222	187.5148	3.0269	54.7414	17.6930	129.3747
TARGET-50-CAAAAO	18.5532	7.8657	17.4121	79.8202	0.5636	51.1846	281.5599	5.9082	128.6639	8.5728	237.6825	38.8380	107.5795	44.4984	0.2596	150.1434	0.6935	40.1516	23.1170	103.7078
TARGET-50-PAJNCZ	32.6535	6.3483	26.0256	83.9450	1.5240	111.9636	324.0492	8.6775	157.3746	7.8069	171.3128	30.9183	110.1631	44.1408	0.3246	151.7215	3.4562	37.5749	17.6198	130.7229
TARGET-50-PAJMIZ	20.0871	4.0706	14.6912	53.0018	0.9918	99.5425	362.0511	9.0240	147.0965	8.8525	283.9715	29.7053	87.5267	57.4308	0.2242	180.2445	1.0563	58.1432	25.4750	106.3808
TARGET-50-PAJMFY	26.5431	5.5302	37.9939	78.5283	2.1259	80.0655	338.5036	10.7436	178.0448	13.7467	192.8684	33.6682	103.3940	45.4474	0.3200	154.5264	2.7899	29.8257	21.7983	105.4816
TARGET-50-PAKULH	20.0254	5.6984	23.4775	54.4048	0.9138	86.0800	190.5945	6.4674	175.6618	4.9299	127.1104	37.3750	107.9197	35.9239	0.1215	131.3586	0.8887	74.8521	20.0322	212.4885
TARGET-50-PAKVET	89.1339	10.8899	95.3037	77.0479	3.5837	156.6567	720.1401	22.8660	344.5904	11.7295	305.2933	71.8090	153.7720	129.1641	1.5987	393.2384	39.2904	22.4664	25.6050	125.9343
TARGET-50-PAJMEN	17.8303	6.8350	25.8026	71.4559	2.6849	80.2613	289.5735	6.8200	149.3035	6.0704	220.4678	43.3892	124.5907	38.5425	0.3109	159.3532	0.9657	44.9263	19.5083	111.7700
TARGET-50-PALDTE	34.4876	6.9073	27.5126	73.7012	1.3516	92.0496	328.8198	10.6408	170.2304	14.9786	198.2745	49.5006	92.3269	45.4091	0.2953	145.7038	5.0905	41.9478	25.7969	87.9143
TARGET-50-CAAAAH	6.9826	7.5477	7.6703	85.0421	1.8127	92.1468	510.6756	3.7392	112.2727	5.1905	288.7581	82.3284	126.8637	39.7367	0.2783	134.6244	0.1499	207.7305	16.6457	87.4435
TARGET-50-PAJNRH	27.5294	6.1904	44.2419	53.5848	1.0510	85.0314	333.7116	7.4365	116.9729	12.5577	217.2857	37.3222	94.5367	52.1920	0.5961	207.3328	1.2015	28.9571	26.8244	114.9098
TARGET-50-PAJNRL	32.6235	5.2682	17.0738	69.9126	1.2671	89.0313	278.6600	6.0112	61.8175	11.3156	132.1720	31.2902	91.6951	34.9138	0.2726	124.9103	1.5756	55.5017	24.0037	90.0421
TARGET-50-PAJNUP	166.0200	29.4355	132.0927	213.0792	2.7838	259.5490	343.2075	21.8779	381.3633	9.2721	518.1259	79.8589	386.5193	74.4114	2.5636	485.8134	19.3832	240.3794	34.0392	316.7536
TARGET-50-PAJPDC	50.0011	5.5445	16.2276	48.5109	0.3054	100.9704	325.6372	7.5777	196.4400	8.2120	148.5097	42.8735	107.0455	27.9590	0.3640	119.9938	2.4530	38.4346	52.5935	195.2256
TARGET-50-PAJMLI	29.3252	6.0929	25.4540	77.9233	3.8565	97.9055	377.2202	11.0013	153.4459	6.6649	169.2972	36.8005	101.1484	33.1191	0.3068	174.9470	3.2192	56.8636	13.1284	115.7551
TARGET-50-PAJLWT	29.1254	7.4312	26.5604	63.3130	0.7300	71.9130	449.1607	6.5070	166.9120	8.4287	250.3937	38.5295	127.2074	59.2958	0.3160	146.6417	1.1892	21.3828	18.7966	168.2478
TARGET-50-PALEZT	27.1060	6.0366	33.6984	59.1167	2.5998	85.6245	253.1777	7.6394	86.4676	10.0379	216.9322	52.2380	80.0256	109.3483	0.3737	153.0712	1.2221	63.4380	24.5772	83.5889
TARGET-50-PAJLLF	131.9733	15.7358	53.6763	204.9823	1.4211	129.4312	507.4066	17.4007	389.2895	28.5781	271.7454	97.7318	219.8637	58.3633	0.3524	226.1146	4.7805	243.4788	66.9402	261.0170
TARGET-50-PAJLNJ	18.6826	5.0421	11.6135	75.0535	0.5037	58.3151	299.1572	6.2547	116.2143	7.0030	191.9572	33.5576	104.2775	41.0829	0.2202	140.4392	0.9363	48.1982	16.7592	107.0936
TARGET-50-PALFRD	16.1648	5.8152	20.4380	66.6210	1.1232	65.1259	285.5697	5.5687	40.5722	5.8089	182.6765	30.0563	98.9881	54.2502	0.3223	165.3532	0.8331	32.9613	7.5025	72.0408
TARGET-50-PAKKSE	48.0539	6.3503	31.9563	92.3917	1.1801	97.8791	420.4329	8.0221	205.4986	5.2977	215.9103	24.0585	170.8305	36.3942	0.3776	190.9717	3.8382	60.5797	16.3733	173.3139
TARGET-50-PAJLPX	38.2359	6.9427	27.0933	92.9083	1.7177	168.9452	660.3414	10.8341	300.6960	5.2205	200.0785	63.9669	141.3946	39.5253	0.6359	224.0768	1.8533	110.5812	21.8378	212.2257
TARGET-50-PAJMKJ	33.3440	11.4045	22.0861	90.3853	1.1878	147.1205	434.9919	8.7346	177.8393	3.4882	176.4688	52.9952	143.6707	49.6404	0.2770	201.6157	2.1428	63.2318	15.4300	214.3422
TARGET-50-PAKSDG	28.0937	5.6112	27.7883	67.3866	2.3628	94.8491	400.6981	9.5537	185.1180	8.6074	158.3917	33.5960	115.9512	33.6467	0.3830	141.4353	2.0082	33.9388	17.4703	160.4134
TARGET-50-PAKZFK	25.3241	8.0327	34.8709	90.9307	2.1294	127.4671	399.1957	11.9817	135.8715	8.3878	225.1354	28.5500	113.8216	42.2533	0.2528	219.4145	1.9525	59.4664	25.9318	159.2575
TARGET-50-PAKYFC	53.5635	7.9069	24.0410	101.2343	1.5834	92.7696	337.1640	12.4107	184.4677	7.0211	175.4561	40.9608	126.7594	35.9787	0.3971	169.2115	3.6557	72.5035	23.2297	153.8086
TARGET-50-PAJNNR	46.6241	10.4225	34.4341	97.7586	1.0289	93.9468	447.4948	12.9698	209.8569	25.1100	239.7415	41.4428	166.4372	41.8677	0.3870	215.2682	2.4670	73.1190	30.7280	148.5262
TARGET-50-PAJLIP	83.5619	27.8212	105.1581	110.8295	7.9290	211.5594	464.0990	43.7508	414.8245	11.6894	266.1253	144.0164	161.8673	129.2301	0.8518	285.5028	15.9692	75.5263	19.3124	318.4055
TARGET-50-PAJNYT	61.2642	12.5750	41.3351	116.0452	2.1876	152.4034	520.3912	14.2737	329.7736	7.8382	155.4646	38.8301	150.1688	42.4001	0.5213	203.8889	7.3746	109.1250	18.7266	226.4139
TARGET-50-PALGVY	15.2872	8.8713	15.7390	97.2180	0.7384	67.3870	519.8953	6.2853	137.1759	6.4828	207.2054	39.9678	143.2051	36.2107	0.2342	190.0663	1.0061	64.8219	19.8560	142.9123
TARGET-50-PAECJB	23.7951	3.6391	8.5138	63.3579	1.9450	63.3215	321.2333	5.9637	127.2383	4.3478	125.3038	28.1665	92.1535	23.6216	0.2004	117.6808	1.1080	40.7689	14.6007	98.7744
TARGET-50-CAAAAM	43.0042	48.5778	458.8181	214.2236	8.5978	360.6579	395.6047	11.8060	381.8151	3.4198	327.2806	114.4292	432.0692	11.4615	6.0673	677.6452	14.2232	192.1166	10.4454	342.4430
TARGET-50-PAKSCC	17.9601	3.8954	14.5555	54.0107	1.1332	55.2644	334.7646	3.8861	147.4707	4.5364	201.8526	31.9099	107.7304	37.7831	0.2501	111.9111	0.5717	35.5695	14.8964	127.6571
TARGET-50-PAKRZW	30.1860	10.8951	81.8393	95.2680	1.4409	132.3272	455.9502	6.5934	201.2969	4.7328	258.9616	49.7818	161.9048	36.9856	0.8307	242.9312	3.1668	38.2907	11.1220	204.7082
TARGET-50-PAJNSL	25.5019	5.9142	25.4999	83.1715	2.2641	48.7816	379.1982	8.9039	183.3522	8.1623	157.7773	47.1930	102.8383	31.8966	0.3049	144.2359	2.4664	13.9537	14.6194	97.9159
TARGET-50-PAKKNS	38.6746	9.2215	45.9433	98.2583	1.0858	122.8186	617.2331	16.6086	293.9717	7.4086	290.4559	57.2954	174.5841	73.6411	0.5872	265.4657	3.1381	51.3230	23.1987	198.1860
TARGET-50-CAAAAL	38.1761	9.7096	41.7729	102.2133	1.6151	106.3795	401.1408	15.1342	203.4889	14.3794	238.3219	44.6770	127.1558	47.8971	0.3955	170.5356	9.7502	42.5497	31.8248	140.4606
TARGET-50-CAAAAJ	25.4263	6.3560	9.1459	103.4170	0.7450	61.1590	376.3117	7.2761	132.1327	10.5678	200.6532	38.0767	137.6960	45.7778	0.1875	123.3302	1.4724	52.4014	24.4323	119.6108
TARGET-50-PAKNAL	48.5416	9.7352	19.8866	82.0877	2.5012	215.6398	592.6112	13.6296	345.3051	4.2785	238.7352	52.0449	183.9805	45.1649	0.3711	225.9813	4.0289	36.1954	20.1683	228.4830
TARGET-50-PAKXXF	11.1104	5.9984	25.3546	83.2636	2.3753	31.0063	310.9462	5.4483	126.4286	8.5040	214.6997	65.1335	119.5426	33.5251	0.4495	137.5499	0.9173	118.4062	32.3621	116.4936
TARGET-50-PAKECR	42.5958	16.6284	57.6301	98.3150	1.0839	118.2589	585.6174	8.5307	417.2123	4.4391	261.0896	58.0466	188.9707	22.6832	0.5616	325.6667	1.1593	117.4157	23.0859	395.3867
TARGET-50-CAAAAP	30.7925	6.7771	58.2859	93.4871	2.7207	110.0549	281.0538	9.7502	482.5890	8.9692	156.5647	31.7300	129.1150	57.5648	0.3108	197.8271	2.6971	93.8210	37.7002	209.2982
TARGET-50-PAEAFB	49.6255	7.3872	17.0619	96.2939	1.2287	77.4521	393.6794	11.3333	232.5750	5.8110	235.5360	46.1952	166.3943	39.8906	0.1631	163.4991	1.4225	75.1614	25.2736	159.8842
TARGET-50-PAJLKR	35.2063	8.9427	28.6066	94.5048	0.7295	132.0566	532.4004	13.0588	270.7710	12.8790	189.2921	53.2681	175.2625	56.6428	0.2960	195.6232	2.2693	78.2700	16.0842	187.6149
TARGET-50-PAKMSV	10.6586	2.2953	3.4899	48.7885	0.8581	48.4722	189.0497	3.1589	65.8187	5.2965	178.6095	25.8401	77.6495	34.2109	0.1459	103.4087	0.3232	58.5228	17.0992	65.9570
TARGET-50-PALLFB	29.3270	10.0837	37.3138	106.2366	2.9799	104.4964	293.1779	11.8568	241.2847	13.4948	166.4919	59.1499	127.0308	53.8451	0.4197	184.8423	1.5908	48.7176	24.4405	194.3908

Supplementary table 1 continue4

	Lapatinib_1558	Luminespib_1559	Alpelisib_1560	Taselisib_1561	EPZ5676_1563	SCH772984_1564	IWP-2_1576	Leflunomide_1578	Entinostat_1593	OSI-027_1594	LGK974_1598	VE-822_1613	WZ4003_1614	CZC24832_1615	AZD5582_1617	GSK2606414_1618	PFI3_1620	PCI-34051_1621	Wnt-C59_1622	I-BET-762_1624
TARGET-50-PAJNAV	9.6843	0.6738	49.9253	15.1606	352.2391	9.2853	14.5790	130.2632	6.5637	62.9203	101.6184	41.2712	58.5572	209.2089	10.8707	93.5582	188.2495	121.3127	75.7867	25.0456
TARGET-50-PAJPEW	23.0628	0.1265	68.7198	13.6180	294.3400	11.2028	25.7057	154.4198	13.6306	140.5469	105.9117	37.1960	37.1649	191.0964	7.1136	56.0351	244.3850	131.5095	71.0077	19.4753
TARGET-50-PAJMLZ	21.8226	0.2130	48.6077	13.5400	296.6656	15.4554	18.7236	181.8823	13.9759	214.7783	54.7202	57.4294	117.4631	197.4180	22.5022	65.5666	219.3970	138.4313	97.1833	65.6393
TARGET-50-PAKRVH	32.4969	0.1290	51.1963	10.3729	255.6895	13.5474	17.3993	174.4120	12.3544	187.5992	72.5750	35.3275	43.9052	173.9215	4.8298	46.4124	212.1347	83.7338	75.7351	30.0865
TARGET-50-PADZUB	36.0293	0.1633	49.1347	12.3980	323.3167	11.3626	20.1802	141.5864	8.6041	142.3990	48.5016	38.1708	36.8614	190.7997	13.7147	56.9197	257.3717	104.7832	65.7358	20.6871
TARGET-50-PADXAY	60.6225	0.2761	147.1263	29.9333	609.6790	18.0846	14.8308	113.2130	14.1507	159.3546	103.3968	37.7817	72.7340	134.3698	2.9014	62.3126	201.8154	87.8066	46.8191	18.0367
TARGET-50-PAJPCM	13.0706	0.1593	38.3563	9.2214	189.2858	11.5013	12.4986	136.3769	13.9422	278.2384	29.8478	26.7450	61.6456	154.7321	19.0021	39.1784	184.9176	108.5181	51.4915	24.6895
TARGET-50-PAJPAR	13.8610	0.4299	55.9017	17.7019	343.7996	15.4696	16.2245	188.7435	14.0573	168.4264	34.5848	39.0528	86.8433	230.2802	72.3569	44.5448	201.8487	177.2727	60.0386	55.9715
TARGET-50-PAJNZS	27.4285	0.2677	54.1223	16.7166	299.8547	14.1358	18.6783	238.5295	11.6924	209.5264	41.9892	50.2016	55.1947	184.4512	17.3261	45.2927	205.5656	142.8996	71.7286	39.2462
TARGET-50-PAJMVC	13.2727	0.0284	28.4888	3.2601	179.1377	11.7443	16.4184	139.0571	6.0443	102.9608	77.3421	8.4004	65.8698	103.5236	2.0100	52.0087	126.0124	59.6526	47.8011	20.5134
TARGET-50-PALGAZ	21.9019	0.0907	34.1839	6.2436	268.1102	14.1352	16.6439	215.3412	13.2367	122.3013	53.1511	29.4666	45.3604	151.7001	28.1643	48.8840	206.8973	118.9545	85.3860	28.0929
TARGET-50-CAAAAR	15.1748	0.1047	24.4658	3.5845	185.0405	13.1197	15.4671	206.2569	10.3398	194.2997	59.6925	38.7588	38.3672	140.4822	13.9813	32.1952	179.2809	86.1676	84.0589	23.7612
TARGET-50-PAKGMU	47.7872	0.1031	49.4320	15.9093	355.1680	14.4732	21.5029	151.7728	10.0768	95.6921	68.5417	38.4948	71.8438	200.2813	13.1977	64.4527	197.9876	109.6281	104.4687	38.0381
TARGET-50-PAKRCC	14.4416	0.0754	22.2784	6.7362	189.9580	15.5895	11.4475	139.5197	6.2756	82.0363	36.8063	17.8960	39.1790	141.9609	26.0415	41.3875	164.6662	76.9409	54.9236	23.8929
TARGET-50-PAKFME	31.3472	0.1270	40.6778	10.2650	222.6826	13.8954	17.0167	139.6691	8.5126	170.7567	56.2121	27.8799	40.4575	155.5588	8.2498	38.4003	187.8067	78.6170	93.6487	26.2928
TARGET-50-PAJNNC	13.6979	0.1476	25.9724	6.0114	325.4046	13.1680	18.7074	178.0000	9.2833	192.9218	51.4663	30.3968	51.4546	216.2186	17.4571	80.6947	238.8339	127.2198	101.2540	31.5734
TARGET-50-PAJNJJ	24.5486	0.1576	31.8189	8.8825	451.0400	12.8561	26.8372	225.7446	16.5924	244.7354	74.0081	60.5872	79.2633	273.1592	48.9766	54.9459	283.3117	135.4778	117.4970	35.5955
TARGET-50-PAKJGM	13.5828	0.0351	43.1939	13.8773	171.9213	12.3598	12.9333	72.4826	7.1402	33.0318	55.7667	13.6622	30.5610	93.4912	0.8738	25.2639	132.8521	37.8352	29.9129	9.4638
TARGET-50-PAJNGH	14.3327	0.1113	16.8578	4.5620	183.3989	14.9105	9.8595	171.4629	9.2995	134.6475	35.1488	35.4427	47.5239	129.0749	3.1819	32.4209	169.4020	80.8852	46.2087	28.9292
TARGET-50-CAAAAC	23.6008	0.1231	29.5913	9.8756	282.8600	17.4815	10.7770	93.4125	7.0739	86.1744	44.2551	9.9960	52.9836	148.5371	5.1475	79.5624	196.0599	64.7501	49.9882	48.4586
TARGET-50-PAJNBN	11.3508	0.0471	19.6035	5.9622	182.8020	14.6544	10.2996	110.9421	8.2992	111.2931	30.4123	15.3650	39.1731	144.1421	8.8002	30.3280	164.7551	60.2149	41.5636	23.1114
TARGET-50-PAKYLT	14.4373	0.3050	28.9578	6.3770	220.2352	16.3161	13.5050	175.0458	12.5557	96.2026	39.7525	30.9302	26.7048	160.4018	8.1187	48.1930	162.3255	90.6502	77.9209	34.8497
TARGET-50-PAJMKN	15.3050	0.0852	40.7266	4.6229	342.7623	11.7719	20.9339	216.9008	19.5671	95.9639	60.1180	57.0101	61.9707	202.3949	28.7948	36.8671	229.6585	133.3844	96.6894	27.6131
TARGET-50-PALERC	10.8258	0.0621	37.3726	6.2864	199.0317	14.0886	13.2782	151.2575	9.6214	124.9020	37.6161	21.5011	33.8604	143.6773	11.9278	28.8710	164.1636	77.0208	57.9506	22.9134
TARGET-50-PAJMFU	39.2820	0.0693	46.4363	13.2219	276.9760	13.1818	17.6798	133.7857	12.8751	149.4415	39.7544	42.6127	45.1424	204.1720	7.1972	27.7391	216.4184	77.0219	66.4918	29.6529
TARGET-50-PAKZHF	18.1192	0.0893	28.8176	5.4604	233.8701	10.7211	19.1880	140.3802	6.9847	178.6965	72.3553	25.5893	22.4953	150.1614	4.8924	29.9054	200.1075	112.9187	62.8443	22.7504
TARGET-50-PAJLSP	14.5653	0.1510	22.9378	5.4170	247.1799	14.9722	18.3902	115.6617	6.2008	101.7544	56.1831	20.9395	78.2468	171.0802	5.8700	84.9429	212.4097	84.1454	60.1665	20.1062
TARGET-50-PAJMKI	22.9498	0.0551	32.3934	8.0164	233.4845	17.3302	13.0118	132.2379	11.2788	84.4919	47.4459	24.2772	42.7845	123.8035	5.9936	32.2715	162.2505	72.6799	55.0254	33.8640
TARGET-50-PAKUIT	25.7845	0.1136	36.2191	12.1395	275.3737	14.9301	14.5271	147.7732	9.0935	277.9907	54.8875	15.7454	72.0098	206.4208	30.3118	67.6017	184.2315	110.4016	72.4588	75.4910
TARGET-50-PALDWP	15.2062	0.0399	30.0877	10.0109	171.1947	14.8079	11.4640	120.2130	6.2548	105.9300	35.9068	14.1444	27.7778	124.7797	12.2368	34.9827	168.4501	65.0097	53.1020	20.3710
TARGET-50-PAJMJT	21.3182	0.1351	45.1760	9.2802	243.3429	15.0335	17.5041	142.7838	10.3966	125.5607	68.9523	23.9744	25.2457	123.7356	6.1310	52.5734	206.4647	66.4569	65.4155	21.7119
TARGET-50-PAKWPM	20.5374	0.0449	25.7852	4.4756	227.2596	13.1173	19.7730	119.0824	11.5307	103.9662	53.0806	38.5925	55.6369	152.7051	7.8046	44.1253	206.6434	93.4519	95.9066	27.3103
TARGET-50-PAJLKC	8.7132	0.1031	16.7996	4.3164	239.2985	14.9839	12.8175	102.1323	4.3219	66.8286	37.2227	21.2357	33.3116	147.7399	4.8947	46.0261	168.9056	61.7094	48.2432	19.4462
TARGET-50-PALKRS	22.6882	0.0615	40.2844	8.3888	318.6839	11.4740	25.3302	133.6002	8.5191	72.6639	169.2127	20.0912	45.9626	155.9755	4.5182	51.9676	181.5173	75.0851	87.8093	22.4267
TARGET-50-PAKNRX	28.7823	0.4205	106.5312	23.1756	558.3934	15.7361	23.9756	221.8174	33.1883	118.7841	75.0218	136.0599	110.9759	235.2908	16.3503	94.9069	299.1332	173.6927	64.4438	47.0091
TARGET-50-PAJNTJ	11.2540	0.0997	34.2086	4.9197	298.4578	14.1926	18.7628	196.6685	9.7812	114.2164	43.8710	67.9959	37.2062	151.7904	9.5688	37.9264	216.0275	128.2993	72.0180	19.7287
TARGET-50-PAJNUS	17.5937	0.0988	32.0139	6.2062	213.1085	15.1273	16.3144	152.9899	10.0212	121.7348	62.4987	26.1354	26.2535	134.3689	9.0249	40.2521	194.1027	73.9434	66.1093	24.1986
TARGET-50-PAKNTW	15.3648	0.0375	30.3496	14.2338	117.9669	11.0398	9.9483	69.1818	3.8416	86.8751	44.3624	6.1028	9.5835	81.7637	1.7374	22.8158	128.5691	41.7448	41.0575	6.7135
TARGET-50-PAJPAU	13.1354	0.0863	22.3218	4.9752	207.1159	11.9186	12.0603	135.6523	8.4691	59.8201	41.9694	19.2937	37.5807	140.1134	5.3990	34.8231	172.7565	65.3311	50.6925	20.8168
TARGET-50-PAJMVU	36.2396	0.0940	56.2451	13.0927	423.9577	15.0667	22.8170	147.4384	16.0554	124.0949	78.0122	39.6659	56.1465	236.0413	9.3801	28.5959	272.3682	101.6736	64.6891	18.8388
TARGET-50-PAJNZI	13.0954	0.1115	32.0329	10.1735	205.6382	15.0773	10.0159	94.8448	6.3047	79.3812	23.4572	20.5371	31.7757	132.1253	16.6401	34.2358	152.3810	80.7393	44.7241	35.5642
TARGET-50-PAJPDN	32.7878	0.0538	76.9239	17.6859	268.3660	11.9637	19.2788	154.6965	9.8980	168.9879	108.2752	24.1546	51.9895	144.8048	9.6433	41.3250	172.6334	72.0681	63.4924	28.1917
TARGET-50-PAJNVE	9.3462	0.1649	23.8268	9.0102	163.4933	16.2933	8.4733	111.5839	5.6977	95.2522	18.4866	8.8555	35.9443	141.6665	6.0804	25.5225	132.0793	43.6834	39.7777	32.3399
TARGET-50-PAJMSE	28.1423	0.0969	40.0607	8.4767	243.8569	13.7788	12.7153	161.3082	8.6556	191.8308	46.2948	32.5041	29.4568	178.9905	10.5054	32.3247	218.8861	71.2419	82.8487	51.3275
TARGET-50-PAJNZU	17.9813	0.1196	32.1931	5.2061	213.2041	15.2558	15.4436	222.9060	9.7165	274.4588	46.8270	33.0499	33.6358	168.3438	19.0912	33.5179	203.0835	80.0860	83.5129	46.3004
TARGET-50-PALGLU	7.8931	0.0611	25.2607	5.5820	189.7289	11.2693	13.7548	123.9289	4.8881	119.9864	59.0662	19.1041	33.9419	126.0947	20.4949	34.7334	162.8116	72.2587	77.6570	30.3342
TARGET-50-PAJNZK	15.0289	0.1610	19.9345	5.1276	271.6479	14.7794	12.1322	171.8763	5.2937	110.1981	46.7292	12.2524	37.2944	143.3377	13.7162	43.2729	177.5336	71.8334	42.6079	24.8239
TARGET-50-PAJMUF	15.6093	0.0550	31.2403	5.7839	174.7185	13.1816	13.1802	125.6617	7.2740	73.9524	44.8644	25.5470	24.7177	140.0699	6.8569	20.8976	165.2200	101.2815	50.4470	17.6496
TARGET-50-PAJMRL	31.6014	0.1614	54.7484	18.8367	246.9779	17.9415	15.5711	148.5155	21.1029	58.1744	29.1671	59.6206	73.9716	212.8506	6.8462	38.8025	189.6890	122.3912	69.6927	45.4686
TARGET-50-PAJMXF	10.6957	0.0638	26.7032	6.1018	224.2333	13.6585	12.6434	120.2583	9.9130	46.0993	36.5968	29.3556	36.3899	116.2599	4.0074	29.2915	153.0461	58.0751	37.5229	14.2395
TARGET-50-PAKPDF	29.8327	0.1033	27.9020	6.4359	171.5736	14.7557	10.4898	110.9021	7.9730	163.2117	33.2652	17.1680	35.7822	130.7195	10.0987	34.4708	144.6431	64.1348	55.6363	23.9144
TARGET-50-PAEBXA	18.0913	0.0664	36.5777	7.0186	296.6163	12.6617	18.6890	165.3478	12.5822	125.2216	70.0378	35.1642	32.5173	178.8334	8.5033	31.9238	210.6450	128.2825	75.5407	28.9180
TARGET-50-CAAAAS	20.7069	0.1740	29.1134	8.2570	249.3053	15.5866	13.4041	243.9472	7.8415	99.3666	43.8806	28.0667	44.8202	147.1087	13.5520	74.8853	231.4907	71.5366	59.9171	30.5491
TARGET-50-PAJNLT	16.2595	0.0811	33.8042	14.6895	218.0929	15.1823	13.0747	152.5902	8.4150	104.8850	46.2475	16.1068	38.9631	145.3759	3.8660	41.2303	189.0120	75.3067	50.0556	22.3594
TARGET-50-PAJPHA	17.4989	0.0979	34.8712	6.1207	258.1585	15.2514	14.4662	160.3279	10.3995	133.6170	64.5470	19.9440	34.7996	155.5832	3.5547	42.8108	187.7211	91.5065	76.0870	32.4062
TARGET-50-PAJMMY	11.7169	0.0556	14.9352	3.7791	162.1518	16.7131	8.9346	145.9358	4.5466	77.3561	41.2641	15.3583	25.4865	110.5850	1.0965	32.5762	123.9533	45.9347	54.9799	24.6572
TARGET-50-PAJLTH	16.5450	0.0911	18.5382	3.0001	248.3436	12.2153	15.1133	113.5464	6.7464	59.6211	55.5288	9.3961	37.8334	165.7380	4.7699	37.3626	192.2759	56.7523	47.7903	12.3720
TARGET-50-PAJNCC	18.8507	0.0959	61.2001	7.7952	365.6038	16.6600	16.7703	144.0882	5.0293	156.9382	45.4864	11.7278	51.3763	215.5326	15.6534	64.3188	256.6919	102.8835	71.4167	55.4273
TARGET-50-PAJLUJ	13.5441	0.0351	23.5281	3.9718	195.3989	13.1109	16.9314	124.4150	5.7574	64.5310	55.0336	19.8693	22.9374	97.8915	4.0888	25.6852	155.1734	77.3609	67.6519	17.6955
TARGET-50-PAJNCJ	17.1806	0.1506	37.0499	6.4009	234.9508	13.3469	12.1429	154.6536	9.3886	219.5585	49.4300	35.6805	41.0274	164.4679	20.7265	29.0532	200.2084	95.5096	65.2814	22.3443
TARGET-50-PAKZER	16.9021	0.0616	20.7874	4.9294	229.2948	14.3212	14.4240	116.0150	6.7854	114.1393	66.3218	13.8312	28.0969	141.5422	13.2684	29.7803	183.7101	132.7546	94.9993	44.7974
TARGET-50-PAJMEP	12.4616	0.0619	20.1704	5.6201	168.3278	15.3873	10.5045	170.6636	6.2358	86.6028	33.7067	21.9236	30.6782	121.2630	5.2556	30.3145	152.2107	57.6585	48.8762	17.4508
TARGET-50-PAJMEL	14.4120	0.0828	25.8353	3.3926	190.5815	13.5209	18.3591	225.3978	10.0671	210.1083	59.1558	48.9251	22.7188	137.4544	15.9833	35.3588	187.4219	87.0247	84.7993	20.0209
TARGET-50-PAJNVX	56.4645	0.5680	39.0018	5.0847	304.8333	13.5295	25.0303	356.8777	14.9993	244.4977	81.7734	171.5912	47.3194	212.4898	15.6169	54.5415	213.2608	383.9612	221.2516	87.9310
TARGET-50-PAKXWB	15.9306	0.0808	21.8737	4.4269	224.5618	12.9265	13.3979	148.2397	4.7345	42.7858	58.4885	30.2963	31.6499	103.9345	3.3186	28.7597	145.8391	97.0005	59.8091	22.0313
TARGET-50-CAAAAB	9.5914	0.1417	17.5084	5.9867	258.0556	15.6559	14.8488	119.1534	4.7677	99.6666	48.7887	19.6560	63.7531	156.7838	3.0716	52.8087	162.0108	60.6747	53.4753	25.9896
TARGET-50-PALJIP	9.7237	0.1501	24.9521	5.0119	229.6888	12.4336	13.9301	119.8203	5.5628	80.8497	55.8353	12.1907	28.4385	120.1941	6.7833	41.2428	159.7877	74.3396	46.4443	14.8401
TARGET-50-PAKMUB	23.0045	0.2605	41.2955	4.6949	335.5659	12.8977	29.3400	283.7764	10.7588	132.8811	110.1178	67.7360	45.7899	170.3781	27.5612	49.5651	251.0278	207.3689	200.3075	36.8760
TARGET-50-PALKCW	22.8428	0.0518	43.6793	6.8777	249.0370	11.2822	15.5632	132.2423	11.8098	136.7955	42.8398	43.9840	41.7184	158.1750	10.9447	21.5676	195.8934	114.8016	62.9509	22.3299
TARGET-50-CAAAAQ	8.8143	0.0606	21.5544	7.6859	149.4410	13.8572	10.2263	95.0683	4.2217	61.8359	39.3758	8.8573	27.0863	126.2540	3.3869	28.2774	145.8025	41.4497	41.3504	18.1685
TARGET-50-PALFME	24.7694	0.1094	35.4215	13.3358	285.9748	15.6087	12.6597	112.9883	7.7877	161.7197	47.6700	19.1414	38.0055	174.4620	8.5987	41.1581	213.8857	77.4875	50.1059	34.9754
TARGET-50-PAKNXS	26.5681	0.4013	70.7450	16.5777	307.2178	14.5692	16.9379	175.3542	11.1567	124.1831	81.9474	39.7040	101.7224	223.4807	18.3261	72.0516	218.1219	119.0531	76.9054	31.9660
TARGET-50-PAJNAA	62.3133	0.5432	76.6897	13.2660	943.3122	11.9353	36.2072	406.0207	37.0376	229.0223	203.5957	175.3181	54.7163	366.4545	30.9985	99.9756	388.0157	254.3303	159.9716	43.9521
TARGET-50-PAJPGY	11.5454	0.0884	24.7937	5.6834	258.7176	13.8975	11.9678	108.3794	5.9266	140.5735	60.5781	27.2417	24.1018	124.2061	6.7881	35.8109	153.6001	73.6712	69.9424	41.2722
TARGET-50-PAJNEC	27.1873	0.0152	41.3499	11.7820	401.8084	12.5286	24.8922	102.4337	9.3783	38.8553	245.5959	18.3814	52.8646	147.6414	1.2841	59.1763	191.0651	47.0951	52.6383	17.4512
TARGET-50-PAJNDU	8.2966	0.0160	38.5344	8.5693	195.1455	11.2027	16.0326	88.5852	5.9121	44.1961	129.3906	6.4435	29.0747	106.9763	2.6756	24.1659	133.9641	47.8112	40.4910	9.3190
TARGET-50-PAKGZX	23.4377	0.0505	21.8173	5.4223	179.5594	11.4019	16.7948	340.1781	4.7412	184.7102	77.5147	42.2745	15.6179	101.1106	10.3070	27.5675	157.4285	110.2665	113.6382	13.9028
TARGET-50-PAKFYV	26.1526	0.1068	52.8154	21.8907	235.6591	14.7087	10.4519	145.4287	9.1373	173.4625	26.6617	41.8890	54.1673	149.7042	16.2183	48.7035	162.6361	83.4352	44.3378	47.0749
TARGET-50-PAKGED	7.5795	0.0596	39.1148	11.1037	272.9873	8.9853	38.2630	144.6912	7.5562	37.4716	42.4001	11.3189	42.5688	135.5292	19.6275	120.8107	163.9871	45.0827	69.8516	7.1313
TARGET-50-PAJLTI	22.5447	0.0932	21.2591	3.2914	239.5737	14.9328	17.6588	186.7353	13.3999	147.9542	60.6245	37.9657	42.3454	188.5327	13.2233	47.1214	223.4909	100.8024	90.6394	40.5690
TARGET-50-CAAAAO	21.1407	0.0651	59.1084	14.2534	240.9329	11.6033	15.9383	115.9226	8.1415	115.1893	44.8398	25.9908	30.3554	160.1574	7.7410	30.8881	175.7426	73.3617	49.4185	24.1598
TARGET-50-PAJNCZ	25.8904	0.0602	35.1774	6.7175	210.3420	14.5894	14.6114	165.6357	13.5490	151.7798	41.0497	36.8340	38.4206	156.1986	11.4023	31.3092	186.1229	83.5224	72.6697	32.4933
TARGET-50-PAJMIZ	42.6315	0.0573	39.9007	9.1863	302.0395	12.6171	16.2740	116.4755	9.6917	85.3792	74.2605	42.3069	60.6288	160.9919	2.2036	38.4877	191.4392	72.5545	68.6936	36.0771
TARGET-50-PAJMFY	19.0081	0.0688	37.0871	8.9576	255.0255	13.8497	13.6972	156.3831	8.6266	134.1672	41.3418	37.0980	42.7089	159.9195	9.7901	33.0004	191.6151	66.6851	59.1239	26.4010
TARGET-50-PAKULH	25.9955	0.0961	29.8494	9.2756	201.0063	14.9399	12.3886	83.3177	4.0717	61.6078	43.4337	20.7446	49.0995	126.2980	6.8197	47.7677	158.8349	62.4026	60.4288	49.9498
TARGET-50-PAKVET	26.5439	0.5148	56.0470	7.3011	338.4035	11.8589	18.7982	345.9691	30.8731	172.1782	70.1891	95.0650	56.9611	215.7574	51.6358	45.6334	265.1812	142.5059	111.8461	35.1779
TARGET-50-PAJMEN	14.7043	0.0522	31.3275	5.5110	262.6592	11.5543	12.0808	114.0408	11.4081	114.4207	45.0499	27.0341	54.8762	145.1609	11.1393	36.0785	188.3351	97.3159	58.7349	26.2584
TARGET-50-PALDTE	32.8265	0.0935	40.3622	11.3332	220.6498	14.3249	13.1919	136.5384	8.7800	168.4276	51.1859	40.7429	37.6430	167.5756	8.7232	34.4324	192.3257	63.3722	67.1331	37.2570
TARGET-50-CAAAAH	37.4971	0.0706	50.2101	39.0481	267.3344	10.8309	16.7361	102.6209	6.8955	70.3643	163.3561	6.7235	50.8775	153.7336	6.5122	43.7660	165.9666	45.6554	48.6907	16.6757
TARGET-50-PAJNRH	14.5829	0.0720	34.2470	6.6427	264.6253	12.1856	15.1940	118.6295	10.9967	57.9933	38.2174	31.5903	70.0458	173.1488	9.4875	34.7757	191.3544	105.7586	59.7944	34.7881
TARGET-50-PAJNRL	23.7300	0.0789	18.3863	8.2158	243.2043	13.9970	11.6827	101.8467	5.1885	100.0032	32.6707	15.9120	43.8331	157.8118	2.4026	36.6628	161.1935	69.2921	46.5515	24.2554
TARGET-50-PAJNUP	73.4865	0.7184	61.8341	11.3022	624.2197	12.5475	42.9017	285.2180	12.9712	158.7640	214.0900	65.1634	51.5248	235.4138	18.3186	92.6311	293.0244	275.1260	177.5263	32.5375
TARGET-50-PAJPDC	10.6977	0.0980	19.4622	6.2624	185.9246	10.3759	14.2088	139.3740	2.7297	150.3177	52.6527	31.7323	26.1050	98.6716	12.0000	41.2999	141.6907	89.6973	76.0276	21.4661
TARGET-50-PAJMLI	28.8230	0.0748	24.4664	4.9156	184.0287	15.1400	13.8206	142.9388	9.6748	216.2977	50.6695	34.5340	35.9064	163.9192	5.2569	39.6322	161.8369	82.3811	82.1409	32.0849
TARGET-50-PAJLWT	10.5850	0.0587	51.6209	10.4980	251.3637	13.0549	17.3732	127.5707	10.2197	72.8908	47.0933	40.1346	41.2559	183.2922	6.8784	32.1893	216.5523	98.7082	63.1345	28.6205
TARGET-50-PALEZT	34.6516	0.0647	52.1656	13.0463	224.4221	12.9781	14.1054	126.9519	15.4254	145.3037	37.7845	49.8323	84.9423	125.1350	5.3402	30.8289	155.5817	118.8704	75.9412	40.2830
TARGET-50-PAJLLF	72.3336	0.1942	50.9103	15.3906	503.2025	16.3226	26.7405	135.7761	10.0892	148.4859	76.7001	71.9617	78.4955	245.4380	12.7384	94.4330	231.8021	143.2822	131.1799	49.7735
TARGET-50-PAJLNJ	17.7480	0.0549	39.3697	7.1701	190.9863	12.9587	13.9834	121.5518	7.4145	133.0317	49.4376	19.0160	28.4691	138.0688	5.7196	25.4705	178.1120	69.9476	65.7032	18.6978
TARGET-50-PALFRD	16.1653	0.0462	29.0797	4.9907	219.0937	11.6248	13.4917	110.2455	7.7900	41.9160	43.9031	25.6233	38.8222	126.7501	2.0524	22.6238	149.1129	75.0685	45.7957	12.8963
TARGET-50-PAKKSE	20.7483	0.0780	26.2750	4.7754	226.9623	13.7536	17.7158	198.2278	9.9830	123.9312	61.5330	41.6158	24.3393	140.6775	10.3227	31.7371	195.6293	87.8189	78.6855	18.8399
TARGET-50-PAJLPX	48.5828	0.1527	38.9339	12.4850	310.8237	14.7339	14.3782	156.7911	8.5723	216.9279	51.3040	16.8689	43.5204	171.0861	24.1109	56.9869	190.7090	121.7948	93.3968	62.9861
TARGET-50-PAJMKJ	24.0624	0.0972	27.9780	3.9410	316.9238	13.6117	17.3749	119.2577	6.2760	113.1050	86.7118	25.2132	47.2274	141.2433	11.4219	70.3563	191.6669	151.1368	104.8960	54.2989
TARGET-50-PAKSDG	9.6640	0.0803	25.8104	6.0189	220.1065	15.3015	12.4481	142.8277	7.7412	85.4821	49.0810	22.8484	37.2822	150.4164	13.0398	30.7816	185.0211	72.9846	48.9392	22.4703
TARGET-50-PAKZFK	30.4741	0.0911	40.8355	8.1721	289.8291	13.5884	17.2251	147.1441	10.8498	179.2077	68.2736	38.9791	41.0207	185.5237	3.2630	36.6494	220.0358	105.1300	105.9672	35.6085
TARGET-50-PAKYFC	35.7182	0.0872	27.2265	5.8470	211.1356	13.4414	14.2134	219.3999	14.7074	229.3842	41.6099	37.9738	35.5403	162.9442	17.7968	37.4630	208.1396	118.4424	95.9896	43.9279
TARGET-50-PAJNNR	40.1676	0.0890	47.7200	10.7409	283.8939	14.5106	20.8055	157.7727	10.9065	108.1359	64.0101	24.8689	110.7958	203.7923	10.9553	65.1670	207.6515	97.1477	82.7897	36.4310
TARGET-50-PAJLIP	37.8961	0.3023	69.4401	15.2258	526.0213	18.5526	20.3637	274.5551	18.8389	171.7892	69.3899	86.3458	107.0658	226.6715	30.8756	138.5438	302.5402	239.7400	133.1300	103.5726
TARGET-50-PAJNYT	34.7037	0.2338	46.4623	5.3430	273.6701	14.7709	18.1249	237.7922	8.2252	331.6476	61.3836	93.9109	37.9170	182.0457	32.7928	34.8988	208.2545	122.6399	97.0596	47.1985
TARGET-50-PALGVY	19.7341	0.0884	45.0886	9.1770	232.1884	10.4071	19.9551	113.7410	11.7272	106.1058	74.7384	22.0411	23.9641	151.1498	10.4653	28.5189	196.7804	94.4997	70.7023	12.5728
TARGET-50-PAECJB	13.7028	0.0731	21.2289	3.1477	152.8265	14.1608	11.8211	150.1615	6.0804	122.3623	36.2891	22.0949	30.8345	112.3262	11.8297	24.0422	146.2344	72.5432	58.6719	17.9175
TARGET-50-CAAAAM	15.1923	1.0932	39.4039	6.8166	318.1624	11.1019	29.2795	114.0865	3.4563	54.8902	133.2996	25.1115	16.1205	111.8755	16.4711	65.8812	212.8783	112.0355	91.1099	9.4930
TARGET-50-PAKSCC	9.6705	0.0402	40.3666	9.9885	223.9869	14.6109	14.1764	101.9053	4.9548	78.7555	45.6600	14.5078	35.1679	143.4728	6.1179	29.5715	149.6167	66.4289	57.0551	23.1875
TARGET-50-PAKRZW	10.4292	0.0759	24.8971	2.7236	250.1173	11.7416	19.6568	155.9498	6.7405	54.1625	58.3654	29.5018	33.4862	146.8511	5.8434	32.3757	193.8407	116.0860	77.0028	20.8224
TARGET-50-PAJNSL	8.6821	0.0851	28.3792	4.1467	189.1013	14.0788	12.4638	164.2018	9.9171	149.8937	38.0161	22.5532	29.6006	141.7470	15.0386	26.9591	160.0761	70.9029	55.7764	22.7061
TARGET-50-PAKKNS	38.3151	0.0947	45.7238	12.5984	314.1456	12.8719	22.2276	224.6764	15.0603	172.0379	61.5522	49.2732	61.8944	207.1859	12.8133	40.8145	210.1795	147.9645	105.4293	32.1219
TARGET-50-CAAAAL	35.5896	0.1082	41.3648	9.2424	353.9515	14.5898	18.4589	170.6654	19.0098	188.8900	46.4875	44.3511	48.3062	215.7133	13.4086	35.3176	253.2424	80.0529	63.0793	34.2053
TARGET-50-CAAAAJ	21.6919	0.0689	49.5099	10.2251	219.2870	14.2689	17.4054	124.9108	9.0251	200.5036	55.5295	22.5837	29.9424	158.7852	7.0108	32.1485	190.1301	64.1386	69.8384	25.2480
TARGET-50-PAKNAL	18.3391	0.1245	26.4756	3.3982	268.4704	15.4054	24.5453	182.7511	8.1146	298.2066	89.0687	65.0828	70.8177	193.2725	13.1447	73.9979	215.3048	130.3359	147.4413	32.0720
TARGET-50-PAKXXF	22.5572	0.1061	47.1679	35.2236	187.7473	12.0006	12.7514	88.7455	13.4182	38.8403	45.6908	12.9453	42.3936	138.7155	6.2279	37.9128	152.6666	82.6392	43.4598	22.0715
TARGET-50-PAKECR	19.4866	0.2990	25.1255	4.6092	330.1635	11.7697	23.3749	108.9296	5.7165	146.7877	80.4626	31.6525	30.7069	208.8569	35.6523	45.9461	229.4363	144.2526	114.8700	37.7191
TARGET-50-CAAAAP	42.0648	0.1463	36.1172	9.4381	305.7934	14.4607	16.7657	149.8266	6.5441	273.1417	46.4665	53.1273	37.7352	168.7899	33.0558	60.3136	189.7478	118.1125	84.4817	49.0094
TARGET-50-PAEAFB	31.8925	0.0760	38.9489	10.0832	251.2276	13.8176	15.8704	132.4129	12.2996	133.7221	45.4720	31.1009	36.2002	188.5971	13.7922	41.1178	233.0518	98.2691	81.8222	32.1452
TARGET-50-PAJLKR	35.8572	0.1366	36.0956	13.5397	484.1525	14.8393	25.2859	148.2202	7.8504	149.1000	76.7066	30.0574	44.3448	187.0413	6.1779	89.5026	247.8352	98.6608	61.9036	22.5118
TARGET-50-PAKMSV	17.4822	0.0310	33.4333	13.2496	168.6055	13.9073	10.3409	83.5577	5.2734	45.1868	44.1505	11.7141	22.9660	92.1484	1.0584	26.4052	143.3992	42.3885	36.1403	15.9528
TARGET-50-PALLFB	26.2027	0.0816	37.4714	12.3353	242.6574	15.9033	19.0539	192.6041	13.4277	145.4037	29.9773	32.4231	64.0262	193.1522	32.1675	47.7391	183.3553	122.6520	65.7267	72.3213

Supplementary table 1 continue5

	RVX-208_1625	OTX015_1626	GSK343_1627	ML323_1629	Entospletinib_1630	PRT062607_1631	Ribociclib_1632	AGI-6780_1634	Picolinici-acid_1635	AZD5153_1706	CDK9_5576_1708	CDK9_5038_1709	Eg5_9814_1712	ERK_2440_1713	ERK_6604_1714	IRAK4_4710_1716	JAK1_8709_1718	AZD5991_1720	PAK_5339_1730	TAF1_5496_1732
TARGET-50-PAJNAV	150.2949	13.4613	15.1949	95.9814	53.4755	23.4326	40.7834	54.6709	174.4388	4.8527	0.5681	0.0720	0.0614	9.0878	61.2290	175.0832	90.2800	222.9608	11.6629	62.0442
TARGET-50-PAJPEW	144.5757	7.0558	20.8703	108.8153	59.9093	37.9690	42.6071	88.5767	274.2575	3.6841	0.9428	0.0978	0.0459	6.6629	15.9302	182.8809	103.1446	220.7065	14.2024	41.4344
TARGET-50-PAJMLZ	170.4832	39.8162	21.2948	116.0462	64.8640	42.6326	48.2485	96.4264	212.1828	12.9303	0.7540	0.0918	0.0588	23.0977	60.4899	169.6291	84.5417	133.7279	11.1582	47.2402
TARGET-50-PAKRVH	112.6092	13.0367	19.7058	109.1007	45.0093	32.1542	48.1905	79.1909	223.9427	7.3291	0.8146	0.1396	0.0534	21.4877	38.1444	144.8898	82.7361	92.4758	11.0163	64.4980
TARGET-50-PADZUB	123.0423	7.2775	19.6897	71.8568	61.7934	27.5937	46.5756	83.1067	220.0072	2.2760	0.6783	0.0930	0.0255	5.1431	9.9479	141.4126	87.2632	79.0666	9.8542	19.8335
TARGET-50-PADXAY	145.8978	11.1977	16.3653	55.4811	39.5850	45.0850	53.6090	33.3415	133.9555	8.7792	1.4649	0.3166	0.0606	38.3730	75.6327	199.9756	156.6440	79.7930	16.0462	52.2414
TARGET-50-PAJPCM	108.5142	16.2952	17.4653	62.0473	48.7317	44.2676	50.3062	43.3396	119.6810	6.0784	0.8452	0.2626	0.1659	34.0644	56.1447	118.9333	124.8531	84.8442	14.5788	18.4771
TARGET-50-PAJPAR	147.5123	23.5499	21.2980	59.9557	75.1439	54.5398	50.0024	47.6539	235.5910	10.8282	0.8835	0.1475	0.1477	46.8089	85.7409	120.6547	78.9851	158.2149	12.5907	16.6784
TARGET-50-PAJNZS	134.6647	17.9182	20.9195	104.6088	48.4547	39.7631	47.9605	66.8531	164.8088	11.1307	1.4094	0.2160	0.1084	20.6626	34.9124	172.4417	97.8890	103.3572	14.5292	60.4671
TARGET-50-PAJMVC	105.4492	11.0554	11.6048	78.7645	25.1133	24.1426	47.4718	39.3176	123.1771	6.1521	0.3268	0.0439	0.0549	24.3806	56.2390	154.5533	54.1490	25.5890	10.9904	83.3566
TARGET-50-PALGAZ	122.9585	12.9027	18.4974	112.1850	35.4235	28.0636	50.8653	78.2278	179.1528	6.0499	0.8348	0.1391	0.0557	10.2469	32.4869	142.1743	76.3039	91.6286	11.1338	51.0801
TARGET-50-CAAAAR	102.0292	8.5769	15.5784	112.5738	35.5270	24.7269	49.1339	77.8745	158.1726	3.6226	0.7020	0.1102	0.0347	9.5223	30.4717	138.6844	59.0467	57.4965	11.9855	45.7829
TARGET-50-PAKGMU	170.2823	17.1581	19.2264	105.8623	56.4601	35.2289	51.9844	63.3341	192.3690	9.0759	0.9281	0.1298	0.0679	19.1959	32.0616	173.8919	98.6180	127.2600	11.6170	62.5878
TARGET-50-PAKRCC	95.1401	10.1077	13.9149	50.8281	54.3131	32.3857	54.8180	43.7760	170.0929	3.9800	0.6709	0.1094	0.0420	18.4901	45.8045	143.1685	78.1745	36.6584	8.9763	16.3978
TARGET-50-PAKFME	105.9455	9.6947	16.8344	85.9942	40.4154	25.4691	49.9191	66.0582	181.1083	4.8939	0.6275	0.0999	0.0282	10.2792	21.1999	137.7518	65.5655	42.8542	10.1522	51.4793
TARGET-50-PAJNNC	142.6835	16.2317	21.3381	113.2211	60.4548	30.2248	46.7574	74.6568	197.9609	4.0093	0.6835	0.0878	0.0466	10.5298	26.7479	161.3762	108.7674	154.2293	14.0375	53.6957
TARGET-50-PAJNJJ	174.7383	21.2126	28.3387	131.2026	72.1865	47.2430	51.0267	83.3067	274.1322	8.9346	1.3437	0.2070	0.0883	15.1788	33.7288	212.9156	82.7552	277.1315	13.8082	42.7538
TARGET-50-PAKJGM	61.2633	4.3517	10.6367	54.6745	21.1047	17.6309	46.5460	38.1823	123.5256	2.6757	0.3539	0.0413	0.0145	7.0203	10.8783	96.1147	54.9793	27.1071	4.8161	70.7130
TARGET-50-PAJNGH	87.6897	14.9558	13.4156	91.0747	31.9007	26.2312	48.4478	70.2948	142.1274	6.1673	0.6129	0.1015	0.0716	11.7463	26.4239	120.8626	47.8767	70.3900	10.1721	33.1950
TARGET-50-CAAAAC	103.4261	28.5513	12.8201	65.1141	65.8950	30.0174	51.1883	55.8861	157.5516	15.5688	0.8386	0.1055	0.0892	25.8556	38.2476	126.6802	78.3981	30.9873	8.7883	21.8419
TARGET-50-PAJNBN	79.6499	7.2933	14.0636	64.8697	31.3880	20.2196	53.0088	55.6107	130.2207	2.9362	0.3516	0.0560	0.0265	10.0305	25.6526	80.4275	42.9557	25.2534	6.9227	23.7673
TARGET-50-PAKYLT	121.3630	14.1231	19.1676	96.6902	41.6169	25.2228	48.6885	49.3724	144.5406	6.6957	0.7181	0.0710	0.0986	51.0232	169.1072	150.4578	53.8438	91.8008	15.0125	48.1895
TARGET-50-PAJMKN	137.1659	10.9026	19.5234	115.2385	29.6878	34.0381	45.6075	82.4771	210.3460	4.4616	0.5932	0.1284	0.0542	20.0874	59.7644	192.3792	86.9424	132.5802	11.0855	49.0822
TARGET-50-PALERC	95.5067	9.2750	15.2577	71.4576	29.3192	25.0849	46.3227	50.0803	141.0425	4.4468	0.5354	0.0902	0.0381	13.7949	41.1772	115.6249	55.3419	44.0397	8.5758	40.6372
TARGET-50-PAJMFU	107.0669	10.5440	18.3275	99.0800	35.1137	28.0248	49.5292	83.2331	167.3050	5.6091	0.5751	0.0974	0.0417	12.8025	22.5385	113.0549	54.1942	74.7205	7.9200	45.0659
TARGET-50-PAKZHF	112.8969	6.8743	15.2453	117.6293	34.7859	19.2558	46.1042	110.4250	203.7497	3.7229	0.5752	0.0695	0.0359	4.1186	16.7996	158.0294	61.0753	100.1125	8.9457	51.3000
TARGET-50-PAJLSP	93.5989	12.7836	15.2185	79.9895	79.6953	36.2431	48.1794	79.6121	176.2066	5.3121	1.1444	0.1506	0.0343	15.2030	34.8095	153.4100	87.5104	72.1746	9.3834	30.6686
TARGET-50-PAJMKI	134.7999	16.5276	17.4986	84.3763	44.0752	30.0003	55.5433	49.6587	139.1650	8.9842	0.7295	0.1240	0.0452	15.8892	30.0537	133.1474	72.3841	60.2933	12.4418	40.2577
TARGET-50-PAKUIT	137.9499	44.8379	17.5508	72.8012	77.7213	33.9966	46.7363	32.0899	175.7411	15.6065	0.5376	0.0660	0.0839	20.1704	39.9928	122.2386	70.9176	157.5629	14.7868	30.3150
TARGET-50-PALDWP	91.8136	7.4347	13.5049	65.1482	32.3467	18.3941	52.6385	50.6107	148.4586	2.8977	0.4409	0.0552	0.0252	8.3755	20.8293	90.6715	39.5703	39.6813	6.5044	29.5831
TARGET-50-PAJMJT	92.4485	6.8501	14.0086	99.3903	41.6601	19.3826	50.1630	89.5555	195.2796	2.4378	0.6808	0.0942	0.0250	7.0317	13.4316	125.0593	51.7793	53.4190	9.1381	43.5406
TARGET-50-PAKWPM	122.9042	13.8932	17.9406	103.8543	45.8998	33.2350	47.2749	84.3710	192.2130	6.1748	0.6445	0.0784	0.0271	8.9562	19.0128	159.1490	68.0370	94.3769	9.3622	49.1532
TARGET-50-PAJLKC	85.4199	8.8000	13.0423	60.1452	40.0196	23.4612	55.1645	56.2230	147.4942	3.1196	0.5908	0.0665	0.0335	7.5627	23.9005	119.1926	75.6049	62.5195	7.8157	19.1125
TARGET-50-PALKRS	127.4608	10.1312	17.1188	102.3517	35.3010	22.0587	45.1401	65.4579	193.5663	4.4190	0.3956	0.0439	0.0289	15.5718	20.8796	170.9196	74.3590	44.0771	11.3028	100.8546
TARGET-50-PAKNRX	172.7122	25.6526	28.4666	132.4040	72.3510	62.1538	53.8046	105.7718	206.1802	10.5231	1.5164	0.4490	0.2965	29.9921	38.4249	184.9830	164.5492	150.5743	15.3831	31.6083
TARGET-50-PAJNTJ	79.8245	7.1113	15.5133	92.8864	34.3325	26.0952	45.9785	73.9749	177.3747	3.2615	0.9266	0.1529	0.0333	8.7023	25.1608	114.9464	67.0404	76.6916	8.7228	45.1982
TARGET-50-PAJNUS	103.1724	8.1488	15.6517	92.6211	39.9794	19.0440	50.4503	80.8564	180.7555	3.2174	0.5705	0.0754	0.0258	7.9073	20.9235	118.7943	45.3244	62.7969	8.7857	44.7863
TARGET-50-PAKNTW	61.8014	2.4055	11.1147	51.4108	29.1586	9.2879	44.2509	38.6241	101.9796	0.9776	0.3239	0.0397	0.0130	4.5947	8.5643	76.8992	24.8916	28.8389	6.9310	42.3632
TARGET-50-PAJPAU	68.3983	9.5972	14.0689	81.8242	26.5009	18.2926	49.0814	56.9455	143.7577	5.2670	0.6936	0.1755	0.0568	13.1528	24.4362	131.5837	38.0901	98.5088	12.2335	52.4031
TARGET-50-PAJMVU	100.3316	7.6667	20.5432	83.3592	35.4850	32.1611	55.2430	72.4370	252.7379	5.3731	1.1914	0.1716	0.0447	19.9496	28.1519	150.6089	104.9891	99.6589	8.7868	62.6541
TARGET-50-PAJNZI	104.9734	14.2803	14.1101	47.9009	47.9175	27.9645	52.3297	36.7549	132.3166	4.4838	0.3264	0.0413	0.0253	13.3878	23.3999	74.3348	42.6794	19.6103	9.6746	16.7115
TARGET-50-PAJPDN	112.5715	14.6049	15.9486	86.7155	32.4330	26.2412	42.1818	52.5460	165.5506	6.7431	0.3974	0.0700	0.0681	29.4295	32.9718	157.8644	96.0423	54.4142	14.8797	81.0225
TARGET-50-PAJNVE	70.3763	14.6016	11.5278	39.5896	25.1472	22.0201	47.9927	21.9565	107.9378	4.5982	0.3228	0.0543	0.0259	27.8930	72.4059	61.0663	30.8226	18.0200	8.5904	16.8951
TARGET-50-PAJMSE	124.1276	21.3687	16.9929	108.0498	27.4223	23.8483	50.0257	77.7135	183.4562	8.4381	0.5254	0.0932	0.0350	12.5549	38.1468	141.0419	59.1148	114.9149	8.9140	48.8608
TARGET-50-PAJNZU	138.0968	15.5267	19.6510	112.1986	36.8469	25.1985	51.2399	85.7221	179.1897	5.4762	0.5952	0.0949	0.0442	8.7164	32.8573	140.2026	50.7024	60.3363	11.5746	43.6463
TARGET-50-PALGLU	131.4472	12.7786	15.6330	73.8260	35.8177	20.4471	46.6684	40.2139	156.0403	4.8159	0.4872	0.0598	0.0370	16.7380	33.1001	120.7150	51.0302	39.4281	11.9516	40.1441
TARGET-50-PAJNZK	89.4959	8.5929	15.3347	66.7234	48.2550	20.6352	51.4813	44.2861	141.5761	4.1860	0.5223	0.0773	0.0567	12.5048	18.8741	88.5550	43.3603	51.3363	13.2591	27.1351
TARGET-50-PAJMUF	95.0799	6.5974	13.8582	64.5608	39.0373	22.8705	48.2830	42.0806	149.9573	3.1684	0.5628	0.0824	0.0196	8.7990	15.4029	106.7512	58.3966	52.1257	9.0372	36.1585
TARGET-50-PAJMRL	187.8496	26.0853	22.3843	78.6411	67.4893	83.0849	55.3146	45.0054	193.8148	10.5314	1.2427	0.1903	0.0399	43.8373	76.1157	122.5914	109.5838	31.6111	12.6167	23.8053
TARGET-50-PAJMXF	73.7662	5.3349	12.5086	68.5530	31.2623	20.6287	48.0959	60.7336	141.2511	2.1863	0.4386	0.0599	0.0146	8.9225	24.7172	117.9105	45.7534	36.3322	7.6927	33.8415
TARGET-50-PAKPDF	83.1264	10.3253	13.0292	68.1163	36.7664	28.7215	51.7244	40.3690	97.8118	4.1328	0.4919	0.1062	0.0559	21.7128	56.8197	125.9266	60.8720	47.8960	12.3603	22.0528
TARGET-50-PAEBXA	138.3185	13.7879	18.3915	104.0438	32.3820	24.1934	47.5818	78.5428	198.7099	6.8477	0.9176	0.1217	0.0821	13.0079	38.5186	151.4278	55.2951	148.6514	10.5011	72.4441
TARGET-50-CAAAAS	101.1383	13.1356	14.5361	107.1982	54.3634	25.1784	51.0587	81.0272	161.1006	5.1146	0.9282	0.1248	0.0451	14.2964	37.2211	165.8136	56.4742	45.5282	12.7922	35.4696
TARGET-50-PAJNLT	113.4598	9.2877	15.7913	74.3232	37.2353	31.3071	49.4953	51.9150	165.1727	5.1660	0.8026	0.1029	0.0305	17.2942	48.4628	138.3903	73.4739	90.8167	12.6904	35.7437
TARGET-50-PAJPHA	124.6815	15.1473	15.6632	88.0664	35.4127	23.9112	46.1953	56.0241	179.1025	9.1486	0.9806	0.1251	0.0545	16.0198	32.4538	136.9357	56.0644	93.1719	11.5476	66.5988
TARGET-50-PAJMMY	77.5064	10.1396	12.1924	64.5069	28.7807	19.3583	53.7418	37.8322	143.8820	4.8116	0.5100	0.0499	0.0276	13.9796	42.8639	114.2218	56.5651	65.8420	8.3099	48.7007
TARGET-50-PAJLTH	77.3543	5.5399	13.2148	61.3262	37.3082	19.1278	50.0703	49.6756	146.8022	2.2655	0.5928	0.0847	0.0255	5.1742	11.3227	125.8261	40.5955	93.1037	8.7909	38.8980
TARGET-50-PAJNCC	180.2432	22.5686	22.7369	85.6418	54.0526	15.0499	33.6840	60.8040	199.6853	5.1994	0.6482	0.0736	0.0497	15.6544	23.2803	120.2012	72.1237	191.2241	8.3846	38.3474
TARGET-50-PAJLUJ	86.9923	5.5695	12.9836	103.1853	21.4862	14.4669	45.4945	61.3045	141.8853	2.8377	0.3484	0.0396	0.0210	5.3988	15.3352	102.3551	25.3300	41.2866	7.5824	90.9964
TARGET-50-PAJNCJ	103.4027	10.6267	15.9127	81.9645	40.2120	22.8950	45.8124	57.9199	133.8461	2.9106	0.6561	0.1130	0.0594	13.9169	39.5496	156.8160	56.2999	52.2285	11.5795	30.2823
TARGET-50-PAKZER	174.7384	23.2779	17.2006	81.2268	34.2733	22.5697	47.5409	49.1017	174.6445	16.0803	1.3329	0.1337	0.2029	18.2275	44.1718	130.7983	51.2850	129.2791	12.5040	75.5882
TARGET-50-PAJMEP	77.0174	9.5052	13.6289	62.5642	30.6191	21.1517	49.4655	45.1127	135.5512	4.2898	0.6339	0.0830	0.0237	11.9767	43.9159	121.4843	45.8676	58.1319	9.6820	30.7203
TARGET-50-PAJMEL	100.0475	6.7528	16.6793	129.9166	31.1431	17.3047	45.9888	96.1368	151.6709	3.2085	0.4988	0.0593	0.0308	6.1780	25.9153	163.1936	42.0805	112.1703	14.3890	69.9774
TARGET-50-PAJNVX	185.8041	43.9151	21.6017	243.2785	45.0987	38.1785	44.0834	102.9543	248.8139	26.4237	1.9686	0.1125	0.3252	65.9490	74.9574	174.7522	53.6455	38.1162	19.7602	140.9107
TARGET-50-PAKXWB	80.1399	7.9511	13.4926	96.4079	23.6293	15.2902	45.5384	56.6468	134.9127	6.0373	0.7423	0.0811	0.0452	13.7830	36.5443	131.1388	39.5457	86.0132	7.5390	119.5588
TARGET-50-CAAAAB	92.0912	13.0893	12.1613	68.3611	51.0960	25.9145	54.1337	54.1696	134.8360	7.2385	0.7961	0.1008	0.0552	16.3786	29.7129	134.8975	80.6391	106.5792	7.5183	29.2046
TARGET-50-PALJIP	99.9720	5.6282	12.8061	62.4079	33.4864	18.8930	50.5552	57.0081	123.9613	1.7072	0.6074	0.0749	0.0846	5.7426	23.2189	147.4725	67.3822	67.2830	10.5502	33.2524
TARGET-50-PAKMUB	184.5626	15.4610	20.9770	259.5970	43.4096	35.6780	44.4319	108.3622	216.6731	5.4999	0.9255	0.1025	0.1065	11.8650	45.5511	183.2397	60.1175	191.0642	15.7828	152.7251
TARGET-50-PALKCW	107.0068	8.9967	15.6321	87.1263	26.7998	25.3225	46.8083	74.8997	159.2297	3.4083	0.5258	0.0779	0.0307	7.2908	20.1281	134.1115	48.0417	77.4108	7.5667	45.6341
TARGET-50-CAAAAQ	87.2826	8.1947	11.7011	48.8408	39.0300	16.8933	50.7127	44.7359	133.1967	3.1575	0.3999	0.0505	0.0161	12.9065	24.2209	84.0118	32.6823	31.7507	6.7035	29.3434
TARGET-50-PALFME	117.1498	15.0067	15.6937	65.7711	43.4290	26.2895	51.7508	64.1228	177.6054	5.2022	0.6164	0.0923	0.0340	10.2541	18.3550	99.2266	52.6817	51.5328	8.4492	25.8828
TARGET-50-PAKNXS	138.2338	14.6125	23.0408	110.0290	70.6961	47.7922	49.3920	62.9916	168.5008	7.5337	0.6308	0.1432	0.1242	52.1752	96.8581	137.7982	117.4807	195.3349	14.4118	36.1281
TARGET-50-PAJNAA	170.9975	19.5354	34.2065	238.4599	83.3838	50.7123	43.0669	170.9114	322.9353	12.1155	3.2472	0.6070	0.9042	60.2557	109.1605	324.5921	192.7925	499.1621	29.1923	98.0869
TARGET-50-PAJPGY	168.4868	19.4208	14.4932	79.3939	36.8366	15.3743	48.6210	50.9031	180.7762	8.9077	0.8984	0.0717	0.0442	12.5254	24.7956	152.7656	63.6113	113.9658	10.1775	58.1791
TARGET-50-PAJNEC	149.1655	10.5168	15.0773	65.6848	41.5512	27.8298	48.0895	44.8402	178.6377	8.2205	0.4637	0.0571	0.0434	30.8475	11.3323	255.0083	287.9375	49.4799	13.5298	244.8577
TARGET-50-PAJNDU	94.8822	5.2999	12.2050	41.9127	17.5894	13.7019	41.8489	28.1004	105.0162	2.3615	0.1779	0.0206	0.0157	15.4400	17.8795	125.9188	61.5654	21.4131	8.8981	80.2304
TARGET-50-PAKGZX	77.5483	4.0329	13.3883	218.9532	22.3690	13.8943	48.8554	88.3007	146.3859	2.5818	0.4762	0.0422	0.0651	2.6516	7.3814	132.5325	31.4918	132.8540	12.1771	167.0149
TARGET-50-PAKFYV	118.3319	31.8204	13.4596	57.9892	28.8152	27.9911	50.0950	34.8410	105.7816	16.6988	0.5462	0.1207	0.0484	25.9264	43.2154	116.5561	47.2353	19.0827	9.3237	15.6329
TARGET-50-PAKGED	121.0821	2.3925	17.2946	70.8262	55.7610	15.0250	43.7150	58.5190	176.0669	0.5864	0.2060	0.0193	0.0509	12.7321	20.1370	317.2259	101.3579	89.6397	23.1820	278.7480
TARGET-50-PAJLTI	118.0607	20.1152	19.5679	117.2445	36.5614	23.8943	47.2593	79.3429	206.1929	9.5596	0.8790	0.1215	0.0342	15.1145	33.7352	148.5490	53.2734	111.6802	12.4593	58.2706
TARGET-50-CAAAAO	119.0572	9.3534	15.8289	70.8018	32.5487	21.5759	47.8720	59.0145	168.2690	4.3046	0.5279	0.0720	0.0370	8.7905	21.8259	120.6091	60.1701	68.8997	8.4685	51.8001
TARGET-50-PAJNCZ	117.1838	12.4158	16.6218	92.4901	30.9398	25.2353	49.9887	81.3901	175.1045	5.5459	0.4701	0.0679	0.0321	11.3086	34.6597	122.3065	43.4275	45.7441	9.3026	38.7051
TARGET-50-PAJMIZ	141.5885	18.0071	17.0735	93.4442	35.0780	28.2523	51.7930	67.1041	167.2904	10.7533	0.7536	0.1088	0.0546	16.2073	25.2730	160.9204	90.3477	52.8510	10.3607	121.7322
TARGET-50-PAJMFY	104.2254	11.5072	16.2101	73.3068	35.7354	24.7704	52.0799	60.1818	156.3358	4.9636	0.5578	0.0883	0.0371	13.4101	35.9145	127.6679	54.9407	48.3718	8.7977	37.5832
TARGET-50-PAKULH	143.9420	24.8228	13.1512	74.4990	57.1970	24.5963	55.5322	51.8819	180.0694	9.5082	0.6008	0.0623	0.0224	7.4549	15.1107	114.2658	46.2832	60.7259	6.7074	33.2493
TARGET-50-PAKVET	124.8583	14.6170	22.7872	171.2538	31.6258	41.5856	47.4367	137.5800	243.8133	5.8186	0.9358	0.4021	0.3525	43.9580	120.9649	247.1069	112.2024	138.5193	18.0745	53.2611
TARGET-50-PAJMEN	111.7065	11.1466	14.8562	67.6192	29.9704	21.1559	48.4022	50.8545	137.5930	3.3246	0.4247	0.0624	0.0236	12.8772	45.2215	149.2625	63.6779	46.4858	10.5801	42.3009
TARGET-50-PALDTE	114.4104	15.9208	16.7972	88.3452	43.6346	27.4703	51.9022	71.7294	205.0843	7.7265	0.5590	0.0953	0.0376	14.1998	33.9484	137.5279	76.6513	73.1035	8.9629	38.7013
TARGET-50-CAAAAH	139.0571	8.9221	15.3867	52.0124	42.0925	22.9558	45.1996	36.2679	121.9122	3.2225	0.3051	0.0460	0.0226	20.2409	19.6823	178.0523	81.8505	22.6416	18.9668	89.4517
TARGET-50-PAJNRH	118.5475	18.3543	16.1492	88.4282	30.8798	30.2410	47.6909	61.3453	151.8802	7.0462	0.5780	0.1335	0.0717	13.7415	20.6212	108.8296	44.1913	112.1648	9.9241	42.4530
TARGET-50-PAJNRL	95.3746	8.9583	13.8264	65.2855	48.3973	25.0469	54.3887	44.5816	146.3137	4.7457	0.5667	0.0603	0.0197	5.5104	12.9420	107.4748	51.5737	54.4102	8.9269	21.5578
TARGET-50-PAJNUP	165.7951	11.7441	27.7833	227.8927	73.1945	34.0034	42.5439	133.0565	308.1281	8.5869	2.7863	0.2942	0.4101	26.5655	45.0365	270.8510	120.2855	439.9766	21.8644	177.4373
TARGET-50-PAJPDC	81.2324	8.8405	11.5777	117.1143	45.3768	17.7712	49.3330	57.4868	138.8086	5.9245	1.1992	0.1263	0.0455	6.1227	7.2160	147.8088	53.7768	63.5693	8.0251	62.9355
TARGET-50-PAJMLI	107.1083	11.9786	15.0723	95.7440	40.4752	20.2494	49.7603	68.1638	160.6429	6.5107	0.6397	0.0732	0.0269	16.9014	37.9673	137.6231	54.9503	42.7305	11.3035	49.3714
TARGET-50-PAJLWT	128.2417	11.7091	17.4806	70.7555	34.5842	25.5959	45.5943	56.1567	167.9217	5.0223	0.5416	0.0784	0.0237	12.6847	22.9051	119.5055	56.5233	77.0652	7.4752	34.4194
TARGET-50-PALEZT	116.3295	24.0324	14.0115	86.0765	37.1665	35.7196	47.5848	54.0036	136.7566	8.1261	0.5642	0.0788	0.0406	24.1974	51.6261	135.9103	64.9089	43.6117	9.1389	49.1714
TARGET-50-PAJLLF	181.3870	23.8955	26.4257	117.1199	117.6757	35.3542	56.1691	80.8556	258.5519	22.5902	2.8852	0.4202	0.0500	19.0696	12.6910	184.6264	157.8059	146.6077	15.1431	39.2656
TARGET-50-PAJLNJ	100.1861	6.9430	14.0241	80.0373	26.5624	18.5484	47.5239	62.2641	157.5751	3.4450	0.5331	0.0728	0.0326	8.0156	20.6068	112.5011	37.7701	49.2418	7.0149	61.3728
TARGET-50-PALFRD	86.7100	5.2160	12.9425	66.3799	28.3863	21.5023	46.2798	46.6837	120.9779	2.1549	0.4208	0.0543	0.0229	7.9938	21.4122	119.9767	43.5375	52.3937	9.5586	47.0061
TARGET-50-PAKKSE	106.6706	5.7096	17.1306	117.7646	28.2594	17.5378	44.1789	83.3602	165.5985	3.2712	0.6630	0.0690	0.0274	8.3139	23.5615	140.8135	45.6997	91.0050	12.2493	69.7234
TARGET-50-PAJLPX	156.3148	32.3906	19.6174	78.8068	59.0656	27.5227	51.1910	37.6997	189.6492	16.4131	1.0893	0.2210	0.1429	32.6315	36.2729	138.0547	99.4993	75.2744	18.8963	41.7466
TARGET-50-PAJMKJ	162.5940	30.5119	15.6387	102.0442	62.0275	27.3442	48.1432	82.4207	210.3309	11.7971	0.7986	0.1042	0.0788	10.3069	20.4286	166.8046	91.0503	139.3424	9.9967	61.1817
TARGET-50-PAKSDG	89.8899	8.8603	14.2424	67.8569	30.7044	23.1013	50.4139	56.2905	161.5923	3.5852	0.5208	0.0862	0.0256	12.7280	34.3532	106.4034	64.5756	70.5445	9.2054	33.1656
TARGET-50-PAKZFK	163.4804	19.1080	18.3609	115.6934	57.2708	28.8735	50.7304	103.4417	184.5265	9.5672	1.0653	0.1148	0.0762	14.8274	48.2146	188.7104	70.8524	104.4949	11.7713	73.7917
TARGET-50-PAKYFC	130.0632	18.1527	19.0371	116.2771	33.7060	22.5928	47.5357	81.3339	167.9165	8.6765	0.8140	0.1162	0.0564	11.7474	38.6033	137.7443	38.8254	70.5709	13.8315	54.9931
TARGET-50-PAJNNR	169.3045	16.0975	21.4459	101.2548	68.3578	34.5305	50.8438	76.1013	205.1126	8.3364	1.0247	0.1476	0.0299	17.9028	23.0408	159.0045	85.1104	134.0485	13.1723	42.4757
TARGET-50-PAJLIP	213.5210	52.8068	33.0320	147.6963	90.3065	70.0181	54.2574	139.4358	274.5182	19.6764	1.9032	0.2515	0.2304	111.5290	140.2842	244.2445	189.7030	278.3084	19.6775	35.2756
TARGET-50-PAJNYT	133.8568	19.2820	19.8487	188.6489	55.9877	24.4448	42.3872	97.3641	222.8355	9.1727	1.0568	0.1705	0.0861	23.4882	49.9459	170.0807	60.1311	124.7956	13.9359	59.1869
TARGET-50-PALGVY	105.9742	4.7852	16.5172	80.5145	34.2989	20.1301	45.8555	89.5342	170.6542	1.8071	0.5145	0.0784	0.0418	5.5626	17.4174	137.6225	59.7909	60.6943	10.3261	54.1285
TARGET-50-PAECJB	77.3393	6.8088	12.7408	76.8996	31.8878	19.5446	46.8027	46.3567	121.6748	3.1791	0.4757	0.0708	0.0242	8.8559	24.8005	104.0344	41.3309	30.0221	9.2828	35.8602
TARGET-50-CAAAAM	84.5036	2.9596	14.9573	102.3778	53.2331	10.7327	32.9241	61.0090	222.7266	1.1664	2.0615	0.1401	0.0797	30.2477	164.7565	330.5676	53.1411	206.8767	27.2885	114.3520
TARGET-50-PAKSCC	97.6563	8.7783	13.0141	54.9988	29.5791	20.2852	47.9757	32.8898	137.9605	5.2004	0.4764	0.0620	0.0275	17.1700	28.1288	94.4580	41.3380	29.3057	6.1521	40.2110
TARGET-50-PAKRZW	114.6656	8.3858	15.5896	100.0228	29.0507	16.6623	41.0512	69.4478	186.4609	4.0239	0.7254	0.0766	0.0544	14.2552	51.4484	161.7904	36.8976	101.2236	9.9378	63.6358
TARGET-50-PAJNSL	87.7242	8.4537	14.6549	71.4905	30.6278	25.0866	47.7136	50.1805	139.5379	4.2169	0.5682	0.0967	0.0364	14.3608	49.1840	125.6212	55.3988	33.0624	9.9361	31.8844
TARGET-50-PAKKNS	135.6827	15.4547	21.1826	123.4837	49.2407	36.7368	47.9730	78.5778	215.7806	6.8812	0.8774	0.1244	0.0618	11.4290	24.3722	178.9511	81.2616	162.5385	11.4759	60.7833
TARGET-50-CAAAAL	119.5073	10.5120	21.3896	109.6862	37.0815	32.5762	53.5070	103.0602	205.8377	5.5519	0.6075	0.1346	0.0514	12.3825	27.3392	131.1274	75.8111	83.5707	10.2550	43.8881
TARGET-50-CAAAAJ	117.4595	7.5586	16.9052	82.8325	35.5477	19.8196	49.9547	73.8419	181.0542	3.5104	0.5646	0.0759	0.0332	10.2855	19.0238	114.5075	51.2351	51.6834	8.0879	48.2099
TARGET-50-PAKNAL	100.3340	14.5335	17.7765	167.6998	73.3858	29.4270	48.7911	93.6172	212.9218	6.6820	1.3227	0.1746	0.0402	22.0939	38.6844	187.3254	91.3638	111.3127	11.9270	69.3413
TARGET-50-PAKXXF	109.3816	8.3937	15.2012	50.9189	49.2696	29.1899	43.5100	30.7570	115.2363	3.7380	0.4160	0.0786	0.0260	25.0891	30.0117	93.8782	65.1675	22.2207	13.1326	32.3111
TARGET-50-PAKECR	164.5375	16.1004	20.5555	110.8358	61.6361	19.1220	46.3947	74.2626	216.7784	7.1041	1.3172	0.1501	0.0785	15.3614	32.5793	174.1819	53.3179	211.1293	14.6667	65.6124
TARGET-50-CAAAAP	117.5997	17.4791	17.3291	99.2445	58.1171	25.6577	52.0835	57.1323	183.1351	7.2377	0.8496	0.0951	0.0429	14.4729	27.6702	140.6813	64.7947	50.4445	8.8931	40.1894
TARGET-50-PAEAFB	125.7758	13.5807	19.5177	80.2627	43.9699	23.3298	48.2574	82.8182	182.9111	7.9517	1.0640	0.2357	0.0385	15.1753	28.5296	141.0256	60.8197	198.1114	13.0035	44.9036
TARGET-50-PAJLKR	141.6435	8.5586	19.6940	104.5700	74.9931	29.1681	52.3154	72.8423	269.0623	3.0333	0.8213	0.0761	0.0276	4.9835	5.0502	148.7185	78.4111	82.1153	10.9065	39.9784
TARGET-50-PAKMSV	82.4675	7.6852	11.4603	50.7173	26.1226	15.2208	44.4702	33.6689	125.4969	4.0244	0.3449	0.0404	0.0145	12.3096	20.3480	82.8905	33.1373	30.8050	5.9194	55.5215
TARGET-50-PALLFB	185.6194	32.2176	21.4294	77.9782	64.0602	55.3880	55.3476	54.2998	213.5406	11.3086	0.5968	0.0866	0.0690	31.5210	57.9626	109.7781	75.3169	78.1454	12.9913	19.9800

Supplementary table 1 continue6

	ULK1_4989_1733	VSP34_8731_1734	Selumetinib_1736	IGF1R_3801_1738	JAK_8517_1739	AZD4547_1786	Ibrutinib_1799	Zoledronate_1802	Acetalax_1804	Oxaliplatin_1806	Carmustine_1807	Topotecan_1808	Teniposide_1809	Mitoxantrone_1810	Dactinomycin_1811	Fludarabine_1813	Nelarabine_1814	Fulvestrant_1816	Vincristine_1818	Docetaxel_1819
TARGET-50-PAJNAV	7.7216	6.9232	63.1426	5.9479	38.3908	34.0165	68.1485	88.8419	63.4965	184.7205	1315.6844	2.3672	1.6100	1.9878	0.1060	92.1621	516.9854	112.0213	0.3528	0.1550
TARGET-50-PAJPEW	7.4014	15.0116	55.0305	10.4238	31.9884	26.2773	170.9988	59.4153	77.0439	115.7546	721.3462	1.3847	2.6711	2.9908	0.0649	180.0623	779.2943	121.9905	0.2346	0.0700
TARGET-50-PAJMLZ	13.9702	15.9033	144.9371	14.9411	37.6564	23.6802	110.2676	50.8956	214.4716	400.3994	573.3115	1.1571	2.5706	2.4499	0.2094	215.1432	437.6119	115.8400	0.2570	0.3028
TARGET-50-PAKRVH	14.4383	11.3675	111.6225	7.8014	20.8865	16.6519	91.0219	48.2270	223.8206	187.6079	474.3122	1.8113	2.4663	1.6965	0.0993	208.6437	577.5418	144.8718	0.4101	0.2178
TARGET-50-PADZUB	7.2626	6.5015	24.3519	3.1807	15.5909	22.7963	181.3544	53.5264	85.2197	73.2502	416.3393	1.3913	2.3876	3.7295	0.1017	140.1174	499.3318	105.4423	0.2264	0.0747
TARGET-50-PADXAY	3.5765	7.7537	275.1682	10.3279	54.8712	30.1163	337.2636	76.8450	672.5820	288.9754	924.1736	2.3201	2.1570	4.3115	0.1149	166.0952	712.1354	132.4837	0.1730	0.2022
TARGET-50-PAJPCM	29.0176	16.8876	71.2680	9.7915	36.5165	17.4145	92.4548	41.7252	192.6736	203.1240	226.2500	1.1615	1.5365	4.5327	0.1050	96.4523	314.7192	95.8384	0.1177	0.0533
TARGET-50-PAJPAR	27.4075	17.5735	244.3912	11.9857	39.9355	24.1886	65.7439	52.6881	157.0023	258.2561	538.4837	2.1166	2.7342	5.3533	0.2741	317.2254	478.5929	76.3038	0.3594	0.1329
TARGET-50-PAJNZS	17.9004	18.7487	55.8465	12.6144	30.2091	19.5684	111.8905	51.6431	177.2876	266.7210	537.1903	1.2704	3.5963	4.0079	0.1973	371.3809	586.0926	115.4383	0.8035	0.3449
TARGET-50-PAJMVC	3.0157	4.9181	158.2106	7.0977	26.7295	13.1611	49.3029	50.6372	70.7843	175.9354	443.5436	0.7073	1.1483	1.2271	0.0484	52.3953	344.8848	69.9834	0.1007	0.0340
TARGET-50-PALGAZ	21.1692	13.4895	38.7922	4.8724	18.9304	18.7161	80.0778	46.3223	146.8898	271.2532	446.7534	1.8190	2.4587	2.0735	0.1416	196.4435	414.4522	108.5958	0.2899	0.1242
TARGET-50-CAAAAR	15.2023	13.8224	52.1704	3.5931	13.0415	16.7300	72.6379	35.9478	84.6486	171.7622	405.8280	1.3407	1.7740	1.3040	0.0793	121.9750	286.0671	114.7759	0.1164	0.0426
TARGET-50-PAKGMU	8.1114	14.4524	61.8652	5.7897	23.7331	18.8107	147.5068	58.7259	177.0564	187.9698	785.1000	1.1005	2.7457	2.9717	0.1429	233.4489	646.7166	124.7461	0.2821	0.1154
TARGET-50-PAKRCC	15.1478	12.5392	72.9997	4.6352	21.7766	16.0094	65.6913	29.4710	111.4493	106.3472	289.2030	2.5092	2.3527	2.7185	0.1006	142.3801	247.1008	65.1344	0.0809	0.0855
TARGET-50-PAKFME	8.5071	10.2412	54.8290	4.1392	16.5999	18.2694	143.2953	41.4409	137.6231	139.9411	433.1036	1.0934	1.8895	1.5019	0.0820	138.4894	397.0841	110.1687	0.1211	0.0684
TARGET-50-PAJNNC	28.1011	10.8813	36.9030	4.0084	20.6292	21.8022	68.7233	55.6516	76.3964	164.2479	439.2433	0.6680	1.5731	1.2022	0.1160	235.5344	408.3117	102.9646	0.2742	0.2050
TARGET-50-PAJNJJ	17.4266	18.2871	83.8007	11.1096	28.9203	22.5721	142.0130	77.8145	185.8163	224.0090	636.6727	0.8324	2.5506	2.6434	0.1845	200.0727	669.4034	133.8724	0.5555	0.6401
TARGET-50-PAKJGM	2.1967	3.9395	34.6499	2.6472	12.1265	18.4942	58.5151	33.2069	134.7365	51.2443	287.8846	0.4329	0.4076	1.2511	0.0293	54.7234	279.1008	44.5386	0.0537	0.0209
TARGET-50-PAJNGH	12.0281	11.5570	57.6546	5.1095	12.5129	16.5901	47.6930	39.1615	167.6957	285.2472	323.6416	1.0195	1.4238	1.7428	0.1082	216.4613	272.0908	82.1795	0.3284	0.2190
TARGET-50-CAAAAC	8.7361	9.8962	91.7647	11.7357	22.8807	16.7064	68.5398	31.6205	59.7348	212.2235	341.2941	0.9123	2.0715	1.4159	0.1438	106.9251	344.4677	77.8119	0.2094	1.9553
TARGET-50-PAJNBN	12.7027	8.2638	80.3323	2.4892	10.0490	11.0529	38.1800	30.1316	140.5506	104.4098	200.4909	0.7933	1.0025	1.5612	0.0559	93.8629	193.0698	51.9707	0.0849	0.0406
TARGET-50-PAKYLT	11.0903	11.7683	220.2214	5.2413	22.5661	16.7450	52.6009	30.6178	56.0190	417.5033	563.3310	3.6726	3.2716	2.9169	0.1645	136.6780	449.5992	99.9350	0.0940	0.0953
TARGET-50-PAJMKN	17.1774	7.4408	99.5432	12.9728	32.2878	28.7927	43.1827	61.1147	100.5809	185.9473	579.3507	1.3573	2.1075	2.3010	0.1090	208.9689	581.7584	157.2638	0.4630	0.1656
TARGET-50-PALERC	17.8645	8.5279	63.4054	5.9587	17.8203	12.9743	41.9903	36.9552	76.1390	133.4679	313.0220	0.6588	1.0722	1.2243	0.0676	138.6948	322.6879	72.9900	0.1125	0.0379
TARGET-50-PAJMFU	9.3235	9.6483	54.9639	6.2336	15.4494	13.7082	99.4882	38.8326	211.9001	125.2332	350.2690	0.8562	1.6457	2.3436	0.0732	117.4140	383.2614	100.9956	0.2175	0.1192
TARGET-50-PAKZHF	5.2015	7.4087	29.6343	3.3199	16.9055	19.9653	101.7988	46.0265	99.9070	67.0045	484.3344	0.4613	0.7182	0.6858	0.0488	207.7033	673.3029	119.1908	0.1438	0.0621
TARGET-50-PAJLSP	6.3746	12.1489	148.3047	6.9594	21.8162	12.3323	87.1490	40.1598	125.1147	136.0835	340.9398	0.6155	0.6730	0.6602	0.1036	105.4540	396.9401	84.4607	0.1143	0.1661
TARGET-50-PAJMKI	12.6795	13.6620	72.0275	3.8326	12.3970	17.7116	103.0733	39.7373	383.6974	218.3712	307.5633	1.3774	2.5491	4.0047	0.1096	234.5252	324.8276	96.9153	0.1832	0.0761
TARGET-50-PAKUIT	44.0926	15.1091	88.1354	11.1657	25.2840	16.7124	124.6388	35.7384	181.9013	171.0957	370.4786	1.0286	2.6794	1.8035	0.1486	166.8236	374.7765	63.7130	0.1585	0.2177
TARGET-50-PALDWP	8.2678	7.0515	37.1116	2.1143	10.0849	11.8884	54.1654	31.3481	219.8779	116.8321	270.5758	0.8259	0.8781	1.1711	0.0619	121.6536	294.823	59.3267	0.0670	0.0284
TARGET-50-PAJMJT	7.3973	8.5793	26.9351	5.3218	16.5403	24.1727	86.5405	42.2411	178.5864	94.5790	367.0828	1.4484	1.4065	1.2665	0.0777	203.9344	400.6721	98.3083	0.1486	0.0910
TARGET-50-PAKWPM	7.5421	10.0790	25.7214	4.6020	14.5845	17.6892	96.9246	47.0445	169.2899	116.2128	522.3330	0.5820	1.1748	1.6868	0.0754	130.4024	405.8148	100.1970	0.1875	0.1594
TARGET-50-PAJLKC	3.8205	12.4863	78.3597	4.1711	14.2876	12.0933	59.9990	26.5035	52.8600	111.7143	324.3409	0.5314	0.3666	0.7502	0.0670	150.8116	376.9941	82.5875	0.0561	0.0627
TARGET-50-PALKRS	2.3652	6.6980	77.0787	5.1151	35.7501	22.2833	143.5039	60.6297	153.2176	107.8888	711.9040	0.6723	0.9801	1.1203	0.0337	65.9838	553.703	107.8768	0.0537	0.0298
TARGET-50-PAKNRX	26.6381	15.4521	136.4428	27.8691	77.9239	32.2686	123.1135	71.7765	123.6496	326.0157	643.7164	3.0250	4.3556	9.6244	0.3519	338.3000	626.6842	186.9111	2.2768	1.5872
TARGET-50-PAJNTJ	9.3480	13.0068	73.6059	8.4248	19.8512	27.1389	59.5968	38.3573	183.4785	178.2067	543.8691	1.0068	0.9268	1.0732	0.0925	291.5394	466.571	114.1350	0.3244	0.1293
TARGET-50-PAJNUS	7.2286	9.0608	50.2211	3.1053	11.3597	19.0993	77.9812	40.5305	119.3141	106.6590	355.4890	1.2459	1.5896	1.5589	0.0743	190.1513	390.5732	93.2478	0.1145	0.0557
TARGET-50-PAKNTW	3.2964	4.1876	24.0907	1.1459	10.2289	11.3207	133.9541	29.6823	227.9315	15.9863	190.2230	0.4409	0.6883	0.9653	0.0175	79.4863	322.9742	64.7040	0.0362	0.0114
TARGET-50-PAJPAU	17.6140	9.5437	47.7739	4.6319	17.9999	21.6890	52.4780	36.8059	82.6596	123.8962	322.4310	0.6033	0.5126	0.7557	0.0865	182.8515	370.3434	78.8044	0.2615	0.1848
TARGET-50-PAJMVU	10.5134	18.6755	176.8091	9.1699	20.8045	27.2257	170.0618	60.0703	183.3892	173.7464	608.8828	1.0475	1.9827	2.5081	0.1115	174.1730	748.2058	113.9565	0.1684	0.0726
TARGET-50-PAJNZI	19.5380	11.2400	88.0127	3.7841	14.3477	16.1123	41.0014	24.4002	85.1530	93.3021	217.4399	1.4271	1.6167	2.5339	0.0820	117.6884	212.4458	46.9775	0.0515	0.0337
TARGET-50-PAJPDN	8.1680	7.5681	102.8966	10.1433	43.8367	19.5959	120.2542	45.5153	184.7915	128.5497	482.1719	0.5732	0.8077	1.1647	0.0301	98.8485	494.1834	92.6106	0.1132	0.0513
TARGET-50-PAJNVE	7.6303	7.2783	142.7525	4.0063	10.0721	10.6300	41.9123	28.8771	110.7280	128.6738	268.9594	0.4572	0.9168	0.8033	0.1133	132.3662	239.1124	49.5297	0.0513	0.0226
TARGET-50-PAJMSE	16.9734	10.2434	62.3363	4.4863	12.3188	17.1356	73.0882	38.0042	136.9341	133.3424	453.0553	0.9281	0.8733	0.8663	0.0864	209.9033	345.8702	83.5570	0.1036	0.0925
TARGET-50-PAJNZU	21.8556	13.2616	45.8220	5.0215	14.1619	21.6065	70.4453	37.9328	144.5996	253.7587	357.5655	1.3033	2.4415	1.7791	0.1152	145.3447	291.3978	87.9975	0.1973	0.0766
TARGET-50-PALGLU	8.1519	8.8815	51.6841	4.4514	21.7546	15.2865	87.0492	45.3642	55.9613	70.2530	460.3451	0.6645	1.2291	0.9366	0.0571	101.6257	352.3705	90.4322	0.0872	0.0416
TARGET-50-PAJNZK	17.1944	12.6169	48.4447	5.0126	12.8682	14.4136	83.6331	33.3365	225.8583	181.7334	315.5285	1.3637	0.9635	0.8003	0.1010	114.6301	255.4445	56.1643	0.1321	0.0937
TARGET-50-PAJMUF	6.9240	7.8812	76.2093	2.4387	11.3639	11.7613	62.1615	29.3149	101.8948	153.8728	362.1445	0.7175	1.1513	1.4324	0.0401	92.7231	301.1707	69.4256	0.0412	0.0165
TARGET-50-PAJMRL	9.3761	17.2736	180.4462	6.8168	24.7574	11.9416	112.8664	39.3792	186.3131	282.1445	393.8518	0.9335	0.9426	3.8362	0.0978	200.6808	454.9582	127.7810	0.0939	0.0372
TARGET-50-PAJMXF	6.1065	6.6523	55.7717	5.6275	17.3256	20.0746	38.9635	36.2181	112.9621	116.4529	354.7186	1.1495	1.3139	1.5848	0.0829	156.2873	299.9731	70.1619	0.0920	0.0565
TARGET-50-PAKPDF	22.4866	14.1844	70.6066	3.3331	10.3859	8.4136	38.9228	27.1791	73.9292	131.3837	246.5204	0.8638	1.2718	1.3220	0.0825	190.6014	258.6803	82.9751	0.0863	0.0824
TARGET-50-PAEBXA	11.6814	13.5581	78.7755	5.9668	21.6725	14.0897	109.0991	52.3916	95.8809	126.8456	590.3204	1.2089	2.0538	1.7439	0.0784	195.9666	590.6625	99.8978	0.3531	0.1692
TARGET-50-CAAAAS	20.2795	17.0614	45.6329	6.7925	14.3759	26.1093	69.1533	50.3009	135.9550	253.2785	435.3544	1.8011	2.3272	1.7197	0.3173	187.4866	346.6225	107.6101	0.3310	0.9204
TARGET-50-PAJNLT	12.0340	14.0836	130.2374	7.8699	21.1375	19.8110	84.7189	56.4934	151.8164	172.3303	379.8949	0.6145	0.8521	1.2802	0.0859	164.0562	365.0544	82.5175	0.0854	0.0451
TARGET-50-PAJPHA	8.3340	10.4416	66.2639	4.7199	14.5259	15.1117	64.2136	49.3502	159.1157	190.9945	491.8414	0.8146	1.8303	1.3765	0.0919	191.9679	379.8534	96.0174	0.2511	0.0881
TARGET-50-PAJMMY	5.7061	8.0066	65.0745	2.0408	10.5907	19.0689	30.5055	29.4476	117.7562	145.5994	290.6956	1.5618	1.2562	1.1473	0.0841	164.8900	162.5311	64.4585	0.0595	0.0351
TARGET-50-PAJLTH	7.4418	9.7943	17.7419	3.7426	14.6495	13.2302	74.0448	37.0528	114.4058	58.3084	357.5472	0.5919	1.0185	0.9035	0.0567	135.5842	457.5504	70.9112	0.1012	0.0638
TARGET-50-PAJNCC	13.7078	13.0170	32.0270	3.1856	11.3051	16.9954	61.0150	57.3457	109.7540	49.4815	471.8622	0.4019	0.9239	0.8186	0.0640	205.9710	537.707	53.4752	0.0641	0.1006
TARGET-50-PAJLUJ	2.8167	4.2653	24.3817	1.7151	7.3322	12.0603	93.4935	33.0099	69.9558	48.8245	489.3573	0.5289	0.6683	0.6866	0.0319	84.1017	295.1635	72.4468	0.1027	0.0336
TARGET-50-PAJNCJ	27.2174	16.2184	60.8166	5.6808	16.6617	15.5514	85.2412	39.3837	128.1135	179.4780	337.0805	1.4502	2.4081	1.7931	0.1013	225.1888	393.9962	110.2465	0.2314	0.1185
TARGET-50-PAKZER	9.1060	19.7918	92.1409	3.2594	20.7758	15.4319	108.7777	48.1294	39.3950	123.2679	582.2910	0.5330	1.9772	1.3913	0.0682	123.2101	472.462	112.1366	0.1935	0.1522
TARGET-50-PAJMEP	10.3603	11.9600	81.0635	3.4226	12.4992	14.8157	54.8242	35.3870	150.5555	139.4514	314.6178	1.2108	1.1502	1.1512	0.0955	175.8099	246.1733	64.0785	0.0929	0.0337
TARGET-50-PAJMEL	13.7604	13.6264	48.4143	3.0608	12.6373	17.0740	97.4213	38.0982	70.6698	193.3621	440.7308	2.0238	3.0731	1.9855	0.0976	159.7579	344.4797	132.3265	0.1091	0.0628
TARGET-50-PAJNVX	6.5861	22.3958	146.5285	12.2420	49.5315	17.4536	231.6550	51.4323	99.9610	1227.591	1149.317	35.8295	51.6761	15.3616	0.4334	118.6151	656.1271	314.8993	2.1928	12.9411
TARGET-50-PAKXWB	3.6260	8.3687	35.6119	3.4652	14.7333	16.6900	82.2131	40.6020	97.8865	138.896	639.483	1.6716	3.0866	2.5871	0.0972	126.7903	395.2801	88.4881	0.4681	0.3213
TARGET-50-CAAAAB	4.3254	11.5530	55.9468	5.3089	15.7570	14.7105	45.3296	34.1143	117.4123	123.0812	356.6214	0.7401	1.4314	1.5989	0.1159	172.9495	341.8521	72.6107	0.1356	0.1282
TARGET-50-PALJIP	8.5193	9.1181	24.8107	3.7522	20.0338	19.9874	61.1506	38.6853	71.4388	149.3663	360.0901	1.2760	2.2245	2.2608	0.0984	195.5465	404.4859	117.9618	0.1285	0.0797
TARGET-50-PAKMUB	9.2876	13.5350	56.2214	4.0717	15.2859	23.7839	149.6126	62.4823	89.1692	262.8612	1306.531	1.4031	3.5063	2.0221	0.1895	267.0938	774.8413	279.4296	0.8418	0.7431
TARGET-50-PALKCW	7.4558	7.1324	36.0263	4.6788	16.5045	12.7904	77.0522	39.4154	89.8126	114.6474	469.7726	1.1174	1.7870	2.1892	0.0572	184.3252	453.3509	107.5168	0.1907	0.0851
TARGET-50-CAAAAQ	6.2434	6.8469	68.0261	2.3350	10.8159	12.9451	34.5911	29.8895	82.1572	40.35019	221.1405	0.3663	0.6493	0.6699	0.0418	71.3307	227.3513	48.6550	0.0360	0.0206
TARGET-50-PALFME	12.3462	12.5842	63.2499	3.2490	12.5693	12.6381	123.9997	40.3140	198.3949	99.19197	308.8004	0.5343	1.0548	1.2659	0.0681	141.1103	337.9372	68.2305	0.1215	0.1148
TARGET-50-PAKNXS	13.1198	11.8269	159.8132	9.0434	44.2202	26.3361	76.1371	62.1812	339.7712	276.8524	722.7126	1.3425	3.1258	4.0237	0.1757	266.3992	654.365	138.7816	0.2391	0.0877
TARGET-50-PAJNAA	66.4773	38.5728	285.5913	41.7814	94.7352	49.2968	233.9263	145.1357	306.8611	514.7049	1359.884	6.4844	6.5221	12.1920	0.3919	399.7263	1398.278	501.0830	8.4632	5.5255
TARGET-50-PAJPGY	4.7093	9.8363	47.7382	2.7985	15.7514	21.5171	78.0125	47.9389	247.8991	147.31	594.8476	0.9644	2.1188	1.5539	0.0769	140.1318	320.9058	93.6023	0.1028	0.0919
TARGET-50-PAJNEC	1.6136	9.4500	138.5134	9.4552	126.8477	42.6751	308.5303	66.9124	394.8230	57.10614	528.1573	0.2019	0.2664	0.9039	0.0100	93.4129	642.4728	73.0747	0.0333	0.0454
TARGET-50-PAJNDU	2.2184	3.9166	109.0043	4.4070	31.0042	13.8450	59.5117	45.3321	120.5028	42.58731	399.1781	0.2482	0.1880	0.5123	0.0073	52.5315	391.7068	45.6465	0.0102	0.0032
TARGET-50-PAKGZX	5.7046	9.6924	10.8890	1.4748	4.3510	13.0306	146.0993	39.6940	147.0288	105.2145	460.9206	1.3288	1.4553	1.0510	0.0703	108.2268	292.0079	123.0051	0.3633	0.1693
TARGET-50-PAKFYV	25.9760	9.4953	105.0111	5.8533	11.0927	8.5512	42.3590	38.6907	307.8315	222.786	456.2976	3.0842	3.9644	3.2023	0.1420	177.3349	237.8084	67.9065	0.1635	0.1359
TARGET-50-PAKGED	3.3988	7.6256	86.0784	17.2932	233.5018	54.9860	364.7783	91.6391	472.2546	97.75398	806.4381	0.5740	0.3713	1.4886	0.0132	137.1618	891.1671	53.6419	0.0180	0.0056
TARGET-50-PAJLTI	14.2537	15.5133	97.6139	4.6001	13.5876	17.6864	108.9653	45.6765	123.8099	230.3318	548.6161	1.0382	0.9251	0.8154	0.1070	191.9644	437.8039	99.3929	0.1892	0.0798
TARGET-50-CAAAAO	6.7489	6.5564	48.4710	4.9331	20.3484	14.1191	83.1805	39.9826	143.3990	89.60038	391.7915	0.6258	1.1636	1.5446	0.0483	128.7268	462.4005	76.5019	0.1205	0.0518
TARGET-50-PAJNCZ	12.9888	9.6127	56.4560	4.1715	11.7912	12.6399	58.9451	36.0872	108.9778	163.0199	322.9699	1.1830	2.0128	1.9594	0.0741	154.8627	283.3567	96.3737	0.1492	0.0755
TARGET-50-PAJMIZ	5.2004	8.2784	81.6166	5.3720	22.8478	17.1049	122.7380	47.0181	225.2372	121.3327	538.0711	0.5258	0.9317	1.2546	0.0586	183.0929	562.2711	127.7069	0.2757	0.1514
TARGET-50-PAJMFY	14.5815	10.5631	80.9374	5.3697	13.3322	11.3007	60.5719	37.6007	168.4794	144.5841	342.2152	1.0428	1.6196	1.5802	0.1048	236.1126	389.1677	79.8262	0.1722	0.1032
TARGET-50-PAKULH	3.5695	8.2059	26.0866	2.6551	13.3611	13.6524	90.1323	32.0637	107.6689	104.7787	421.5981	0.7245	0.6374	0.8469	0.0623	62.9266	269.1424	54.5430	0.0514	0.1105
TARGET-50-PAKVET	61.9153	11.0301	124.9909	17.0773	53.5668	56.4146	47.1467	69.4824	107.8514	371.1278	573.4798	3.1361	3.1911	4.3186	0.2572	366.5427	597.1283	227.1777	2.4832	1.1785
TARGET-50-PAJMEN	10.2724	7.1557	77.1717	7.9399	26.8965	14.7602	101.3037	39.8841	127.9204	115.2876	439.7899	0.7534	1.1797	1.4801	0.0470	176.1395	374.7813	69.7514	0.0876	0.0258
TARGET-50-PALDTE	15.1666	10.8396	83.8366	5.7148	17.8388	18.0603	85.2208	36.7732	205.2279	177.127	342.404	1.0517	1.4675	1.5085	0.0860	180.3257	325.3429	89.7161	0.1252	0.0838
TARGET-50-CAAAAH	3.9584	9.8290	109.4131	6.9661	45.1176	21.6844	263.0131	56.4676	465.0312	98.48794	498.5732	0.2451	0.3678	0.9024	0.0193	188.7500	761.7782	60.0768	0.0147	0.0064
TARGET-50-PAJNRH	8.8278	5.5360	47.0045	4.8319	19.6022	17.2089	52.5724	38.7104	39.9980	99.53353	435.828	0.8081	1.2213	3.4508	0.0512	129.4772	411.1041	65.9612	0.4280	0.1668
TARGET-50-PAJNRL	5.5405	7.6941	31.2012	2.2491	9.9916	16.2471	72.3193	28.8620	112.4714	63.72798	322.5844	0.6142	0.7065	1.3371	0.0583	104.3998	232.8541	60.5213	0.1148	0.0667
TARGET-50-PAJNUP	10.5489	25.2187	95.3009	10.1426	51.4265	36.9109	393.5443	75.5830	235.7166	696.593	1541.426	5.1751	12.7861	5.2157	0.2819	312.8574	1410.884	246.1145	1.5859	1.2296
TARGET-50-PAJPDC	9.1131	10.3685	5.5228	2.9084	12.1386	26.9606	78.5198	29.6369	135.3157	79.52399	471.1769	1.8132	2.0149	1.6392	0.1049	43.4588	238.1567	103.3270	0.4574	0.8453
TARGET-50-PAJMLI	9.3607	12.0452	81.9243	3.8816	13.1058	12.0673	78.2282	37.9116	102.9920	174.5909	405.3123	1.2508	1.6005	1.2103	0.0785	171.3933	332.3165	91.7855	0.0863	0.0430
TARGET-50-PAJLWT	4.7250	6.9704	59.8457	7.1354	17.7653	13.5371	52.8798	41.4946	32.7866	82.42467	479.5047	0.8797	1.8349	3.2615	0.0562	156.6262	444.1833	71.4702	0.1165	0.0497
TARGET-50-PALEZT	4.7563	6.8195	76.9360	11.7650	25.2001	12.4036	98.5081	32.0644	177.3491	181.4444	427.8611	0.8081	1.6968	1.9749	0.0724	130.4554	305.475	94.4713	0.3254	0.2436
TARGET-50-PAJLLF	14.2261	22.9082	74.4201	8.7143	35.4565	25.7882	451.4431	41.0407	436.5261	190.0225	605.219	1.5027	1.6807	3.9294	0.1604	229.9755	543.0174	118.6601	0.3858	0.0987
TARGET-50-PAJLNJ	6.2252	7.4508	33.6612	3.2358	12.5800	12.9668	92.7406	35.6785	91.5171	88.19059	349.1327	0.4908	1.0051	0.8739	0.0446	96.9092	344.6048	76.5587	0.1047	0.0367
TARGET-50-PALFRD	3.5196	6.1662	42.1874	4.9911	20.0795	12.0153	84.5879	36.9245	99.8690	75.33092	406.8454	0.8622	1.2808	1.8688	0.0459	130.9686	387.3297	81.1819	0.1000	0.0435
TARGET-50-PAKKSE	7.1824	12.1637	42.9120	2.3399	10.5514	15.5779	95.0616	38.9749	68.7602	148.1333	462.0621	1.5903	2.3342	1.7622	0.0721	137.5712	386.9209	93.9660	0.1114	0.0416
TARGET-50-PAJLPX	31.6012	21.2060	81.1497	6.0828	24.7867	17.7817	166.1036	50.6939	191.6869	225.1325	475.332	1.4158	2.0790	1.6170	0.1318	207.1489	424.9255	106.5522	0.2784	0.1880
TARGET-50-PAJMKJ	12.4830	11.1508	44.0436	4.9878	26.1409	26.5809	106.2361	41.2764	103.0764	131.6946	626.7537	2.1119	1.8745	1.4957	0.0829	112.0596	413.4037	105.5002	0.2095	0.8632
TARGET-50-PAKSDG	17.0058	12.3916	88.0923	3.4153	14.2556	14.3967	51.7229	42.3214	117.3849	192.2986	372.1319	1.9222	1.8481	2.3177	0.0723	147.4479	270.4707	66.3097	0.0813	0.0320
TARGET-50-PAKZFK	7.6658	11.0828	103.3140	4.8231	17.7672	13.2887	131.0358	45.6379	119.7527	171.7887	521.0055	0.8062	1.5318	1.3909	0.0836	223.8558	635.8524	139.4534	0.2115	0.1239
TARGET-50-PAKYFC	22.5356	16.2300	59.8128	5.2924	14.9854	18.4955	133.4288	43.2935	144.4333	295.1123	456.3578	1.3094	2.6119	1.7398	0.1198	181.5525	346.6667	127.3194	0.3219	0.2313
TARGET-50-PAJNNR	9.2950	19.8128	40.4843	5.9118	15.9798	16.0929	165.5575	46.2932	202.2756	165.3746	504.5547	0.7072	2.1612	1.9147	0.1417	350.8279	603.2859	89.4919	0.1296	0.0335
TARGET-50-PAJLIP	29.9037	25.2426	231.0596	35.5113	65.5652	21.4430	143.0613	70.6769	233.2028	862.1322	812.0657	2.3524	2.2277	5.8726	0.4510	520.2063	725.7388	173.3441	0.5185	0.3526
TARGET-50-PAJNYT	19.9585	19.0808	58.6382	4.8038	15.8310	25.1250	117.7306	38.6753	129.5504	268.0501	530.6708	2.6501	5.3116	2.5431	0.1319	128.8437	378.5667	164.8327	0.5733	0.7491
TARGET-50-PALGVY	8.6686	7.9497	46.9401	4.3959	19.3353	17.5032	138.8574	45.1160	61.5211	69.0004	378.3393	0.7684	1.3997	1.5851	0.0465	130.7451	661.8766	122.6440	0.1157	0.0288
TARGET-50-PAECJB	12.4497	8.5241	49.8283	2.4596	9.0788	11.3201	48.8391	27.9511	72.9622	112.9761	295.897	0.4919	0.9118	0.8137	0.0507	96.9837	254.0335	72.8362	0.0700	0.0318
TARGET-50-CAAAAM	13.1053	13.4998	127.9348	5.6482	90.6155	78.6703	106.7203	34.8386	24.6980	597.8667	864.0123	44.4861	66.2368	7.7141	0.1295	89.9391	777.4417	110.2635	0.2692	0.3491
TARGET-50-PAKSCC	5.0749	5.9003	54.2191	4.6397	13.5349	10.2944	44.2914	34.4087	94.4383	74.67895	399.5368	0.4835	1.0106	1.3533	0.0400	79.0659	287.4724	52.2975	0.0715	0.0328
TARGET-50-PAKRZW	6.1900	8.0467	51.4790	5.7021	27.8304	30.3046	61.9473	36.3729	19.5031	165.461	624.6737	2.0027	4.5369	2.1915	0.0578	75.5281	383.565	87.6093	0.2164	0.1324
TARGET-50-PAJNSL	27.3727	11.5563	70.1048	5.9088	17.1696	15.5469	29.5873	37.7916	72.7381	151.0592	283.2639	0.9022	1.0488	1.2065	0.0755	153.3050	281.2241	71.9098	0.0887	0.0340
TARGET-50-PAKKNS	10.2199	14.5897	33.9300	11.0632	28.6367	20.8755	107.6323	54.9648	229.8607	254.7784	538.4367	2.6385	6.5936	6.1085	0.1468	222.9987	644.039	148.6202	0.5514	0.4740
TARGET-50-CAAAAL	19.9784	13.6603	93.0428	6.6870	17.6904	18.4819	91.5907	44.7926	270.9496	224.2716	340.9283	1.2410	2.0755	2.4922	0.0981	198.5355	423.9493	119.5403	0.2343	0.1125
TARGET-50-CAAAAJ	7.4468	9.7031	43.5735	3.4009	11.9017	13.1927	99.0206	35.5033	128.2907	74.63415	306.7326	0.4607	0.9920	1.0034	0.0456	128.0532	345.3073	85.1542	0.0831	0.0306
TARGET-50-PAKNAL	12.0364	16.0379	61.8538	4.4100	20.6263	17.3498	100.8136	46.7023	129.9233	137.8185	536.2245	2.2370	2.3786	1.7615	0.1064	130.7423	396.1987	129.1544	0.2346	0.3479
TARGET-50-PAKXXF	6.9325	11.4808	129.1878	6.3608	34.0770	16.7103	89.7238	45.6074	203.0760	142.1846	376.3049	0.6269	1.0719	2.0839	0.0604	170.9643	560.9708	73.5918	0.0455	0.0070
TARGET-50-PAKECR	10.4945	17.7727	65.9807	3.4719	25.4066	23.4925	178.2545	46.4392	80.4218	145.4865	787.7541	1.6577	3.4335	1.6674	0.0813	104.5106	605.4572	116.1863	0.2149	0.1962
TARGET-50-CAAAAP	13.0634	13.3290	70.0878	3.0207	10.9211	13.8705	116.6929	34.8103	362.0937	183.2036	543.1082	3.1129	1.5223	1.6713	0.1421	181.1601	393.5124	85.4050	0.1880	0.2077
TARGET-50-PAEAFB	19.2784	16.1230	77.4044	4.0475	16.9150	15.4481	129.4689	42.6478	202.7975	101.5872	438.7658	0.6447	0.9417	1.2616	0.0492	203.4846	480.9214	81.4398	0.1696	0.0468
TARGET-50-PAJLKR	5.1741	9.0713	19.1637	4.5248	23.5920	22.1432	224.4828	61.5746	368.8785	131.2638	498.9687	0.7171	1.0947	1.7524	0.0999	148.5384	487.0164	80.3127	0.1700	0.1448
TARGET-50-PAKMSV	2.3416	4.2791	44.7672	2.3477	10.1099	13.1933	78.1533	33.0560	219.2145	40.63731	284.8454	0.4228	0.3775	1.0046	0.0253	85.7858	318.8308	46.5709	0.0588	0.0237
TARGET-50-PALLFB	12.3252	14.6954	132.0518	6.8389	23.5006	11.4642	91.7967	42.4964	148.0649	271.596	430.3623	0.7759	1.8560	3.0782	0.1912	222.2645	428.9554	101.6579	0.1654	0.1067

Supplementary table 1 continue7

	Podophyllotoxin bromide_1825	Dihydrorotenone_1827	Gallibiscoquinazole_1830	Elephantin_1835	Sinularin_1838	Sabutoclax_1849	LY2109761_1852	OF-1_1853	MN-64_1854	KRAS (G12C) Inhibitor-12_1855	MG-132_1862	BDP-00009066_1866	Buparlisib_1873	Ulixertinib_1908	Venetoclax_1909	ABT737_1910	Dactinomycin_1911	Afuresertib_1912	AGI-5198_1913	AZD3759_1915
TARGET-50-PAJNAV	0.8531	3.2567	16.4623	17.1598	33.8096	0.4309	92.4457	64.6020	87.5515	77.8446	0.3192	11.3019	3.5123	24.4936	7.3067	14.7980	0.0219	11.2518	124.9504	16.0475
TARGET-50-PAJPEW	0.7711	1.7511	17.3890	48.1320	90.4580	0.4200	282.5895	86.7700	287.8716	121.6710	0.1727	13.6980	2.7619	16.7021	16.1107	20.1146	0.0051	24.6929	114.7771	18.6908
TARGET-50-PAJMLZ	0.7350	1.7649	14.1441	30.2808	32.5625	1.0811	125.2014	66.9507	128.9035	108.4347	0.2182	14.5705	4.3080	37.6357	9.5583	9.5707	0.0182	18.3537	103.7688	17.6118
TARGET-50-PAKRVH	0.8184	4.8410	18.9000	48.3741	51.4738	1.0789	238.9331	84.3080	125.8486	142.9945	0.1988	10.8327	3.5492	25.7035	12.1257	14.5953	0.0129	31.6250	132.6859	11.4429
TARGET-50-PADZUB	0.7211	1.3066	18.4078	39.6656	49.8606	1.0343	271.4169	52.5138	155.9052	87.0039	0.1832	10.2280	2.6842	7.7968	8.6209	6.3444	0.0047	22.4143	97.0182	23.3464
TARGET-50-PADXAY	0.4792	4.2065	13.2590	27.1144	75.8268	0.4579	188.0622	67.6362	210.9170	72.2173	0.1516	16.4821	3.3332	34.8609	7.8684	9.2402	0.0180	8.7817	121.1917	23.2439
TARGET-50-PAJPCM	0.4654	1.3452	14.1727	39.5061	25.3718	2.3045	230.6540	37.9743	103.7118	97.0998	0.1262	8.7276	2.1966	8.6961	11.3905	7.8266	0.0138	22.4738	94.5854	9.4653
TARGET-50-PAJPAR	0.8975	0.9462	13.7349	36.8789	29.2619	1.9272	140.7055	30.4089	124.9787	96.9869	0.2021	13.2653	4.0032	22.1522	11.9612	7.9472	0.0325	8.9168	89.1189	12.2186
TARGET-50-PAJNZS	1.0874	2.9933	18.0044	82.5852	51.3270	1.2069	242.9694	99.6913	170.0089	243.8470	0.1559	16.0485	3.5765	19.3172	8.0847	8.7035	0.0176	26.7481	120.8719	14.5575
TARGET-50-PAJMVC	0.4932	3.2968	9.8956	31.0952	32.7867	0.2544	267.9294	50.5891	175.2524	46.2933	0.2306	9.8340	2.3449	17.9980	9.0438	6.8020	0.0084	1.6312	130.1426	7.4055
TARGET-50-PALGAZ	0.6797	3.2587	15.9092	43.0448	32.7417	1.4069	151.1520	67.1166	102.7276	93.4670	0.2025	11.6871	2.5656	11.9388	12.9095	11.2623	0.0102	18.0018	109.2405	16.0432
TARGET-50-CAAAAR	0.3890	2.4213	15.1773	43.0483	28.5942	0.8655	142.0732	66.9162	89.1264	101.4529	0.1858	11.9267	2.2015	12.7424	7.0897	5.4311	0.0068	8.6878	104.0360	10.4196
TARGET-50-PAKGMU	0.7250	3.3323	16.0144	32.6007	57.0514	0.7975	232.5721	72.0346	143.1125	120.7137	0.1926	9.4924	3.8574	19.8104	10.9420	9.2919	0.0146	15.3639	111.0936	18.0829
TARGET-50-PAKRCC	0.4503	0.8744	9.6216	26.1076	19.1609	0.8806	107.9641	26.3761	78.0110	66.5402	0.1637	12.8474	2.9321	13.2193	6.1118	1.4628	0.0122	8.0710	81.5171	15.4145
TARGET-50-PAKFME	0.4503	2.7502	16.3283	40.0520	42.2677	0.6860	174.0540	64.3457	110.8119	95.2712	0.1738	11.8562	2.9062	15.4919	7.2325	3.5377	0.0078	16.4633	95.4808	16.0283
TARGET-50-PAJNNC	0.4730	2.1235	14.8289	23.9809	35.3416	0.5056	163.1817	60.8074	107.1787	98.5524	0.1998	12.0005	1.9913	14.7487	9.5596	15.8747	0.0077	11.4575	114.8080	13.0693
TARGET-50-PAJNJJ	1.7180	1.7565	18.3131	67.6048	67.2820	0.5593	220.1195	51.4409	123.8838	106.5589	0.2254	16.1430	3.0352	15.7811	7.9763	5.0947	0.0091	16.4367	137.5878	14.2238
TARGET-50-PAKJGM	0.2741	3.6783	7.1843	13.2692	22.5959	0.4967	190.2367	86.2068	77.6294	28.8371	0.1606	4.1719	1.6747	10.3096	5.9059	20.7757	0.0027	8.3465	89.7175	10.7331
TARGET-50-PAJNGH	0.7303	1.8637	11.8095	28.5487	23.8515	1.1323	98.0968	59.6119	69.4855	68.1112	0.1717	12.9438	2.6162	18.5830	8.6741	11.0919	0.0081	12.3039	123.7470	11.0370
TARGET-50-CAAAAC	0.6137	1.5918	9.9314	20.7864	22.4576	0.6649	91.7232	38.6943	42.4412	39.7875	0.1912	11.6292	3.4899	21.3197	12.0371	5.0283	0.0109	16.2320	124.8276	16.2731
TARGET-50-PAJNBN	0.3813	1.0665	9.3527	22.0794	15.2209	0.7927	94.9170	39.3785	74.5688	44.6245	0.1471	6.8085	2.2631	12.8558	4.3410	2.9635	0.0046	14.5850	81.3661	10.6267
TARGET-50-PAKYLT	0.4028	4.0219	12.7449	31.7489	30.3722	0.8592	163.8883	59.7849	99.8494	60.6200	0.2599	20.4576	4.0858	40.9579	11.4294	3.7807	0.0195	23.0830	107.2793	12.5949
TARGET-50-PAJMKN	1.0038	2.6427	21.2361	58.7072	43.7537	1.6018	196.2360	61.9804	116.0194	109.8669	0.2824	11.3127	3.2930	18.2572	10.5305	13.6589	0.0164	12.4496	125.1381	9.6834
TARGET-50-PALERC	0.3300	2.1749	11.7664	29.7710	27.3781	0.7075	131.5933	50.4471	91.5716	63.2183	0.1474	9.1512	2.2286	10.3036	6.4413	6.9795	0.0049	10.9659	72.2405	7.5970
TARGET-50-PAJMFU	0.6060	2.0313	12.0284	25.6177	30.1846	0.8410	188.6566	53.6231	137.9486	92.3550	0.1698	8.3130	2.7978	13.4472	6.7059	10.8296	0.0076	19.5473	92.5598	16.1157
TARGET-50-PAKZHF	0.4943	2.6377	15.5846	31.2317	55.2695	0.4091	258.1680	90.0797	142.8975	140.5613	0.1835	9.1426	2.3182	15.9119	15.1576	25.4967	0.0039	22.2664	118.9390	18.2611
TARGET-50-PAJLSP	0.5782	1.6959	10.9940	33.0743	33.1430	0.7275	124.7790	43.1509	92.4081	60.7445	0.1660	14.4727	2.4961	20.7638	7.9069	8.9166	0.0063	14.5383	101.0815	11.5153
TARGET-50-PAJMKI	0.6403	2.0857	12.8133	34.8959	30.6680	1.6914	190.6810	59.9596	132.2131	88.3000	0.1453	8.7370	3.2789	19.3826	8.7092	1.7955	0.0098	12.6112	116.8670	16.9487
TARGET-50-PAKUIT	0.5676	1.4041	12.1813	13.4121	32.6201	0.5997	116.7105	39.7488	125.4879	59.1459	0.1353	9.1306	3.0383	17.2858	9.3943	4.2504	0.0139	14.4576	97.9565	20.2117
TARGET-50-PALDWP	0.2938	1.8644	10.2979	22.5573	19.8240	0.6474	141.9155	54.1717	72.7505	35.8137	0.1320	7.0994	2.0171	10.7888	7.3834	4.0460	0.0040	17.6802	78.3191	13.4478
TARGET-50-PAJMJT	0.4184	2.8780	13.6259	24.7751	26.8626	0.7424	217.4169	107.6694	93.6608	75.8988	0.1710	9.8219	2.3544	13.9603	14.8351	23.6864	0.0040	16.6929	107.7037	16.0294
TARGET-50-PAKWPM	0.6537	2.4715	12.0941	33.5439	46.6055	0.7710	244.6955	66.5193	91.9481	89.5310	0.1588	9.1845	2.3630	13.6003	7.5693	5.6046	0.0046	12.7705	93.8679	16.0128
TARGET-50-PAJLKC	0.3412	0.9542	9.9302	14.2404	17.2491	0.6103	106.3978	35.3471	62.3198	52.8819	0.1494	10.0109	2.3679	14.0054	8.8607	4.9761	0.0064	6.8932	84.6307	15.4996
TARGET-50-PALKRS	0.3830	5.0387	12.5566	30.5137	62.8362	0.1932	343.4658	83.9413	177.1059	44.9617	0.3469	7.0867	1.6524	18.8728	8.9040	26.2057	0.0039	4.8270	116.5010	11.7009
TARGET-50-PAKNRX	2.2010	1.6214	21.3961	59.4520	38.6017	3.2584	311.7095	82.5370	196.0850	264.3442	0.1955	17.3836	5.5603	20.0196	11.6943	9.3390	0.0240	39.1585	174.0160	12.0704
TARGET-50-PAJNTJ	0.6205	2.0565	12.4694	34.3072	26.6094	0.9762	122.9171	68.8968	93.4630	102.9115	0.2166	15.2065	2.3916	13.1375	13.3844	23.8167	0.0074	11.3090	98.7192	15.2533
TARGET-50-PAJNUS	0.3994	2.3092	12.9580	25.0444	29.0417	0.7389	188.2560	84.4369	89.9193	88.0201	0.1672	8.6040	2.2150	15.0594	10.0894	11.7417	0.0045	12.1320	100.4967	15.7678
TARGET-50-PAKNTW	0.1857	2.1477	8.3817	18.9722	21.6781	0.2394	192.8030	61.2202	121.6135	32.1913	0.1259	5.1940	1.4557	6.4628	5.6517	7.6357	0.0017	15.1139	65.8748	14.5354
TARGET-50-PAJPAU	0.6822	2.0602	13.0330	49.6104	25.8039	0.9992	120.2919	49.1064	59.1846	66.9143	0.1909	11.2656	2.5726	12.5406	6.2812	4.4740	0.0086	26.0908	134.1545	10.8652
TARGET-50-PAJMVU	0.6109	3.0145	16.9104	53.8057	67.8024	0.6913	275.0883	80.5732	119.1453	88.1967	0.2342	8.3412	2.7161	19.6225	12.1347	17.0189	0.0087	15.0481	133.6121	25.5129
TARGET-50-PAJNZI	0.2499	0.9316	8.2263	14.2832	11.1547	0.9927	94.0656	24.0525	62.7920	49.7285	0.1592	11.5507	2.6732	18.9274	6.4565	5.7686	0.0116	16.4638	80.3349	15.5292
TARGET-50-PAJPDN	0.5522	4.5729	10.7240	47.4382	55.0155	0.2403	264.2133	58.6494	182.7174	45.3951	0.3073	8.8381	2.1838	12.7963	6.7849	15.2274	0.0041	8.3939	105.4101	10.1528
TARGET-50-PAJNVE	0.2608	1.2479	10.7542	24.6368	14.5284	0.7408	77.7634	25.6553	64.4698	47.7847	0.2202	9.8904	2.6690	17.8972	3.7498	2.3121	0.0091	12.7251	83.2942	9.2810
TARGET-50-PAJMSE	0.3776	3.0337	15.7947	23.5352	31.8426	0.7860	125.5039	59.9831	78.1680	119.8117	0.2226	11.2754	3.0012	14.8307	9.3602	8.7751	0.0081	12.9482	85.4225	14.7936
TARGET-50-PAJNZU	0.5329	1.9003	16.3830	34.8471	28.3959	1.0397	152.0398	68.9571	101.8335	116.0347	0.1946	12.4100	2.8865	14.2802	6.6609	1.8645	0.0101	14.3290	108.7488	11.0863
TARGET-50-PALGLU	0.3503	2.2584	14.1815	35.8170	30.5507	0.3414	172.9186	47.1882	83.9927	64.5683	0.2386	7.2771	1.5075	8.8598	8.6644	5.2533	0.0033	8.0856	83.3443	9.1118
TARGET-50-PAJNZK	0.4126	1.6695	12.1034	42.1309	32.1827	0.5894	103.5241	40.1628	64.9512	50.3355	0.1701	9.8406	2.2495	10.5137	6.7542	4.6101	0.0114	9.8209	105.0720	15.9211
TARGET-50-PAJMUF	0.2088	1.2689	9.6514	27.0215	30.1872	0.5721	133.2482	56.5133	82.0383	59.6021	0.1786	8.0078	1.9602	11.9265	5.8241	8.4996	0.0047	10.3363	84.8604	18.2460
TARGET-50-PAJMRL	0.4814	1.8662	12.1692	38.0125	32.3239	1.7826	169.7433	45.0380	131.7996	100.9222	0.2298	14.1862	3.7360	17.8592	10.8808	8.3011	0.0151	24.4879	101.7142	15.8208
TARGET-50-PAJMXF	0.3531	1.4228	10.4261	24.0957	18.3187	0.9102	132.3276	48.4702	65.3515	60.2603	0.1865	9.2210	2.2888	18.4467	5.7137	13.7363	0.0073	9.5694	100.4476	10.7652
TARGET-50-PAKPDF	0.4162	1.5375	11.3220	25.7611	24.3758	1.0160	94.6147	29.0325	65.7232	83.6548	0.2026	10.4391	3.1253	19.3088	7.9838	4.7389	0.0131	17.7724	92.4978	12.0810
TARGET-50-PAEBXA	0.7395	3.0156	15.6209	31.9063	49.1139	0.4773	202.8900	82.3210	149.9375	129.1995	0.1851	10.5102	2.2432	16.6616	12.0450	16.6263	0.0059	11.8037	105.2116	16.8960
TARGET-50-CAAAAS	0.7111	1.9598	16.2576	40.6074	25.4294	1.2438	110.6542	54.6363	50.4174	73.5570	0.1950	21.6559	3.0339	23.1746	8.8541	6.4921	0.0198	15.1143	114.9492	9.8431
TARGET-50-PAJNLT	0.3319	1.6664	12.3973	37.4024	29.6015	0.8640	150.9846	60.1711	111.7726	60.6138	0.2068	16.1170	2.6389	17.3785	8.8452	7.9881	0.0066	18.3795	100.3423	11.8689
TARGET-50-PAJPHA	0.4784	3.1430	11.2511	42.4322	38.7815	0.5921	155.0777	93.7471	109.2461	78.0148	0.2104	15.1862	2.6969	17.6779	9.1384	14.8828	0.0088	8.4768	116.0315	11.6497
TARGET-50-PAJMMY	0.2647	2.4283	9.2256	24.2109	20.0646	0.5511	75.1281	48.3195	62.2680	52.7676	0.1931	14.2749	2.2755	19.9920	5.8162	5.9706	0.0065	5.6498	86.6826	8.6588
TARGET-50-PAJLTH	0.3819	2.0345	13.6472	25.6516	38.6312	0.5149	168.7152	51.6047	89.0282	61.0526	0.1961	11.9200	2.2636	9.4107	7.6765	6.5545	0.0048	9.4190	122.3302	16.9025
TARGET-50-PAJNCC	0.2886	2.2295	12.3162	16.0845	29.8066	0.2795	195.9154	63.4793	115.9090	46.4928	0.1395	9.8187	2.2962	13.8638	9.3613	8.9487	0.0077	9.2663	76.3159	14.3716
TARGET-50-PAJLUJ	0.3143	4.5861	10.9178	16.9149	24.6362	0.3650	187.7481	104.7011	95.0397	72.7136	0.1693	4.7946	1.4978	9.7334	5.2772	13.3674	0.0031	5.5241	69.9964	11.7183
TARGET-50-PAJNCJ	0.5355	1.5920	14.6228	33.5334	37.0348	1.0371	140.8142	43.4723	108.2294	71.4452	0.1992	11.8747	2.8163	12.8109	8.5200	5.6830	0.0082	11.6543	103.2740	13.8082
TARGET-50-PAKZER	0.5119	2.9219	16.5637	22.7978	43.4590	0.2753	142.3744	66.0056	90.9648	77.3652	0.1689	11.1565	1.6874	13.5158	7.4071	3.2915	0.0038	5.6388	93.6312	18.7437
TARGET-50-PAJMEP	0.4037	1.6385	11.3034	30.6052	23.7496	0.7087	89.4353	39.8668	67.0767	51.7296	0.1717	12.9431	2.2018	12.5842	4.7336	3.5682	0.0053	9.2834	79.0064	9.3364
TARGET-50-PAJMEL	0.3716	3.0268	14.8552	36.2691	33.6048	0.7172	176.8441	79.4214	121.2893	137.3056	0.2165	13.0753	2.2274	14.8904	8.4065	6.0185	0.0061	11.2813	98.7025	13.2228
TARGET-50-PAJNVX	2.6949	6.6889	24.8022	118.9033	63.9082	1.5820	258.4885	125.6903	154.2026	435.7746	0.3370	22.7650	4.6063	54.6808	16.1208	12.8511	0.1250	13.7286	142.3534	38.5823
TARGET-50-PAKXWB	0.8279	5.6362	13.2897	49.0893	29.9003	1.2290	190.1821	99.6182	97.1335	156.3404	0.1806	7.6415	2.2896	19.9298	4.0259	6.1658	0.0116	6.0268	90.7233	13.6603
TARGET-50-CAAAAB	0.5091	1.2773	9.5063	20.7248	21.6193	0.9295	97.1296	43.5312	92.2674	81.8448	0.1764	11.7016	3.0406	17.7625	4.7742	2.5038	0.0085	5.7650	92.4464	13.9759
TARGET-50-PALJIP	0.4200	1.8333	14.6409	19.9557	29.0457	0.7932	197.2631	49.1937	83.2574	76.2089	0.1207	9.6845	2.3632	9.4338	9.9622	5.0310	0.0067	5.5968	105.4714	12.1449
TARGET-50-PAKMUB	0.9844	6.9001	25.8003	55.7797	59.4123	1.0194	223.5591	132.7395	168.6053	300.3403	0.2692	13.6589	2.7365	24.9206	13.7889	18.2072	0.0205	8.5869	122.6163	19.8049
TARGET-50-PALKCW	0.5542	2.1698	14.4601	23.3768	33.3551	0.6607	167.9535	58.5243	122.8677	102.5117	0.1571	7.9248	2.2434	9.5070	7.2944	12.5050	0.0043	13.1774	85.3085	14.4106
TARGET-50-CAAAAQ	0.2230	1.4642	7.5981	16.9955	16.2260	0.3395	107.0291	42.5579	59.6038	26.4928	0.1682	7.3373	1.6820	12.3014	5.7580	7.6262	0.0026	11.2977	80.4315	8.3328
TARGET-50-PALFME	0.4130	1.4598	11.7585	20.3709	24.6221	0.5990	132.7558	50.7028	97.1357	65.8276	0.1831	10.1661	2.4128	14.4211	9.5997	14.8411	0.0058	19.2514	87.6419	18.6072
TARGET-50-PAKNXS	0.8341	2.4448	14.5008	63.7503	46.6608	1.7402	136.8489	50.0935	157.4499	92.3880	0.1984	10.5095	4.4800	27.2937	9.0673	5.5625	0.0203	23.5851	119.8450	10.4951
TARGET-50-PAJNAA	7.3199	6.0822	42.7884	192.4690	124.3400	3.8287	431.7003	116.7657	326.7727	432.9368	0.3401	21.5086	6.5009	47.1236	19.8324	31.9406	0.1704	39.0352	241.3046	36.9360
TARGET-50-PAJPGY	0.3874	2.8029	13.4438	18.1899	27.5146	0.3783	129.8713	71.4743	76.6806	61.8743	0.2566	14.0431	2.1039	17.7918	8.2283	5.3888	0.0060	6.8694	101.8095	17.1133
TARGET-50-PAJNEC	0.3248	9.1498	10.6114	23.0740	176.6502	0.1163	924.0107	154.7094	268.9704	44.3760	0.2604	4.6525	1.5737	11.3779	14.0140	48.9572	0.0018	8.6247	172.8783	18.2946
TARGET-50-PAJNDU	0.1700	4.1458	7.7893	20.3442	56.4705	0.0653	238.2026	54.0724	136.9600	12.2313	0.2536	3.9545	1.1745	5.6978	6.2123	30.5668	0.0010	2.9281	88.2140	7.6547
TARGET-50-PAKGZX	0.5939	7.9379	18.5584	49.3549	44.0251	0.7122	166.3841	115.2109	98.4781	165.0638	0.1621	7.2025	2.0477	15.1675	8.3778	15.8845	0.0084	12.3764	103.0370	20.7173
TARGET-50-PAKFYV	0.4668	0.8647	11.8909	28.0371	24.3806	0.9517	86.3367	28.8421	85.4918	48.7814	0.2246	11.0234	3.8109	12.8043	7.5391	12.0694	0.0163	20.6634	81.5608	9.5993
TARGET-50-PAKGED	0.5456	8.9384	9.4943	21.4250	159.5803	0.0560	592.1510	103.0299	687.7091	38.4680	0.4087	6.2705	1.2711	16.6073	22.7342	52.3980	0.0019	3.9057	180.1268	12.0617
TARGET-50-PAJLTI	0.5411	2.7002	17.4235	47.3852	46.0248	0.7231	152.3508	71.3486	111.8536	90.6602	0.2246	14.1743	2.3150	20.2540	8.7693	7.9573	0.0064	10.5839	105.7572	13.4395
TARGET-50-CAAAAO	0.4358	2.2398	12.0850	23.7186	34.0588	0.3344	189.9625	55.7101	131.4454	70.7092	0.1708	6.6774	2.1031	8.4875	6.7514	12.2362	0.0033	15.6959	79.3121	12.4195
TARGET-50-PAJNCZ	0.4488	1.6075	12.3283	26.8710	25.9909	0.7068	142.8153	52.9880	102.4575	71.6843	0.1840	9.8116	2.6980	13.6004	7.3399	7.6874	0.0056	13.8195	96.6637	13.1784
TARGET-50-PAJMIZ	0.7302	5.7291	14.4785	33.6952	49.0908	0.5292	355.5634	101.6197	113.4203	94.0176	0.2198	5.7390	2.5955	13.9718	10.8474	21.0555	0.0055	17.1346	119.2288	13.7337
TARGET-50-PAJMFY	0.5958	1.8499	13.2418	24.7316	30.9663	0.6078	154.6099	46.0947	87.1308	80.6382	0.1794	8.7444	2.9325	14.6079	6.6536	3.9835	0.0068	16.1236	95.4538	12.6603
TARGET-50-PAKULH	0.2856	2.5336	11.0731	13.3245	21.1535	0.2867	111.5002	50.3486	58.5213	50.9579	0.1720	9.3315	2.1909	20.1606	9.4961	11.5727	0.0052	7.4116	79.1926	17.1519
TARGET-50-PAKVET	3.3845	3.0614	31.8047	120.0676	50.5421	4.6608	157.0237	67.9770	110.8661	152.3030	0.2443	17.6332	5.5463	36.5776	14.0652	5.4256	0.0758	27.1758	191.7180	9.4999
TARGET-50-PAJMEN	0.4225	1.9537	14.4996	22.2346	27.9566	0.7012	171.0183	48.0619	116.1815	59.4186	0.2509	6.7712	2.0134	10.4983	7.5108	13.7932	0.0052	6.7867	77.0980	13.4074
TARGET-50-PALDTE	0.4565	2.1438	12.9894	25.6811	35.0276	0.6628	161.2071	54.4665	103.5245	84.3459	0.1801	9.8520	3.0536	15.6731	6.1342	4.6674	0.0073	21.2796	104.6161	13.4558
TARGET-50-CAAAAH	0.2094	3.6010	9.7700	27.3342	102.8101	0.0845	545.5957	54.4765	267.5084	28.4080	0.2922	5.0491	1.8109	10.3343	11.5391	27.6752	0.0027	10.0169	159.6994	21.7693
TARGET-50-PAJNRH	1.2731	1.9615	11.0743	29.5893	26.8420	0.6944	145.3510	52.4153	103.5333	59.9117	0.1613	7.6587	3.0436	13.5800	5.4598	5.7081	0.0096	19.6690	113.0956	7.8356
TARGET-50-PAJNRL	0.3955	0.9686	9.6804	19.3764	19.5563	0.4695	108.7619	36.9719	74.0920	57.6162	0.1963	9.6801	2.3750	13.8509	5.9829	8.3789	0.0064	13.8406	102.9079	16.9328
TARGET-50-PAJNUP	1.2901	8.5735	30.4477	134.0376	157.4285	1.1637	562.1175	146.2444	424.8568	339.9992	0.4346	27.7739	4.6628	52.7593	24.6794	23.4832	0.0620	20.2520	220.6152	40.0876
TARGET-50-PAJPDC	0.6725	3.1139	14.4449	38.3264	24.6933	0.6540	124.2634	60.3933	50.7121	103.1668	0.1679	8.4868	1.7942	12.5180	6.3970	3.4571	0.0092	13.8850	88.7838	15.5492
TARGET-50-PAJMLI	0.3761	2.3110	12.3231	29.2081	34.8873	0.5155	144.9756	68.0921	103.3393	96.6498	0.2184	12.3423	2.6567	21.5570	6.5415	4.0357	0.0068	9.9350	98.0214	14.8577
TARGET-50-PAJLWT	0.4357	1.7211	11.6307	25.4910	30.2548	0.5398	184.6063	56.6713	105.2934	69.2214	0.1640	7.7583	2.5216	11.4086	7.1642	10.4472	0.0041	12.0392	85.6526	8.3697
TARGET-50-PALEZT	0.7097	1.7123	11.6146	35.2725	27.1075	1.2849	135.1226	41.2284	84.0964	90.2581	0.1763	7.5342	3.3150	20.0087	5.0417	7.9657	0.0090	12.6916	80.8489	11.7050
TARGET-50-PAJLLF	0.8962	2.2458	17.1834	45.2075	65.9348	1.1070	335.3867	89.4791	291.1770	160.8732	0.1390	13.5613	3.5469	16.8347	8.9803	2.4020	0.0156	24.4049	169.3163	27.7495
TARGET-50-PAJLNJ	0.3143	2.3583	10.3244	24.4628	31.5553	0.4243	180.9349	74.1763	124.0766	58.9833	0.1643	8.0581	1.9519	8.5404	5.5756	8.5584	0.0032	11.4328	78.6507	13.1115
TARGET-50-PALFRD	0.4087	2.1432	11.3437	20.6948	26.7277	0.6026	170.9590	46.3091	117.1771	68.4076	0.1689	6.9963	2.0081	11.7489	5.7180	14.7695	0.0053	4.4337	82.6164	13.4046
TARGET-50-PAKKSE	0.3635	3.0467	13.5478	33.1984	33.8972	0.6353	188.0285	84.0585	147.9526	83.7199	0.2254	13.5702	2.3475	16.2552	9.5284	9.3798	0.0067	12.0586	103.2962	17.1720
TARGET-50-PAJLPX	0.4951	2.5920	16.5931	82.7827	58.9470	0.5729	154.2127	48.1102	81.3263	85.0187	0.2418	14.9489	2.5297	13.5307	10.3383	4.7488	0.0142	19.8421	111.3833	21.8927
TARGET-50-PAJMKJ	0.4716	2.6843	10.7538	20.0357	33.8492	0.5146	146.9076	60.5408	99.2708	98.0476	0.1662	10.0029	2.1257	20.0465	14.8619	17.3793	0.0088	4.8441	104.1820	18.0695
TARGET-50-PAKSDG	0.3591	1.9523	11.7487	25.3978	22.2907	1.0196	164.3313	62.8947	78.8337	73.7524	0.1761	8.9728	2.4158	14.5763	7.4194	4.7405	0.0061	8.9919	91.8941	10.3143
TARGET-50-PAKZFK	0.6830	3.6680	16.7145	32.6541	60.4666	0.4603	212.5277	85.1074	163.0663	110.0651	0.1747	9.5188	3.0764	18.1853	15.1484	19.8909	0.0061	17.8143	111.8610	14.6523
TARGET-50-PAKYFC	0.6489	2.1919	16.3558	48.7263	35.5951	0.9673	139.3774	63.4479	115.7310	96.7122	0.2459	15.1349	2.4638	13.2026	7.3714	5.8137	0.0084	15.1146	91.3059	17.0126
TARGET-50-PAJNNR	0.6472	2.7913	15.9292	45.7103	59.0467	1.0804	189.2857	56.0766	155.9824	113.9014	0.1461	8.9940	3.4401	17.2309	9.2698	7.5593	0.0070	16.7624	123.3199	18.3332
TARGET-50-PAJLIP	0.9785	3.3146	24.4175	113.8068	75.8065	2.9271	261.5629	77.3287	207.3720	285.8938	0.2834	34.7856	6.2987	47.9191	20.6348	12.6170	0.0378	26.9790	144.3204	19.0311
TARGET-50-PAJNYT	1.0365	4.5552	15.6881	40.8319	41.1269	0.8372	157.2786	83.5358	175.2183	158.3654	0.3298	21.5071	3.3353	19.2028	8.4691	3.9011	0.0149	9.9888	93.5682	15.0670
TARGET-50-PALGVY	0.4674	2.7821	13.6261	27.3302	51.9644	0.3523	246.5692	68.8509	160.0035	78.3840	0.1730	6.8692	1.8990	8.7877	13.2706	22.1565	0.0028	13.8269	99.9888	19.3696
TARGET-50-PAECJB	0.2618	1.6282	10.2082	25.7109	22.2149	0.6246	98.2464	52.4917	64.1161	57.0361	0.1844	8.8048	1.8813	11.7058	4.8337	3.9734	0.0042	7.3683	72.2608	11.3185
TARGET-50-CAAAAM	0.5094	7.5070	23.3837	32.4802	58.3809	0.6566	352.4945	96.8375	286.1296	102.8986	2.0158	48.2439	5.1791	102.0246	31.3945	8.9363	0.0538	9.9714	219.8478	22.6493
TARGET-50-PAKSCC	0.2498	2.2308	9.6838	16.6427	26.4362	0.2673	128.6948	48.0408	69.6410	39.9502	0.1700	5.7673	1.9688	11.1821	3.7949	3.4504	0.0035	7.6495	72.0841	9.1002
TARGET-50-PAKRZW	0.4898	3.3870	16.6133	20.7512	30.0785	0.5120	207.2140	74.1422	157.1001	62.3784	0.2962	13.4479	2.7569	22.7302	10.8169	10.6963	0.0121	6.8483	101.1732	10.8700
TARGET-50-PAJNSL	0.3199	1.8256	12.4797	31.1368	26.5070	0.7496	111.1438	46.4733	74.7551	70.8910	0.1709	11.8555	2.4870	12.3626	7.3658	4.9317	0.0057	10.2614	84.5845	6.4663
TARGET-50-PAKKNS	1.1812	2.8136	20.0660	53.5363	47.5628	0.8782	255.9722	89.2940	149.9200	168.6272	0.2418	11.4742	3.4712	15.0215	8.1728	5.2436	0.0135	21.0118	151.2036	23.3166
TARGET-50-CAAAAL	0.6891	2.0081	14.6691	34.7030	30.9172	1.0834	197.3971	58.4783	130.0174	117.6435	0.1939	11.2124	2.9423	16.3227	9.8349	10.4283	0.0088	18.7395	108.7757	17.1035
TARGET-50-CAAAAJ	0.3074	2.3012	10.9860	26.4952	35.2248	0.4046	188.6921	74.1446	128.8757	62.5897	0.1717	8.9518	2.3083	9.8608	7.3381	8.2701	0.0033	14.7827	79.2377	13.6599
TARGET-50-PAKNAL	0.6115	3.1588	11.5857	36.4150	37.3766	0.6265	247.7228	102.2392	127.4536	144.1251	0.2068	16.5325	2.3606	22.5962	6.9546	8.6584	0.0102	8.9232	92.0005	14.4407
TARGET-50-PAKXXF	0.2417	2.4210	11.9788	36.0948	39.2842	0.7542	211.6273	36.7888	143.5203	42.1531	0.2176	7.7238	2.1555	13.3426	7.5995	10.2094	0.0071	21.1983	86.1216	13.2188
TARGET-50-PAKECR	0.5934	3.4073	21.7449	25.4981	52.8079	0.2921	202.2786	69.1096	152.7119	101.7868	0.3257	13.8302	2.3865	17.8940	13.8763	5.5259	0.0097	10.4804	128.2199	27.9645
TARGET-50-CAAAAP	0.5412	2.4228	14.4044	40.2109	38.8336	0.6187	128.6508	53.2589	77.1238	67.2811	0.1426	11.5431	2.7812	18.8949	10.8346	19.8344	0.0191	21.3337	95.2900	30.0270
TARGET-50-PAEAFB	0.4960	2.0412	13.6185	37.1074	47.4729	0.3686	152.2967	63.3251	137.3010	82.9767	0.1831	11.4017	1.9137	12.0155	6.9983	5.5795	0.0026	13.2630	91.9212	15.9215
TARGET-50-PAJLKR	0.5743	2.4285	15.7995	28.8263	49.5491	0.6466	302.1783	77.2915	142.1997	83.5433	0.1403	11.6119	2.7021	11.8794	11.8629	15.7038	0.0087	21.8351	124.1960	22.8272
TARGET-50-PAKMSV	0.2397	2.9590	7.6840	18.8273	24.5248	0.2586	203.8538	65.5407	80.1472	23.3298	0.1343	4.9565	1.6383	11.1672	4.1207	12.8567	0.0027	9.8536	80.9166	12.3476
TARGET-50-PALLFB	0.6531	1.1454	12.9409	51.9359	31.3634	1.2503	116.1785	31.6382	105.6348	121.2477	0.1606	9.8723	3.2592	16.0479	9.3620	6.0591	0.0117	20.9888	86.7301	17.9269

Supplementary table 1 continue8

	AZD5363_1916	AZD6738_1917	AZD8186_1918	Osimertinib_1919	Cediranib_1922	Ipatasertib_1924	GDC0810_1925	GNE-317_1926	GSK2578215A_1927	I-BRD9_1928	Telomerase Inhibitor IX_1930	MIRA-1_1931	NVP-ADW742_1932	P22077_1933	Savolitinib_1936	UMI-77_1939	WIKI4_1940	Sepantronium bromide_1941	MIM1_1996	WEHI-539_1997
TARGET-50-PAJNAV	43.9140	15.3819	12.9536	10.6777	18.0736	71.1251	212.7417	2.8745	255.3520	100.1615	9.1070	564.9221	40.4929	146.9061	11.3674	113.1616	29.8647	0.0310	70.6835	54.0167
TARGET-50-PAJPEW	29.8860	11.1749	21.0442	7.6433	11.3077	48.3662	163.4909	1.7646	174.1021	85.1205	1.3394	246.8653	15.1817	118.4114	17.4041	20.9037	60.7955	0.0226	109.0224	71.0642
TARGET-50-PAJMLZ	38.0344	20.5170	39.2181	5.4945	10.8126	44.4996	194.2788	3.7785	152.1418	88.2721	3.0620	288.1632	38.3762	112.1908	27.7497	13.8284	41.7037	0.0056	63.7930	36.6282
TARGET-50-PAKRVH	32.4021	16.6111	33.5517	4.7299	10.1349	53.5367	202.4458	2.9880	159.6707	67.5470	2.0105	247.4738	18.1193	150.4971	22.7784	16.3637	48.4473	0.0198	61.6604	70.3660
TARGET-50-PADZUB	24.4857	5.8571	34.5383	6.9514	11.1503	37.4104	167.1946	2.2704	160.4437	79.7716	1.3626	258.7960	10.5053	110.0514	19.7024	15.0155	46.7500	0.0140	69.5491	36.7802
TARGET-50-PADXAY	23.0279	8.4711	38.7226	16.3391	10.8940	35.1601	80.5827	2.0983	197.5044	110.0983	2.1255	335.4153	70.5807	120.2166	6.3919	11.8125	39.4305	0.0036	21.7622	31.2513
TARGET-50-PAJPCM	50.6334	5.9757	52.0962	4.5945	8.2304	44.7491	125.2295	1.5858	111.0691	48.9040	0.9533	142.3200	7.1143	49.6740	12.4369	13.2382	34.0777	0.0110	57.1704	14.3598
TARGET-50-PAJPAR	17.4754	11.1166	32.7731	4.0200	5.4993	42.3270	125.4827	2.9885	115.1708	68.0436	2.3692	255.3855	22.9385	59.9100	18.1967	13.6001	34.0661	0.0084	64.3725	17.0608
TARGET-50-PAJNZS	33.4714	17.3391	42.8916	5.3295	9.5172	55.7817	166.8946	2.7579	153.8043	104.4757	3.1962	275.2890	17.0838	87.6186	15.4925	15.1694	40.5598	0.0105	54.1483	40.9219
TARGET-50-PAJMVC	4.7625	6.5627	6.0757	3.5351	6.6500	11.4195	78.1904	1.1939	103.5254	127.0943	1.8255	259.1957	20.9644	93.8332	14.1281	15.3615	67.4386	0.0244	30.1051	37.6712
TARGET-50-PALGAZ	21.6659	7.2774	26.1402	6.4514	7.9337	47.5840	185.9825	1.8701	169.3205	104.3194	1.7420	245.2060	13.6014	92.0695	14.4438	16.0423	39.8622	0.0171	60.7970	43.1391
TARGET-50-CAAAAR	10.1435	9.0744	12.2788	3.1700	5.2551	18.4949	137.1216	1.4624	138.7164	81.0402	1.7276	180.1802	9.3848	72.3383	11.0148	12.4988	41.7865	0.0175	61.2315	31.9669
TARGET-50-PAKGMU	32.4851	10.9780	53.6083	11.8700	8.9486	63.4080	196.8849	2.0679	173.8202	98.7898	2.0245	397.0249	24.7750	182.9941	15.8618	22.1228	44.8305	0.0112	46.1970	22.8146
TARGET-50-PAKRCC	15.1352	4.0318	33.5059	4.0473	6.7597	22.8911	100.3268	2.9295	108.1970	37.7901	1.3167	127.7182	10.3767	56.1736	8.5984	8.9746	37.8891	0.0163	43.7728	11.2942
TARGET-50-PAKFME	21.5133	6.2664	26.5836	5.6037	9.6353	31.3332	183.5583	1.9476	141.3078	87.7695	1.7947	204.3040	14.2074	85.7973	15.2651	11.2747	40.3846	0.0184	48.7414	39.5853
TARGET-50-PAJNNC	22.1625	6.1882	23.2042	4.1924	8.1017	30.7871	179.5079	1.2277	139.9871	92.2120	1.3685	213.1738	14.9620	110.4102	19.5196	14.3207	35.0110	0.0126	56.6735	28.7289
TARGET-50-PAJNJJ	23.6863	13.0138	29.4558	6.7515	9.8532	46.7022	184.2808	1.5357	282.3906	144.5314	2.6854	430.7749	28.9910	91.9641	14.2831	12.0283	39.3326	0.0074	68.2614	51.7496
TARGET-50-PAKJGM	13.0611	3.0495	24.8357	5.5252	5.1572	26.2929	62.7897	1.1190	68.9880	38.6792	0.6718	165.7419	20.6020	67.4692	11.0527	13.6365	32.3998	0.0065	16.9944	20.8902
TARGET-50-PAJNGH	16.2719	11.3424	21.9187	2.9784	6.4377	28.5390	116.1202	2.4354	120.7040	77.6729	1.7220	176.5504	15.5627	68.9349	15.4607	7.7137	33.6961	0.0092	45.5653	21.6353
TARGET-50-CAAAAC	13.7245	4.7999	54.5262	4.9895	13.9805	27.1858	162.9526	2.4531	112.5296	71.3468	1.3924	217.9677	34.0119	81.6356	15.5814	8.6459	31.7908	0.0059	54.7871	22.0332
TARGET-50-PAJNBN	18.4094	3.4353	34.2248	2.7324	4.7861	29.7311	94.3012	1.9199	84.6071	37.2987	1.0916	85.8561	7.6488	47.3911	18.5037	7.4705	32.0887	0.0090	41.6816	11.5448
TARGET-50-PAKYLT	35.7180	15.4781	34.3752	7.4704	16.6776	57.9030	208.2932	2.5759	161.6030	78.1987	2.8704	242.9550	8.9524	83.2519	11.1153	21.2659	44.0874	0.0160	68.5749	23.2536
TARGET-50-PAJMKN	14.3290	16.8533	14.0519	4.1684	10.4093	30.8544	196.9840	1.7846	221.4771	80.7938	4.2942	418.7134	56.2739	116.3413	17.5016	38.2233	41.7647	0.0185	67.2103	58.0181
TARGET-50-PALERC	13.2730	5.5813	22.0669	2.4832	4.7576	25.4727	100.0795	1.5291	94.4616	45.8196	1.1153	145.8915	10.7360	47.8585	11.6595	9.2968	36.4469	0.0113	35.5244	29.6543
TARGET-50-PAJMFU	27.4538	7.2500	46.1866	6.7394	6.0975	47.3262	129.5654	1.8762	115.4150	66.1925	1.6419	189.2029	17.3094	103.6668	17.7079	18.3521	46.3804	0.0198	51.3311	35.8703
TARGET-50-PAKZHF	19.3921	8.2160	21.4067	7.2910	8.2256	43.7526	171.3541	1.5815	140.1714	90.5055	1.1888	173.3748	8.1635	94.0487	19.4360	31.8401	38.6635	0.0171	76.8338	88.1364
TARGET-50-PAJLSP	19.4405	7.2863	28.0573	2.7522	9.4940	26.6712	125.7616	2.0164	167.9782	51.4370	1.6624	161.0843	15.3783	62.7100	11.1176	8.5864	33.4480	0.0065	48.8411	21.8549
TARGET-50-PAJMKI	12.9388	5.5589	32.3078	5.5581	6.4974	23.9817	114.8377	1.5405	120.3737	78.8289	1.2223	186.1022	10.8849	81.3398	20.6048	10.1002	53.7772	0.0123	37.0887	6.2085
TARGET-50-PAKUIT	30.5240	2.8267	40.5910	7.9449	12.5657	32.4192	127.3600	1.7969	106.9649	66.7004	1.3725	164.6198	12.0882	51.7326	30.3993	5.9031	45.6722	0.0068	50.0520	20.3276
TARGET-50-PALDWP	20.1896	3.7960	41.0258	3.6778	6.1862	38.2962	128.1718	1.9603	90.8298	44.4519	0.8356	126.8140	8.4463	46.0679	10.8604	9.9787	33.1577	0.0087	34.6783	18.3748
TARGET-50-PAJMJT	14.3584	5.9144	26.8550	4.2854	9.4695	28.2342	169.5603	1.4608	121.7721	74.5425	0.9338	195.5324	12.6788	110.5126	20.3474	16.9312	43.0736	0.0084	76.6281	53.4247
TARGET-50-PAKWPM	15.8018	6.1299	30.9837	4.2248	8.1323	27.5522	140.1470	1.4796	128.9877	73.3910	1.3536	220.3249	15.1961	102.8631	16.8409	11.7557	34.2414	0.0103	49.5308	27.5658
TARGET-50-PAJLKC	10.6407	6.0198	42.0786	3.6324	8.8966	18.6768	109.6641	2.3725	94.6522	37.7011	1.1304	96.2774	12.2897	54.2312	8.4023	8.2697	28.5024	0.0062	27.2810	10.6844
TARGET-50-PALKRS	14.2116	5.7357	11.4968	9.7789	10.1976	25.5629	113.0795	1.0077	127.8185	97.7671	1.4135	429.7990	45.8381	207.7262	11.0826	30.1216	48.2155	0.0151	34.5980	81.3877
TARGET-50-PAKNRX	52.2987	56.8971	42.3132	5.9482	10.2923	98.6661	167.5542	2.2040	231.1949	128.6044	2.1869	333.5370	45.5302	160.6421	27.6956	16.2256	42.4753	0.0037	57.9842	17.8384
TARGET-50-PAJNTJ	9.6656	21.5069	30.5883	3.1015	9.4616	27.1440	115.5841	1.6044	126.1654	62.4388	1.8422	222.2961	15.8478	81.8136	11.1033	15.3350	36.5727	0.0134	46.0311	58.3157
TARGET-50-PAJNUS	12.7347	5.7621	26.5631	4.2966	7.3790	23.7064	136.8647	1.3545	107.4449	76.5747	0.9397	179.9917	9.0353	71.6327	18.8848	14.1185	43.2756	0.0107	60.2303	37.8715
TARGET-50-PAKNTW	18.6482	2.2095	30.8977	5.6572	7.4059	31.0719	59.3102	1.0647	71.9624	29.8832	0.4937	79.1162	4.3225	41.6788	9.2353	10.8595	44.8878	0.0154	29.9862	20.5535
TARGET-50-PAJPAU	34.4527	6.5284	17.1793	3.5174	13.9893	53.3054	163.7200	1.8379	202.1254	85.9543	2.5137	282.9192	18.2297	92.8832	12.9308	12.0247	33.5301	0.0272	51.0999	23.0144
TARGET-50-PAJMVU	15.9807	7.7754	42.5922	9.5428	11.5689	39.0943	126.8718	1.6089	168.2880	89.8092	1.3916	358.0002	33.2345	96.8536	16.3343	13.6703	41.8148	0.0076	45.3875	57.4212
TARGET-50-PAJNZI	22.1316	4.3237	44.8642	3.7542	8.4955	39.6077	82.1988	1.7855	73.7657	41.9911	1.4153	98.7615	11.7515	46.6184	13.6433	9.2158	37.2596	0.0128	71.4294	19.6272
TARGET-50-PAJPDN	17.4855	5.4206	13.1184	9.1386	8.8788	38.2571	90.4975	1.3298	96.6423	110.2717	1.0236	330.3183	37.2149	85.9754	7.8085	15.0567	52.3794	0.0154	29.4680	56.1816
TARGET-50-PAJNVE	16.4692	2.9735	25.0034	3.4704	9.1146	33.1368	104.5066	2.2528	121.3307	49.3728	1.4699	123.4479	11.2946	33.4779	8.1995	7.7550	25.7799	0.0121	28.2186	14.8633
TARGET-50-PAJMSE	15.0048	6.3709	25.6006	5.4936	6.7566	30.6150	205.4209	2.0360	129.4287	80.2570	1.5231	182.3642	11.9520	79.7385	14.7882	16.8281	36.4103	0.0165	54.4515	34.3307
TARGET-50-PAJNZU	18.4345	8.2192	17.5649	2.8727	4.9558	26.4148	182.3065	1.6883	144.7049	108.3518	1.5963	179.1207	9.1996	59.1438	19.0903	9.7544	41.1225	0.0105	64.5201	20.4828
TARGET-50-PALGLU	14.4156	3.9192	10.4593	3.8493	7.6674	19.7938	115.3661	0.9256	154.7261	81.7473	0.9873	219.7556	9.3686	54.6908	7.5431	13.3550	32.7625	0.0136	37.8390	38.6994
TARGET-50-PAJNZK	17.2679	4.2526	29.1469	5.0916	11.0590	35.4379	124.4580	1.6972	101.1000	52.8153	1.5156	148.4572	9.5507	51.6651	10.4066	7.6168	31.6360	0.0117	38.5463	28.7626
TARGET-50-PAJMUF	12.5551	6.0691	27.2099	4.6084	4.9148	27.3688	91.4115	1.3362	122.2636	58.2051	1.0038	199.1659	11.8836	82.6192	9.7957	13.4642	50.5799	0.0215	44.6989	23.9839
TARGET-50-PAJMRL	32.3427	10.6549	111.1177	10.2079	17.1815	69.2964	175.8966	4.1466	149.9730	86.9142	2.3836	227.8508	29.9546	71.9083	10.1794	13.7317	51.1294	0.0093	39.6296	13.0624
TARGET-50-PAJMXF	9.2464	10.2074	17.7491	2.8141	7.2464	22.5342	123.0806	1.3851	150.1917	48.8602	1.3131	201.6562	21.2433	87.6788	14.0698	13.0631	29.2597	0.0070	49.7014	29.8238
TARGET-50-PAKPDF	29.7159	8.5137	36.8177	5.0109	9.4520	51.9837	157.4259	2.7796	114.1869	62.2494	1.8769	163.7097	9.6213	46.8889	13.5652	10.4881	35.4546	0.0092	45.7609	23.1895
TARGET-50-PAEBXA	15.1399	7.2466	21.7252	6.2195	7.2838	30.6280	124.6158	1.3511	165.9973	102.0209	1.3504	233.9478	12.6853	88.2798	13.8238	21.4794	44.1238	0.0193	53.5042	56.9263
TARGET-50-CAAAAS	13.1359	9.5301	24.5906	3.0475	13.8701	28.8765	193.9724	2.0891	146.5376	81.9174	2.1185	180.1579	21.5641	119.7004	15.0820	11.9066	34.9498	0.0151	86.7559	32.8178
TARGET-50-PAJNLT	17.3809	5.6750	20.7240	3.6525	9.2557	42.1210	116.6406	2.2541	155.5083	75.9471	0.9536	137.4973	20.4176	65.2942	9.7044	8.8318	40.2181	0.0093	47.7279	20.9011
TARGET-50-PAJPHA	8.5173	8.1113	22.5694	3.9113	5.3750	20.7191	106.8192	1.1839	139.7499	98.1569	1.5066	246.8724	19.7481	103.0620	12.4673	10.6779	45.9770	0.0145	46.4722	48.0836
TARGET-50-PAJMMY	7.8133	7.2936	16.3985	2.5658	4.8637	14.8982	104.2940	1.4658	93.5737	59.6445	1.3206	124.3177	8.3643	37.3776	7.4888	5.8907	29.0867	0.0054	28.5918	14.5993
TARGET-50-PAJLTH	18.3233	6.0259	36.0286	6.6315	15.9708	28.9849	176.7908	1.5306	189.7773	63.5134	1.3142	257.0700	12.9585	83.3358	14.0438	12.9068	37.4536	0.0150	44.4399	35.5206
TARGET-50-PAJNCC	14.4138	1.5221	23.6383	6.3297	5.2431	30.1785	170.4110	1.2344	121.6875	56.1085	0.8345	157.1802	11.0203	96.5784	13.8810	15.7422	33.0175	0.0121	39.1881	24.1106
TARGET-50-PAJLUJ	7.0822	3.7793	11.1112	5.0635	4.4437	15.7850	77.4569	0.7751	89.6523	59.8899	0.8922	152.0700	10.9087	85.9493	9.7297	20.1187	36.4087	0.0355	36.3624	46.3419
TARGET-50-PAJNCJ	21.2585	9.9686	22.7187	4.4832	6.3886	34.9454	166.8639	2.6970	150.9290	72.9316	1.3707	196.3709	8.5288	61.8166	16.7516	14.9974	42.5370	0.0217	58.0244	46.5221
TARGET-50-PAKZER	8.9756	3.1210	17.2072	6.1153	6.7228	17.1272	118.6514	0.9431	152.6166	111.6294	1.3235	258.0401	10.4700	49.7692	8.2963	11.8930	38.5141	0.0145	45.3256	20.3274
TARGET-50-PAJMEP	12.1120	8.1054	19.2103	2.6682	6.2962	26.7832	97.9365	2.1165	127.9827	61.3775	1.3688	123.3960	10.1333	34.1200	7.1955	6.6689	30.1693	0.0068	27.6433	18.7339
TARGET-50-PAJMEL	13.0379	13.4569	13.7727	3.6604	5.7203	24.4129	151.4086	1.1141	135.4357	94.9405	1.7091	201.1657	6.1339	82.3556	14.4823	12.9524	46.9084	0.0337	70.5475	46.6139
TARGET-50-PAJNVX	20.2383	80.1892	16.7068	15.2384	11.9317	34.8164	252.1839	1.8417	217.6182	205.3998	11.0163	617.1207	19.5444	224.7191	12.6147	39.5835	57.8060	0.2574	130.9597	129.6977
TARGET-50-PAKXWB	12.0193	8.4738	14.8828	5.8862	5.7216	23.3124	91.1259	1.3378	110.5346	79.6279	2.8168	232.2854	15.1835	121.3353	10.7257	25.3686	31.1954	0.0774	37.5212	42.7852
TARGET-50-CAAAAB	12.3710	7.3138	39.0919	2.8390	6.8736	19.0868	111.0133	2.0000	108.1176	60.7961	1.5682	146.1314	13.4741	91.4827	12.6955	8.4903	27.6251	0.0075	29.0225	5.5800
TARGET-50-PALJIP	10.8236	3.6100	18.9507	3.9998	6.8191	14.8683	192.7160	1.5521	141.1734	64.7180	1.1114	192.3546	7.5785	86.0325	11.9126	12.3623	34.6545	0.0135	42.3192	14.4259
TARGET-50-PAKMUB	11.6556	23.6104	14.1455	10.3122	7.5374	41.9470	241.2333	1.1947	249.7616	218.2159	4.3648	466.5826	20.8952	266.5285	16.0639	41.0781	45.9224	0.1545	95.5924	106.5373
TARGET-50-PALKCW	17.9018	7.4903	23.2934	5.6370	5.4467	32.6788	126.8087	1.3930	147.8534	83.2728	1.3977	255.4987	15.4526	66.3638	12.0447	21.2199	36.4348	0.0153	41.7115	51.8434
TARGET-50-CAAAAQ	12.8281	3.3569	26.9213	2.5396	5.2748	23.2348	70.5604	1.0411	88.6214	42.6046	0.6439	96.3385	8.7002	30.9321	9.4618	9.1496	30.8232	0.0068	34.2646	18.6699
TARGET-50-PALFME	24.9667	3.7948	52.6236	6.3565	9.3630	38.0664	117.0902	2.0527	108.5693	66.2724	1.0082	151.7104	12.7650	70.9715	15.4605	11.2186	41.3866	0.0093	52.4574	32.2950
TARGET-50-PAKNXS	29.3926	11.0756	42.3635	6.7487	14.0807	77.5126	144.6407	2.8682	213.8861	98.3505	3.2348	511.7594	17.6863	66.4294	14.7442	14.8924	37.9548	0.0071	49.3174	16.3704
TARGET-50-PAJNAA	52.3493	84.4937	35.9648	15.9814	21.1718	108.6794	331.5731	2.7681	310.5686	175.4159	10.0722	933.9907	50.3541	428.5539	49.8540	46.4050	75.9786	0.0751	206.1980	105.7771
TARGET-50-PAJPGY	12.5167	6.8017	15.3443	4.5254	6.2169	22.6029	139.8708	0.8799	141.8443	127.8313	1.5610	268.4658	14.6079	114.1507	12.9873	11.8947	36.9247	0.0075	44.1956	22.7736
TARGET-50-PAJNEC	26.8212	4.9721	37.2978	8.4754	16.0422	24.7404	87.8894	0.8787	117.7212	99.6216	0.6294	393.4151	70.3999	316.7789	9.5964	8.7723	59.1588	0.0041	13.0326	71.9487
TARGET-50-PAJNDU	9.7355	2.6749	6.9338	4.4289	5.6333	14.2084	40.1032	0.7986	70.7633	37.1459	0.3903	220.3934	26.5961	68.4832	8.4656	11.8143	49.6575	0.0068	13.0299	67.5756
TARGET-50-PAKGZX	16.1119	9.1920	15.6116	10.6993	5.3253	44.2538	150.1468	1.5148	117.3313	110.0958	2.1671	161.5697	10.1691	132.6771	9.8630	17.3935	36.3942	0.0866	56.3250	100.7191
TARGET-50-PAKFYV	39.5834	7.1625	53.8706	3.9577	4.9006	61.8621	113.0081	6.5993	113.9179	85.7367	2.2878	133.1872	13.2751	89.5978	16.1176	15.1225	38.9115	0.0115	38.4365	27.1969
TARGET-50-PAKGED	25.9682	2.8261	2.3267	9.1397	13.4572	21.0933	73.7690	0.5856	134.6731	50.1077	0.7196	318.3886	22.1284	149.7666	15.2927	13.4079	65.8855	0.0076	36.6184	68.3187
TARGET-50-PAJLTI	12.2966	9.9071	23.5080	4.7469	9.3822	28.3183	159.5507	1.5609	153.3571	95.9643	1.9493	223.6424	14.6224	90.9475	19.2905	11.7175	40.4653	0.0126	54.7322	41.6198
TARGET-50-CAAAAO	21.6961	4.6261	29.3730	5.9016	6.6262	38.1918	112.5548	1.5636	108.0923	64.5051	1.1027	172.5306	11.0925	54.9883	9.9774	15.5831	41.7460	0.0110	32.1588	38.2063
TARGET-50-PAJNCZ	16.2189	7.4201	28.5227	3.9591	5.6310	32.1062	144.2627	1.7017	121.6827	77.3879	1.2753	158.5991	13.1511	57.2732	15.2674	12.3715	41.6421	0.0119	44.3307	34.3147
TARGET-50-PAJMIZ	26.5613	10.6007	39.8815	6.6608	8.3915	38.4971	150.8519	1.6154	134.1935	74.6940	1.3826	291.6284	31.9539	152.6524	11.7017	22.4783	46.1081	0.0111	29.3037	47.9406
TARGET-50-PAJMFY	24.8626	8.8822	35.3509	3.8374	5.5252	40.4348	155.0857	2.2608	132.7450	55.5544	1.4279	166.3746	14.3133	78.2430	14.6446	12.4390	38.2638	0.0127	39.2545	28.0661
TARGET-50-PAKULH	16.5736	2.7532	37.4561	8.1620	7.0695	25.9377	151.9209	1.4932	112.9859	69.7162	1.1026	208.6532	20.2504	99.1347	8.7951	17.2447	29.1516	0.0089	42.1586	18.1043
TARGET-50-PAKVET	27.1319	31.2859	11.8391	4.6765	12.9417	56.8454	333.8607	3.3042	303.4522	138.2962	15.6418	675.1229	79.0398	132.3491	30.7720	23.7036	42.0200	0.0252	125.7257	36.8131
TARGET-50-PAJMEN	12.4913	5.5810	17.7246	4.4040	8.1066	16.6381	107.7560	1.3773	131.5351	51.8773	1.1592	188.8423	22.1987	57.6986	13.9156	21.1311	38.1886	0.0116	35.9240	54.6086
TARGET-50-PALDTE	26.6745	9.1054	39.9277	4.4943	6.7746	49.0814	167.2675	2.2839	138.6732	71.8904	1.4123	176.6824	12.4364	71.2936	17.2700	10.4620	41.3214	0.0097	47.5039	24.1197
TARGET-50-CAAAAH	34.1721	3.4611	39.1000	17.5601	23.7725	53.2734	87.3291	1.4970	90.1964	94.9212	0.8435	344.2979	27.0643	111.7158	12.3578	14.4475	67.2660	0.0051	17.9646	54.6057
TARGET-50-PAJNRH	32.7189	6.7438	22.8139	4.6287	8.3022	39.4374	118.4569	1.9940	130.2475	96.1343	2.2244	321.7218	26.5220	64.4489	13.7036	22.2805	35.2071	0.0256	45.6381	28.6176
TARGET-50-PAJNRL	22.9955	5.6162	41.3398	6.7080	8.3526	38.6563	106.6150	1.9028	129.4490	72.4648	1.0841	147.4017	12.6611	65.1636	10.9520	9.3387	33.4547	0.0084	35.4554	20.5661
TARGET-50-PAJNUP	33.4501	52.9636	34.6547	32.5215	37.0558	84.3838	305.8971	1.7927	263.2024	248.0602	11.5608	745.1133	28.1209	405.2527	25.3421	118.6811	74.7054	0.0838	148.8891	110.4866
TARGET-50-PAJPDC	24.5757	7.5391	18.5476	5.8604	5.9892	39.2150	125.9897	0.9508	120.2434	87.0325	1.6658	166.1223	8.3261	118.2207	9.6121	18.8224	26.3836	0.0437	71.6143	29.8768
TARGET-50-PAJMLI	12.6565	9.0921	24.9275	4.7554	7.1601	24.8670	158.0003	2.0295	117.9578	76.7199	1.5057	179.9473	11.0156	73.9432	14.7513	12.2243	43.3771	0.0100	51.1512	29.6683
TARGET-50-PAJLWT	16.3857	7.9764	31.1970	3.3561	4.7846	27.3140	85.9397	1.2641	102.6615	57.8870	1.2340	212.3669	15.6704	67.1037	12.3065	16.7196	39.9700	0.0105	40.8734	40.5293
TARGET-50-PALEZT	19.0361	10.0513	31.9758	6.8191	8.3360	34.2623	112.2882	2.1553	155.5181	81.3092	2.3113	240.3762	50.8498	87.7177	7.3570	20.1587	40.2116	0.0161	35.2639	39.7432
TARGET-50-PAJLLF	51.3406	14.4399	96.9357	15.5927	15.1540	70.1417	170.2899	2.1052	178.5726	107.3824	2.4022	359.3565	20.7465	170.0538	18.3536	15.5333	62.3101	0.0182	51.7228	18.7113
TARGET-50-PAJLNJ	15.9847	4.0187	23.7040	5.0269	5.6962	29.2468	103.5309	1.2882	98.5235	61.1241	0.9140	154.3356	8.2572	50.6270	10.0464	13.0588	42.0861	0.0158	31.0733	30.3358
TARGET-50-PALFRD	9.6199	5.0703	16.8778	5.4974	6.7629	20.7961	98.2736	1.3025	116.2679	57.0346	1.0946	198.2992	16.9319	60.7198	7.6099	17.7790	35.5092	0.0119	28.2377	30.8459
TARGET-50-PAKKSE	16.0915	13.2668	16.8745	5.8923	6.4337	32.2308	143.1318	1.2918	130.3070	82.3028	1.8406	232.8320	10.1260	105.1762	14.6296	19.3716	49.3795	0.0220	65.9579	42.9990
TARGET-50-PAJLPX	28.1347	5.0353	38.8291	9.8259	18.0478	54.7800	186.8168	1.5952	162.4083	186.0966	1.7630	305.2049	21.3929	86.7631	10.9250	10.8826	40.2021	0.0139	57.9225	42.6940
TARGET-50-PAJMKJ	9.2145	3.3366	16.5950	5.6434	5.8136	19.4696	131.4203	0.9941	129.9021	95.7687	1.4710	260.3659	15.5287	125.9510	13.0583	18.2961	36.3811	0.0127	85.6821	39.4108
TARGET-50-PAKSDG	12.4193	5.4756	27.3450	3.0641	5.5609	23.5907	112.2651	1.9065	110.1192	54.6263	1.3672	172.3781	11.0272	60.2914	15.0466	10.3056	31.4395	0.0100	43.4680	17.7065
TARGET-50-PAKZFK	24.3249	9.0526	38.5448	5.2607	5.0012	36.2859	200.2315	2.1758	156.7557	82.1601	1.4511	215.7379	11.2746	71.4708	14.1513	21.9641	39.4735	0.0113	54.9911	49.2745
TARGET-50-PAKYFC	19.0413	9.7615	24.1667	5.7109	9.0326	36.3412	153.8644	1.7028	161.6916	127.3961	2.0874	187.4702	13.1412	78.5372	14.9109	13.3408	45.7060	0.0276	62.5648	47.4041
TARGET-50-PAJNNR	29.8183	7.5594	52.3582	7.7399	10.0300	51.1595	192.3589	1.8055	185.5158	75.7379	1.4879	323.8134	13.6308	83.0081	20.5531	10.0496	54.2182	0.0076	33.4441	23.7832
TARGET-50-PAJLIP	37.4082	14.8203	59.8355	9.0543	15.6420	73.2374	245.1163	4.0031	196.7219	110.6340	4.1200	347.2937	45.0480	241.4323	15.7195	30.9890	45.9844	0.0175	82.0925	24.9268
TARGET-50-PAJNYT	14.5470	20.5351	11.2739	7.1365	6.1605	35.2793	214.5624	1.9236	134.5983	131.2863	2.7172	278.6088	9.2564	99.6157	11.4431	22.8672	47.3819	0.0550	81.6890	37.4080
TARGET-50-PALGVY	17.9357	4.0689	18.1813	6.9105	7.2967	33.3199	127.8386	1.2798	134.1287	66.8926	0.9403	228.2509	8.1381	61.2234	11.1940	22.2694	45.0775	0.0212	48.9233	69.2916
TARGET-50-PAECJB	9.0106	6.2015	16.5173	2.9137	4.2606	20.8486	94.4944	1.3525	93.0278	52.8027	0.9836	150.7308	8.6847	55.9615	10.3577	10.4877	37.9669	0.0173	42.3133	22.3572
TARGET-50-CAAAAM	31.8364	207.6223	18.7275	19.6638	146.3554	27.6801	404.6123	1.6759	285.6940	193.8032	33.9163	984.2397	8.0691	196.9905	19.6252	17.172	73.5132	0.0455	269.9504	95.5987
TARGET-50-PAKSCC	13.5948	3.9095	34.4299	4.4894	5.2855	20.7596	81.2533	1.0239	84.3365	35.0662	0.9620	196.5584	14.6859	69.1729	10.6072	13.9604	34.1146	0.0123	24.5865	23.5378
TARGET-50-PAKRZW	14.5813	21.5068	13.5890	6.5901	13.6413	16.5844	137.5802	1.1327	157.5139	89.8089	2.7731	344.6694	16.5802	118.8351	16.1937	52.3018	50.0817	0.0290	75.1794	62.5029
TARGET-50-PAJNSL	11.5347	7.7692	18.7454	1.9103	5.1751	21.5132	110.8125	1.8341	106.1682	47.9860	1.2055	141.6745	8.9751	51.9286	13.9626	8.8796	37.0940	0.0110	46.9770	29.1300
TARGET-50-PAKKNS	28.8501	10.5515	31.5577	8.5355	12.4749	48.3467	175.6175	2.1045	231.2350	143.4085	2.5785	396.2651	29.8651	104.0987	14.5103	17.1138	46.1055	0.0118	57.3158	37.6338
TARGET-50-CAAAAL	23.2497	8.8724	32.7716	5.2997	7.0276	40.5905	167.1437	1.9870	131.7806	80.8154	1.6940	201.5506	14.1375	102.2889	23.5929	14.5894	49.1002	0.0172	71.8907	36.0959
TARGET-50-CAAAAJ	18.5537	4.1836	31.5673	4.0273	5.1699	30.8536	109.8874	1.4242	94.4598	55.9925	0.8857	122.4335	7.9524	53.8501	13.1171	10.7918	46.5587	0.0129	35.2446	31.2226
TARGET-50-PAKNAL	13.4512	9.4791	20.6978	3.8811	6.6348	20.4814	136.4156	1.1060	140.8726	87.8138	1.6540	284.4575	15.1880	105.1275	11.7508	17.1901	39.7750	0.0152	78.6484	43.3431
TARGET-50-PAKXXF	34.9525	4.3292	55.9128	9.0680	14.6489	71.4922	84.2044	1.8186	79.7456	51.9127	1.3299	203.4841	18.1326	94.8749	16.7749	12.8550	53.4455	0.0104	39.6157	23.0791
TARGET-50-PAKECR	25.1633	8.5480	26.9697	13.5097	19.6365	38.7138	239.9175	1.0340	221.5024	130.3889	3.7805	441.4734	9.0524	126.4854	12.9394	51.0044	43.0417	0.0295	105.2388	50.1209
TARGET-50-CAAAAP	29.3101	9.4465	54.4206	9.4702	12.0269	65.0792	167.8724	2.3673	110.1921	71.2268	2.6179	239.6095	21.0402	140.2995	9.2242	19.2089	34.2403	0.0206	68.5798	69.1177
TARGET-50-PAEAFB	20.4959	5.2331	39.2325	5.5884	8.0702	35.5312	123.3497	1.2089	115.2211	81.2683	1.2931	225.7323	10.5601	58.9884	11.5377	13.6899	40.4639	0.0146	44.8626	26.4224
TARGET-50-PAJLKR	32.5084	6.4261	42.3859	9.5940	14.7652	51.2157	162.9795	2.1579	144.5440	87.5177	1.2773	257.4019	24.2945	150.5015	20.5284	13.0042	41.6926	0.0087	61.8276	41.2107
TARGET-50-PAKMSV	14.7281	2.9640	32.7288	6.8775	7.3114	30.4292	64.5481	1.1083	62.6357	45.3212	0.6471	147.7661	15.7029	51.6350	8.0514	10.7477	37.4529	0.0073	17.0880	23.2417
TARGET-50-PALLFB	32.0295	6.0358	56.6785	5.3844	8.1088	53.9182	158.1322	2.3697	136.6388	76.1459	1.4345	205.0667	15.9969	62.0278	14.3487	10.5195	39.4964	0.0082	40.3187	15.3485

Supplementary table 1 continue9

	BPD-00008900_1998	Foretinib_2040	BIBR-1532_2043	Pyridostatin_2044	AMG-319_2045	MK-8776_2046	Ulixertinib_2047	Vinorelbine_2048	VX-11e_2096	Uprosertib_2106	LJI308_2107	AZ6102_2109	GSK591_2110	VE821_2111	AZD6482_2169	AT13148_2170	BMS-754807_2171	JQ1_2172
TARGET-50-PAJNAV	169.3175	2.4620	172.2698	76.5622	149.5155	42.1959	15.0341	0.4116	24.0339	13.1896	297.4707	18.9461	176.0005	103.3759	22.8950	49.5308	0.2977	16.1026
TARGET-50-PAJPEW	143.1903	1.6762	180.9539	31.4162	138.9018	38.1249	9.6377	0.0159	5.0424	9.6537	120.8850	9.9886	77.2627	65.1661	24.3076	47.8558	0.7460	13.7013
TARGET-50-PAJMLZ	133.0870	5.2011	164.0376	35.4144	112.7090	57.9388	11.6557	0.1419	59.3741	38.9246	305.8415	17.4567	133.6732	118.3100	29.7578	119.6681	3.5937	10.8295
TARGET-50-PAKRVH	82.2083	3.3080	152.3152	34.5598	115.1639	35.7271	10.8137	0.0630	32.0315	45.0332	146.5047	12.6853	77.1471	61.3074	25.3601	47.4188	1.2942	6.5130
TARGET-50-PADZUB	107.4342	2.3202	186.6170	35.4611	140.3593	35.8407	6.2855	0.0195	7.4882	40.8140	173.0811	9.8985	101.0601	54.5339	43.1245	68.0077	1.6753	12.9774
TARGET-50-PADXAY	156.0449	3.4485	232.9299	27.8649	180.7471	31.0683	11.6374	0.0462	9.6299	8.4219	206.4997	10.1446	121.4810	55.1518	22.7377	16.7825	2.4057	19.0413
TARGET-50-PAJPCM	58.8608	3.1577	126.4622	50.6063	191.4884	18.0846	4.2434	0.0495	11.6375	57.8644	133.7491	9.7344	38.7708	49.5680	36.1652	83.9968	3.3377	31.0385
TARGET-50-PAJPAR	103.7953	4.6188	159.5466	58.1062	194.4001	34.9345	10.5610	0.5131	42.5108	61.0509	268.0270	12.6584	114.9636	93.9271	30.6828	98.2005	3.0586	25.5266
TARGET-50-PAJNZS	102.6820	3.5992	191.3777	57.3107	173.0296	38.6887	12.0261	0.1272	18.8484	45.6787	252.7307	18.4869	104.7644	127.1160	33.1903	130.8819	3.0547	25.0909
TARGET-50-PAJMVC	104.4027	3.1572	56.0154	22.1155	94.5476	18.7773	10.1249	0.0249	20.5620	0.6075	94.6528	14.2224	39.6067	37.0930	13.3560	12.6661	1.6710	10.2482
TARGET-50-PALGAZ	85.1589	2.6700	156.8925	26.2649	118.0488	24.0638	8.3520	0.0753	17.4828	22.5625	198.1105	11.5570	123.4555	86.7103	27.4729	67.9499	1.3742	8.0165
TARGET-50-CAAAAR	97.9556	1.2579	127.9987	22.5546	83.7356	31.2615	9.4145	0.0385	16.2019	10.1986	124.4038	9.4430	95.7399	78.4788	26.9987	34.8152	0.8730	7.8157
TARGET-50-PAKGMU	81.1059	3.0263	187.6357	41.5974	207.0998	47.6561	10.0081	0.0708	18.0733	27.5780	244.3840	15.6273	123.6272	80.5217	28.5734	48.0093	2.4451	21.1601
TARGET-50-PAKRCC	71.2553	3.0277	108.8314	24.2245	95.7420	9.4668	8.5841	0.0641	19.9294	19.2772	138.1291	10.0213	120.0241	51.3722	28.6243	39.8855	2.6046	7.2550
TARGET-50-PAKFME	93.6820	1.8204	157.4290	36.6349	107.9851	19.2632	8.2873	0.0375	14.9187	20.9452	143.7856	9.6686	88.4905	40.6602	28.6049	43.6105	1.3990	8.7052
TARGET-50-PAJNNC	89.7186	2.5347	198.6893	31.2109	94.5964	16.4035	6.9265	0.0682	11.5515	15.9696	145.4956	10.2704	90.3353	82.7353	24.8104	74.6375	1.3751	6.3363
TARGET-50-PAJNJJ	200.9417	4.0951	209.2130	56.4735	202.5561	66.7115	10.4424	0.1954	24.1587	36.8164	259.6882	13.0560	211.0841	163.2263	31.5135	98.0937	2.9599	15.9339
TARGET-50-PAKJGM	47.4982	2.7096	76.3845	10.0731	87.1127	11.7503	4.3340	0.0053	6.8134	11.2839	115.9771	7.7205	43.8559	23.1633	17.4114	10.2784	1.6035	17.5986
TARGET-50-PAJNGH	90.2519	4.2995	136.4178	19.4318	109.0406	26.8903	12.0829	0.0791	26.9263	36.5556	158.9035	13.2633	83.9638	74.0885	26.5720	52.5618	2.0989	5.3178
TARGET-50-CAAAAC	124.7449	10.9376	117.4912	13.7078	131.5187	9.1288	8.6527	0.1383	36.7149	16.5676	159.3585	15.4248	90.0846	36.8608	36.6261	56.5899	2.2893	5.7425
TARGET-50-PAJNBN	57.4484	1.5865	85.7239	20.6491	80.7360	13.4359	7.3003	0.0341	19.6225	35.3790	143.6257	9.6628	72.8552	37.8573	34.1512	47.4412	5.1061	9.1286
TARGET-50-PAKYLT	108.6109	3.5150	135.7163	33.0462	114.4278	15.1491	15.1993	0.0559	42.0208	10.2192	132.9783	12.9891	126.5876	67.9996	22.8998	57.6212	0.5109	8.0387
TARGET-50-PAJMKN	102.0237	2.6978	167.7421	51.1755	124.1944	76.1729	12.6650	0.2255	33.2114	22.4936	248.4511	13.2670	135.9157	128.9043	25.5578	38.6538	1.2334	17.7296
TARGET-50-PALERC	63.8176	1.5105	102.4210	20.3771	77.3299	19.0788	6.8453	0.0363	14.5656	16.1569	147.4598	10.3525	64.1699	33.0380	23.1683	31.6849	1.8939	9.2750
TARGET-50-PAJMFU	87.2643	2.7424	122.8370	30.9234	121.6981	24.9579	8.3564	0.0479	17.6125	52.0562	217.7650	10.5194	115.9600	70.7362	32.1531	59.5423	2.2496	18.0638
TARGET-50-PAKZHF	87.6805	1.5515	157.2176	28.3320	106.5732	32.0288	11.0972	0.0182	6.5975	13.2958	119.9617	7.3044	83.6134	77.1866	36.8324	47.8362	0.8211	12.6786
TARGET-50-PAJLSP	131.8170	4.6322	130.9225	16.1652	128.5794	20.4650	10.2396	0.0276	38.3248	39.3541	126.9267	10.7094	79.5356	46.7825	22.3087	38.4316	1.3226	3.9017
TARGET-50-PAJMKI	62.1645	2.2032	105.2023	21.2168	144.6624	19.5285	9.9010	0.0587	16.4356	22.6884	200.2862	12.5684	103.3085	58.6686	24.7953	26.3556	2.0159	9.6995
TARGET-50-PAKUIT	89.1209	4.5827	136.8176	30.4436	150.9988	11.6592	5.3654	0.1812	27.3429	31.4852	209.1323	14.8010	83.8014	35.2285	30.5241	75.7774	3.0685	8.4619
TARGET-50-PALDWP	54.4221	1.6774	104.2366	15.9211	94.0647	12.0512	5.7174	0.0175	14.7975	27.2437	122.6613	8.3518	67.8630	33.0312	32.8220	38.6753	2.3680	10.3242
TARGET-50-PAJMJT	98.9245	4.7233	144.6502	15.1558	115.4405	28.2547	6.8491	0.0210	14.3888	17.1191	180.1081	10.2420	77.2527	53.0891	36.1778	44.1665	0.7202	8.9762
TARGET-50-PAKWPM	87.5440	2.1710	134.9441	23.5385	159.5810	29.1856	7.3063	0.0176	11.8230	10.8100	132.1861	8.0124	107.6926	52.4503	32.1546	28.7711	2.1616	12.0156
TARGET-50-PAJLKC	69.1613	3.7171	129.3122	15.2571	105.8938	11.1819	8.4309	0.0326	23.3029	16.3773	110.5480	10.6180	121.0282	45.9027	33.4760	26.4713	1.0069	8.0691
TARGET-50-PALKRS	123.3485	3.0331	158.2428	22.6030	162.1523	35.0840	12.0913	0.0097	13.0802	3.1414	135.5002	8.6799	77.3204	59.5545	18.7125	7.9038	0.8917	13.2004
TARGET-50-PAKNRX	156.5482	8.0765	279.6418	67.8328	256.3411	160.7758	15.8961	0.5277	28.4778	127.1446	399.9765	20.1346	167.6753	281.6083	34.4195	75.0095	4.2361	19.9548
TARGET-50-PAJNTJ	79.6507	4.0429	163.1980	25.2448	133.4920	67.8652	8.8196	0.0439	25.5110	21.3903	179.1721	14.4493	116.9975	108.5915	32.8005	39.4568	0.9440	8.5418
TARGET-50-PAJNUS	90.9913	2.2416	121.6666	16.0814	111.7409	21.7118	8.1706	0.0186	14.7120	11.1286	156.3302	8.5629	76.7915	47.2350	37.6196	32.1019	0.6143	12.2815
TARGET-50-PAKNTW	39.9649	0.8286	84.9186	12.8574	68.4243	3.6301	3.4175	0.0052	2.4318	12.1635	55.3015	6.2690	30.6752	12.9598	26.8690	11.7571	1.1054	14.4580
TARGET-50-PAJPAU	94.2061	5.0449	139.4401	40.2557	102.1505	23.9840	14.5534	0.1040	22.3595	59.0881	160.5280	18.8916	151.1164	56.2097	29.8678	60.5441	2.5739	7.0555
TARGET-50-PAJMVU	109.2257	4.1640	186.4694	34.7932	166.3350	33.8137	12.2504	0.0253	15.9112	30.2458	267.5784	11.3674	164.7692	79.4732	40.2542	74.1808	2.9877	25.8059
TARGET-50-PAJNZI	65.3344	4.2135	91.7089	36.4481	135.8643	14.5198	4.9925	0.0844	21.0070	30.5255	158.4421	10.7111	77.1088	45.8548	30.7286	60.0736	3.4712	16.6323
TARGET-50-PAJPDN	117.0254	3.7236	121.4068	23.6597	118.6287	27.2010	9.6252	0.0254	16.8709	5.7121	84.5663	8.6095	49.9817	53.3720	13.2027	11.8547	1.5303	11.3908
TARGET-50-PAJNVE	67.7690	2.9382	92.1003	25.6175	94.9339	6.4749	9.1891	0.0487	30.8705	35.7141	102.5272	11.4091	56.2878	27.5290	31.6293	50.4587	2.7658	7.1556
TARGET-50-PAJMSE	87.4731	2.6227	119.4464	33.4600	102.4253	16.5156	12.4938	0.0796	22.1478	20.5795	142.9988	9.0433	113.7576	61.0846	29.7655	81.4264	1.3700	11.6513
TARGET-50-PAJNZU	104.4183	1.5248	143.3035	26.8288	105.3972	37.2331	11.5392	0.0759	22.4274	24.8494	141.4306	9.9360	115.1033	66.2100	26.4491	47.3693	1.6429	4.5264
TARGET-50-PALGLU	90.9501	1.2883	119.7435	25.8591	114.2064	13.4624	7.5215	0.0207	7.9462	3.9955	91.3179	6.9127	71.4746	30.0669	22.9539	10.8631	1.1758	8.5636
TARGET-50-PAJNZK	98.2439	4.3931	139.2775	36.3487	116.1060	11.7712	7.7019	0.0851	18.3998	16.3192	162.5455	11.1770	109.2359	41.0900	27.1315	71.3450	3.5095	6.2940
TARGET-50-PAJMUF	71.6313	1.0154	104.5937	15.7043	80.5309	25.2284	7.7062	0.0103	8.0478	9.1440	115.6812	10.3364	65.3104	31.5576	25.3184	16.7868	0.8044	8.8491
TARGET-50-PAJMRL	102.1033	4.5089	137.8012	24.4517	194.4890	14.3193	9.2215	0.0221	28.4097	59.2704	200.9028	19.1796	107.4094	73.7722	23.5074	86.0106	1.5868	16.2946
TARGET-50-PAJMXF	82.4563	3.9747	106.0501	20.7805	105.0914	35.1396	8.8940	0.0432	24.0519	15.8212	174.1867	10.1969	102.1003	70.5583	23.3176	23.7884	2.8975	10.3660
TARGET-50-PAKPDF	78.2951	2.0377	123.4541	37.7559	120.6306	20.9525	9.5806	0.0829	28.9919	32.4044	146.8789	8.7162	80.1706	44.2480	33.7813	46.8374	1.9008	8.0082
TARGET-50-PAEBXA	93.8601	1.7537	154.7886	31.7653	122.4125	23.1928	11.3137	0.0451	14.5348	11.9021	196.2420	12.7285	113.6274	72.5368	31.0470	38.7927	1.5232	16.8121
TARGET-50-CAAAAS	140.5022	9.0180	134.3420	23.1941	105.9123	17.2888	12.0173	0.1692	36.8862	25.1141	150.2484	16.1967	116.9989	91.9429	27.7480	66.7209	1.4662	4.5071
TARGET-50-PAJNLT	110.6941	2.8953	109.8098	20.4340	83.0024	14.7391	8.4007	0.0357	20.8355	20.7985	123.2700	11.2351	58.2487	43.1832	22.1592	41.6433	3.4433	6.1540
TARGET-50-PAJPHA	97.1283	1.8356	157.1603	18.0978	117.8197	18.3514	9.3615	0.0583	19.3878	7.8139	170.4982	13.0207	74.1868	50.9070	19.9832	40.7478	1.1999	7.0686
TARGET-50-PAJMMY	67.5759	1.9660	89.9622	18.1985	81.1906	12.4686	9.9574	0.0423	25.9659	7.6577	121.1850	7.8997	68.5797	45.2757	20.1355	22.8689	2.6242	3.7905
TARGET-50-PAJLTH	100.6338	3.6633	130.2824	22.8636	139.8536	18.0066	5.5257	0.0286	9.1733	11.9016	127.2220	11.9744	137.3421	57.0725	32.3650	48.6579	1.1378	7.8086
TARGET-50-PAJNCC	80.0298	1.7317	191.9975	21.6240	125.8354	3.0072	5.1967	0.0395	8.8838	9.0417	141.2823	8.4608	97.5868	22.3470	15.2402	74.0021	1.7500	4.0179
TARGET-50-PAJLUJ	53.1498	0.9203	102.6525	11.7914	77.1963	15.2653	7.3310	0.0090	7.8120	3.8108	105.8063	9.0922	59.2149	27.7150	28.8987	14.7783	0.4732	17.1367
TARGET-50-PAJNCJ	95.6853	1.4368	130.4926	26.4365	85.3773	24.1965	8.0948	0.0918	22.0673	18.9877	157.9721	12.1263	104.6062	101.4544	25.9197	43.8420	0.9232	6.1262
TARGET-50-PAKZER	102.2339	1.0783	148.1525	26.9767	138.5461	10.7876	8.4839	0.0368	10.1446	4.1786	137.6546	11.9584	99.6895	32.1366	31.6492	25.9834	1.5190	12.0713
TARGET-50-PAJMEP	75.7651	1.9149	97.1615	19.3594	77.4155	19.0890	10.3415	0.0452	29.6780	18.3525	136.9323	10.5060	94.1659	66.3212	21.8730	31.0340	1.9364	4.4945
TARGET-50-PAJMEL	94.3535	1.1116	121.2118	24.4727	90.4862	32.4923	9.3364	0.0334	11.3860	8.7545	120.2728	8.7200	99.4275	87.6948	23.4055	37.7629	0.4404	5.7007
TARGET-50-PAJNVX	184.9725	3.8489	308.3108	66.0712	215.5224	54.4810	17.1566	0.9567	40.5200	8.9284	349.7401	40.7093	239.9582	248.5395	25.4097	104.6494	0.6945	10.9836
TARGET-50-PAKXWB	57.0105	1.8777	127.6884	23.2492	101.8326	15.1384	15.3991	0.0369	14.1201	7.7347	181.1724	14.3651	95.6328	53.6070	17.9331	23.1666	1.2632	12.7238
TARGET-50-CAAAAB	74.8393	3.9293	104.9852	20.8344	138.1071	21.2341	9.4141	0.0490	27.0087	19.8917	135.5249	14.4188	100.4361	75.6958	30.0456	29.9929	2.1712	7.9603
TARGET-50-PALJIP	73.2693	2.1639	140.7302	25.1277	111.0677	12.0359	6.2730	0.0403	5.6078	5.7254	100.5950	9.4685	76.3393	39.6694	24.1076	26.5941	1.3125	7.7532
TARGET-50-PAKMUB	115.9954	1.2582	232.5289	35.9470	146.4624	38.5195	14.6250	0.1384	17.4172	4.9794	174.4759	16.3984	191.4463	170.2181	26.4569	42.6381	0.1338	11.2363
TARGET-50-PALKCW	76.1407	1.6588	144.0194	30.7967	129.1997	30.5472	7.8228	0.0334	11.2890	14.9480	192.3587	8.5600	113.7565	64.1744	26.0575	29.7122	1.7416	18.2408
TARGET-50-CAAAAQ	67.9701	1.9253	71.4228	12.4730	90.0009	13.2339	4.8193	0.0114	11.8347	11.1655	100.1858	7.5274	43.5818	25.6962	30.6508	18.1383	2.4457	11.5305
TARGET-50-PALFME	92.7819	3.3615	136.2623	18.5371	128.4125	9.5575	8.2980	0.0350	14.6735	42.6851	197.6858	12.5180	79.2908	36.2610	37.2835	76.1682	1.9025	10.4201
TARGET-50-PAKNXS	144.0444	4.2046	206.3667	65.4004	300.8319	43.4830	19.3243	0.1791	33.2128	43.2213	226.5339	11.5936	137.6161	79.4902	26.2054	74.4369	2.3932	17.8625
TARGET-50-PAJNAA	268.5333	8.6719	473.2783	167.2909	324.3806	160.8357	21.0163	1.7088	80.0177	158.8597	412.6703	40.0647	267.1585	488.8259	28.7478	229.7329	0.3768	16.2367
TARGET-50-PAJPGY	113.7320	1.3269	141.5596	21.7650	129.7022	13.2689	8.4637	0.0455	14.4274	4.6411	192.6042	10.7448	114.8564	59.0120	21.8674	14.6935	1.0279	8.6284
TARGET-50-PAJNEC	100.1765	8.3693	159.7599	18.3351	241.9877	39.8129	9.3172	0.0037	6.9943	3.7721	127.4270	8.5927	67.4642	38.7134	17.6477	6.3531	5.2265	18.0808
TARGET-50-PAJNDU	57.4732	2.8951	89.9758	12.5529	105.5177	18.5726	4.3782	0.0032	4.9033	0.8794	65.6066	6.6284	29.6116	13.8901	11.2560	1.7715	4.0715	11.6920
TARGET-50-PAKGZX	57.4086	0.8904	189.2393	33.4333	114.1439	13.8380	9.3300	0.0445	5.7725	13.7947	137.8509	9.5727	111.6967	72.5989	24.5852	25.0518	1.6551	13.7599
TARGET-50-PAKFYV	67.5237	3.0669	128.8916	48.3376	181.3058	24.8875	7.4421	0.1579	53.3619	146.7420	244.6920	12.0956	87.2606	47.5329	20.6736	110.0725	2.8268	14.6759
TARGET-50-PAKGED	142.8001	5.1942	129.0853	33.7023	150.6464	23.9050	12.7746	0.0048	4.9291	0.6936	99.5199	6.1847	55.9794	42.6204	15.4492	1.8257	4.3769	10.5536
TARGET-50-PAJLTI	108.2145	2.4494	149.5907	25.3217	123.0563	34.1046	14.0605	0.0463	35.6113	20.5075	178.0690	13.4606	110.2766	85.9292	25.8705	47.5645	0.8416	6.7644
TARGET-50-CAAAAO	70.7986	1.7326	102.9792	24.6796	96.7466	17.5830	5.2649	0.0209	8.3818	18.4972	160.4731	8.6056	76.4699	32.8019	27.7319	29.0616	2.2631	18.4925
TARGET-50-PAJNCZ	76.4167	1.6771	117.8003	24.6568	107.6879	30.4393	8.6944	0.0421	20.9387	21.2859	185.4256	9.2616	92.1318	66.5166	25.7136	33.6932	1.9494	6.6775
TARGET-50-PAJMIZ	73.2176	3.1783	122.9272	15.0914	132.1669	48.7903	6.5069	0.0183	15.7401	20.6861	164.2899	10.6233	79.8703	55.8651	24.8830	22.2927	1.5765	16.8474
TARGET-50-PAJMFY	74.0139	2.3728	116.8242	28.2007	94.6962	30.4306	9.8827	0.0848	30.1915	41.9453	200.4408	12.5141	118.3804	85.6539	27.3806	60.7557	2.4186	10.6786
TARGET-50-PAKULH	88.7725	3.1532	103.2302	14.9448	117.3469	8.1948	8.1965	0.0191	10.6934	8.0549	140.4959	8.2015	112.8320	26.1691	30.5690	29.0899	1.3037	17.5301
TARGET-50-PAKVET	159.0871	5.5624	278.8126	112.0286	152.4177	109.9246	24.6831	2.9590	66.1921	85.6966	349.2826	17.1536	233.1499	167.1803	30.6093	105.3230	1.4728	14.3582
TARGET-50-PAJMEN	71.3924	2.1281	112.3829	19.0365	92.5386	26.8795	5.3969	0.0199	21.0873	10.1063	173.3503	10.4632	89.8557	53.1860	24.6255	22.4500	1.8735	15.0973
TARGET-50-PALDTE	88.1192	2.2789	105.7944	29.6090	101.4708	38.3808	9.3167	0.0548	21.4925	34.3679	147.5167	8.3341	92.8554	62.7187	27.3613	45.6795	1.7025	5.9816
TARGET-50-CAAAAH	83.3486	4.5050	95.4188	21.3614	187.4734	13.0454	4.2843	0.0047	5.7174	3.5360	102.7846	9.9000	36.4811	28.6285	16.2755	6.2764	3.0432	24.8579
TARGET-50-PAJNRH	79.3811	3.4584	184.4748	45.1354	132.4317	39.0908	10.6194	0.0702	16.2307	32.3886	220.9109	12.1876	121.9515	51.8020	33.3540	46.2063	4.1493	27.9389
TARGET-50-PAJNRL	87.1797	3.4949	116.2506	24.8920	133.6769	18.2789	6.6866	0.0364	13.4350	32.9593	167.2055	10.3359	95.5741	49.9216	32.0934	60.6795	2.0583	12.0766
TARGET-50-PAJNUP	145.9843	4.3696	369.2846	114.3387	279.5911	38.8172	23.9182	0.1467	22.9568	12.8420	179.1296	37.3329	188.7840	264.9226	18.4121	108.7127	0.3928	12.0674
TARGET-50-PAJPDC	76.6448	2.1240	127.4908	28.9386	103.8024	18.5032	6.5431	0.0462	5.4486	20.9743	140.0586	10.6189	101.1581	51.2355	34.6621	27.9446	0.9130	17.9061
TARGET-50-PAJMLI	79.2226	1.8919	114.3984	26.7111	109.1103	32.3145	10.2998	0.0344	27.1797	12.0444	152.9534	10.8438	99.3962	78.7794	25.1515	39.8236	1.5406	6.3022
TARGET-50-PAJLWT	77.2975	1.9785	134.6315	27.4475	153.2508	39.9968	8.6451	0.0156	12.0917	14.9972	191.2954	8.0882	88.8127	48.4425	24.3057	29.4678	1.4338	20.8225
TARGET-50-PALEZT	65.5621	2.6770	133.9755	26.4315	100.0969	22.3840	9.6689	0.0484	29.2689	21.1846	160.4251	13.1001	89.9725	57.1750	21.7938	24.1964	2.4631	15.8248
TARGET-50-PAJLLF	106.3377	5.0075	213.6686	47.9248	272.2694	86.9930	11.3334	0.0602	31.2291	89.1503	307.0819	27.5669	204.7104	108.5987	32.9972	107.6843	0.6998	17.2072
TARGET-50-PAJLNJ	69.3000	0.9858	106.8971	17.9268	83.0421	15.5106	5.9886	0.0124	7.8975	11.2152	141.3198	9.5310	66.9246	32.4572	23.9904	22.9149	1.5070	10.7043
TARGET-50-PALFRD	66.7474	2.5645	127.7790	31.5674	128.0597	15.0288	7.6715	0.0243	11.7958	6.7673	172.3318	8.4743	96.7169	45.5664	16.6358	14.5451	2.6329	17.1238
TARGET-50-PAKKSE	92.0007	1.1335	160.4774	24.7845	117.4242	32.3697	9.1443	0.0232	11.5579	9.4562	147.3307	12.0414	108.8603	76.7691	24.6316	29.6102	0.8687	10.8267
TARGET-50-PAJLPX	108.4659	3.4775	185.0646	58.7601	183.1105	15.7492	9.2424	0.1332	15.7266	19.0707	154.2506	12.8875	99.0579	55.0338	28.5635	63.8023	2.1048	7.6168
TARGET-50-PAJMKJ	87.8251	2.7765	175.2742	24.6377	135.8993	10.2928	8.3618	0.0898	10.7519	5.5314	152.1969	9.4810	117.9725	30.5307	28.7735	29.8919	1.1397	15.3495
TARGET-50-PAKSDG	75.8611	2.1430	123.2271	21.0729	108.5836	18.8178	11.9480	0.0446	22.0862	18.8173	160.6330	10.1890	83.4406	51.8357	26.0406	51.4276	2.8965	14.0809
TARGET-50-PAKZFK	84.1608	1.4580	145.7538	22.6059	116.9101	28.2195	7.3328	0.0302	15.6112	15.0988	157.0350	9.3059	107.5252	49.9468	33.0336	53.3288	0.9476	13.1858
TARGET-50-PAKYFC	111.5333	1.8502	131.0024	30.1891	104.1520	26.8041	9.2366	0.0600	20.0078	24.6696	174.7650	11.9025	106.7881	80.5659	27.8743	55.2506	1.1842	6.2184
TARGET-50-PAJNNR	92.4239	3.0443	142.0600	40.5213	233.9930	64.8479	11.7208	0.0255	20.3443	40.1535	271.4792	16.5755	129.4044	84.8962	25.4032	121.6416	1.5834	12.6502
TARGET-50-PAJLIP	126.8828	8.9221	367.4210	93.2975	223.1671	29.8423	25.1312	0.1629	60.8187	48.7895	280.3934	16.2617	125.2105	144.6221	33.7276	187.0472	3.0098	15.4174
TARGET-50-PAJNYT	162.6712	1.7316	135.6458	31.8661	107.4276	36.5941	15.6569	0.1849	37.7424	10.2910	144.7871	12.5561	147.5508	143.0883	17.1166	60.1348	0.1626	2.9480
TARGET-50-PALGVY	77.4905	1.2925	139.0306	30.5855	99.6630	20.9745	6.6089	0.0135	5.3508	10.1237	121.5947	7.6138	95.1138	38.2718	33.3594	26.9270	0.8402	18.9982
TARGET-50-PAECJB	68.5611	1.0852	92.7667	16.1228	63.2271	21.1527	7.4429	0.0231	13.8743	9.6639	115.3203	10.1280	66.4688	39.4446	26.8764	20.6706	0.6810	6.3638
TARGET-50-CAAAAM	223.9450	5.5580	312.3233	124.7415	304.6454	20.4819	18.6821	0.1219	19.3754	2.4383	118.2671	31.6263	211.0589	293.0125	8.6755	58.2371	0.0065	3.6604
TARGET-50-PAKSCC	60.2082	1.7549	97.1687	15.9936	101.3068	18.9415	5.8760	0.0164	14.0647	8.8815	133.4643	10.6647	63.5866	23.2847	24.7489	17.0524	2.0892	13.4730
TARGET-50-PAKRZW	104.8828	2.4790	153.1181	38.6383	153.0870	50.4930	11.5917	0.0385	16.8082	4.2924	177.2278	17.3460	109.3722	92.9885	19.3827	15.3258	0.2730	11.2492
TARGET-50-PAJNSL	74.2712	1.6011	103.6807	23.2989	72.9654	24.3328	7.4836	0.0584	19.8384	16.5786	137.2137	10.5045	72.2875	48.6108	24.5400	39.0574	1.5216	7.1854
TARGET-50-PAKKNS	117.8919	3.8754	214.3716	67.3822	206.3498	37.6288	12.9591	0.1169	17.3206	30.6856	258.7744	14.4787	192.1389	127.4041	31.5910	67.8437	2.6368	15.0281
TARGET-50-CAAAAL	111.9469	3.0339	132.3130	36.0380	123.2876	32.2091	11.4367	0.0843	24.6650	57.1004	227.8542	10.2519	137.1768	107.6090	33.4139	108.2726	2.0040	12.7642
TARGET-50-CAAAAJ	73.9893	1.0859	118.9479	19.0274	93.3829	15.9100	5.9881	0.0129	8.8909	20.1477	128.9789	8.9625	66.9794	37.2515	26.8525	38.4517	1.8552	10.1215
TARGET-50-PAKNAL	134.2654	2.4129	189.1676	30.0046	154.5297	28.2434	12.1864	0.0514	26.8900	11.0417	160.5125	12.4547	136.9063	102.2393	26.3262	43.5564	1.0139	7.4638
TARGET-50-PAKXXF	73.4072	3.1544	101.5932	36.2651	151.0082	11.4981	5.0742	0.0126	11.0004	27.5791	101.9494	14.5517	35.3300	24.7786	20.5661	17.7047	2.8819	34.0032
TARGET-50-PAKECR	148.6452	1.8728	213.8003	61.7930	198.4554	17.6777	9.7110	0.0440	8.8864	6.3471	154.7690	15.4474	202.3162	65.8096	36.1329	39.5626	0.4480	15.9738
TARGET-50-CAAAAP	94.2488	2.3689	182.9916	34.6259	149.5213	16.7669	6.8214	0.0798	31.5688	27.8455	180.0099	10.5274	160.6059	54.4456	31.3313	64.6267	1.9523	8.4291
TARGET-50-PAEAFB	94.7411	1.8497	137.4156	32.4633	146.7301	23.8759	11.0997	0.0231	15.4091	25.0809	168.8315	9.4736	90.8585	49.9975	34.2961	56.6885	1.6052	22.8731
TARGET-50-PAJLKR	149.9184	6.6220	262.4955	32.3862	164.3257	23.8911	5.4230	0.0398	9.7867	36.9605	246.4059	10.3139	114.5957	62.1777	29.8543	74.2913	2.8695	10.4624
TARGET-50-PAKMSV	51.4192	2.2179	94.9861	9.2808	103.4758	8.9973	5.8573	0.0060	7.8545	8.6147	84.8390	6.8331	37.4630	17.9642	18.5374	10.3641	2.4174	18.9402
TARGET-50-PALLFB	72.3296	3.5364	147.3225	37.0944	157.5919	21.7941	9.3055	0.0774	25.3920	40.3736	235.7401	10.0783	105.5194	90.8850	30.0358	86.1599	4.4920	10.1447
