# Supplementary material for: Development of a Multilayered Prognostic Model for Wilms’ Tumor Based on Characteristic Lymphocyte Genes
Source: Genet Res (Camb). 2025 Dec 18;2025:1964582. doi: 10.1155/genr/1964582 (PMC12721763; doi:10.1155/genr/1964582)
Supplement: Supplementary file 1 — Supporting Information Additional supporting information can be found online in the Supporting Information section. [file GENR-2025-1964582-s001.zip › supplementary table 2.docx]

Supplementary Table 2. P values corresponding to the drug-sensitivity.

| Drug | *P*-Value |
| --- | --- |
| MIM1 | 0.0001 |
| Sepantronium bromide | 0.0004 |
| OSI-027 | 0.0008 |
| IAP_5620 | 0.0010 |
| Crizotinib | 0.0019 |
| LCL161 | 0.0052 |
| AZD5582 | 0.0063 |
| BDP-00009066 | 0.0065 |
| Camptothecin | 0.0077 |
| Alisertib | 0.0100 |
| XAV939 | 0.0120 |
| Paclitaxel | 0.0122 |
| BPD-00008900 | 0.0122 |
| AZ960 | 0.0124 |
| Luminespib | 0.0127 |
| Irinotecan | 0.0148 |
| MK-1775 | 0.0165 |
| PFI3 | 0.0172 |
| Docetaxel | 0.0181 |
| Docetaxel | 0.0181 |
| Pyridostatin | 0.0204 |
| GSK343 | 0.0232 |
| IWP-2 | 0.0261 |
| Talazoparib | 0.0278 |
| Epirubicin | 0.0315 |
| Buparlisib | 0.0315 |
| Teniposide | 0.0327 |
| Niraparib | 0.0335 |
| BMS-754807 | 0.0347 |
| Picolinici-acid | 0.0374 |
| WIKI4 | 0.0378 |
| Cytarabine | 0.0401 |
| Obatoclax Mesylate | 0.0421 |
| Leflunomide | 0.0447 |
| Vinblastine | 0.0452 |
| Wnt-C59 | 0.0463 |
| Ruxolitinib | 0.0502 |
| Ribociclib | 0.0532 |
| AT13148 | 0.0532 |
| WEHI-539 | 0.0550 |
| PCI-34051 | 0.0576 |
| ML323 | 0.0582 |
| Gemcitabine | 0.0589 |
| Mitoxantrone | 0.0629 |
| BMS-345541 | 0.0673 |
| Temozolomide | 0.0687 |
| Gallibiscoquinazole | 0.0695 |
| AZD7762 | 0.0703 |
| YK-4-279 | 0.0703 |
| Afuresertib | 0.0726 |
| Dinaciclib | 0.0734 |
| LY2109761 | 0.0742 |
| BIBR-1532 | 0.0750 |
| GDC0810 | 0.0774 |
| Mirin | 0.0800 |
| Topotecan | 0.0800 |
| Cediranib | 0.0800 |
| Elephantin | 0.0817 |
| Eg5_9814 | 0.0843 |
| Pevonedistat | 0.0861 |
| Vinorelbine | 0.0888 |
| Afatinib | 0.0898 |
| Vincristine | 0.0898 |
| Venetoclax | 0.0936 |
| UMI-77 | 0.0985 |
| CZC24832 | 0.0995 |
| Wee1 Inhibitor | 0.1016 |
| Savolitinib | 0.1047 |
| IGF1R_3801 | 0.1079 |
| KU-55933 | 0.1090 |
| Fulvestrant | 0.1101 |
| Fulvestrant | 0.1101 |
| AZD1332 | 0.1146 |
| AZD6738 | 0.1203 |
| AZD5363 | 0.1215 |
| Vorinostat | 0.1251 |
| ULK1_4989 | 0.1326 |
| KRAS (G12C) Inhibitor-12 | 0.1364 |
| Ibrutinib | 0.1378 |
| Erlotinib | 0.1391 |
| PAK_5339 | 0.1417 |
| I-BET-762 | 0.1444 |
| Telomerase Inhibitor IX | 0.1444 |
| MN-64 | 0.1485 |
| Podophyllotoxin bromide | 0.1499 |
| VE-822 | 0.1542 |
| BI-2536 | 0.1571 |
| AZD4547 | 0.1614 |
| Staurosporine | 0.1674 |
| Entospletinib | 0.1736 |
| Tamoxifen | 0.1767 |
| Dactinomycin | 0.1767 |
| Dactinomycin | 0.1767 |
| CDK9_5576 | 0.1848 |
| PRIMA-1MET | 0.1881 |
| EPZ5676 | 0.1931 |
| Dabrafenib | 0.1982 |
| Sapitinib | 0.1982 |
| AZD6482 | 0.1999 |
| Alpelisib | 0.2017 |
| AGI-6780 | 0.2017 |
| GSK2606414 | 0.2088 |
| P22077 | 0.2142 |
| GSK1904529A | 0.2272 |
| 5-Fluorouracil | 0.2368 |
| Oxaliplatin | 0.2408 |
| Oxaliplatin | 0.2408 |
| Nelarabine | 0.2428 |
| I-BRD9 | 0.2448 |
| VE821 | 0.2468 |
| Sinularin | 0.2488 |
| VSP34_8731 | 0.2508 |
| Ulixertinib | 0.2508 |
| Ulixertinib | 0.2508 |
| AZD5991 | 0.2529 |
| AZD1208 | 0.2549 |
| Cyclophosphamide | 0.2633 |
| ERK_2440 | 0.2633 |
| Osimertinib | 0.2654 |
| IRAK4_4710 | 0.2675 |
| JAK_8517 | 0.2739 |
| Pictilisib | 0.2805 |
| PD0325901 | 0.2805 |
| Dasatinib | 0.2805 |
| ERK_6604 | 0.2827 |
| Olaparib | 0.2849 |
| Carmustine | 0.2894 |
| Palbociclib | 0.2917 |
| GSK2578215A | 0.2917 |
| Nutlin-3a (-) | 0.3172 |
| CDK9_5038 | 0.3172 |
| Daporinad | 0.3220 |
| Cisplatin | 0.3367 |
| Tozasertib | 0.3392 |
| Ipatasertib | 0.3392 |
| AZ6102 | 0.3442 |
| RVX-208 | 0.3493 |
| AZD3759 | 0.3648 |
| MIRA-1 | 0.3753 |
| BMS-536924 | 0.3807 |
| GSK591 | 0.3888 |
| MK-2206 | 0.3997 |
| Dactolisib | 0.4053 |
| AMG-319 | 0.4109 |
| Sorafenib | 0.4137 |
| OTX015 | 0.4137 |
| Trametinib | 0.4222 |
| Entinostat | 0.4308 |
| JQ1 | 0.4366 |
| Nilotinib | 0.4425 |
| NU7441 | 0.4425 |
| PRT062607 | 0.4425 |
| AZD5438 | 0.4484 |
| Acetalax | 0.4513 |
| Lapatinib | 0.4693 |
| SB505124 | 0.4754 |
| ABT737 | 0.4754 |
| OF-1 | 0.4846 |
| MK-8776 | 0.4877 |
| EPZ004777 | 0.5033 |
| Gefitinib | 0.5256 |
| Dihydrorotenone | 0.5256 |
| Foretinib | 0.5321 |
| Uprosertib | 0.5517 |
| Uprosertib | 0.5517 |
| PD173074 | 0.5550 |
| Doramapimod | 0.5584 |
| Fludarabine | 0.5584 |
| Bortezomib | 0.5717 |
| NVP-ADW742 | 0.5717 |
| Sabutoclax | 0.5954 |
| VX-11e | 0.6023 |
| LJI308 | 0.6023 |
| GNE-317 | 0.6161 |
| WZ4003 | 0.6266 |
| AZD5153 | 0.6266 |
| Rapamycin | 0.6301 |
| ZM447439 | 0.6549 |
| AZD2014 | 0.6728 |
| AZD8186 | 0.6800 |
| SCH772984 | 0.6837 |
| PF-4708671 | 0.7055 |
| AGI-5198 | 0.7240 |
| Axitinib | 0.7314 |
| GSK269962A | 0.7500 |
| RO-3306 | 0.7538 |
| Linsitinib | 0.7992 |
| LGK974 | 0.8107 |
| JAK1_8709 | 0.8183 |
| MG-132 | 0.8183 |
| Selumetinib | 0.8337 |
| Zoledronate | 0.8415 |
| PLX-4720 | 0.8531 |
| TAF1_5496 | 0.8764 |
| SB216763 | 0.8998 |
| AZD8055 | 0.9350 |
| Taselisib | 0.9429 |
| Navitoclax | 0.9941 |
